# Characterization of the Highly Variable Immune Response Gene Family, *He185/333*, in the Sea Urchin, *Heliocidaris erythrogramma*

**DOI:** 10.1371/journal.pone.0062079

**Published:** 2014-10-21

**Authors:** Mattias O. Roth, Adam G. Wilkins, Georgina M. Cooke, David A. Raftos, Sham V. Nair

**Affiliations:** Department of Biological Sciences, Macquarie University, North Ryde, NSW, Australia; Chang Gung University, Taiwan

## Abstract

This study characterizes the highly variable *He185/333* genes, transcripts and proteins in coelomocytes of the sea urchin, *Heliocidaris erythrogramma*. Originally discovered in the purple sea urchin, *Strongylocentrotus purpuratus*, the products of this gene family participate in the anti-pathogen defenses of the host animals. Full-length *He185/333* genes and transcripts are identified. Complete open reading frames of *He185/333* homologues are analyzed as to their element structure, single nucleotide polymorphisms, indels and sequence repeats and are subjected to diversification analyses. The sequence elements that compose *He185/333* are different to those identified for *Sp185/333*. Differences between *Sp185/333* and *He185/333* genes are also evident in the complexity of the sequences of the introns. He185/333 proteins show a diverse range of molecular weights on Western blots. The observed sizes and pIs of the proteins differ from predicted values, suggesting post-translational modifications and oligomerization. Immunofluorescence microscopy shows that He185/333 proteins are mainly located on the surface of coelomocyte subpopulations. Our data demonstrate that *He185/333* bears the same substantial characteristics as their *S. purpuratus* homologues. However, we also identify several unique characteristics of *He185/333* (such as novel element patterns, sequence repeats, distribution of positively-selected codons and introns), suggesting species-specific adaptations. All sequences in this publication have been submitted to Genbank (accession numbers JQ780171-JQ780321) and are listed in table S1.

## Introduction

Many invertebrate immune systems studied to-date consist of highly complex repertoires of pattern recognition receptors (PRRs), regulatory and effector systems, but lack hypervariable recognition molecules that are homologous to vertebrate immunoglobulins. Recent studies also indicate the ability of some invertebrate immune systems, such as those of some arthropods, to specifically discriminate between different pathogens [1]–[3] and there is also evidence which suggest that some invertebrate immune systems may be capable of heightening responses to repeated challenge by the same type of pathogen [4], [5], a phenomenon analogous to immunological memory [6]. However, the molecular bases of these immunological features have not been established. Some invertebrate immune genes, such as the scavenger receptor cysteine rich repeat (SRCR) genes of the sea urchins [7], are organized as large gene families that specify diverse repertoires of closely-related products [8], [9]. This diversity is presumably brought about by gene duplication and divergence, gene conversion and gene rearrangement during PRR expression [10]. Another strategy involves post-transcriptional diversification of a small number of immune-response genes. An example of this is the down syndrome cell adhesion molecule (Dscam) gene family in *Drosophila melanogaster* and *Anopheles gambiae*, which can generate thousands of alternatively spliced transcripts from single copy genes [4], [11]. Other examples of diversified families of genes in invertebrates include those encoding the fibrinogen related proteins (FREPs) in snails [12] and those encoding the variable chitin binding proteins (VCBPs) in cephalochordates [13].

Sea urchins are members of the echinoderm phylum, which is a sister group to the chordates. Thus, sea urchins are important for investigations of the immunology and the evolution of immune systems in the deuterostome lineage. The purple sea urchin, *Strongylocentrotus purpuratus*, has an elaborately equipped immune system [8]. The numbers of genes encoding putative PRRs (e.g. TLR, SRCR, NOD and NLR) in the *S. purpuratus* genome are much higher than those found in vertebrate genomes [14]. In addition, a unique class of highly variable immune-gene family, known as *Sp185/333*, also functions in immunity and is considerably upregulated upon immunological challenge [15]–[17]. More than 860 full-length *Sp185/333* cDNAs [16], [17] and 171 genes [18] have been sequenced to-date. The diversity of *Sp185/333* s is based on the presence or absence of 25 to 27 sequence blocks, called elements, depending on how the sequences are aligned [15], [17], [19]. Elements do not randomly appear in the genes, but are present in distinct element patterns (i.e. combinations of elements; 15–18) and all *Sp185/333* sequences can be categorized according to their element patterns. Diversity is further enhanced by single nucleotide polymorphisms (SNPs), short insertions and deletions (indels) and a number of sequence repeats that appear throughout *Sp185/333* sequences [15], [18] that enable two equally feasible alignments of the sequences. Previous studies indicated that *Sp185/333* with *E2*, *D1*, *01*, and *E3* element patterns were under positive selection for diversification (dn/ds>1; see Ref. 16). Furthermore, sequences from the first element of all Sp185/333 sequences were under positive selection. However, *Sp185/333* with *C1, A2,* and *E1* element patterns were under negative selection (dn/ds<1). The mechanisms that generate the high variability in *Sp185/333* sequences are unknown. However, gene conversion and DNA recombination driven by microsatellites that flank *Sp185/333* genes have been purported as possible mechanisms that promote gene diversification [19], [20]. Post-transcriptional processing of *Sp185/333* mRNAs is also thought to contribute to diversity after their synthesis [21]. The deduced Sp185/333 polypeptides carry a predicted signal peptide, an N-terminal glycine-rich, a C-terminal histidine-rich region and patches of acidic residues [16]. The Sp185/333 proteins include up to six different types of tandem and interspersed repeats [19] and several conserved N- and O- linked glycosylation sites [16]. Almost all have an RGD motif and lack cysteine residues. *De novo* predictions point to the lack of discernible secondary and tertiary structures, including the absence of known functional domains. Sp185/333 proteins are localized to the cell surface of small phagocytes and are present in peri-nuclear vesicles of both small and polygonal phagocytes [22]. It has been speculated that Sp185/333 proteins may play a role in cell-cell interactions to form syncytia and initiate encapsulation of invading pathogens [22]. Although genome sequencing projects indicate the presence of *185/333* homologues in other sea urchins, only the *Sp185/333* gene family has been characterized to-date [15]–[20], [22]–[25].

We report here the *He185/333* genes, transcripts and proteins in coelomocytes of the sea urchin, *Heliocidaris erythrogramma*. This family exhibits characteristic features of the *Sp185/333* family and the striking sequence diversity of the genes and proteins are common to both families. Full-length *He185/333* genes and transcripts possess element patterns, SNPs, short indels, as well as tandem and interspersed repeats. He185/333 proteins show a broad range of diversity in sizes and pIs and are expressed on the surfaces of some coelomocytes. Although *He185/333* and *Sp185/333* share many attributes, there are a few substantial differences between them. For example, *He185/333* sequences tend to be shorter than *Sp185/333* sequences. *He185/333* sequences also consist of element patterns that are not found amongst the *Sp185/333* sequences. There are also significant differences in intron structures, codon diversity, sequence repeats, amongst others.

## Materials and Methods

All reagents were purchased from Sigma Aldrich or Amresco, unless otherwise indicated.

### Sea urchins

All animals were collected according the rules indicated in the scientific collection permit (permit number F95/403–7.1) issued to Macquarie University by the NSW Department of Primary Industries, Australia. *Heliocidaris erythrogramma* specimens were collected at Clifton Gardens in Sydney Harbour and maintained at 22°C in the Macquarie University sea water facility in 50-liter tubs with continuous recycling of sea water from Sydney Harbor. Sea urchins were fed once a week with fresh algae that was collected from Sydney Harbour. All necessary permits were obtained for the described field studies.

### Immune challenge and extraction of coelomic fluid

As described previously [24], between 0.5 ml and 25 ml of coelomic fluid was harvested from each animal and mixed with an equal volume of ice-cold calcium- and magnesium-free sea water (CMFSW-EI; 460 mM sodium chloride, 10.73 mM potassium chloride, 7.04 mM disodium sulfate, 2.38 mM sodium hydrogen carbonate) with 30 mM EDTA and 50 mM imidazole, pH 7.4. Coelomocytes were pelleted (1200–5000×*g* for 4 min at 4°C) and washed in CMFSW-EI before further processing. When required, sea urchins were challenged with 1.2×10^8^ heat-killed *E. coli* cells (inoculum volume of 0.2 ml) per animal for 24 h or 48 h (for subsequent protein extraction and microscopy) prior to harvesting coelomic fluid.

### Total RNA and DNA isolation

Coelomocytes collected from sea urchins 24 hr after challenge with *E. coli* were pelleted and extracted using TRI Reagent^®^ (Molecular Research Center Inc.) for RNA and DNA isolation according to the manufacturer's instructions. The extracted RNA and DNA were each dissolved in 50 µl of nuclease free water. The quality of RNA was determined by formaldehyde agarose gel electrophoresis [26], while DNA quality was assessed by standard agarose gel electrophoresis. RNA quality was considered good when formaldehyde agarose gels showed 18 S and 28 S ribosomal RNA bands, without smears. DNA preparations that contained high molecular weight (MW) DNA, without smearing, were considered to be of good quality.

### RT-PCR

RT-PCR was carried out with 0.05–3 µg of total RNA, using either Superscript III (Invitrogen) or PowerScript reverse transcriptase (Clontech) in conjunction with SpAncRT or SMARTIV and CDIII (Clontech) oligonucleotides (Table 1). All RT-PCR procedures were carried out in accordance with manufacturers' protocols. Both the 5′ and 3′ oligonucleotides used in reverse transcription reactions provided binding sites for primers used in PCR amplifications.

**Table 1:** Oligonucleotides used in RT-PCR, PCR and sequencing.

Letter	Name	Function	Sequence (5′– 3′)
A	SMARTIV Oligo	5′ linker used in RT-PCR	AAGCAGTGGTATCAACGCAGAGTGGCCATTACGGCCGGG
B	CDSIII 3′ PCR primer	3′ linker used in RT-PCR and as generic 3′ PCR primer	ATTCTAGAGGCCGAGGCGGCCGACATGd(T)_30_N_-1_N
C	SpAncRT	3′ linker used in RT-PCR	ACTATCTAGAGCGGCCGC(T)_16_V
D	SMARTIVPCRF	5′ PCR primer, binds to SMARTIV Oligo	AAGCAGTGGTATCAACGCAGAGT
E	SpR1	3′ PCR primer, binds to SpAncRT	ACTATCTAGAGCGGCCGCTT
F	185F5	*Sp185/333* forward primer	GGAACYGARGAMGGATCTC
G	185R6	*Sp185/333* reverse primer	GCAGCATCAGTTTCTTCKTCTC
H	MRHE1855′UTRF1	*He185/333* 5′ UTR primer	GCTAGTTCTCTCTTGGAAGCTGGACGA
I	MRHE1855′UTRF4	*He185/333* 5′ UTR primer	TCTCTTGGAAGCTGGACGAA
J	MRHE1855′UTRF2	*He185/333* 5′ UTR primer	TGGAAGCTGGACGAAGGGAAAGGA
K	MRHE185R6	*He185/333* 3′ UTR primer	TGGAAAAGACATCAGTGACA
L	MRHE185R9	*He185/333* 3′ UTR primer	CTTTCAGGAATGTAATTGTCTTGAT
M	MRHE185R10	*He185/333* 3′ UTR primer	CTGCAATTTTTCTACAAACTCA
	MRPGEMTM13F	Forward sequencing primer	CGCCAGGGTTTTCCCAGTCACGAC
	MRPGEMTM13R	Reverse sequencing primer	TCACACAGGAAACAGCTATGAC

The letters in the first column refer to the primer binding sites indicated in Fig. S1.

### *He185/333* PCR amplification optimization

PCR amplification of 185/333 sequences was carried out in 50 μl reactions using the Advantage 2 PCR system (Clontech) or Phusion DNA polymerase (Finnzymes). Each reaction consisted of 0.2 μM dNTPs, 0.4 µM of each primer (Table 1) and 1 µl of RT-PCR reaction or gDNA as template. The cycling parameters were as follows; for Advantage 2, an initial denaturation at 94°C for 2 min, followed by 35 cycles of denaturation at 94°C for 30 s, annealing at 60°C for 30 s and extension at 72°C for 3 min followed by a final extension at 72°C for 5 min. For amplification using Phusion DNA polymerase, the following conditions were used: an initial denaturation at 98°C for 1.5 min, followed by 35 cycles of denaturation at 98°C for 15 s, annealing at 60°C for 20 s and extension at 72°C for 20–40 s, followed by a final extension at 72°C for 3 min. Amplification products were analyzed by agarose gel electrophoresis.

### Amplification strategy

**Figure 1:**
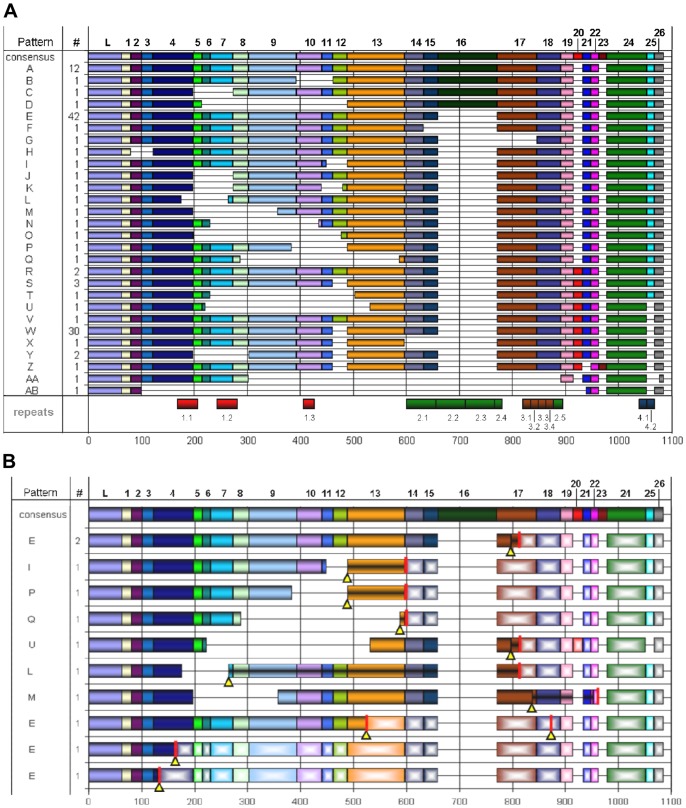
Element patterns of *He185/333* cDNAs. **A.** A total of 26 distinct elements (1–26 and leader (L) – see consensus pattern in the top row of the alignment) have been identified amongst He*185/333* sequences, and these are arranged into 28 unique element patterns. A horizontal line indicates elements that are “missing”. The locations of sequence repeats are shown at the bottom of the figure. The numbers at the bottom of the alignments indicate nucleotide positions. **B.** Element patterns of *He185/333* cDNAs with mutations. Sequences with the element patterns E, I, P, Q, U, L, M contained insertions, deletions or point mutations, resulting in frame shifts and/or early stop codons. Mutations are marked by black lined yellow arrowheads, early stop codons as red bars across an element. Arrowheads falling together with bars are cases of stop codons caused by point mutations, as seen in the three lower element patterns. Arrowheads and bars that are located apart from each other are cases of insertions or deletions, resulting in frame shifts and downstream early stop codons. Elements between mutations and early stop codons contain black central lines to point out missense translations, due to the frame shift. Elements after early stops are patterned with white centres to highlight non-translated regions of the cDNAs.

Attempts to amplify *He185/333* sequences from *H. erythrogramma* using *Sp185/333* primers [16] were not successful. This was not unexpected, as the diversity of *185/333* sequences creates formidable barriers to amplification, especially when applied across species. Thus, a modified rapid amplification of cDNA ends (RACE) strategy was conducted (Fig. 1): Linker sequences at the 5′ (SMARTIV oligo) and 3′ (CDSIII) ends of the double stranded *He185/333* cDNAs provided annealing sites for PCR primers. Primers (5′ SMARTIV primer and 3′ CDSIII or 3′ SpR1) specific to the linker regions were used in combination with *Sp185/333* primers to generate partial *He185/333* sequences (5′ and 3′ ends) that in turn enabled primers specific to *He185/333* UTRs to be designed (MRHE1855UTRF1, MRHE1855UTRF2, MRHE5UTRF4, MRHE185R6, MRHE185R9, MRHE185R10; see Table 1). By using these primers in PCR reactions, full length (i.e. complete ORFs) *He185/333* cDNA and gDNA sequences were amplified from *H. erythrogramma*. Those PCR primers were designed to bind to highly conserved nucleotide stretches in the UTRs and the leader/element 1 regions. Although this approach allowed us to identify more than 100 unique *He185/333* sequences (see Results), *He185/333* sequences that contain variant UTRs, if present, will not be identified. Despite this, the population of unique *He185/333* sequences that we identified was sufficiently large for this study.

### Cloning and sequencing of PCR products

Amplicons from PCR reaction mixes or amplicons separated by agarose gel electrophoresis were purified with the PCR purification or QIAquick gel extraction kits according to the manufacturer's instructions (Qiagen) and eluted in 30 µl or 50 µl of 10 mM Tris-HCl, pH 8.0. The DNA was quantified by spectrophotometry. Purified DNA were ligated to pGEM T-Easy Vectors (Promega) overnight at 4°C and subsequently used to transform chemically competent One Shot^®^ Top Ten (Invitrogen) or JM109 (Promega) *E. coli* cells according to standard protocols. Transformed bacterial culture (200 µl) were plated on pre-warmed LB-Agar plates with 100 μg/ml ampicillin, 50 μg/ml X-Gal and 0.5 mM IPTG, and incubated overnight at 37°C.

Plasmids were isolated from overnight cultures of *E. coli* using the QIAprep miniprep kit (Qiagen) according to the manufacturer's instructions. Purified plasmids were eluted in 50 µl of 10 mM Tris-HCl, pH 8.0 and evaluated by spectrophotometry and agarose gel electrophoresis. DNA inserts were sequenced at the Australian Genome Research Facility (AGRF, University of Brisbane, Queensland). Inserts were sequenced in both the forward and reverse directions with MRPGEMTM13F and MRPGEMTM13R sequencing primers (Table 1).

### Analysis of DNA sequences

After removing vector and primer sequences computationally, the forward and reverse sequences for each cloned insert were aligned using the BioEdit sequence alignment editor [27]. Homology searches were performed using nucleotide BLAST [28]. Sequences homologous to *Sp185/333* were aligned with ClustalW2 (default parameters, nucleotide sequences: gap initiation penalty  = 3, gap extension penalty  = 1, base match score  = 2, base mismatch penalty  = 1; amino acid sequences: gap initiation penalty  = 8, gap extension penalty  = 2; see Refs. [29], [30]) and the alignments were further edited manually in BioEdit.

### Immunofluorescence labeling and confocal microscopy

Coelomocytes were collected from sea urchins 48 hrs after challenge with E. coli. The density of coelomocytes in the final suspension was determined using a Neubauer Cell haemocytometer and adjusted to 1×106 cells/ml with CMFSW-EI (on ice). The cell suspension (100 µl) was pipetted onto glass slides and cells were allowed to settle and adhere to the slide surface for 5 min. Cells were fixed at 17°C in 0.5%–1% paraformaldehyde (PFA) in CMFSW-EI in a two-step procedure: (i) in 0.5% PFA for 15 min, (ii) 1% PFA for 15 min. After three washes in CMFSW-I, cells were permeabilized with 0.025% Triton X100 in CMFSW-I for 3 min followed by three washes for 5 min each. Non-specific epitopes were blocked with heat inactivated horse serum for 30 min at 17°C before the primary rabbit antisera mix was added for 1 h at 17°C. The primary antisera mix contained three polyclonal antibodies, anti-Sp185–66, anti-Sp185–68 and anti-Sp185–71 (each diluted 1:10,000 in CMFSW-I, kindly provided by L. Courtney Smith), which targeted the N-terminal-, central- and C-terminal- 185/333 regions [22]. The antibodies were raised in rabbits against synthetic peptides corresponing to those regions. The anti-Sp185/333 antibodies cross react with the He185/333 proteins as described [24]. After 3 washes, slides were incubated for 1 h in the dark at 17°C in a cocktail consisting of secondary antibody (mouse anti rabbit IgG-Alexafluor 546 conjugate), actin counterstain (phalloidin-Alexafluor 488 conjugate) and/or nuclear counterstain (Toto; all dyes from Invitrogen). Slides were washed three times in CMFSW-I. Finally, Biomeda Gel Mount (ProSciTech) was added, a coverslip overlaid and sealed with nail polish, and the slides stored at 4°C until analyzed on the Fluoview 300 laser scanning confocal microscope (Olympus).

### Protein extraction

Total protein from coelomocytes was extracted and purified for subsequent SDS-polyacrylamide gel electrophoresis (SDS-PAGE). Total coelomic fluid (12.5–25 ml depending on the size of the sea urchin) was collected after removal of Aristotle's Lantern and coelomocytes were pelleted at 3000×*g* for 5 min at 4°C. The cell pellet was lysed in lysis buffer (5 ml; 7 M urea, 2 M thiourea, 1% 3-[4-heptyl]phenyl-3-hydroxypropyldimethylammoniopropanesulfo-nate [ASB-C7BzO], 40 mM Tris base, 15 mM acrylamide [Bio-Rad], 10 mM tributyl phosphine [TBP], and 1x complete protease inhibitors [Roche]) using a French press (Thermo Spectronic). The lysate was incubated for 1 h at room temperature to allow complete reduction and alkylation of cysteines. Cell debris was pelleted by centrifugation for 10 min at 5000×*g*, the supernatant was transferred to a fresh tube and proteins were precipitated for 30 min at room temperature with five volumes of acetone and centrifuged for 10 min at 5000×*g*. The acetone supernatant was decanted, the pellet was air dried for 5 min and dissolved in 0.5–1.5 ml of sample buffer (7 M urea, 2 M thiourea, 1% ASB-C7BzO). Finally, the protein solutions were desalted using Micro Biospin tubes (Bio-Rad) and the eluates stored at −20°C.

### Protein assay

Proteins were quantified using the Non Interfering™ Protein Assay kit (G-Biosciences) according to the manufacturer's instructions. BSA was used as the protein standard reference.

### SDS polyacrylamide gel electrophoresis (SDS-PAGE)

SDS-polyacrylamide gels (10%) were loaded with 20 μg protein/lane and separated for an initial 10 min at 100 V followed by 80 min at 150 V and stained with Coomassie blue silver stain (20% methanol, 10% (w/v) ammonium sulfate, 1.6% orthophosphoric acid, 0.15% (w/v) Coomassie G250). Other gels were loaded with 10 μg proteins per lane followed by Western blotting (see below). Either Broad Range or Precision Plus protein markers (Bio-Rad) were used as size standards.

### Western blotting and immunodetection

Proteins were blotted onto polyvinylidene fluoride membranes for 1 h at a constant current of 2 mA/cm^2^ in a Transblot semidry transfer cell (Bio-Rad) followed by two washes in Tris buffered saline (TBS buffer, 20 mM Tris-HCl, pH 7.5, 150 mM sodium chloride) for 10 min each at room temperature, blocking of the membrane at 4°C overnight in TBS buffer containing 10% skim milk powder (block buffer), three washes at room temperature in TBS-Tween/Triton buffer (TBS buffer, 0.05% (v/v) Tween 20, 0.2% (v/v) Triton X-100) and incubation with the primary antibody (equal mix of anti-185/333 antisera diluted 1∶10000 in block buffer). This was followed by three washes in TBS-Tween/Triton buffer for 10 min each at room temperature and incubation in the secondary antibody (goat anti rabbit IgG Alkaline Phosphatase conjugate, 1∶20000 in block buffer), followed by three final washes in TBS-Tween/Triton buffer. Protein-antibody complexes were visualized by colour reaction with 3 ml BCIP/NBT per 150 cm^2^ membrane area for 2–5 min at room temperature and photographed with a digital camera (Canon EOS 40 d).

### Phylogenetic analyses

Twenty-five randomly chosen *185/333* cDNA sequences from each species, *H. erythrogramma* and *S. purpuratus*, were aligned in ClustalW with default parameters and the alignment was further refined manually in BioEdit. Phylogenetic analyses were performed in PAUP*4.0b10 [31] using character based, distance-based and model-based (Maximum Parsimony, MP; Neighbor-joining, NJ and Maximum Likelihood, ML) methods of analysis. For MP analysis, a heuristic search strategy was employed to identify the most parsimonious tree. All characters were treated as unordered and un-weighted, while gaps were treated as missing data. Bootstrap re-sampling based on 1000 replicates was used to assess the support of relationships for the majority-rule consensus tree. For the NJ and ML phylogenetic analyses MODELTEST v.3.06 [32] was used to estimate the most likely model of sequence evolution for the sequence data. Based on the Akaike Information Criterion (AIC), Tamura-Nei (+G) was selected as the most likely model of sequence evolution for *185/333*. Corrected genetic distances based on 2025 positions in the alignment were calculated in PAUP*4.0b10. NJ and ML trees were obtained in PAUP*4.0b10 using model parameters specified by MODELTEST and NJ was also assessed with 1000 bootstrap replicates.

### Diversity analysis

The diversity of *He185/333* sequences was determined from their alignments using the HyPhy suite of algorithms that were accessed via Datamonkey [33], [34]. Unique, full-length *He185/333* cDNA sequences were processed to remove 5′ and 3′ untranslated regions (UTRs). As there were 112 *He185/333* sequences in our dataset, we customized our analytical approach to circumvent data processing restrictions on Datamonkey, which will only process a maximum of 100 sequences at a time. We developed a custom script to randomly select 100 *He185/333* sequences from our dataset for the analysis. These sequences were aligned using ClustalW [29] as described above and uploaded to Datamonkey for diversity analysis. Each alignment was subjected to automatic nucleotide substitution model detection, generation of NJ trees and then SLAC (Single Likelihood Ancestor Counting, see Ref. [35]), FEL (Fixed Effects Likelihood, see Ref. [35]), IFEL (Internal Fixed Effects Likelihood, see Ref. [36]) analyses. The diversity scores were considered to be significant at a confidence interval of p≤0.1. The final diversity score for *He185/333* sequences was the consensus of the data output from all three analytical algorithms (SLAC, FEL and IFEL). This was repeated a further nine times (i.e. a total of ten sets of sequences, each containing 100 sequences, were analyzed) and a consensus diversity score was generated. A similar analysis was conducted on 231 *Sp185/333* cDNA sequences so that the data from the two families of *185/333* sequences could be compared.

## Results

### *He185/333* cDNA sequences

A total of 112 unique full-length cDNAs were obtained. BLAST searches revealed significant homology of all sequences only to *Sp185/333* mRNAs and genes, with sequence identity of 68% to 74%. All cDNAs contained an open reading frame (ORF) of 219 to 1050 nucleotides, a Kozak consensus sequence (5′-CAGACATGG-3′; see Ref. [37]) and an in-frame stop codon.

Optimal alignment of the 112 *He185/333* revealed conserved sequence blocks or sequence elements (Fig. 1A and S2), a feature associated with *185/333* sequences. Each element has a conserved sequence and amongst the 112 He*185/333* sequences that were characterised in this study, a total of 31 different elements were identified. The shortest elements were 15 bp in length (elements 7, 11, 14, 16, 26, 27, 28 and 30), while the largest one was 111 bp long (element 21). Each *185/333* sequence is composed of a mosaic of elements, which is referred to as an element pattern. 29 distinctive element patterns were evident amongst the He*185/333* sequences (alphabetically labelled A–AC, Fig. 1A). In our library, all except for six element patterns were singletons; A (12 clones), E (41), R (2), S (3), W (30) and Y (2). Depending on the primers that were used, regions of up to 33 bp of the 5′ UTRs and up to 105 bp of the 3′UTRs were amplified (note: the location of the 5′UTR was based on the alignment of the *He185/333* sequences with *Sp185/333* sequences, especially with the region surrounding the initiating codon). One partial *He185/333* cDNA contained the 3′end of the ORF and an entire 3′UTR including the polyadenylation signal sequence (PAS, 5′-ATTAAA-3′) was located 185 bp downstream of the stop codon and 21 bp upstream of the poly A-tail (data not shown). Of the 112 cDNA sequences, 11 had early stop codons and/or frame shifts resulting from indels or point mutations (Fig. 1B) and encode truncated polypeptides, some with missense sequence.

### *He185/333* gDNA sequences

**Figure 2:**
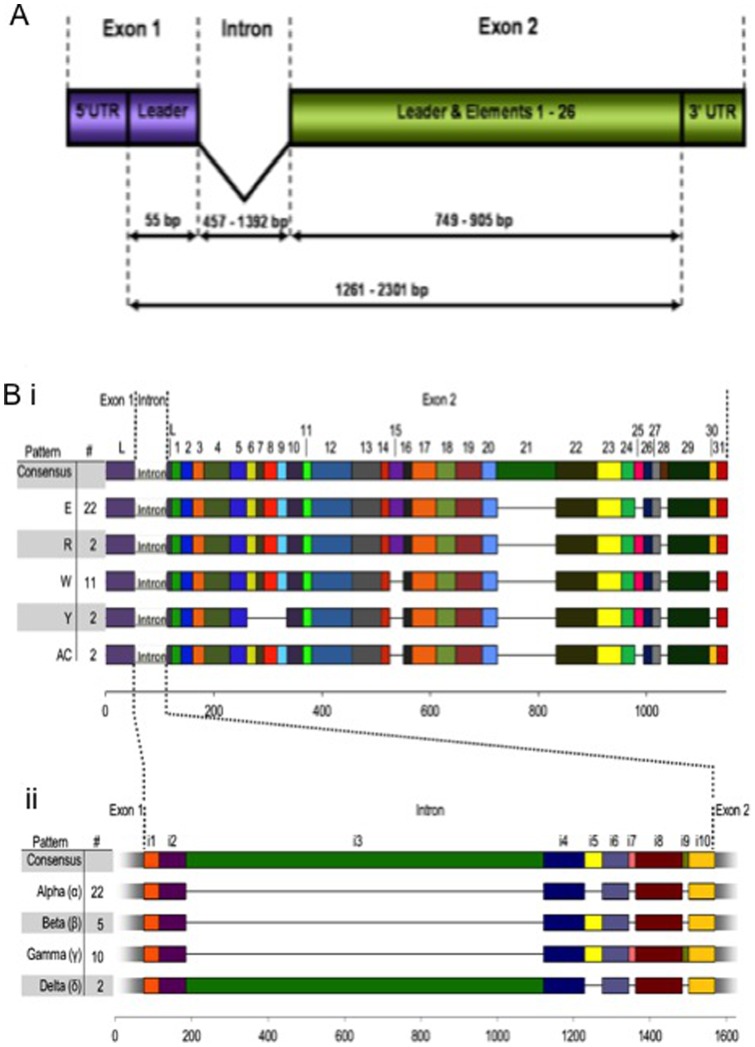
*He185/333* gene structure. **A.** Genes consist of a short exon 1 and a longer exon 2, separated by one intron. The leader is 63 bp in length and is interspersed between positions 55/56 by the Intron. Thus, the leader represents exon 1 (excluding the 5′UTR) but also forms the first eight nucleotides of the second exon. The intron varies in size between 457 bp and 1392 bp. The second, long exon ranges from 749 bp to 905 bp (excluding the 3′UTR) and contains the mosaic organisation of elements. The 5′ and 3′ UTRs of *He185/333* genes have only been partially sequenced. **B. Element patterns of 39 manually aligned** ***He185/333*** **gDNA sequences. i.** The coding regions (exons 1 and 2) are interspersed by one intron, dividing the leader in two parts, as indicated at the top of the diagram. Genes identified to-date show the five exon element patterns E, I, R, W and Y as described for cDNAs in figure 1A. Introns are simplified by interrupted, checkered boxes. The consensus at the top of the diagram is based on all element patterns, including cDNA element patterns as shown in figure 1A. **ii.** Similar to exons, introns align optimally with insertion of large gaps, resulting in ten intron elements and four intron element patterns, designated alpha (α), beta (β), gamma (γ) and delta (δ). Individual intron elements are named i1 to i10 and are shown as differently shaded gray boxes. Exons are represented as fading extensions to the left and right of intron element patterns. **i & ii.** Combinations of exon and intron element patterns define gene element patterns of which the following nine have been identified among the 39 gene sequences (number of individual sequences with according gene element pattern in brackets: E-α (11), I-α (2), W-α (8), Y-a (1), E-β (2), R-β (2), W-β (1), E-γ (10), W-δ (2). The first element pattern is the consensus.

To date, 39 unique genes were sequenced, and these varied in length between 1261 bp and 2301 bp (Fig. 2 and S3). They consisted of two exons, the first and shorter of which was 55 bp long (excluding the putative 5′ UTR, of which 33 bp were amplified). The size of the larger second exon ranged from 749 bp to 905 bp (excluding the 3′ UTR, of which either 30 bp or 105 bp were amplified, depending on the primers used in PCRs). The intron ranged in size from 457 bp to 1392 bp. The element patterns in the second exon matched the cDNA element patterns A, E, I, W and Y (Fig. 2B). An additional element pattern, AC, which was not identified in the library of cDNA sequences was found among the gDNA sequences. Element patterns were also evident in the introns (Fig. 2B). A total of 10 intron elements (i1– i10) were identified and these were in four recognizable intron element patterns (alpha (α), beta (β), gamma (γ) and delta (δ)). The intron patterns, when combined with exon patterns, form nine gene element patterns; E-α, AC-α, W-α, Y-α, E-β, R-β, W-β, E-γ and W-δ.

### Deduced polypeptide sequences

**Figure 3:**
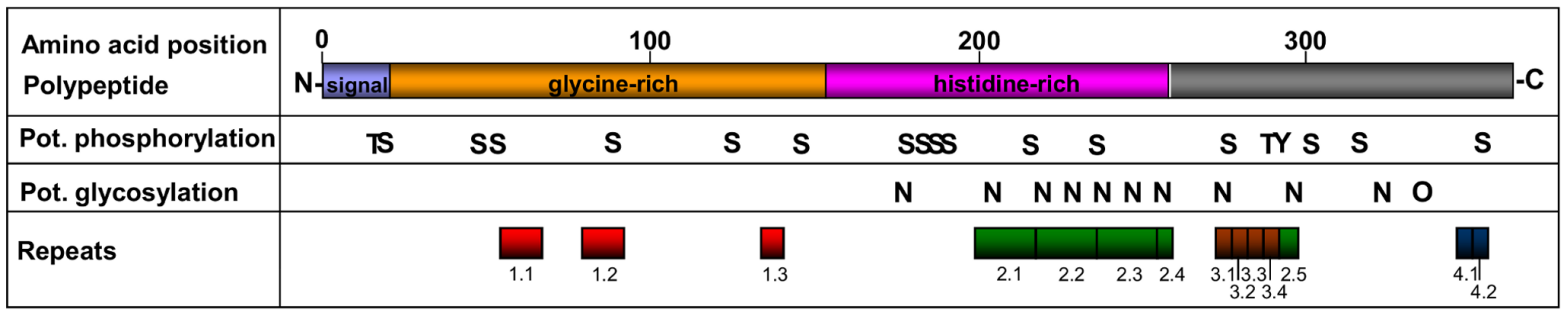
Structural overview of deduced consensus of He185/333 polypeptides. Approximate amino acid positions are given at the top, amino/carboxy-terimini are labeled with N and C to the left and right of the consensus, respectively. Polypeptides carry a signal sequence at the N-terminus, followed by glycine-rich and histidine-rich regions. Potential phosphorylated serine, threonine and tyrosine residues are marked with S, T and Y respectively and potential N- and O-linked glycosylations with N and O, respectively. Repeats, as shown for cDNA element patterns in figure 2A and in table S2, are shown at the bottom and are labeled with repeat type followed by copy number.

The deduced polypeptides had between 72 and 349 aa with predicted MW ranging from 8 to 39 kDa and the predicted isoelectric points (pIs) ranged from 4.63 to 6.99. The first 21 aa represented a predicted signal sequence (see Ref. [38] and Fig. 3), suggesting an extracellular destination of the He185/333 proteins. At the same time the He185/333 proteins were predicted to lack both transmembrane regions [39] and canonical signatures of glycosylphosphatidylinositol-anchors [40]. The deduced polypeptides also contained up to ten potential N- and one O-linked glycosylation sites [41] and a total of 16 potential serine-, two threonine- and one tyrosine-phosphorylation sites were predicted [42]. The translated sequences included an N-terminal glycine-rich and a central histidine-rich region. The predicted polypeptides were also rich in arginine, which were evenly distributed along the polypeptide and typically constituted 11%–12% of total number of amino acids. The histidine rich region contained a poly-histidine stretch of at least six, but usually more (8 to 13) consecutive residues. Common web-based programs did not predict extensive secondary structures or folding patterns.

### Repeats

We found four types of tandem and interspersed sequence repeats in 104 of the 112 185/333 deduced polypeptide sequence, named types 1 to 4 (Fig. S4 and S5) and were comparable to five repeat types in *S. purpuratus* (Fig. S6). Eight sequences that contained non-synonymous substitutions and frameshift mutations (sequences He_185/333_cDNA_105–112) were omitted in the analysis of sequence repeats because major parts of their deduced amino acid sequence were not homologous to 185/333 proteins. Most of the He185/333 repeats were homologous to repeats found in *S. purpuratus* but varied in the maximum repeat copy numbers and in their length.

### Phylogenetic analysis

**Figure 4:**
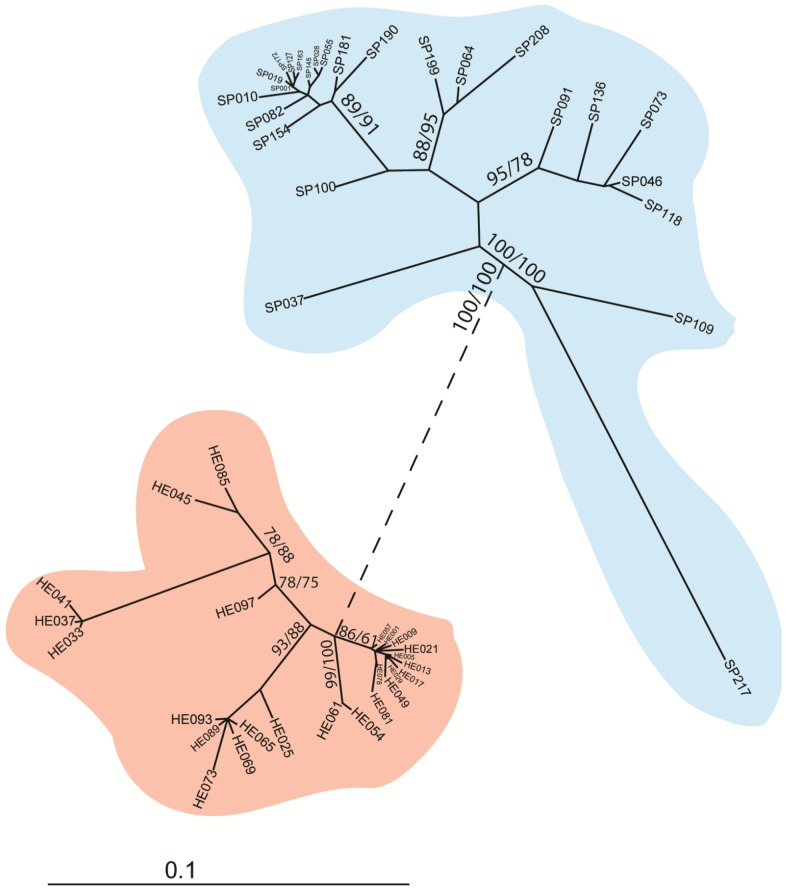
Phylogenetic relationship between 25 *He185/333* and *Sp185/333* cDNAs. *He185/333* clades are highlighted in a darker shade than *Sp185/333* clades. The phylogenetic tree shows clearly that 185/333 sequences cluster according the species they originated from. The main branch separating the two clades was shortened for display purposes (dashed line). It is representative of the corrected genetic distance of 0.27949 and is supported by 100% of bootstraps. Hence, all sequences within one species had lower genetic distances to each other (highest value 0.07705 for *He185/333* and 0.12781 for *Sp185/333*, respectively) than to any sequence from the opposite species. Boot Strap values are shown next to the branches with the ordering NJ/MP. Values below 50 are not displayed.

As *185/333* sequences are members of a diversifying gene family [15]–[19], [21], the identification of these sequences in a second species (*H. erythrogramma*) enabled us to compare the sequence similarity amongst *185/333* sequences from these two animal groups. Analysis of the phylogenetic relationships between unique *He185/333* and *Sp185/333* cDNAs indicates that the sequences clustered according to the species from which they derived (Fig. 4). Of the 2025 bp of the aligned sequences, 418 characters were variable and 287 were parsimony informative. The Tamura-Nei corrected genetic distances ranged from 0.00216 to 0.07705 for *He185/333* and from 0.00115 to 0.12781 for *Sp185/333* (Table S2). The genetic distances between *He185/333* and *Sp185/333* were significantly greater and ranged from 0.27949 to 0.38071. Moreover, groups of *185/333* within each species clustered separately from one another in branches with well-supported bootstrap values, indicating presence of subfamilies that may have originated by duplication and divergence from a common founding member.

Within the *H. erythrogramma* clade, there was a correlation between the sequence clusters and the element patterns of the *He185/333* sequences within those clusters. Three distinct clusters were evident: one cluster consisted exclusively of sequences with exon element pattern W (sequences HE085, HE045, HE041, HE037 and HE033), while the second contained sequences with element patterns E, F, M and O (sequences HE061, HE054, HE081, HE078, HE049, HE029, HE017, HE013, HE005, HE021, HE009, HE001 and HE057). The third cluster, which was most diverse in terms of the element patterns, contained the patterns A, C, G and S (sequences HE097, HE093, HE089, HE073, HE069, HE065 and HE025).

### Diversity analysis

**Figure 5:**
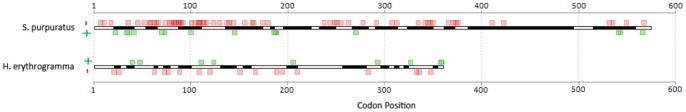
A comparison of codon diversification between *S. purpuratus* and *H. erythrogramma 185/333* cDNA alignments. Sequence alignments of 231 *S. purpuratus* and 112 *H. erythrogramma* cDNAs were used to investigate recombination and selective pressure at the codon level. Element patterns are shown as black and white banding, while positively- and negatively-selected codons are shown as green and red boxes above and below each alignment, respectively. Negative selection (against codon diversification) appears to be prevalent across *185/333* sequences from both species. Significant (p<0.1) negative selection was detected in 64 codons across the 581 codon-long *S. purpuratus* alignment, while 15 codons were positively-selected. The 360 codon-long *H. erythrogramma* alignment contained 17 codons under significant negative selective pressure and nine codons with positive selective pressure. *S. purpuratus* element patterns adapted from [16].

A total of 112 unique *He185/333* cDNAs were analyzed using the HyPhy suite via Datamonkey to detect diversity and selection pressure at the level of individual codons (Fig. 5, and table S3). The consensus output from SLAC, FEL and IFEL identified 17 codons (4.6%) that were under negative selection, while nine codons (2.5%) were under positive selection (p<0.1). In comparison, the *Sp185/333* sequences had 64 codons (11%) under negative selection and 15 codons (2.6%) under positive selection. While positively and negatively selected sites were evenly distributed along the length of the *He185/333* alignments, 39 of the 64 negatively selected sites were located within the first 200 codons of the 581 codon *Sp185/333* alignment, while only six sites were located in the last 200 codons. Positively selected sites also appeared to cluster within the first 200 codons, with eleven of the 15 positively selected sites present.

### He185/333 protein expression profile

**Figure 6:**
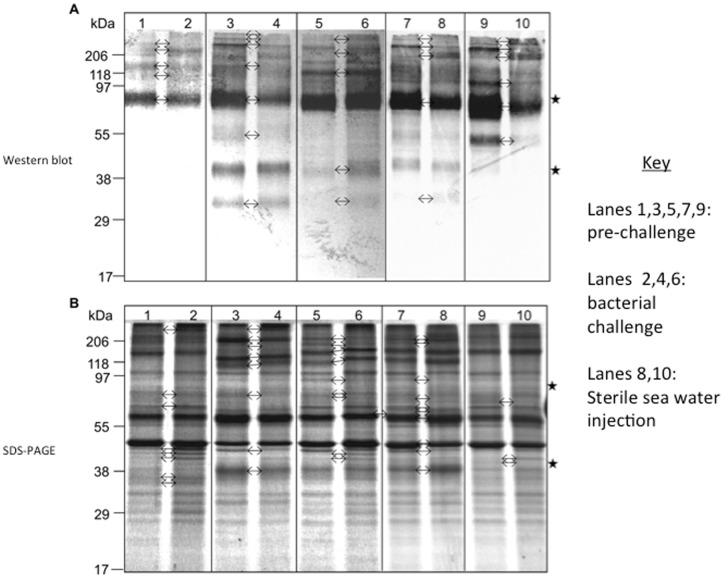
Total coelomocyte proteins from five sea urchins were analysed by Western blotting (A) and SDS-PAGE (B). Coelomocyte proteins were extracted from animals either before (pre-) or after (post-) immunological challenge with heat killed bacteria. Other treatments included injections with filtered sea water (FSW) and injury (pricked with a sterile needle). In both panels A and B, lanes with odd numbers (1, 3, 5, 7 and 9) show pre-challenged protein profiles while lanes with even numbers (2, 4, and 6) show post-challenge protein profiles. Lanes 8 and 10 show the profiles after filtered sea water injection and Injury, respectively. Asterisks to the right of the figures indicate regions that are not stained with Coomassie Blue (B), which contain *He185/333* ^+^ bands in the Western blot. **A.** The Western blot shows a diverse pattern of *He185/333* proteins between animals but also changes within individuals before and after immunological challenge, FSW injection and injury. Bands on the blot are not as discrete and sharp as their corresponding bands on the Coomassie Blue stained gel, but appear to be rather diffuse and large. Arrows between pre- and post-challenged samples indicate bands that change in intensity or are present/absent as a result of the experimental treatment. **B.** The Coomassie Blue stained gel shows discreet, sharp protein bands, some of which differ in size and intensity between animals and within individuals before and after immune challenge. None of the bands, however, could unambiguously be identified as *He185/333* ^+^ band when compared to the Western blot.

Western blots identified a broad range of MW for He185/333 proteins, ranging from expected values for monomers deduced from cDNA sequences to MW greater than 206 kDa (Fig. 6A). The majority of He185/333 positive bands were greater than 75 kDa, and their MW were not altered by the strong reducing agent TBP. There were considerable variations in the repertoires (relative sizes and intensities on Western blots) of He185/333 proteins between different individual sea urchins (Fig. 6A). Such variations in He185/333 protein repertoires were also evident within individuals after they were injected with heat-killed bacteria, filtered sea water or sterile needle injury (Fig. 6A).

Interestingly, He185/333-positive bands on 1DE Western blots could not be associated with specific bands on Coomassie-stained gels. In fact, strong He185/333 signals on Western blots were associated with non-stained areas on Coomassie gels (Fig. 6A and 6B). Dheilly et al. also noted similar observations of Sp185/333 proteins [24]. This is thought to be due to the chemistry of protein-dye interactions: the post-translational modifications of the 185/333 proteins are believed to interfere with Coomassie blue interacting with those proteins, resulting in a poor alignment of Coomassie stained 185/333 proteins and the corresponding bands on Western blots (see Ref. [24] and asterisks in Fig. 6A and 6B).

2DE Western blots showed a diverse range of MW and isoelectric points (data not shown), and were very similar to those of Sp185/333 proteins [24]. Disparities between observed MW and pI and those predicted from He*185/333* sequences were observed. This suggests that He*185/333* proteins are post-translationally modified. Furthermore, the protein spots were arranged in trains, an effect often associated with differential glycosylation [24].

### Localization of He185/333 proteins in coelomocytes

**Figure 7:**
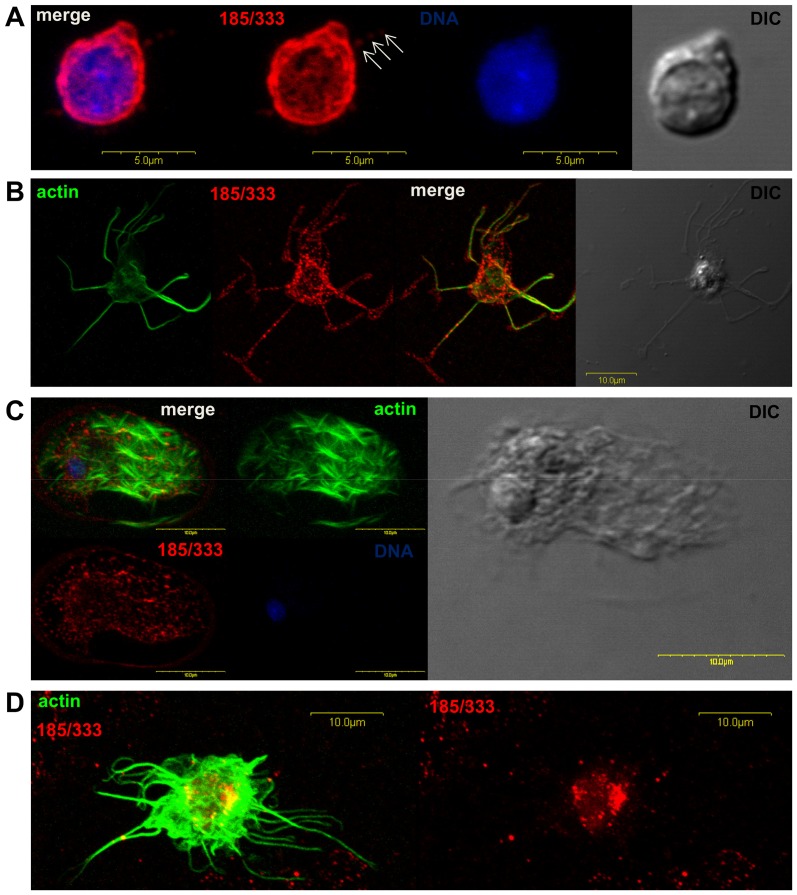
Cellular expression of He185/333 proteins. **A–D.** Immunofluorescence and differential intereference contrast (DIC) microscopic images of different coelomocyte types expressing He185/333 proteins (red) and actin (green). Nuclear DNA appears in blue. **A.** A small filopodial amoebocyte expressing *He185/333* proteins. *He185/333* staining is found on the cell surface and the clustering of *He185/333*-associated fluorescence in knobs is evident in a filopodium (white arrows). **B.** A large filopodial amoebocyte expressing *He185/333* proteins in dense knobs. *He185/333* signals are not uniformly distributed but are found in patches. **C.** A large filopodial amoebocyte expressing *He185/333* within the cell body in perinuclear areas. **D.** A lamellipodial amoebocyte expressing *He185/333* as knobs on the cell surface.

Immunofluorescence confocal microscopy showed that He185/333 expression was present in two distinct coelomocyte subpopulations: filopodial and lamellipodial amoebocytes (Fig. 7). These coelomocytes are morphologically and functionally (phagocytosis) equivalent to the corresponding cell types in *S. purpuratus* [25], [43]. Z-stack analysis of the images indicated that the majority of the He185/333 proteins were located on the plasma membranes (data available on request). In some instances, He185/333 proteins were detected in perinuclear areas of large filopodial cells (Fig. 7C), which may reflect their distribution within the organelles involved in protein biosynthesis (e.g. endoplasmic reticulum, Golgi apparatus and transport vesicles). He185/333-specific immunofluorescence on amoebocyte surfaces appears to be patchy (Fig. 7A–D), suggesting that the distribution of He185/333 proteins may be tightly clustered. This was particularly evident for filopodial amoebocytes, where distinct knobs of He185/333-associated fluorescence were observed (Fig. 7A).

## Discussion

We describe the discovery and characterization of the *He185/333* gene family in the sea urchin, *Heliocidaris erythrogramma*, and present characterization of this gene family in a second group of sea urchins, after its original discovery in *S. purpuratus* [15], [23]. Sequences that are homologous to *Sp185/333* have been identified in other sea urchin genome sequencing projects, including *S. franciscanus and A. fragilis* (see Ref. [23] and http://www.ncbi.nlm.nih.gov/genome/?term=strongylocentrotus+purpuratus). All evidence to-date indicate that the *185/333* gene families are unique to the sea urchins, as no homologues have been identified in other animal groups.

### Similarities between *He185/333* and *Sp185/333*

Our analysis shows that *He185/333* sequences bear many similarities to those from *S. purpuratus*. These include (i) the organization of *185/333* cDNA and gene sequences into elements, (ii) sequence variations due to indels, SNPs and repeats, (iii) structure of *185/333* genes (two exons separated by an intron), (iv) structural features of the predicted 185/333 polypeptides (a hydrophobic leader, a glycine rich and a histidine rich region and several potential N- and O-linked glycosylation sites), (v) a large repertoire of 185/333 polypeptide sizes (from ∼39 kDa to >206 kDa), as well as differences in the repertoires of 185/333 polypeptides between individual animals, (vi) changes in the repertoire of 185/333 polypeptides upon immunological insult or injury, and (vii) cell surface and peri-nuclear expression of 185/333 proteins in amoebocytes. These similarities suggest that He185/333 and Sp185/333 are closely related molecular families that bear substantial structural and functional similarities.

### Differences between *He185/333* and *Sp185/333*

Although both *Sp185/333* [15], [16], [18] and *He185/333* sequences are composed of elements (conserved sequence blocks), the nucleotide sequences of elements, as well as the number of elements in each gene family, are very different. Hence, an independent element pattern system had to be developed for *He185/333*, which resulted in 29 cDNA element patterns with 31 elements as opposed to 38 patterns with 25 elements in *Sp185/333* [16], [17]. Also, although all four repeat types in *H. erythrogramma* find their homologous counterparts in *S. purpuratus*, the overall repeat lengths and maximum copy numbers vary between the two species [19].

Phylogenetic analysis shows that despite all similarities, the sequences cluster into two groups that are defined by the species from which they originate. Hence, although there are clear homologies between *Sp185/333* and *He185/333* the distinct clustering of the two groups suggests independent evolution of the two gene families after the divergence of the host animals. Although extensive divergence is evident within *He185/333* sequences and Sp*185/333* sequences, there is also a clear distinction between these homologous sequences in the two groups of animals. Hence, although there are clear homologies between *Sp185/333* and *He185/333* the distinct clustering of the two groups suggests independent evolution of the two gene families after the divergence of the host animals. It is estimated that the *Strongylid* sea urchins diverged from *Heliocidaris* approximately 35 MYA [47]. These groups of sea urchins are geographically isolated: *H. erythrogramma* is predominantly found in the southern hemisphere, while Strongylocentrotus' habitats are located in the northern hemisphere [46]–[47]. Although it is not clear if the differences in their developmental life histories contribute to the differences that exist in the *185/333* homologues from these species (*S. purpuratus* is an indirect developer, while *H. erythrogramma* is a direct developer), it is reasonable to speculate that the pathogen pressures that are present in their respective habitats may have driven the evolution of *185/333* sequences in these two groups of sea urchins. Experimentally, this was also evident from the difficulties in our initial attempts to amplify He185/333 sequences in H. erythrogramma based on Sp185/333 sequences. Experimentally, this was also evident from the difficulties in our initial attempts to amplify *He185/333* sequences in *H. erythrogramma* based on *Sp185/333* sequences. The analysis of diversity within *He185/333* and *Sp185/333* cDNA sequences revealed a number of similarities as well as several differences between the two families. In both families, a greater proportion of codons were under negative selection than under positive selection. Negatively-selected codons represent those sites that are invariant and are likely to encode amino acids that mediate critical structural or functional roles [36]. In both families, negatively-selected codons occurred throughout the lengths of the sequences. In *He185/333*, positively-selected sites are also distributed throughout the sequence, whilst in *Sp185/333*, the majority of the positively-selected codons are located in the first half of the sequence. The significance of this is not clear. In concurrence with previous studies, our data support the notion that 185/333 polypeptides lack distinct ‘hypervariable’ regions. Our data, the combined result of three separate diversity analyses, indicate that pressure to diversify (positive selection) or conserve (negative selection) is not evident at the level of individual sequence elements (p<0.1). In their paper, Buckley et al. have suggested that RNA editing (deamination) may be a potential source of sequence diversification [21]. While we have not looked for evidence of RNA editing in our study, our demonstration of codon-level selection supports this notion.

Taken together, the *185/333* sequences from both *S. purpuratus* and *H. erythrogramma* appear to be under positive selection for diversification, although the selective pressures that drive this diversification are not known [15]–[21], [23]. Another, albeit unrelated, gene family in the sea urchins also undergoes positive selection and diversification. The bindin proteins on sea urchin sperm cells are involved in species-specific fertilisation. Molecular analyses indicate that the regions flanking the highly conserved core region of the *bindin* genes accumulate point mutations and indels. Phylogenetic analysis of the evolutionary rates of the bindin genes from several sea urchin species indicates that *Strongylocentrotus* and *Heliocidaris bindin* alleles have undergone rapid evolutionary divergence, compared to the other sea urchins (approximately four times greater in these species, compared to the others) [47]–[48]. It will be interesting to compare the evolutionary diversification rates between *185/333* genes with *bindin* genes, as well as those of the other immune response gene families.

Overall, our data indicated that *He185/333* cDNA sequences are less diverse than their *Sp185/333* counterparts; however, this may be because the set of unique *He185/333* cDNA sequences was smaller than the set of *Sp185/333* sequences. The *He185/333* cDNA sequences were obtained solely through RT-PCR experiments, implying that biases arising from the use of PCR primers may have reduced our ability to amplify diverse repertoires of *He185/333* cDNA sequences. *Sp185/333* sequences were initially obtained from screening of cDNA libraries, which enabled primer design for the subsequent production of RT-PCR amplicons that were cloned and sequenced [15]–[17].

While *Sp185/333* [18] and *He185/333* genes share structural similarities, there are major differences in their intron sequences. Firstly, the conservation of sequences between *Sp185/333* and *He185/333* introns is low (identity <50%) and BLAST searches using *He185/333* intron sequences, as queries do not match to *Sp185/333* gene sequences. Secondly, intron-types in *He185/333* genes are structurally different from those in *Sp185/333* and consist of elements, which enable their classification into intron-element patterns (designated as α, β, γ, and δ). In contrast, *S. purpuratus* introns (α, β, γ, δ, and ε) are defined by sequence dissimilarities and SNPs as based on phylogenetic analysis because the alignment of the *Sp185/333* introns is not improved by the insertion of gaps to identify elements [18]. Last, some *He185/333* genes carry unusually long introns of more than 1300 bp. This is approximately 2–3 times the average length of most *He185/333* and all *Sp185/333* introns and exceeds the combined lengths of *He185/333 coding regions of* exons 1 and 2 by several hundred base pairs. Introns as large as this have not, as yet, been identified in *S. purpuratus*.

In general, homologous stretches of sequence between *Sp185/333* and *He185/333* were not well conserved. On average, sequence identity of corresponding sequence stretches was about 70–80%, depending on the length of sequence that was compared. However, there were regions that were highly conserved: the leader sequences (first ∼55 bp of the open reading frame) were relatively well conserved between *Sp185/333* and *He185/333* (approximately 90% sequence identity at the amino acid level). Some well-conserved blocks of sequence, ranging from 10 to 15 amino acids showed identities of up to 100% between the two species. These sequence blocks often corresponded to regions of repeated sequences.

None of the predicted He185/333 polypeptide sequences contain RGD motifs. In Sp185/333 sequences, most of the predicted polypeptides have a single RGD motif [15], [16] but several Sp185/333 sequences that lack this motif have also been identified [17]. RGD motifs serve as binding sites for integrins [44], a family of plasma membrane anchor proteins which interact with cytoskeleton–connecting proteins and are involved in cell adhesion and signal transduction [45]. Assuming that both Sp185/333 and He185/333 subserve the same general immunological functions, it seems unlikely that RGD motifs in 185/333 proteins have fundamental functional significance. Similarly, the fact that cysteine residues are found in He185/333 proteins but are absent in all full-length Sp185/333 proteins (they have been predicted in missense sequences, see Ref. [24]), implies that disulphide-mediated tertiary or quaternary interactions in He185/333, if they do occur at all, may be functionally irrelevant. However, it is possible that such differences represent species-specific adaptations as a consequence of immunological or evolutionary pressures that are specific to each host. Our data indicate that disulphide bonding does not stabilize the high MW forms of He185/333. The presence of high MW *He185/333* proteins even in the presence of the strong reducing agent TBP suggests that they are likely to be non-disulphide stabilized, but covalently-linked oligomers. Similar discrepancies in the sizes of recombinantly-expressed Sp185/333 proteins were also identified [22], again suggesting that oligomerization occurs between Sp185/333 proteins. Since other sea urchin proteins were not present in the recombinant expression system, it is plausible that 185/333 proteins self-oligomerize to form higher-order structures.

In summary, *He185/333* and *Sp185/333* are homologous immune gene families that share many common features. They also demonstrate sufficient differences to suggest that the gene families have undergone independent evolution after the divergence of the host species. The data provided in the manuscript will contribute to our understanding of the evolution of this gene family that appears to be unique to the echinoderms.

## Supporting Information

Figure S1 **RACE-PCR amplification of** ***He185/333*** **sequences.** Oligonucleotides are represented as arrows and are labeled A-M. Messenger RNA was reverse transcribed (RT) into double stranded cDNA with addition of oligonucleotide linkers (A, B or C) to the 5′ and 3′ ends. Subsequent PCRs with the cDNA used linker primers (D, B or E) in combination with Sp185/333 specific primers (F, G) to produce partial *He185/333* sequences corresponding to 5′ and 3′ ends, including parts of UTRs to which *He185/333* specific primers were designed. PCRs using these *He185/333* UTR-primers (H-M) resulted in the amplification of full length *He185/333* sequences from cDNA and gDNA templates.
(TIF)

Figure S2 **Nucleotide sequence alignment for 112** ***He185/333*** **cDNAs generated in Clustal W and BioEdit.** The best alignment was obtained by insertion of large gaps, resulting in 26 sequence blocks (elements). The 26 elements are numbered along the top and separated by vertical black lines. The first and last three nucleotides represent the start and stop codon, respectively. The alignment shows full-length cDNAs irrespective of mutations that lead to missense and early termination upon translation. The cDNAs with such mutations are translated accordingly in Fig. S4.
(DOC)

Figure S3 **Nucleotide sequence alignment for 39** ***He185/333*** **gDNAs generated in Clustal W and BioEdit.** The untranslated regions (5′UTR and 3′UTR), leader, 26 exon elements and ten intron elements (with suffix “i”) are labeled along the top and separated by vertical black lines. The start and stop codons are shaded in green and red coloured boxes, respectively and labeled along the top. The exon elements have been numbered according to the categorization system based on the 112 cDNA sequences shown in Fig. S2. The leader, which is identical to the predicted signal sequence, counts 63 nucleotides but is separated by the intron between nucleotide positions 55 and 56.
(DOC)

Figure S4 **Alignment of 112 deduced He185/333 polypeptides generated in Clustal W and BioEdit.** The polypeptide sequences were deduced from the cDNA sequences shown in Fig. S2. The 26 elements are numbered along the top and separated by vertical black lines. Four types of repeats with tandem or interspersed-incomplete structures are highlighted in differently coloured boxes and labeled along the top. Also indicated along the top are glycine- and histidine-rich regions (orange and magenta arrows, respectively), predicted O-linked and N-linked glycosylation sites (red and blue triangles, respectively), as well as predicted serine, threonine and tyrosine phosphorylations (red, blue and green lightning bolts, respectively).
(DOC)

Figure S5 **Repeats found in 104 translated** ***He185/333*** **cDNA sequences, presented as sequence logos and linear sequences.** The figure shows repeat types and maximum copy number (left column), as well as sequence variations within repeats (central column) and the structure of each repeat type (right column). For example, there are up to three copies of the type 1 repeat (1.1, 1.2 and 1.3) in He185/333 deduced polypeptides. Sequence variations within repeats are depicted as sequence logos and as plain text. Sequence logos were generated using the software, Geneious (Geneious v5.4, http://www.geneious.com). The size of each letter within the sequence logos is proportional to the frequency of that residue at the specific position in the He185/333 alignment. For example, the first position of repeat 1.1 is G (glycine), with a value of 1, because it is invariant at that position amongst the 104 He185/333 sequences. In the plain text below the logos variant amino acids at specific positions are indicated in brackets.
(DOCX)

Figure S6 **Comparison of the four types of repeats from** ***He185/333*** **translated sequences with five** ***Sp185/333*** **repeat types (15).** The number of copies of each type of repeat varies between the two species and among sequences within one species. For example, type 1 repeat is present as two complete tandem repeats and one interspersed incomplete repeat in *H. erythrogramma* but as up to four tandem repeats in *S. purpuratus*. In *He185/333*, type 3 repeat appears as four tandem copies and is homologous to a portion of Sp185/333 type 5 repeat. Repeat type 5 is present up to three times in Sp185/333. Similarly, the last four residues of *S. purpuratus* repeat type 4 (GDQD) are not part of the homologous repeat sequence in He185/333. Most, but not all, He185/333 repeats had homologues amongst Sp185/333 sequences, as some repeats were unique to each species. For example, although the type 4 repeat sequence in He185/333 was homologous to a sequence stretch in Sp185/333, the latter was not repeated in Sp185/333. Finally, repeat type 2 of *H. erythrogramma* is present up to five times (four complete in tandem plus one incomplete repeats). The homologous sequences in Sp185/333 are composed of types 2, 3 and 4.
(DOCX)

Table S1 **Genbank accession numbers of He185/333 and Sp185/333 sequences.**
(DOC)

Table S2 **Tamura-Nei genetic distance matrix of phylogenetic comparison between 25 He185/333 and 25 Sp185/333 cDNA sequences.** The rates are assumed to follow gamma distribution with shape parameter  = 0.8094. The distances in light and dark gray shadings represent intra-species comparisons, while those without background shading represent comparisons between He185/333 and Sp185/333 sequences.
(XLSX)

Table S3 **Summary of diversity analysis carried out on He185/333 and Sp185/333 sequences.** Only those codons (codon#) that are positively or negatively selected are indicated in this table. The diversity analysis was carried out using three separate algorithms (SLAC, FEL and iFEL) and the table indicates whether codons are positively (+) or negatively (−) selected according to each of the algorithms. Codons are considered to be under selection (+ or −) if two or more of the analytical algorithms indicate significant selection pressure (columns entitled ‘consensus’). Blank columns specify codons that are not considered to be under significant selection by an algorithm.
(DOC)

## References

[1] Kurtz J (2005) Specific memory within innate immune systems. Trends in Immunology 26: 186–192. 15797508 10.1016/j.it.2005.02.001

[2] Roth O, Kurtz J (2009) Phagocytosis mediates specificity in the immune defence of an invertebrate, the woodlouse Porcellio scaber (Crustacea: Isopoda). Developmental and Comparative Immunology 33: 1151–1155. 19416736 10.1016/j.dci.2009.04.005

[3] Kurtz J, Armitage S (2006) Alternative adaptive immunity in invertebrates. Trends in Immunology 27: 493–496. 16979938 10.1016/j.it.2006.09.001

[4] Dong Y, Taylor HE, Dimopoulos G (2006) AgDscam, a hypervariable immunoglobulin domain-containing receptor of the Anopheles gambiae innate immune system. PLoS Biology 4: e229. 16774454 10.1371/journal.pbio.0040229 PMC1479700

[5] Sadd BM, Schmid-Hempel P (2006) Insect immunity shows specificity in protection upon secondary pathogen exposure. Current Biology 16: 1206–1210. 16782011 10.1016/j.cub.2006.04.047

[6] Little TJ, Kraaijeveld AR (2004) Ecological and evolutionary implications of immunological priming in invertebrates. Trends in Ecology and Evolution 19: 58–60. 16701227 10.1016/j.tree.2003.11.011

[7] Pancer Z (2000) Dynamic expression of multiple scavenger receptor cysteine-rich genes in coelomocytes of the purple sea urchin. Proceedings of the National Academy of Sciences of the United States of America 97: 13156–13161. 11069281 10.1073/pnas.230096397 PMC27194

[8] Hibino T, Loza-Coll M, Messier C, Majeske AJ, Cohen AH, et al (2006) The immune gene repertoire encoded in the purple sea urchin genome. Developmental Biology 300: 349–365. 17027739 10.1016/j.ydbio.2006.08.065

[9] Rast JP, Smith LC, Loza-Coll M, Hibino T, Litman GW (2006) Genomic insights into the immune system of the sea urchin. Science 314: 952–956. 17095692 10.1126/science.1134301 PMC3707132

[10] Pasquier LD (2006) Germline and somatic diversification of immune recognition elements in Metazoa. Immunology Letters 104: 2–17. 16388857 10.1016/j.imlet.2005.11.022

[11] Watson FL, Püttmann-Holgado R, Thomas F, Lamar DL, Hughes M, et al (2005) Extensive diversity of Ig-superfamily proteins in the immune system of insects. Science 309: 1874–1878. 16109846 10.1126/science.1116887

[12] Zhang SM, Loker ES (2003) The FREP gene family in the snail Biomphalaria glabrata: additional members, and evidence consistent with alternative splicing and FREP retrosequences. Fibrinogen-related proteins. Developmental and Comparative Immunology 27: 175–187. 12590969 10.1016/s0145-305x(02)00091-5

[13] Cannon JP, Haire RN, Litman GW (2002) Identification of diversified genes that contain immunoglobulin-like variable regions in a protochordate. Nature Immunology 3: 1200–1207. 12415263 10.1038/ni849

[14] Södergren E, Weinstock GM, Davidson EH, Cameron RA, Gibbs R a, et al (2006) The genome of the sea urchin Strongylocentrotus purpuratus. Science 314: 941–952. 17095691 10.1126/science.1133609 PMC3159423

[15] Nair SV, Del Valle H, Gross PS, Terwilliger DP, Smith LC (2005) Macroarray analysis of coelomocyte gene expression in response to LPS in the sea urchin. Identification of unexpected immune diversity in an invertebrate. Physiological Genomics 22: 33–47. 15827237 10.1152/physiolgenomics.00052.2005

[16] Terwilliger DP, Buckley KM, Mehta D, Moorjani PG, Smith LC (2006) Unexpected diversity displayed in cDNAs expressed by the immune cells of the purple sea urchin, Strongylocentrotus purpuratus. Physiological Genomics 26: 134–144. 16837652 10.1152/physiolgenomics.00011.2006

[17] Terwilliger DP, Buckley KM, Brockton V, Ritter NJ, Smith LC (2007) Distinctive expression patterns of 185/333 genes in the purple sea urchin, Strongylocentrotus purpuratus: an unexpectedly diverse family of transcripts in response to LPS, beta-1, 3-glucan, and dsRNA. BMC Molecular Biology 8: 16. 17331248 10.1186/1471-2199-8-16 PMC1831783

[18] Buckley KM, Smith LC (2007) Extraordinary diversity among members of the large gene family, 185/333, from the purple sea urchin, Strongylocentrotus purpuratus. BMC Molecular Biology 8: 68. 17697382 10.1186/1471-2199-8-68 PMC1988830

[19] Buckley KM, Munshaw S, Kepler TB, Smith LC (2008) The 185/333 gene family is a rapidly diversifying host-defense gene cluster in the purple sea urchin Strongylocentrotus purpuratus. Journal of Molecular Biology 379: 912–928. 18482736 10.1016/j.jmb.2008.04.037

[20] Miller C, Buckley KM, Easley RL, Smith LC (2010) An Sp185/333 gene cluster from the purple sea urchin and putative microsatellite-mediated gene diversification. BMC Genomics 11: 575. 20955585 10.1186/1471-2164-11-575 PMC3091723

[21] Buckley KM, Terwilliger DP, Smith LC (2008) Sequence Variations in 185/333 Messages from the Purple Sea Urchin Suggest Posttranscriptional Modifications to Increase Immune Diversity. The Journal of Immunology 181: 8585–8594. 19050278 10.4049/jimmunol.181.12.8585

[22] Brockton V, Henson JH, Raftos DA, Majeske AJ, Kim YO, et al (2008) Localization and diversity of 185/333 proteins from the purple sea urchin – unexpected protein-size range and protein expression in a new coelomocyte type. Journal of Cell Science 121: 339–348. 18198192 10.1242/jcs.012096

[23] Ghosh J, Buckley KM, Nair SV, Raftos DA, Miller C, et al (2010) Sp185/333: a novel family of genes and proteins involved in the purple sea urchin immune response. Developmental and Comparative Immunology 34: 235–245. 19887082 10.1016/j.dci.2009.10.008

[24] Dheilly NM, Nair SV, Smith LC, Raftos DA (2009) Highly variable immune-response proteins (185/333) from the sea urchin, Strongylocentrotus purpuratus: proteomic analysis identifies diversity within and between individuals. The Journal of Immunology 182: 2203–2212. 19201874 10.4049/jimmunol.07012766

[25] Dheilly NM, Birch D, Nair SV, Raftos DA (2011) Ultrastructural localization of highly variable 185/333 immune response proteins in the coelomocytes of the sea urchin, Heliocidaris erythrogramma. Immunology and Cell Biology: 1–9. 10.1038/icb.2011.3 21577232

[26] Lehrach H, Diamond D, Wozney JM, Boedtker H (1977) RNA Molecular Weight Determinations by Gel Electrophoresis under Denaturing Conditions, a Critical Reexamination. Biochemistry 16: 4743–4751. 911786 10.1021/bi00640a033

[27] Hall TA (1999) A user-friendly biological sequence alignment editor and analysis program for Windows 95/98/NT. Nucl Acids Symp Ser 41: 95–98.

[28] Altschul SF, Madden TL, Schäffer AA, Zhang J, Zhang Z, et al (1997) Gapped BLAST and PSI-BLAST: a new generation of protein database search programs. Nucleic Acids Research 25: 3389–3402. 9254694 10.1093/nar/25.17.3389 PMC146917

[29] Thompson JD, Higgins DG, Gibson TJ (1994) CLUSTAL W: improving the sensitivity of progressive multiple sequence alignment through sequence weighting, position-specific gap penalties and weight matrix choice. Nucleic acids research 22: 4673–4680. 7984417 10.1093/nar/22.22.4673 PMC308517

[30] Larkin MA, Blackshields G, Brown NP, Chenna R, McGettigan PA, et al (2007) Clustal W and Clustal X version 2.0. Bioinformatics 23: 2947–2948. 17846036 10.1093/bioinformatics/btm404

[31] Swofford DL (2003) PAUP*. Phylogenetic Analysis Using Parsimony (*and Other Methods). Version 4. Sunderland, Massachusetts: Sinauer Associates. p.

[32] Posada D, Crandall KA (1998) MODELTEST: testing the model of DNA substitution. BIOINFORMATICS APPLICATIONS NOTE 14: 817–818. 10.1093/bioinformatics/14.9.817 9918953

[33] Delport W, Poon AFY, Frost SDW, Kosakovsky Pond SL (2010) Datamonkey 2010: a suite of phylogenetic analysis tools for evolutionary biology. Bioinformatics 26: 2455–2457. 20671151 10.1093/bioinformatics/btq429 PMC2944195

[34] Pond SLK, Frost SDW, Muse SV (2005) HyPhy: hypothesis testing using phylogenies. Bioinformatics 21: 676–679. 15509596 10.1093/bioinformatics/bti079

[35] Kosakovsky Pond SL, Frost SDW (2005) Not so different after all: a comparison of methods for detecting amino acid sites under selection. Molecular Biology and Evolution 22: 1208–1222. 15703242 10.1093/molbev/msi105

[36] Pond SLK, Frost SDW, Grossman Z, Gravenor MB, Richman DD, et al (2006) Adaptation to different human populations by HIV-1 revealed by codon-based analyses. PLoS Computational Biology 2: e62. 16789820 10.1371/journal.pcbi.0020062 PMC1480537

[37] Cavener DR, Ray SC (1991) Eukaryotic start and stop translation sites. Nucleic Acids Research 19: 3185–3192. 1905801 10.1093/nar/19.12.3185 PMC328309

[38] Bendtsen JD, Nielsen H, von Heijne G, Brunak S (2004) Improved prediction of signal peptides: SignalP 3.0. Journal of Molecular Biology 340: 783–795. 15223320 10.1016/j.jmb.2004.05.028

[39] Krogh A, Larsson B, von Heijne G, Sonnhammer EL (2001) Predicting transmembrane protein topology with a hidden Markov model: application to complete genomes. Journal of molecular biology 305: 567–580. 11152613 10.1006/jmbi.2000.4315

[40] Pierleoni A, Martelli PL, Casadio R (2008) PredGPI: a GPI-anchor predictor. BMC Bioinformatics 9: 392. 18811934 10.1186/1471-2105-9-392 PMC2571997

[41] Caragea C, Sinapov J, Silvescu A, Dobbs D, Honavar V (2007) Glycosylation site prediction using ensembles of Support Vector Machine classifiers. BMC Bioinformatics 8: 438. 17996106 10.1186/1471-2105-8-438 PMC2220009

[42] Blom N, Gammeltoft S, Brunak S (1999) Sequence and structure-based prediction of eukaryotic protein phosphorylation sites. Journal of Molecular Biology 294: 1351–1362. 10600390 10.1006/jmbi.1999.3310

[43] Smith LC, Rast JP, Brockton V, Terwilliger DP, Nair SV, et al (2006) The sea urchin immune system. Invertebrate Survival Journal 3: 25–39.

[44] Giancotti FG, Ruoslahti E (1999) Integrin Signaling. Science 285: 1028–1033. 10446041 10.1126/science.285.5430.1028

[45] Miranti CK, Brugge JS (2002) Sensing the environment: a historical perspective on integrin signal transduction. Nature Cell Biology 4: E83–90. 11944041 10.1038/ncb0402-e83

[46] Pederson HG, Johnson CR (2007) Growth and age structure of sea urchins (Heliocidaris erythrogramma) in complex barrens and native macroalgal beds in eastern Tasmania. ICES Journal of Marine Science 65: 1–11.

[47] Palumbi SR, Lessios HA (2005) Evolutionary animation: How do molecular phylogenies compare to Mayr's reconstruction of speciation patterns in the sea? PNAS. 102: 6566–6572. 10.1073/pnas.0501806102 PMC1131860 15851681

[48] Lessios HA, Zigler KS (2012) Rates of sea urchin bindin evolution. In Rapidly Evolving Genes and Genetic Systems. Rama S. Singh, Jianping Xu, and Rob J. Kulathinal (eds). Oxford University Press.

