# Supplementary material for: Characterization of the Highly Variable Immune Response Gene Family, He185/333, in the Sea Urchin, Heliocidaris erythrogramma
Source: PLoS One. 2014 Oct 21;9(10):e62079. doi: 10.1371/journal.pone.0062079 (PMC4204807; doi:10.1371/journal.pone.0062079)
Supplement: Figure S2 — Nucleotide sequence alignment for 112 He185/333 cDNAs generated in Clustal W and BioEdit. The best alignment was obtained by insertion of large gaps, resulting in 26 sequence blocks (elements). The 26 elements are numbered along the top and separated by vertical black lines. The first and last three nucleotides represent the start and stop codon, respectively. The alignment shows full-length cDNAs irrespective of mutations that lead to missense and early termination upon translation. The cDNAs with such mutations are translated accordingly in Fig. S4. (DOC) [file pone.0062079.s002.doc]

Leader

2

4

1

3

**....|....| ....|....| ....|....| ....|....| ....|....| ....|....| ....|....| ....|....| ....|....| ....|....| ....|....| ....|....| ....|....| ....|....| ....|....| ....|....|**

**5 15 25 35 45 55 65 75 85 95 105 115 125 135 145 155**

**He185/333_cDNA_0001 ATGGAGTTGA AAGTGACACT GATCGTTACC CTTGTGGCTG CTATTACCAT CTCAGTTCAT GCGCAAAGAG AACCGGGAGG AAGAGGAAAT GGCAGAGAGA GGGGACAAGG TCGCTTCGGA GGAAGGCCAG GATCTGATAG ATCCCAAATG ATGGGTGGAC 160**

**He185/333_cDNA_0002 ATGGAGTTGA AAGTAACACT GATCGTTACC CTTGTGGCTG CTATTACCAT CTCAGTTCAT GCGCAAAGAG AACCGGGAGG AAGAGGAAAT GGCAGAGAGA GGGGACAAGG TCGCTTCGGA GGAAGGCCAG GATCTGATAG ATCCCAAATG ATGGGTGGAC 160**

**He185/333_cDNA_0003 ATGGAGTTGA AAGTGACACT GATCGTTACC CTTGTGGCTG CTATTACCAT CTCAGTTCAT GCGCAAAGAG AACCGGGAGG AAGAGGAAAT GGCAGAGAGA GGGGACAAGG TCGCTTCGGA GGAAGGCCAG GATCTGATAG ATCCCAAATG ATGGGTGGAC 160**

**He185/333_cDNA_0004 ATGGAGTTGA AAGTGACACT GATCGTTACC CTTGTGGCTG CTATTACCAT CTCAGTTCAT GCGCAAAGAG AACCGGGAGG AAGAGGAAAT GGCAGAGAGA GGGGACAAGG TCGCTTCGGA GGAAGGCCAG GATCTGATAG ATCCCAAATG ATGGGTGGAC 160**

**He185/333_cDNA_0005 ATGGAGTTGA AAGTGACACT GATCGTTACC CTTGTGGCTG CTATTACCAT CTCAGTTCAT GCGCAAAGAG AACCGGGGGG AAGAGGAAAT GGCAGAGAGA GGGGACAAGG TCGCTTCGGA GGAAGGCCAG GATCTGGTAG ATCCCAAATG ATGGGTGGAC 160**

**He185/333_cDNA_0006 ATGGAGTTGA AAGTGACACT GATCGTTACC CTTGTGGCTG CTATTACCAT CTCAGTTCAT GCGCAAAGAG AACCGGGAGG AAGAGGAAAT GGCAGAGAGA GGGGACAAGG TCGCTTCGGA GGAAGGCCAG GATCTGATAG ATCCCAAATG ATGGGTGGAC 160**

**He185/333_cDNA_0007 ATGGAGTTGA AAGTGACACT GATCGTTACC CTTGTGGCTG CTATTACCAT CTCAGTTCAT GCGCAAAGAG AACCGGGAGG AAGAGGAAAT GGCAGAGAGA GGGGACAAGG TCGCTTCGGA GGAAGGCCAG GATCTGATAG ATCCCAAATG ATGGGTGGAC 160**

**He185/333_cDNA_0008 ATGGAGTTGA AAGTGACACT GATCGTTACC CTTGTGGCTG CTATTACCAT CTCAGTTCAT GCGCAAAGAG AACCGGGAGG AAGAGGAAAT GGCAGAGAGA GGGGACAAGG TCGCTTCGGA GGAAGGCCAG GATCTGATAG ATCCCAAATG ATGGGTGGAC 160**

**He185/333_cDNA_0009 ATGGAGTTGA AAGTGACACT GATCGTTACC CTTGTGGCTG CTATTACCAT CTCAGTTCAT GCGCAAAGAG AACCGGGAGG AAGAGGAAAT GGCAGAGAGA GGGGACAAGG TCGCTTCGGA GGAAGGCCAG GATCTGATAG ATCCCAAATG ATGGGTGGAC 160**

**He185/333_cDNA_0010 ATGGAGTTGA AAGTGACACT GATCGTTACC CTTGTGGCTG CTATTACCAT CTCAGTTCAT GCGCAAAGAG AACCGGGAGG AAGAGGAAAT GGCAGAGAGA GGGGACAAGG TCGCTTCGGA GGAAGGCCAG GATCTGATAG ATCCCAAATG ATGGGTGGAC 160**

**He185/333_cDNA_0011 ATGGAGTTGA AAGTGACACT GATCGTTACC CTTGTGGCTG CTATTACCAT CTCAGTTCAT GCGCAAAGAG AACCGGGAGG AAGAGGAAAT GGCAGAGAGA GGGGACAAGG TCGTTTCGGA GGAAGGCCAG GATCTGATAG ATCCCAAATG ATGGGTGGAC 160**

**He185/333_cDNA_0012 ATGGAGTTGA AAGTGACACT GATCGTTGCC TTTGTGACTG CTATTACCAT CTCAGTTCAT GCTCAAAGAG CACGAGGAGG AAGAAGAAAT GGCAGAGAGA GGGGACAAGG TCGCTTCGGA GGAAGTCCAG GATCTGATAG ACCCCAAATG ACGGGTGGAC 160**

**He185/333_cDNA_0013 ATGGAGTTGA AAGTGACACA GATCGTTACC CTTGTGGCTG CTATTACCAT CTCAATTCAT GCGCAAAGAG AACCGGGAGG AAGAGGAAAT GGCAGAGAGA GGGGACAAGG TCGCTTCGGA GGAAGGCCAG GATCTGATAG ATCCCAAATG ATGGGTGGAC 160**

**He185/333_cDNA_0014 ATGGAGTTGA AAGTGACACT GATCGTTGCC CTTGTGGCTG CTATTACCAT CTCAGTTCAT GCTCAAAGAG CACGAGGAGG AAGAGGATAT GGCAGAAAGA GGGGACAAGG TCGCTTCGGA GGAAGTCCAG GATCTGATAG ACCCCAAATG ACGGGTGGAC 160**

**He185/333_cDNA_0015 ATGGAGTTGA AAGTGACACT GATCGTTGCC CTTGTGACTG CTATTACCAT CTCAGTTCAT GCTCAAAGAG CACGAGGAGG AAGAAGAAAT GGCAGAGAGA GGGGACAAGG TCGCTTCGGA GGAAGTCCAG GATCTGATAG ACCCCAAATG ACGGGTGGAC 160**

**He185/333_cDNA_0016 ATGGAGTTGA AAGTGACACT GATCGTTGCC CTTGTGACTG CTATTACCAT CTCAGTTCAT GCTCAAAGAG CACGAGGAGG AAGAAGAAAT GGCAGAGTGA GGGGACAAGG TCGCTTCGGA GGAAGTCCAG GATCTGATAG ATCCCAAATG ACGGGTGGAC 160**

**He185/333_cDNA_0017 ATGGAGTTGA AAGTGACACT GATCGTTACC CTTGTGGCTG CTATTACCAT CTCAGTTCAT GCGCAAAGAG AACCGGGAGG AAGAGGAAAT GGCAGAGAGA GGGGACAAGG TCGCTTCGGA GGAAGGCCAG GATCTGATAG ATCCCAAATG ATGGGTGGAC 160**

**He185/333_cDNA_0018 ATGGAGTTGA AAGTGACACT GATCGTTACC CTTGTGGCTG CTATTACCAT CTCAGTTCAT GCGCAAAGAG AACCGGGAGG AAGAGGAAAT GGCAGAGAGA GGGGACAAGG TCGCTTCGGA GGAAGGCCAG GATCTGATAG ATCCCAAATG ATGGGTGGAC 160**

**He185/333_cDNA_0019 ATGGAGTTGA AAGTGACACT GATCGTTACC CTTGTGGCTG CTATTACCAT CTCAGTTCAT GCGCAAAGAG AACCGGGAGG AAGAGGAAAT GGCAGAGAGA GGGGACAAGG TCGCTTCGGA GGAAGGCCAG GATCTGATAG ATCCCAAATG ATGGGTGGAC 160**

**He185/333_cDNA_0020 ATGGAGTTGA AAGTGACACT GATCGTTACC CTTGTGGCTG CTATTACCAT CTCAGTTCAT GCGCAAAGAG AACCGGGAGG A--------- ---------- ---------- ---------- ---AGGCCAG GATCTGATAG ATCCCAAATG ATGGGTGGAC 118**

**He185/333_cDNA_0021 ATGGAGTTGA AAGTGACACT GATCGTTACC CTTGTGGCTG CTATTACCAT CTCAGTTCAT GCGCAAAGAG AACCGGGAGG AAGAGGAAAT GGCAGAGAGA GGGGACAAGG TCGCTTCGGA GGAAGGCCAG GATCTGATAG ATCCCAAATG ATGGGTGGAC 160**

**He185/333_cDNA_0022 ATGGAGTTGA AAGTGACACT GATCGTTGCC CTTGTGACTG CTATTACCAT CTCAGTTCAT GCTCAAAGAG CACGAGGAGG AAGAAGAAAT GGCAGAGAGA GT-------- ---------- ---------- ---------- ---------- ---------- 102**

**He185/333_cDNA_0023 ATGGAGTTGA AAGTGACACT GATCGTTGCC CTTGTGGCTG CTATTACCAT CTCAGTTCAT GCACAAAGAG GCCGGGGCGG AAGAGGAAAT GGCAAAGAGA GGGGACAAGG TCGCTTCGGA GGAAGGCCAG GATCC---AG ACCCCAAATG ATGGGTGGAC 157**

**He185/333_cDNA_0024 ATGGAGTTGA AAGTGACACT GATCGTTGCC CTTGTGGCTG CTATTACCAT CTCAGTTCAT GCTCAAAGAG CACGAGGAGG AAGAGGATAT GGCAGAAAGA GGGGACAAGG TCGCTTCGGA GGAAGTCCAG GATCTGATAG ACCCCAAATG ACGGGTGGAC 160**

**He185/333_cDNA_0025 ATGGAGTTGA AAGTGACACT GATCGTTGCC CTTGTGGCTG CTATTACCAT CTCAGTTCAT GCACAAAGAG GCCGGGGCGG AAGAGGAAAT GGCAGAGAGA GGGGACAAGG TCGCTTCGGA GGAAGGCCAG GATCC---AG ACCCCAAATG ATGGGTGGAC 157**

**He185/333_cDNA_0026 ATGGAGTTGA AAGTGACACT GATCGTTGCC CTTGTGGCTG CTATTACCAT CTCAGTTCAT GCTCAAAGAG CACGAGGAGG AAGAGGATAT GGCAGAAAGA GGGGACAAGG TCGCTTCGGA GGAAGCCCAG GATCTGATAG ACCCCAAATG ACGGGTGGAC 160**

**He185/333_cDNA_0027 ATGGAGTTGA AAGTGACACT GATCGTTGCC CTTGTGGCTG CTATTACCAT CTCAGTTCAT GCACAAAGAG GCCGGGGCGG AAGAGGAAAT GGCAAAGAGA GGGGACAAGG TCGCTTCGGA GGAAGGCCAG GATCC---AG ACCCCAAATG ATGGGTGGAC 157**

**He185/333_cDNA_0028 ATGGAGTTGA AAGTGACACT GATCGTTGCC CTTGTGGCTG CTATTACCAT CTCAGTTCAT GCTCAAAGAG CACGAGGGGG AAGAGGATAT GGCAGAAAGA GGGGACAAGG TCGCTTCGGA GGAAGTCCAG GATCTGATAG ACCCCAAATG ACGGGTGGAC 160**

**He185/333_cDNA_0029 ATGGAGTTGA AAGTGACACT GATCGTTGCC CTTGTGGCTG CTATTACCAT CTCAGTTCAT GCACAAAGAG GCCGGGGCGG AAGAGGAAAT GGCAGAGAGA GGGGACAAGG TCGCTTCGGA GGAAGGCCAG GATCC---AG ACCCCAAATG ATGGGTGGAC 157**

**He185/333_cDNA_0030 ATGGAGTTGA AAGTGACACT GATCGTTGCC CTTGTGGCTG CTATTACCAT CTCAGTTCAT GCTCAAAGAG CACGAGGAGG AAGAGGATAT GGCGGAAAGA GGGGACAAGG TCGCTTCGGA GGAAGTCCAG GATCTGATAG ACCCCAAATG ACGGGTGGAC 160**

**He185/333_cDNA_0031 ATGGAGTTGA AAGTGACACT GATCGTTGCC CTTGTGGCTG CTATTACCAT CTCAGTTCAT GCACAAAGAG GCCGGGGCGG AAGAGGAAAT GGCAAAGAGA GGGGACAAGG TCGCTTCGGA GGAAGGCCAG GATCC---AG ACCCCAAATG ATGGGTGGAC 157**

**He185/333_cDNA_0032 ATGGAGTTGA AAGTGACACT GATCGTTGCC CTTGTGGCTG CTATTACCAT CTCAGTTCAT GCTCAAAGAG CACGAGGAGG AAGAGGATAT GGCAGAAAGA GGGGACAAGG TCGCTTCGGA GGAAGTCCAG GATCTGATAG ACCCCAAATG ACGGGTGGAC 160**

**He185/333_cDNA_0033 ATGGAGTTGA AAGTGACACT GATCGTTGCC CTTGTGGCTG CTATTACCAT CTCAGTTCAT GCACAAAGAG GCCGGGGCGG AAGAGGAAAT GGCAGAGAGA GGGGACAAGG TCGCTTCGGA GGAAGGCCAG GATCC---AG ACCCCAAATG ATGGGTGGAC 157**

**He185/333_cDNA_0034 ATGGAGTTGA AAGTGACACT GATCGTTGCC CTTGTGGCTG CTATTACCAT CTCAGTTCAT GCACAAAGAG GCCGGGGCGG AAGAGGAAAT GGCAGAGAGA GGGGACAAGG TCGCTTCGGA GGAAGGCCAG GATCC---AG ACCCCAAATG ATGGGTGGAC 157**

**He185/333_cDNA_0035 ATGGAGTTGA AAGGGACACT TATCGTCGCC ATTTTGGCTG CTATTACCAT GTCAGTTCAT GCACAAAGAG ACCGGGGCGG AAGAGGAAAT GCCAGAGAGA GGGGACAAGG TCGCTTCGGA GGAAGGCCAG GATCTAATAG ACCCCAAATG ATGGGTGGAC 160**

**He185/333_cDNA_0036 ATGGAGTTGA AAGTGACACT GATCGTTGCC CTTGTGGCTG CTATTACCAT CTCAGCTCAT GCACAAAGAG AACGGGGAGG AAGAGGAAAT GGCAGAGAGA GGGGACAAGG TCGCTTCGGA GGAAGGCCAG GATCTGATAG ATCCCAAATG ACGGGTGGAC 160**

**He185/333_cDNA_0037 ATGGAGTTGA AAGTGACACT GATCGTTGCC CTTGTGGCTG CTATTACCAT CTCAGTTCAT GCTCAAAGAG CACGAGGAGG AAGAGGATAT GGCAGAAAGA GGGGACAAGG TCGCTTCGGA GGAAGTCCAG GACCTGATAG ACCCCAAATG ACGGGTGGAC 160**

**He185/333_cDNA_0038 ATGGAGTTGA AAGTGACACT GATCGTTACC CTTGTGGCTG CTATTACCAT CTCAGTTCAT GCGCAAAGAG AACCGGGAGG AAGAGGAAAT GGCAGAGAGA GGGGACAAGG TCGCTTCGGA GGAAGGCCAG GATCTGATAG ATCCCAAATG ATGGGTGGAC 160**

**He185/333_cDNA_0039 ATGGAGTTGA AAGTGACACT GATCGTTACC CTTGTGGCTG CTATTACCAT CTCAGTTCAT GCGCAAAGAG AACCGGGAGG AAGAGGAAAT GGCAGAGAGA GGGGACAAGG TCGCTTCGGA GGAAGGCCAG GATCTGATAG ATCCCAAATG ATGGGTGGAC 160**

**He185/333_cDNA_0040 ATGGAGTTGA AAGTGACACT GATCGTTGCC CTTGTGGCTG CTATTACCAT CTCAGTTCAT GCGCAAAGAG AACCGGGAGG AAGAGGAAAT GGCAGAGAGA GGGGACAAGG TCGCTTCAGA GGAAGGCCAG GATCTGATAG ATCCCAAATG ATGGGTGGAC 160**

**He185/333_cDNA_0041 ATGGAGTTGA AAGTGACACT GATCGTTACC CTTGTGGCTG CTATTACCAT CTCAGTTCAT GCGCAAAGAG AACCGGGAGG AAGAGGAAAT GGCAGAGAGA GGGGACAAGG TCGCTTCGGA GGAAGGCCAG GATCTGATAG ATCCCAAATG ATGGGTGGAC 160**

**He185/333_cDNA_0042 ATGGAGTTGA AAGTGACACT GATCGTTACC CTTGTGGCTG CTATTACCAT CTCAGTTCAT GCGCAAAGAG AACCGGGAGG AAGAGGAAAT GGCAGAGAGA GGGGACAAGG TCGCTTCGGA GGAAGGCCAG GATCTGATAG ATCCCAAATG ATGGGTGGAC 160**

**He185/333_cDNA_0043 ATGGAGTTGA AAGTGACACT GATCGTTGCC CTTGTGGCTG CTATTACCAT CTCAGTTCAT GCACAAAGAG GCCGGGGCGG AAGAGGAAAT GGCAAAGAGA GGGGACAAGG TCGCTTCGGA GGAAGGCCAG GATCC---AG ACCCCAAATG ATGGGTGGAC 157**

**He185/333_cDNA_0044 ATGGAGTTGA AAGTGACACT GATCGTTACC CTTGTGGCTG CTATTACCAT CTCAGTTCAT GCGCAAAGAG AACCGGGAGG AAGAGGAAAT GGCAGAGAGA GGGGACAAGG TCGCTTCGGA GGAAGGCCAG GATCTGATAG ATCCCAAATG ATGGGTGGAC 160**

**He185/333_cDNA_0045 ATGGAGTTGA AAGTGACACT GATCGTTGCC CTTGTGGCTG CTATTACCAT CTCAGTTCAT GCGCAAAGAG AACCGGGAGG AAGAGGAAAT GGCAGAGAGA GGGGACAAGG TCGCTTCAGA GGAAGGCCAG GATCTGATAG ATCCCAAATG ATGGGTTGAC 160**

**He185/333_cDNA_0046 ATGGAGTTGA AAGTGACACT GATCGTTGCC CTTGTGGCTG CTATTACCAT CTCAGTTCAT GCGCAAAGAG AACCGGGAGG AAGAGGAAAT GGCAGAGAGA GGGGACAAGG TCGCTTCAGA GGAAGGCCAG GATCTGATAG ATCCCAAATG ATGGGTGGAC 160**

**He185/333_cDNA_0047 ATGGAGTTGA AAGTGACACT GATCGTTGCC CTTGTGGCTG CTATTACCAT CTCAGTTCAT GCTCAAAGAG CACGAGGAGG AAGAGGATAT GGCAGAAAGA GGGGACAAGC TCGCTTCGGA GGAAGTCCAG GATCTGATAG ACCCCAAATG ACGGGTGGAC 160**

**He185/333_cDNA_0048 ATGGAGTTGA AAGTGACACT GATCGTTACC CTTGTGGCTG CTATTACCAT CTCAGTTCAT GCGCAAAGAG AACCGGGAGG AAGAGGAAAT GGCAGAGAGA GGGGACAAGG TCGCTTCGGA GGAAGGCCAG GATCTGATAG ATCCCAAATG ATGGGTGGAC 160**

**He185/333_cDNA_0049 ATGGAGTTGA AAGTGACACT GATCGTTACC CTTGTGGCTG CTATTACCAT CTCAGTTCAT GCGCAAAGAG AACCGGGAGG AAGAGGAAAT GGCAGAGAGA GGGGACAAGG TCGCTTCGGA GGAAGGCCAG GATCTGATAG ATCCCAAATG ATGGGTGGAC 160**

**He185/333_cDNA_0050 ATGGAGTTGA AAGTGACACT GATCGTTACC CTTGTGGCTG CTATTACCAT CGCAGTTCAT GCGCAAAGAG AACCGGGAGG AAGAGGAAAT GGCAGAGAGA GGGGACAAGG TCGCTTCGGA GGAAGGCCAG GATCTGATAG ATCCCAAATG ATGGGTGGAC 160**

**He185/333_cDNA_0051 ATGGAGTTGA AAGTGACACT GATCGTTGCC CTTGTGGCTG CTATTACCAT CTCAGTTCAT GCGCAAAGAG AACCGGGAGG AAGAGGAAAT GGCAGAGAGA GGGGACAAGG TCGCTTCAGA GGAAGGCCAG GATCTGATAG ATCCCAAATG ATGGGTGGAC 160**

**He185/333_cDNA_0052 ATGGAGTTGA AAGTGACACT GATCGTTACC CTTGTGGCTG CTATTACCAT CTCAGTTCAT GCGCAAAGAG AACCGGGAGG AAGAGGAAAT GGCAGAGAGA GGGGACAAGG TCGCTTCGGA GGAAGGCCAG GATCTGATAG ATCCCAAATG ATGGGTGGAC 160**

**He185/333_cDNA_0053 ATGGAGTTGA AAGTGACACT GATCGTTGCC CTTGTGGCTG CTATTACCAT CTCAGTTCAT GCGCAAAGAG AACCGGGAGG AAGAGGAAAT GGCAGAGAGA GGGGACAAGG TCGCTTCAGA GGAAGGCCAG GATCTGATAG ATCCCAAATG ATGGGTGGAC 160**

**He185/333_cDNA_0054 ATGGAGTTGA AAGTGACACT GATCGTTGCC CTTGTGGCTG CTATTACCAT CTCAGTTCAT GCACAAAGAG GCCGGGGCGG AAGAGGAAAT GGCAAAGAGA GGGGACAAGG TCGCTTCGGA GGAAGGCCAG GATCC---AG ACCCCAAATG ATGGGTGGGC 157**

**He185/333_cDNA_0055 ATGGAGTTGA AAGTGACACT GATCGTTGCC CTTGTGGCTG CTATTACCAT CTCAGTTCAT GCTCAAAGAG CACGAGGAGG AAGAGGATAT GGCAGAAGGA GGGGACAAGG TCGCTTCGGA GGAAGTCCAG GATCTGATAG ACCCCAAATG ACGGGTGGAC 160**

**He185/333_cDNA_0056 ATGGAGTTGA AAGTGACACT GATCGTTGCC CTTGTGGCTG CTATTACCAT CTCAGTTCAT GCTCAAAGAG CACGAGGAGG AAGAGGATAT GGCAGAAAGA GGGGACAAGG TCGCTTCGGA GGAAGTCCAG GATTTGATAG ACCCCAAATG ACGGGTGGAC 160**

**He185/333_cDNA_0057 ATGGAGTTGA AAGTGACACT GATCGTTGCC CTTGTGGCTG CTATTACCAT CTCAGCTCAT GCACAAAGAG AACGGGGAGG AAGAGGAAAT GGCAGAGAGA GGAGACAAGG TCGCTTCGGA GGAAGGCCAG GATCTGATAG ATCCCAAATG ATGGGTGGAC 160**

**He185/333_cDNA_0058 ATGGAGTTGA AAGTGACACT GATCGTTGCC CTTGTGGCTG CTATTACCAT CTCAGCTCAT GCACAAAGAG AACGGGGAGG AAGAGGAAAT GGCAGAGAGA GGAGACAAGG TCGCTTCGGA GGAAGGCCAG GATCTGATAG ATCCCAAATG ATGGGTGGAC 160**

**He185/333_cDNA_0059 ATGGAGTTGA AAGTGACACT GATCGTTGCC CTTGTGGCTG CTATTACCAT CTCAGCTCAT GCACAAAGAG AACGGGGAGG AAGAGGAAAT GGCAGAGAGA GGGGACAAGG TCGCTTCGGA GGAAGGCCAG GATCTGATAG ATCCCAAATG ATGGGTGGAC 160**

**He185/333_cDNA_0060 ATGGAGTTGA AAGTGACACT TATCGTCGCC ATTTTGGCTG CTATTACCAT GTCAGTTCAT GCACAAAGAG ACCGGGGCGG AAGAGGAAAT GCCAGAGAGA GGGGACAAGG TCGCTTCGGA GGAAGGCCAG GATCTAATAG ACCCCAAATG ATGGGTGGAC 160**

**He185/333_cDNA_0061 ATGGAGTTGA AAGTGACACT GATCGTTGCC CTTGTGGCTG CTATTACCAT CTCAGCTCAT GCACAAAGAG AACGGGGAGG AAGAGGAAAT GGCAGAGAGA GGGGACAAGG TCGCTTCGGA GGAAGGCCAG GATCTGATAG ATCCCAAATG ATGGGTGGAC 160**

**He185/333_cDNA_0062 ATGGAGTTGA AAGTGACACT GATCGTTGCC CTTGTGGCTG CTATTACCAT CTCAGCTCAT GCACAAAGAG AACGGGGAGG AAGAGGAAAT GGCAGAGAGA GGGGACAAGG TCGCTTCGGA GGAAGGCCAG GATCTGATAG ATCCCAAATG ATGGGTGGAC 160**

**He185/333_cDNA_0063 ATGGAGTTGA AAGTGACACT GATCGTTGCC CTTGTGGCTG CTATTACCAT CTCAGCTCAT GCACAAAGAG AACGGGGAGG AAGAGGAAAT GGCAGAGAGA GGGGACAAGG TCGCTTCGGA GGAAGGCCAG GATCTGATAG ATCCCAAATG ATGGGTGGAC 160**

**He185/333_cDNA_0064 ATGGAGTTGA AAGTGACACT GATCGTTGCC CTTGTGGCTG CTATTACCAT CTCAGCTCAT GCACAAAGAG AACGGGGAGG AAGAGGAAAT GGCAGAGAGA GGGGACAAGG TCGCTTCGGA GGAAGGCCAG GATCTGATAG ATCCCAAATG ATGGGTGGAC 160**

**He185/333_cDNA_0065 ATGGAGTTGA AAGTGACACT GATCGTTGCC CCTGTGGCTG CTATTACCAT CTCAGCTCAT GCACAAAGAG AACGGGGAGG AAGAGGAAAT GGCAGAGAGA GGGGACAAGG TCGCTTCGGA GGAAGGCCAG GATCTGATAG ATCCCAAATG ATGGGTGGAC 160 He185/333_cDNA_0066 ATGGAGTTGA AAGTGACACT GATCGTTGCC CTTGTGGCTG CTATTACCAT CTCAGCTCAT GCACAAAGAG AACGGGGAGG AAGAGGAAAT GGCAGAGAGA GGGGACAAGG TCGCTTCGGA GGAAGGCCAG GATCTGATAG ATCCCAAATG ATGGGTGGAC 160**

**He185/333_cDNA_0067 ATGGAGTTGA AAGTGACACT GATCGTTGCC CTTGTGGCTG CTATTACCAT CTCAGCTCAT GCACAAAGAG AACGGGGAGG AAGAGGAAAT GGCAGAGAGA GGGGACAAGG TCGCTTCGGA GGAAGGCCAG GATCTGATAG ATCCCAAATG ATGGGTGGAC 160**

**....|....| ....|....| ....|....| ....|....| ....|....| ....|....| ....|....| ....|....| ....|....| ....|....| ....|....| ....|....| ....|....| ....|....| ....|....| ....|....|**

Leader

1

2

3

4

**5 15 25 35 45 55 65 75 85 95 105 115 125 135 145 155**

**He185/333_cDNA_0068 ATGGAGTTGA AAGTGACACT GATCGTTGCC CTTGTGGCTG CTATTACCAT CTCAGTTCAT GCGCAAAGAG AACCGGGAGG AAGAGGAAAT GGCAGAGAGA GGGGACAAGG TCGCTTCAGA GGAAGGCCAG GATCTGATAG ATCCCAAGTG ATGGGTGGAC 160**

**He185/333_cDNA_0069 ATGGAGTTGA AAGTGACACT GATCGTTACC CTTGTGGCTG CTATTACCAC CTCAGTTCAT GCGCAAAGAG AACCGGGAGG AAGAGGAAAT GGCAGAGAGA GGGGACAAGG TCGCTTCGGA GGAAGGCCAT GATCTGATAG ATCCCAAATG ATGGGTGGAC 160**

**He185/333_cDNA_0070 ATGGAGTTGA AAGTGACACT GATCGTTACC CTTGTGGCTG CTATTACCAT CTCAGTTCGT GCGCAAAGAG AACCGGGAGG AAGAGGAAAT GGCAGAGAGA GGGGACAAGG TCGCTTCGGA GGAAGGCCAG GATCTGATAG ATCCCAAATG ATGGGTGGAC 160**

**He185/333_cDNA_0071 ATGGAGTTGA AAGTGACACT GATCGTTACC CTTGTGGCTG CTATTACCAT CTCAGTTCAT GCGCAAAGAG AACCGGGAGG AAGAGGAAAT GGCAGAGAGA GGGGACAAGG TCGCTTCGGA GGAAGGCCAG GATCTGATAG ATCCCAAATG ATGGGTGGAC 160**

**He185/333_cDNA_0072 ATGGAGTTGA AAGTGACACC GATCGTTACC CTTGTGGCTG CTATTACCAT CTCAGTTCAT GCGCAAAGAG AACCGGGAGG AAGAGGAAAT GGCAGAGAGA GGGGACAAGG TCGCTTCGGA GGAAGGCCAG GATCTGATAG ATCCCAAATG ATGGGTGGAC 160**

**He185/333_cDNA_0073 ATGGAGTTGA AAGTGACACT GATCGTTACC CTTGTGGCTG CTATTACCAT CTCAGTTCAT GCGCAAAGAG AACCGGGAGG AAGAGGAAAT GGCAGAGAGA GGGGACAAGG TCGCTTCGGA GGAAGGCCAG GATCTGATAG ATCCCAAATG ATGGGTGGAC 160**

**He185/333_cDNA_0074 ATGGAGTTGA AAGTGACACT GATCGTTACC CTTGTGGCTG CTATTACCAT CTCAGTTCAT GCGCAAAGAG AACCGGGAGG AAGAGGAAAT GGCAGAGAGA GGGGACAAGG TCGCTTCGGA GGAAGGCCAG GATCCGATAG ATCCCAAATG ATGGGTGGAC 160**

**He185/333_cDNA_0075 ATGGAGTTGA AAGCGACACT GATCGTTGCC CTTGTGGCTG CTATTACCAT CTCAGCTCAT GCACAAGGAG AACGGGGAGG AAGAGGAAAT GGCAGAGAGA GGGGACAAGG TCGCTTCGGA GGAAGGCCAG GATCTGATAG ATCCCAAATG ATGGGTGGAC 160**

**He185/333_cDNA_0076 ATGGAGTTGA AAGTGACACT GATCGTTACC CTTGTGGCTG CTATTACCAT CTCAGTTCAT GCGCAAAGAG AACCGGGAGG AAGAGGAAAT GGCAGAGAGA GGGGACAAGG TCGCTTCGGA GGAAGGCCAG GATCTGATAG ATCCCAAATG ATGGGTGGAC 160**

**He185/333_cDNA_0077 ATGGAGTTGA AAGTGACACT GATCGTTGCC CTTGTGGCTG CTATTACCAT CTCAGTTCAT GCTCAAAGAG CACGAGGAGG AAGAGGATAT GGCAGAAAGA GGGGACAAGG TCGCTTCGGA GGAAGTCCAG GATCTGATAG ACCCCAAATG ACGGGTGGAC 160**

**He185/333_cDNA_0078 ATGGAGTTGA AAGTGACACT GATCGTTACC CTTGTGGCTG CTATTACCAT CTCAGTTCAT GCGCAAAGAG AACCGGGAGG AAGAGGAAAT GGCAGAGAGA GGGGACAAGG TCGCTTCGGA GGAAGGCCAG GATCTGATAG ATCCCAAATG ATGGGTGGAC 160**

**He185/333_cDNA_0079 ATGGAGTTGA AAGTGACACT GATCGTTACC CTTGTGGCTG CTATTACCAT CTCAGTTCAT GCGCAAAGAG AACCGGGAGG AAGAGGAAAT GGCAGAGAGA GGGGACAAGG TCGCTTCGGA GGAAGGCCAG GATCTGATAG ATCCCAAATG ATGGGTGGAC 160**

**He185/333_cDNA_0080 ATGGAGTTGA AAGTGACACT GATCGTTACC CTTGTGGCTG CTATTACCAT CTCAGTTCAT GCGCAAAGAG AACCGGGAGG AAGAGGAAAT GGCAGAGAGA GGGGACAAGG TCGCTTCGGA GGAAGGCCAG GATCTGATAG ATCCCAAATG ATGGGTGGAC 160**

**He185/333_cDNA_0081 ATGGAGTTGA AAGTGACACT GATCGTTGCC CTTGTGGCTG CTATTACCAT CTCAGTTCAT GCACAAAGAG GCCGGGGCGG AAGAGGAAAT GGCAGAGAGA GGGGACAAGG TCGCTTCGGA GGAAGGCCAG GATCC---AG ACCCCAAATG ATGGGTGGAC 157**

**He185/333_cDNA_0082 ATGGAGTTGA AAGTGACACT GATCGTTGCC CTTGTGGCTG CTATTACCAT CTCAGTTCAT GCGCAAAGAG AACCGGGAGG AAGAGGAAAT GGCAGAGAGA GGGGACAAGG TCGCTTCAGA GGAAGGCCAG GATCTGATAG ATCCCAAATG ATGGGTGGAC 160**

**He185/333_cDNA_0083 ATGGAGTTGA AAGTGACACT GATCGTTGCC CTTGTGGCTG CTATTACCAT CTCAGTTCAT GCACAAAGAG GCCGGGGCGG AAGAGGAAAT GGCAGAGAGA GGGGACAAGG TCGCTTCGGA GGAAGGCCAG GATCC---AG ACCCCAAATG ATGGGTGGAC 157**

**He185/333_cDNA_0084 ATGGAGTTGA AAGTGACACT GATCGTTGCC CTTGTGGCTG CTATTACCAT CTCAGTTCAT GCGCAAAGAG AACCGGGAGG AAGAGGAAAT GGCAGAGAGA GGGGACAAGG TCGCTTCAGA GGAAGGCCAG GATCTGATAG ATCCCAAATG ATGGGTGGAC 160**

**He185/333_cDNA_0085 ATGGAGTTGA AAGTGACACT GATCGTTGCC CTTGTGGCTG CTATTACCAT CTCAGTTCAT GCGCAAAGAG AACCGGGAGG AAGAGGAAAT GGCAGAGAGA GGGGACAAGG TCGCTTCAGA GGAAGGCCAG GATCTGATAG ATCCCAAATG ATGGGTGGAC 160**

**He185/333_cDNA_0086 ATGGAGTTGA AAGTGACACT GATCGTTGCC CTTGTGGCTG CTATTACCAT CTCAGTTCAT GCGCAAAGAG AACCGGGAGG AAGAGGAAAT GGCAGAGAGA GGGGACAAGG TCGCTTCAGA GGAAGGCCAG GATCTGATAG ATCCCAAATG ATGGGTGGAC 160**

**He185/333_cDNA_0087 ATGGAGTTGA AAGTGACACT GATCGTTGCC CTTGTGGCTG CTATTACCAT CTCAGTTCAT GCGCAAAGAG AACCGGGAGG AAGAGGAAAT GGCAGAGAGA GGGGACAAGG TCGCTTCAGA GGAAGGCCAG GATCTGATAG ATCCCAAATG ATGGGTGGAC 160**

**He185/333_cDNA_0088 ATGGAGTTGA AAGTGACACT GATCGTTGCC CTTGTGGCTG CTATTACCAT CTCAGCTCAT GCACAAAGAG AACGGGGAGG AAGAGGAAAT GGCAGAGAGA GGGGACAAGG TCGCTTCGGA GGAAGGCCAG GATCTGATAG ATCCCAAATG ATGGGTGGAC 160**

**He185/333_cDNA_0089 ATGGAGTTGA AAGTGACACT GATCGTTGCC CTTGTGGCTG CTATTACCAT CTCAGCTCAT GCACAAAGAG AACGGGGAGG AAGAGGAAAT GGCAGAGAGA GGGGACAAGG TCGCTTCGGA GGAAGGCCAG GATCTGATAG ATCCCAAAGG ATGGGTGGAC 160**

**He185/333_cDNA_0090 ATGGAGTTGA AAGTGACACT GATCGTTGCC CTTGTGGCTG CTATTACCAT CTCAGTTCAT GCGCAAAGAG AACCGGGAGG AAGAGGAAAT GGCAGAGAGA GGGGACAAGG TCGCTTCAGA GGAAGGCCAG GATCTGATAG ATCCCAAATG ATGGGTGGAC 160**

**He185/333_cDNA_0091 ATGGAGTTGA AAGTGACACT GATCGTTGCC CTTGTGGCTG CTATTACCAT CTCAGTTCAT GCGCAAAGAG AACCGGGAGG AAGAGGAAAT GGCAGAGAGA GGGGACAAGG TCGCTTCAGA GGAAGGCCAG GATCTGATAG ATCCCAAATG ATGGGTGGAC 160**

**He185/333_cDNA_0092 ATGGAGTTGA AAGGGACACT GATCGTTGCC CTTGTGGCTG CTATTACCAT CTCAGTTCAT GCGCAAAGAG AACCGGGAGG AAGAGGAAAT GGCAGAGAGA GGGGACAAGG TCGCTTCAGA GGAAGGCCAG GATCTGATAG ATCCCAAATG ATGGGTGGAC 160**

**He185/333_cDNA_0093 ATGGAGTTGA AAGTGACACT GATCGTTGCC CTTGTGGCTG CTATTACCAT CTCAGTTCAT GCGCAAAGAG AACCGGGAGG AAGAGGAAAT GGCAGAGAGA GGGGACAAGG TCGCTTCAGA GGAAGGCCAG GATCTGATAG ATCCCAAATG ATGGGTGGAC 160**

**He185/333_cDNA_0094 ATGGAGTTGA AAGTGACACT GATCGTTGCC CTTGTGGCTG CTATTACCAT CTCAGTTCAT GCGCAAAGAG AACCGGGAGG AAGAGGAAAT GGCAGAGAGA GGGGACAAGG TCGCTTCAGA GGAAGGCCAG GATCTGATAG ATCCCAAATG ATGGGTGGAC 160**

**He185/333_cDNA_0095 ATGGAGTTGA AAGTGACACT GATCGTTGCC CTTGTGGCTG CTATTACCAT CTCAGCTCAT GCACAAAGAG AACGGGGAGG AAGAGGAAAT GGCAGAGAGA GGGGACAAGG TCGCTTCGGA GGAAGGCCAG GATCTGATAG ATCCCAAAGG ATGGGTGGAC 160**

**He185/333_cDNA_0096 ATGGAGTTGA AAGTGACACT GATCGTTGCC CTTGTGGCTG CTATTACCAT CTCAGTTCAT GCGCAAAGAG AACCGGGAGG AAGAGGAAAT GGCAGAGAGA GGGGACAAGG TCGCTTCAGA GGAAGGCCAG GATCTGATAG ATCCCAAATG ATGGGTGGAC 160**

**He185/333_cDNA_0097 ATGGAGTTGA AAGTGACACT GATCGTTGCC CTTGTGGCTG CTATTACCAT CTCAGTTCAT GCGCAAAGAG AACCGGGAGG AAGAGGAAAT GGCAGAGAGA GGGGACAAGG TCGCTTCAGA GGAAGGCCAG GATCTGATAG ATCCCAAATG ATGGGTGGAC 160**

**He185/333_cDNA_0098 ATGGAGTTGA AAGTGACACT GATCGTTGCC CTTGTGGCTG CTATTACCAT CTCAGCTCAT GCACAAAGAG AACGGGGAGG AAGAGGAAAT GGCAGAGAGA GGGGACAAGG TCGCTTTGGA GGAAGGCCAG GATCTGATAG ATCCCAAATG ATGGGTGGAC 160**

**He185/333_cDNA_0099 ATGGAGTTGA AAGTGACACT GATCGTTGCC CTTGTGGCTG CTATTACCAT CTCAGTTCAT GCGCAAAGAG AACCGGGAGG AAGAGGAAAT GGCAGAGAGA GGGGACAAGG TCGCTTCAGA GGAAGGCCAG GATCTGATAG ATCCCAAATG ATGGGTGGAC 160**

**He185/333_cDNA_0100 ATGGAGTTGA AAGTGACACT GATCGTTGCC CTTGTGGCTG CTATTACCAT CTCAGTTCAT GCACAAAGAG GCCGGGGCGG AAGAGGAAAT GGCAGAGAGA GGGGACAAGG TCGCTTCGGA GGAAGGCCAG GATCC---AG ACCCCAAATG ATGGGTGGAC 157**

**He185/333_cDNA_0101 ATGGAGTTGA AAGTGACACT GATCGTTGCC CTTGTGGCTG CTATTACCAT CTCAGTTCAT GCGCAAAGAG AACCGGGAGG AAGAGGAAAT GGCAGAGAGA GGGGACAAGG TCGCTTCAGA GGAAGGCCAG GATCTGATAG ATCCCAAATG ATGGGTGGAC 160**

**He185/333_cDNA_0102 ATGGAGTTGA AAGTGACACT GATCGTTGCC CTTGTGGCTG CTATTACCAT CTCAGTTCAT GCACAAAGAG GCCGGGGCGG AAGAGGAAAT GGCAGAGAGA GGGGACAAGG TCGCTTCGGA GGAAGGCCAG GATCC---AG ACCCCAAATG ATGGGTGGAC 157**

**He185/333_cDNA_0103 ATGGAGTTGA AAGTGACACT GATCGTTGCC CTTGTGGCTG CTATTACCAT CTCAGCTCAT GCACAAAGAG AACGGGGAGG AAGAGGAAAT GGCAGAGAGA GGGGACAAGG TCGCTTCGGA GGAAGGCCAG GATCTGATAG ATCCCAAATG ATGGGTGGAC 160**

**He185/333_cDNA_0104 ATGGAGTTGA AAGTGACACT GATCGTTGCC CTTGTGGCTG CTATTACCAT CTCAGCTCAT GCACAAAGAG AACGGGGAGG AAGAGGAAAT GGCAGAGAGA GGGGACAAGG TCGCTTCGGA GGAAGGCCAG GATCTGATAG ATCCCAAATG ATGGGTGGAC 160**

**He185/333_cDNA_0105 ATGGAGTTGA AAGTGACACT GATCGTTACC CTTGTGGCTG CTATTACCAT CTCAGTTCAT GCGCAAAGAG AACCGGGAGG AAGAGGAAAT GGCAGAGAGA GGGGACAAGG TCGCTTCGGA GGAAGGCCAG GATCTGATAG ATCCCAAATG ATGGGTGGAC 160**

**He185/333_cDNA_0106 ATGGAGTTGA AAGTGACACT GATCGTTACC CTTGTGGCTG CTATTACCAT CTCAGTTCAT GCGCAAAGAG AACCGGGAGG AAGAGGAAAT GGCAGAGAGA GGGGACAAGG TCGCTTCGGA GGAAGGCCAG GATCTGATAG ATCCCAAATG ATGGGTGGAC 160**

**He185/333_cDNA_0107 ATGGAGTTGA AAGTGACACT GATCGTTACC CTTGTGGCTG CTATTACCAT CTCAGTTCAT GCGCAAAGAG AACCGGGAGG AAGAGGAAAT GGCAGAGAGA GGGGACAAGG TCGCTTCGGA GGAAGGCCAG GATCTGATAG ATCCCAAATG ATGGGTGGAC 160**

**He185/333_cDNA_0108 ATGGAGTTGA AAGTGACACT GATCGTTACC CTTGTGGCTG CTATTACCAT CTCAGTTCAT GCGCAAAGAG AACCGGGAGG AAGAGGAAAT GGCAGAGAGA GGGGACAAGG TCGCTTCGGA GGAAGGCCAG GATCTGATAG ATCCCAAATG ATGGGTGGAC 160**

**He185/333_cDNA_0109 ATGGAGTTGA AAGTGACACT GATCGTTGCC CTTGTGGCTG CTATTACCAT CTCAGTTCAT GCTCAAAGAG CACGAGGAGG AAGAGGATAT GGCAGAAAGA GGGGACAAGG TCGCTTCGGA GGAAGTCCAG GATCTGATAG ACCCCAAATG ACGGGTGGAC 160**

**He185/333_cDNA_0110 ATGGAGTTGA AAGTGACACT GATCGTTACC CTTGTGGCTG CTATTACCAT CTCAGTTCAT GCGCAAAGAG AACCGGGAGG AAGAGGAAAT GGCAGAGAGA GGGGACAAGG TCGCTTCGGA GGAAGGCCAG GATCTGATAG ATCCCAAATG ATGGGTGGAC 160**

**He185/333_cDNA_0111 ATGGAGTTGA AAGTGACACT GATCGTTACC CTTGTGGCTG CTATTACCAT CTCAGTTCAT GCGCAAAGAG AACCGGGAGG AAGAGGAAAT GGCAGAGAGA GGGGACAAGG TCGCTTCGGA GGAAGGCCAG GATCTGATAG ATCCCAAATG ATGGGTGGAC 160**

**He185/333_cDNA_0112 ATGGAGTTGA AAGTGACACT GATCGTTACC CTTGTGGCTG CTATTACCAT CTCAGTTCAT GCGCAAAGAG AACCGGGAGG AAGAGGAAAT GGCAGAGAGA GGGGACAAGG TCGCTTCGGA GGAAGGCCAG GATCTGATAG ATCCCAAATG ATGGGTGGAC 160**

6

8

11

4

5

9

12

**....|....| ....|....| ....|....| ....|....| ....|....| ....|....| ....|....| ....|....| ....|....| ....|....| ....|....| ....|....| ....|....| ....|....| ....|....| ....|....|**

7

10

**165 175 185 195 205 215 225 235 245 255 265 275 285 295 305 315**

**He185/333_cDNA_0001 CTAGGCAAGG TGGTCCGCCA ATGGGCGGAA GGAGGTTTGA T--------- ---------- ---------- ---------- ---------- ---------- ---------- ---------- ---------- ---------- ---------- ---------- 201**

**He185/333_cDNA_0002 CTAGGCAAGG TGGTCCGCCA ATGGGCGGAA GGAGGTTTGA TGGCCCTGGA CAAGGTGACC AGCAGATGGA TGGACGTGGA CCGAATGGTG GGCCAATGGG CGGTAGGAGG TTTGATGGAC CAGGATTCGG TGGCTTCAGA CTCGAAGGTG CAGGGAGACC 320**

**He185/333_cDNA_0003 CTAGGCAAGG TGGTCCGCCA ATGGGCGGAA GGAGGTTTGA TGGCCCTGGA CAAGGTGACC AGCGGATGGA TGGACGTGGA CCGAATGGTG GGCCAATGGG CGGTAGGAGG TTTGATGGAC CAGGATTCGG TGGCTTCAGA CTCGAAGGTG CAGGGAGACC 320**

**He185/333_cDNA_0004 CTAGGCAAGG TGGTCCGCCA ATGGGCGGAA GGAGGTTTGA TGGCCCTGGA CAAGGTGACC AGCAGATGGA TGGACGTGGA CCGAATGGTG GGCCAATGGG CGGTAGGAGG TTTGATGGAC CAGGATTCGG TGGCTTCAGA CTCGAAGGTG CAGGGAGACC 320**

**He185/333_cDNA_0005 CTAGGCAAGG TGGTCCGCCA ATGGGCGGAA GGAGGTTTGA TGGCCCTGGA CAAGGTGACC AGCAGATGGA TGGACGTGGA CCGAATGGTG GGCCAATGGG CGGTAGGAGG TTTGATGGAC CAGGATTCGG TGGCTTCAGA CTCGAAGGTG CAGGGAGACC 320**

**He185/333_cDNA_0006 CTAGGCAAGG TGGTCCGCCA ATGGGCGGAA GGAGGTTTGA TGGCCCTGGA CAAGGTGACC AGCAGATGGA T--------- ---------- ---------- ---------- ---------- ---------- ---------- ---------- ---------- 231**

**He185/333_cDNA_0007 CTAGGCAAGG TGGTCCGCCA ATGGGCGGAA GGAGGTTTGA TGGCCCTGGA CAAGGTGACC AGCAGATGGA TGGACGTGGA CCGAATGGTG GGCCAATGGG CGGTAGGAGG TTTGATGGAC CAGGATTCGG TGGCTTCAGG CTCGAAGGTG CAGGGAGATC 320**

**He185/333_cDNA_0008 CTAGGCAAGG TGGTCCGCCA ATGGGCGGAA GGAGGTTTGA TGGCCCTGGA CAAGGTGACC GGCAGATGGA TGGACGTGGA CCGAATGGTG GGCCAATGGG CGGTAGGAGG TTTGATGGAC CAGGATTCGG TGGCTTCAGA CTCGAAGGTG CAGGGAGACC 320**

**He185/333_cDNA_0009 CTAGGCAAGG TGGTCCGCCA ATGGGCGGAA GGAGGTTTGA TGGCCCTGGA CAAGGTGACC AGCAGATGGA TGGACGTGGA CCGAATGGTG GACCAATGGG CGGTAGGAGG TTTGATGGAC CAGGATTCGG TGGCTTCAGA CTCGAAGGTG CAGGGAGACC 320**

**He185/333_cDNA_0010 CTAGGCAAGG TGGTCCGCCA ATGGGCGGAA GGAGGTTTGA TGGCCCTGGA CAAGGTGACC AGCAGATGGA TGGACGTGGA CCGAATGGTG GGCCAATGGG CGGTAGGAGG TTTGATGGAC CAGGATTCGG TGGCTTCAGA CTCGAAGGTG CAGGGAGACC 320**

**He185/333_cDNA_0011 CTAGGCAAGG TGGTCCGCCA ATGGGCGGAA GGAGGTTTGA TGGCCCTGGA CAAGGTGACC AGCAGATGGA TGGACGTGGA CCGAATGGTG GGCCAATGGG CGGTAGGAGG TTTGATGGAC CAGGATTCGG TGGCTTCAGA CTCGAAGGTG CAGGGAGACC 320**

**He185/333_cDNA_0012 CTAGGCAAGG TGGTCCACCA ATGGGCGGAA GGAGGTTTGA TGGCCCTGGA CAAGGTGACC AACAGATGGA TGGACGTGGA CCGAATGGTG GGCCAATGGG CGGTAGAAGG TTTGATGGAC CAGGATTCGG TGGCTTCAGA CCCGAAGGTG CAGGGAGACC 320**

**He185/333_cDNA_0013 CTAGGCAAGG TGGTCCGCCA ATGGGCGGAA GGAGGTTTGA TGGCCCTGGA CAAGGTGACC AGCAGATGGA TGGACGTGGA CCGAATGGTG GGCCAATGGG CGGTAGGAGG TTTGATGGAC CAGGATTCGG TGGCTTCAGA CTCGAAGGTG CAGGGAGACC 320**

**He185/333_cDNA_0014 CTAGGCAAGG TGGTCCACCA ATGGGCGGAA GGAGGTTTGA TGGCCCTGGA CAAGGTGACC AACAGATGGA TGGACGTGGA CCGAATGGTG GGCCAATGGG CGGTAGGAGG TTTGATGGAC CAGGATTCGG TGGCTTCAGA CCCGAAGGTG CAGGGAGACC 320**

**He185/333_cDNA_0015 CCAGGCAAGG TGGTCCACCA ATGGGCGGAA GGAGGTTTGA TGGCCCTGGA CAAGGTGACC AACAGATGGA TGGACGTGGA CCGAATGGTG GGCCAATGGG CGGTAGGAGG TTTGATGGAC CAGGATTCGG TGGCTTCAGA CCCGAAGGTG CAGGGAGACC 320**

**He185/333_cDNA_0016 CTAGGCAAGG TGGTCCACCA ATGGGCGGAA GGAGGTGTGA TGGCCCTGGA CAAGGTGACC AACAGATGGA TGGACGTGGA CCGAATGGTG GGCCAATGGG CGGTAGGAGG TTTGATGGAC CAGGATTCGG TGGCTTCAGA CCCGAAGGTG CAGGGAGACC 320**

**He185/333_cDNA_0017 CTAGGCAAGG TGGTCCGCCA ATGGGCGGAA GGAGGTTTGA TGGCCCTGGA CAAGGTGACC AGCAGATGGA TGGACGTGGA CCGAATGGTG GGCCAATGGG CGGTAGGAGG TTTGATGGAC CAGGATTCGG TGGCTTCAGA CTCGAAGGTG CAGGGAGACC 320**

**He185/333_cDNA_0018 CTAGGCAAGG TGGTCCGCCA ATGGGCGGAA GGAGGTTTGA TGGCCCTGGA CAAGGTGACC AGCAGATGGA TGGACGTGGA CCGAATGGTG GGCCAATGGG CGGTAGGAGG TTTGATGGAC CAGGATTCAG TGGCTTCAGA CTCGAAGGTG CAGGGAGACC 320**

**He185/333_cDNA_0019 CTAGGCAAGG TGGTCCGCCA ATGGGCGGAA GGAGGTTTGA TGGCCCTGGA CAAGGTGACC AGCAGATGGA TGGACGTGGA CCGAATGGTG GGCCAATGGG CGGTAGGAGG TTTGATGGAC CAGGATTCGG TGGCTTCAGA CTCGAAGGCG CAGGGAGACC 320**

**He185/333_cDNA_0020 CTAGGCAAGG TGGTCCGCCA ATGGGCGGAA GGAGGTTTGA TGGCCCTGGA CAAGGTGACC AGCAGATGGA TGGACGTGGA CCGAATGGTG GGCCAATGGG CGGTAGGAGG TTTGATGGAC CAGGATTCGG TGGCTTCAGA CTCGAAGGTG CAGGGAGACC 278**

**He185/333_cDNA_0021 CTAGGCAAGG TGGTCCGCCA ATGGGCGGAA GGAGGTTTGA TGGCCCTGGA CAAGGTGACC AGCAGATGGA TGGACGTGGA CCGAATGGTG GGCCAATGGG CGGTAGGAGG TTTGATGGAC CAGGATTCGG TGGCTTCAGA CTCGAAGGTG CAGGGAGACC 320**

**He185/333_cDNA_0022 ---------- ---------- ---------- ---------- ---------- ---------- ---------- ---------- ---------- ---------- ---------- ---------- ---------- ---------- ---------- ---------- 102**

**He185/333_cDNA_0023 ATAGGCAAGG TGGTCCACCA ATGGGTGGAA GGAGGTTTGA TGGCAATGGA CAAGGTGACC AACAGATGGC TGGACGTGAA CCGAATGGCC GGCCAGTGGG CAGTAGAAGA TTTGATGGAC CAGGATTCGG TGGCTTCAGA CCCGAAGGTG CCGGGAGACC 317**

**He185/333_cDNA_0024 CTAGGCAAGG TGGTCCACCA ATGGGCGGAA GGAGGTTTGA TGGCCCTGGA CAAGGTGACC AACAGATGGA TGGACGTGGA CCGAATGGTG GGCCAATGGG CGGTAGGAGG TTTGATGGAC CAGGATCCGG TGGCTTCAGA CCCGAAGGTG CAGGGAGACC 320**

**He185/333_cDNA_0025 ATAGGCAAGG TGGTCCACCA ATGGGTGGAA GGAGGTTTGA TGGCAATGGA CAAGGTGACC AACAGATGGC TGGACGTGAA CCGAATGGCC GGCCAGTGGG CAGTAGAAAA TTTGATGGAC CAGGATTCGG TGGCTTCAGA CCCGAAGGTG CCGGGAGACC 317**

**He185/333_cDNA_0026 CTAGGCAAGG TGGTCCACCA ATGGGCGGAA GGAGGTTTGA TGGCCCTGGA CAAGGTGACC AACAGATGGA TGGACGTGGA CCGAATGGTG GGCCAATGGG CGGTAGGAGG TTTGATGGAC CAGGATTCGG TGGCTTCAGA CCCGAAGGTG CAGGGAGACC 320**

**He185/333_cDNA_0027 ATAGGCAAGG TGGTCCACCA ATGGGTGGAA GGAGGTTTGA TGGCAATGGA CAAGGTGACC AACAGATGGC TGGACGTGAA CCGAATGGCC GGCCAGTGGG CAGTAGAAAA TTTGATGGAC CAGGATTCGG TGGCTTCAGA CCCGAAGGTG CCGGGAGACC 317**

**He185/333_cDNA_0028 CTAGGCAAGG TGGTCCACCA ATGGGCGGAA GGAGGTTT-- ---------- ---------- ---------- ---------- ---------- ---------- ---------- ---------- ---------- ---------- ---GAAGGTG CAGGGAGACC 215**

**He185/333_cDNA_0029 ATAGGCAAGG TGGTCCACCA ATGGGTGGAA GGAGGTTTGA TGGCAATGGA CAAGGTGACC AACAGATGGC TGGACGTGAA CCGAATGGCC GGCCAGTGGG CAGTAGAAAA TTTGATGGAC CAGGATTCGG TGGCTTCAGA CCCGAAGGTG CCGGGAGACC 317**

**He185/333_cDNA_0030 CTAGGCAAGG TGGTCCACCA ATGGGCGGAA GGAGGTTTGA TGGCCCTGGA CAAGGTGACC AACAGATGGA TGGACGTGGA CCGAATGGTG GGCCAATGGG CGGTAGGAGG TTTGATGGAC CAGGATTCGG TGGCTTCAGA CCCGAAGGTG CAGGGAGACT 320**

**He185/333_cDNA_0031 ATAGGCAAGG TGGTCCACCA ATGGGTGGAA GGAGGTTTGA TGGCAATGGA CAAGGTGACC AACAGATGGC TGGACGTGAA CCGAATGGCC GGCCAGTGGG CAGTAGAAAA TTTGATGGAC CAGGATTCGG TGGCTTCAGA CCCGAAGGTG CCGGGAGACC 317**

**He185/333_cDNA_0032 CTAGGCAAGG TGGTCCACCA ATGGGCGGAA GGAGGTTT-- ---------- ---------- ---------- ---------- ---------- ---------- ---------- ---------- ---------- ---------- ---GAAGGTG CAGGGAGACC 215**

**He185/333_cDNA_0033 ATAGGCAAGG TGGTCCACCA ATGGGTGGAA GGAGGTTTGA TGGCAATGGA CAAGGTGACC AACAGATGGC TGGACGTGAA CCGAATGGCC GGCCAGTGGG CAGTAGAAAA TTTGATGGAC CAGGATTCGG TGGCTTCAGA CCCGAAGGTG CCGGGAGACC 317**

**He185/333_cDNA_0034 ATAGGCAAGG TGGTCCACCA ATGGGTGGAA GGAGGTTTGA TGGCAATGGA CAAGGTGACC AACAGATGGC TGGACGTGAA CCGAATGGCC GGCCAGTGGG CAGTAGAAAA TTTGATGGAC CAGGATTCGG TGGCTTCAGA CCCGAAGGTG CCGGGAGACC 317**

**He185/333_cDNA_0035 ATAGGCAAGG TGGTCCACCA ATGGGTGGAA GGAGGTTTGA TGGCCCTGGA CAAGGTGACC AACAGATGGC TGGACGTGAA CCAAATGGCC GGCCAGTGGG CAGTAGAAAA TTTGATGGAC CAGGATTCGG TGGCTTCAGA CCCGAAGGTG CCGGGAGACC 320**

**He185/333_cDNA_0036 CTAGGCAAGG TGGTGCACCA ATGGGCGGAA GGAGGTTTGA TGGTCCTGGA CAAGGTGATC AGCAGATGGA CGGACGTGGA CCGAATGGTG GGCCAATGGG CGGTAGGAGG TTTGATGGAC CAGGATTCGG TGGCTTCAGA CCCGAAGGTG CCGGGAGACC 320**

**He185/333_cDNA_0037 CTAGGCAAGG TGGTCCACCA ATGGGCGGAA GGAGGTTTGA TGGCCCTGGA CAAGGTGACC AACAGATGGA TGGACGTGGA CCGAATGGTG GGCCAATGGG CGGTAGGAGG TTTGATGGAC CAGGATTCGG TGGCTTCAGA CCCGAAGGTG CAGGGAGACC 320**

**He185/333_cDNA_0038 CTAGGCAAGG TGGTCCGCCA ATGGGCGGAA GGAGGTTTGA TGGCCCTGGA CAAGGTGACC AGCAGATGGA TGGACGTGGA CCGAATGGTG GGCCAATGGG CGGTAGGAGG TTTGATGGAC CAGGATTCGG TGGCTTCAGA CTCGAAGGTG CAGGGAGACC 320**

**He185/333_cDNA_0039 CTAGGCAAGG TGGTCCGCCA ATGGGCGGAA GGAGGTTTGA TGGCCCTGGA CAAGGTGACC AGCAGATGGA TGGACGTGGA CCGAATGGTG GGCCAATGGG CGGTAGGAGG TTTGATGGAC CAGGATTCGG TGGCTTCAGA CTCGAAGGTG CAGGGAGACC 320**

**He185/333_cDNA_0040 CTAGGCAAGG TGGTCCACCA ATGGGCGGAA GGAGGTTTGA TGGCCCTGGA CAAGGTGACC AGCAGATGGA TGGACGTGGA CCGAATGGTG GGCCAATGGG CGGTAGGAGG TTTGATGGAC CAGGATTCGG TGGCTTCAGA CCCGAAGGTG CAGGGAGACC 320**

**He185/333_cDNA_0041 CTAGGCAAGG TGGTCCGCCA ATGGGCGGAA GGAGGTTTGA TGGCCCTGGA CAAGGTGACC AGCAGATGGA TGGACGTGGA CCGAATGGTG GGCCAATGGG CGGTAGGAGG TTTGATGGAC CAGGATTCGG TGGCTTCAGA CTCGAAGGTG CAGGGAGACC 320**

**He185/333_cDNA_0042 CTAGGCAAGG TGGTCCGCCA ATGGGCGGAA GGAGGTTTGA TGGCCCTGGA CAAGGTGACC AGCAGATGGA TGGACGTGGA CCGAATGGTG GGCCAATGGG CGGTAGGAGG TTTGATGGAC CAGGATTCGG TGGCTTCAGA CTCGAAGGTG CAGGGAGACC 320**

**He185/333_cDNA_0043 ATAGGCAAGG TGGTCCACCA ATGGGTGGAA GGAGGTTTGA TGGCAATGGA CAAGGTGACC AACAGATGGC TGGACGTGAA CCGAATGGCC GGCCAGTGGG CAGTAGAAAA TTTGATGGAC CAGGATTCGG TGGCTTCAGA CCCGAAGGTG CCGGGAGACC 317**

**He185/333_cDNA_0044 CTAGGCAAGG TGGTCCGCCA ATGGGCGGAA GGAGGTTTGA TGGCCCTGGA CAAGGTGACC AGCAGATGGA TGGACGTGGA CCGAATGGTG GGCCAATGGG CGGTAGGAGG TTTGATGGAC CAGGATTCGG TGGCTTCAGA CTCGAAGGTG CAGGGAGACC 320**

**He185/333_cDNA_0045 CTAGGCAAGG TGGTCCACCA ATGGGCGGAA GGAGGTTTGA TGGCCCTGGA CAAGGTGACC AGCAGATGGA TGGACGTGGA CCGAATGGTG GGCCAATGGG CGGTAGGAGG TTTGATGGAC CAGGATTCGG TGGCTTCAGA CCCGAAGGTG CAGGGAGACC 320**

**He185/333_cDNA_0046 CTAGGCAAGG TGGTCCACCA ATGGGCGGAA GGAGGTTTGA TGGCCCTGGA CAAGGCGACC AGCAGATGGA TGGACGTGGA CCGAATGGTG GGCCAATGGG CGGTAGGAGG TTTGATGGAC CAGGATTCGG TGGCTTCAGA CCCGAAGGTG CAGGGAGACC 320**

**He185/333_cDNA_0047 CTAGGCAAGG TGGTCCACCA ATGGGCGGAA GGAGGTTTGA TGGCCCTGGA CAAGGTGACC AACAGATGGA TGGACGTGGA CCGAATGGTG GGCCAATGGG CGGTAGGAGG TTTGATGGAC CAGGATTCGG TGGCTTCAGA CCCGAAGGTG CAGGGAGACC 320**

**He185/333_cDNA_0048 CTAGGCAAGG TGGTCCGCCA ATGGGCGGAA GGAGGTTTGA TGGCCCTGGA CAAGGTGACC AGCTGATGGA TGGACGTGGA CCGAATGGTG GGCCAATGGG CGGTAGGAGG TTTGATGGAC CAGGATTCGG TGGCTTCAGA CTCGAAGGTG CAGGGAGACC 320**

**He185/333_cDNA_0049 CTAGGCAAGG TGGTCCGCCA ATGGGCGGAA GGAGGTTTGA TGGCCCTGGA CAAGGTGACC AGCAGATGGA TGGACGTGGA CCGAATGGTG GGCCAATGGG CGGTAGGAGG TTTGATGGAC CAGGATTCGG TGGCTTCAGA CTCGAAGGTG CAGGGAGACC 320**

**He185/333_cDNA_0050 CTAGGCAAGG TGGTCCGCCA ATGGGCGGAA GGAGGTTTGA TGGCCCTGGA CAAGGTGACC AGCAGATGGA TGGACGTGGA CCGAATGGTG GGCCAATGGG CGGTAGGAGG TTTGATGGAC CAGGATTCGG TGGCTTCAGA CTCGAAGGTG CAGGGAGACC 320**

**He185/333_cDNA_0051 CTAGGCAAGG TGGTCCACCA ATGGGCGGAA GGAGGTTTGA TGGCCCTGGA CAAGGTGACC AGCAGATGGA TGGACGTGGA CCGAATGGTG GGCCAATGGG CGGTAGGAGG TTTGATGGAC CAGGATTCGG TGGCTTCAGA CCCGAAGGTG CAGGGAGACC 320**

**He185/333_cDNA_0052 CTAGGCAAGG TGGTCCGCCA ATGGGCGGTA GGAGGTTT-- ---------- ---------- ---------- ---------- ---------- ---------- ---------- ---GATGGAC CAGGATTCGG TGGCTTCAGA CTCGAAGGTG CAGGGAGACC 245**

**He185/333_cDNA_0053 CTAGGCAAGG TGGTCCACCA ATGGGCGGAA GGAGGTTTGA TGGCCCTGGA CAAGGTGACC AGCAGATGGA TGGACGTGGA CCGAATGGTG GGCCAATGGG CGGTAGGAGG TTTGATGGAC CAGGATTCGG TGGCTTCAGA CCCGAAGGTG CAGGGAGACC 320**

**He185/333_cDNA_0054 ATAGGCAAGG TGGTCCACCA ATGGGTGGAA GGAGGTTTGA TGGCAATGGA CAAGGTGACC AACAGATGGC TGGACGTGAA CCGAATGGCC GGCCAGTGGG CAGTAGAAAA TTTGATGGAC CAGGATTCGG TGGCTTCAGA CCCGAAGGTG CCGGGAGACC 317**

**He185/333_cDNA_0055 CTAGGCAAGG TGGTCCACCA ATGGGCGGAA GGAGGTTTGA TGGCCCTGGA CAAGGTGACC AACAGATGGA TGGACGTGGA CCGAATGGTG GGCCAATGGG CGGTAGGAGG TTTGATGGAC CAGGATTCGG TGGCTTCAGA CCCGAAGGTG CAGGGAGACC 320**

**He185/333_cDNA_0056 CTAGGCAAGG TGGTCCACCA ATGGGCGGAA GGAGGTTTGA TGGCCCTGGA CAAGGTGACC AACAGATGGA TGGACGTGGA CCGAATGGTG GGCCAATGGG CGGTAGGAGG TTTGATGGAC CAGGATTCGG TGGCTTCAGA CCCGAAGGTG CAGGGAGACC 320**

**He185/333_cDNA_0057 CTAGGCAAGG TGGTGCACCA ATGGGCGGAA GGAGGTTTGA TGGCCCTGGA CAAGGTGATC AGCAGATGGA CGGACGTGGA CCGAATGGTG GGCCAATGGG CGGTAGGAGG TTTGATGGAC CAGGATTCGG TGGCTTCAGA CCCGAAGGTG CCGGGAGACC 320**

**He185/333_cDNA_0058 CTAGGCAAGG TGGTGCACCA ATGGGCGGAA GGAGGTTTGA TGGCCCTGGA CAAGGTGATC AGCAGATGGA CGGACGTGGA CCGAATGGTG GGCCAATGGG CGGTAGGAGG TTTGATGGAC CAGGATTCGG TGGCTTCAGA CCC------- ---------- 303**

**He185/333_cDNA_0059 CTAGGCAAGG TGGTGCACCA ATGGGCGGAA GGAGGTTTGA TGGCCCTGGA CAAGGTGATC AGCAGATGGA CGGACGTGGA CCGAATGGTG GGCCAATGGG CGGTAGGAGG TTTGATGGAC CAGGATTCGG TGGCTTCAGA CCCGAAGGTG CCGGGAGACC 320**

**He185/333_cDNA_0060 ATAGGCAAGG TGGTCCACCA ATGGGTGGAA GGAGGTTTGA TGGCCCTGGA CAAGGTGACC AACAGATGGC TGGACGTGAA CCAAATGGCC GGCCAGTGGG CAGTAGAAAA TTTGATGGAC CAGGATTCGG TGGCTTCAGA CCCGAAGGTG CCGGGAGACC 320**

**He185/333_cDNA_0061 CTAGGCAAGG TGGTGCACCA ATGGGCGGAA GGAGGTTTGA TGGCCCTGGA CAAGGTGATC AGCAGATGGA CGGACGTGGA CCGAATGGTG GGCCAATGGG CGGTAGGAGG TTTGATGGAC CAGGATTCGG TGGCTTCAGA CCCGAAGGTG CCGGGAGACC 320**

**He185/333_cDNA_0062 CTAGGCAAGG TGGTGCACCA ATGGGCGGAA GGAGGTTTGA TGGCCCTGGA CAAGGT---- ---------- ---------- ---------- ---------- ---------- ---------- ---------- ---------- ---------- ---------- 216**

**He185/333_cDNA_0063 CTAGGCAAGG TGGTGCACCA ATGGGCGGAA GGAGGTTTGA TGGCCCTGGA CAAGGTGATC AGCAGATGGA CGGACGTGGA CCGAATGGTG GGCCAATGGG CGGTAGGAGG TTTGATGGAC CAGGATTCGG TGGCTTCAGA CCCGAAGGTG CCGGGAGACC 320**

**He185/333_cDNA_0064 CTAGGCAAGG TGGTGCACCA ATGGGCGGAA GGAGGTTTGA TGGCCCTGGA CAAGGTGATC AGCAGATGGA CGGACGTGGA CCGAATGGTG GGCCAATGGG CGGTAGGAGG TTTGATGGAC CAGGATTCGG TGGCTTCAGA CCCGAAGGTG CCGGGAGACC 320**

**He185/333_cDNA_0065 CTAGGCAAGG TGGTGCACCA ATGGGCGGTA GGAGGTTT-- ---------- ---------- ---------- ---------- ---------- ---------- ---------- ---GATGGAC CAGGATTCGG TGGCTTCAGA CCCGAGGGTG CCGGGAGACC 245**

**He185/333_cDNA_0066 CTAGGCAAGG TGGTGCACCA ATGGGCGGAA GGAGGTTTGA TGGCCCTGGA CAAGGTGATC AGCAGATGGA CGGACGTGGA CCGAATGGTG GGCCAATGGG CGGTAGGAGG TTTGATGGAC CAGGATTCGG TGGCTTCAGA CCCGAAGGTG CCGGGAGACC 320**

**He185/333_cDNA_0067 CTAGGCAAGG TGGTGCACCA ATGGGCGGTA GGAGGTTT-- ---------- ---------- ---------- ---------- ---------- ---------- ---------- ---GATGGAC CAGGATTCGG TGGCTTCAGA CCCGAAGGTG CCGGGAGACC 245**

**....|....| ....|....| ....|....| ....|....| ....|....| ....|....| ....|....| ....|....| ....|....| ....|....| ....|....| ....|....| ....|....| ....|....| ....|....| ....|....|**

4

6

7

8

10

11

5

9

12

**165 175 185 195 205 215 225 235 245 255 265 275 285 295 305 315**

**He185/333_cDNA_0068 CTAGGCAAGG TGGTCCACCA ATGGGCGGAA GGAGGTTTGA TGGCCCTGGA CAAGGTGACC AGCAGATGGA TGGACGTGGA CCGAATGGTG GGCCAATGGG CGGTAGGAGG TTTGATGGAC CAGGATTCGG TGGCTTCAGA CCCGAAGGTG CAGGGAGACC 320**

**He185/333_cDNA_0069 CTAGGCAAGG TGGTCCGCCA ATGGGCGGAA GGAGGTTTGA TGGCCCTGGA CAAGGTGACC AGCAGATGGA TGGACGTGGA CCGAATGGTG GGCCAATGGG CGGTAGGAGG TTTGATGGAC CAGGATTCGG TGGCTTCAGA CTCGAAGGTG CAGGGAGACC 320**

**He185/333_cDNA_0070 CTAGGCAAGG TGGTCCGCCA ATGGGCGGAA GGAGGTTTGA TGGCCCTGGA CAAGGTGACC AGCAGATGGA TGGACGTGGA CCGAATGGTG GGCCAATGGG CGGTAGGAGG TTTGATGGAC CAGGATTCGG TGGCTTCAGA CTCGAAGGTG CAGGGAGACC 320**

**He185/333_cDNA_0071 CTAGGCAAGG TGGTCCGCCA ATGGGCGGAA GGAGGTTTGA TGGCCCTGGA CAAGGTGACC AGCAGATGGA TGGACGTGGA CCGAATGGTG GGCCAATGGG CGGTAGGAGG TTTGATGGAC CAGGATTCGG TGGCTTCAGA CTCGAAGGTG CAGGGAGACC 320**

**He185/333_cDNA_0072 CTAGGCAAGG TGGTCCGCCA ATGGGCGGAA GGAGGTTTGA TGGCCCTGGA CAAGGTGACC AGCAGATGGA TGGACGTGGA CCGAATGGTG GGCCGATAGG CGGTAGGAGG TTTGATGGAC CAGGATTCGG TGGCTTCAGA CTCGAAGGTG CAGGGAGACC 320**

**He185/333_cDNA_0073 CTAGGCAAGG TGGTCCGCCA ATGGGCGGAA GGAGGTTTGA TGGCCCTGGA CAAGGTGACC AGCGGATGGA TGGACGTGGA CCGAATGGTG GGCCGATGGG CGGTAGGAGG TTTGATGGAC CAGGATTCGA TGGCTTCAGA CTCGAAGGTG CAGGGAGACC 320**

**He185/333_cDNA_0074 CTAGGCAAGG TGGTCCGCCA ATGGGCGGAA GGAGGTTTGA TGGCCCTGGA CAAGGTGACC AGCAGATGGA TGGACGTGGA CCGAATGGTG GGCCAATGGG CGGTAGGGGG TTTGATGGAC CAGGATTCGG TGGCTTCAGA CTCGAAGGTG CAGGGAGACC 320**

**He185/333_cDNA_0075 CTAGGCAAGG TGGTGCACCA ATGGGCGGAA GGAGGTTTGA TGGCCCTGGA CAAGGTGATC AGCAGATGGA CGGACGTGGA CCGAATGGTG GGCCAATGGG CGGTAGGAGG TTTGATGGAC CAGGATTCGG TGGCTTCAGA CCCGAAGGTG CCGGGAGACC 320**

**He185/333_cDNA_0076 CTAGGCAAGG TGGTCCGCCA ATGGGCGGAA GGAGGTTTGA TGGCCCTGGA CAAGGTGACC AGCAGATGGA TGGACGTGGA CCGAATGGTG GGCCAATGGG CGGTGGGAGG TTTGATGGAC CAGGATTCGG TGGCTTCAGA CTCGAAGGTG CAGGGAGACC 320**

**He185/333_cDNA_0077 CTAGGCAAGG TGGTCCACCA ATGGGCGGAA GGAGGTTTGA TGGCCCTGGA CAAGGTGACC AACAGATGGA TGGACGTGGA CCGAATGGTG GGCCAATGGG CGGTAGGAGG TTTGGTGGAC CAGGATTCGG TGGCTTCAGA CCCGAAGGTG CAGGGAGACC 320**

**He185/333_cDNA_0078 CTAGGCAAGG TGGTCCGCCA ATGGGCGGAA GGAGGTTTGA TGGCCCTGGA CAAGGTGACC AGCAGATGGA TGGACGTGGA CCGAATGGTG GGCCAATGGG CGGTAGGAGG TTTGATGGAC CAGGATTCGG TGGCTTCAGA CTCGAAGGTG CAGGGAGACC 320**

**He185/333_cDNA_0079 CTAGGCAAGG TGGTCCGCCA ATGGGCGGAA GGAGGTTTGA TGGCCCTGGA CAAGGTGACC AGCAGATGGA TGGACGTGGA CCGAATGGTG GGCCAATGGG CGGTAGGAGG TTTGATGGAC CAGGATTCGG TGGCTTCAGA CTCGAAGGTG CAGGGAGACC 320**

**He185/333_cDNA_0080 CTAGGCAAGG TGGTCCGCCA ATGGGCGGAA GGAGGTTTGA TGGCCCTGGA CAAGGTGACC AGCAGATGGA TGGACGCGGA CCGAATGGTG GGCCAATGGG CGGTAGGAGG TTTGATGGAC CAGGATTCGG TGGCTTCAGA CTCGAAGGTG CAGGGAGACC 320**

**He185/333_cDNA_0081 ATAGGCAAGG TGGTCCACCA ATGGGTGGAA GGAGGTTTGA TGGCAATGGA CAAGGTGACC AACAGATGGC TGGACGTGAA CCGAATGGCC GGCCAGTGGG CAGTAGAAAA TTTGATGGAC CAGGATTCGG TGGCTTCAGA CCCGAAGGTG CCGGGAGACC 317**

**He185/333_cDNA_0082 CTAGGCAAGG TGGTCCACCA ATGGGCGGAA GGAGGTTTGA TGGCCCTGGA CAAGGTGACC AGCAGATGGA TGGACGTGGA CCGAATGGTG GGCCAATGGG CGGTAGGAGG TTTGATGGAC CAGGATTCGG TGGCTTCAGA CCCGAAGGTG CAGGGAGACC 320**

**He185/333_cDNA_0083 ATAGGCAAGG TGGTCCACCA ATGGGTGGAA GGAGGTTTGA TGGCAATGGA CAAGGTGACC AACAGATGGC TGGACGTGAA CCGAATGGCC GGCCAGTGGG CAGTAGAAAA TTTGATGGAC CAGGATTCGG TGGCTTCAGA CCCGAAGGTG CCGGGAGACC 317**

**He185/333_cDNA_0084 CTAGGCAAGG TGGTCCACCA ATGGGCGGAA GGAGGTTTGA TGGCCCTGGA CAAGGTGACC AGCAGATGGA TGGACGTGGA CCGAATGGTG GGCCAATGGG CGGTAGGAGG TTTGATGGAC CAGGATTCGG TGGCTTCAGA CCCGAAGGTG CAGGGAGACC 320**

**He185/333_cDNA_0085 CTAGGCAAGG TGGTCCACCA ATGGGCGGAA GGAGGTTTGA TGGCCCTGGA CAAGGTGACC AGCAGATGGA TGGACGTGGA CCGAATGGTG GGCCAATGGG CGGTAGGAGG TTTGATGGAC CAGGATTCGG TGGCTTCAGA CCCGAAGGTG CAGGGAGACC 320**

**He185/333_cDNA_0086 CTAGGCAAGG TGGTCCACCA ATGGGCGGAA GGAGGTTTGA TGGCCCTGGA CAAGGTGACC AGCAGATGGA TGGACGTGGA CCGAATGGTG GGCCAATGGG CGGTAGGAGG TTTGATGGAC CAGGATTCGG TGGCTTCAGA CCCGAAGGTG CAGGGAGACC 320**

**He185/333_cDNA_0087 CTAGGCAAGG TGGTCCACCA ATGGGCGGAA GGAGGTTTGA TGGCCCTGGA CAAGGTGACC AGCAGATGGA TGGACGTGGA CCGAATGGTG GGCCAATGGG CGGTAGGAGG TTTGATGGAC CAGGATTCGG TGGCTTCAGA CCCGAAGGTG CAGGGAGACC 320**

**He185/333_cDNA_0088 CTAGGCAAGG TGGTGCACCA ATGGGCGGAA GGAGGTTTGA TGGCCCTGGA CAAGGTGATC AGCAGATGGA CGGACGTGGA CCGAATGGTG GGCCAATGGG CGGTAGGAGG TTTGATGGAC CAGGATTCGG TGGCTTCAGA CCCGAAGGTG CCGGGAGACC 320**

**He185/333_cDNA_0089 CTAGGCAAGG TGGTGCACCA ATGGGCGGAA GGAGGTTTGA TGGCCCTGGA CAAGGTGATC AGCAGATGGA CGGACGTGGA CCGAATGGTG GGCCAATGGG CGGTAGGAGG TTTGATGGAC CAGGATTCGG TGGCTTCAGA CCCGAAGGTG CCGGGAGACC 320**

**He185/333_cDNA_0090 CTAGGCAAGG TGGTCCACCA ATGGGCGGAA GGAGGTTTGA TGGCCCTGGA CAAGGTGACC AGCAGATGGA TGGACGTGGA CCGAATGGTG GGCCAATGGG CGGTAGGAGG TTTGATGGAC CAGGATTCGG TGGCTTCAGA CCCGAAGGTG CAGGGAGACC 320**

**He185/333_cDNA_0091 CTAGGCAAGG TGGTCCACCA ATGGGCGGAA GGAGGTTTGA TGGCCCTGGA CAAGGTGACC AGCAGATGGA TGGACGTGGA CCGAATGGTG GGCCAATGGG CGGTAGGAGG TTTGATGTAC CAGGATTCGG TGGCTTCAGA CCCGAAGGTG CAGGGAGACC 320**

**He185/333_cDNA_0092 CTAGGCAAGG TGGTCCACCA ATGGGCGGAA GGAGGTTTGA TGGCCCTGGA CAAGGTGACC AGCAGATGGA TGGACGTGGA CCGAATGGTG GGCCAATGGG CGGTAGGAGG TTTGATGGAC CAGGATTCGG TGGCTTCAGA CCCGAAGGTG CAGGGAGACC 320**

**He185/333_cDNA_0093 CTAGGCAAGG TGGTCCACCA ATGGGCGGAA GGAGGTTTGA TGGCCCTGGA CAAGGTGACC AGCAGATGGA C--------- ---------- ---------- ---------- ---------- ---------- ---------- ---------- ---------- 231**

**He185/333_cDNA_0094 CTAGGCAAGG TGGTCCACCA ATGGGCGGAA GGAGGTTTGA TGGCCCTGGA CAAGGTGACC AGCAGATGGA CGGACGTGGA CCGAATGGTG GGCCAATGGG CGGTAGGAGG TTTGATGGAC CAGGATTCGG TGGCTTCAGA CCCGAAGGTG CCGGGAGACC 320**

**He185/333_cDNA_0095 CTAGGCAAGG TGGTGCACCA ATGGGCGGAA GGAGGTTTGA TGGCCCTGGA CAAGGTGATC AGCAGATGGA CGGACGTGGA CCGAATGGTG GGCCAATGGG CGGTAGGAGG TTTGATGGAC CAGGATTCGG TGGCTTCAGA CCCGAAGGTG CCGGGAGACC 320**

**He185/333_cDNA_0096 CTAGGCAAGG TGGTCCACCA ATGGGCGGAA GGAGGTTTGA TGGCCCTGGA CAAGGTGACC AGCAGATGGA TGGACGTGGA CCGAATGGTG GGCCAATGGG CGGTAGGAGG TTTGATGGAC CAGGATTCGG TGGCTTCAGA CCCGAAGGTG CAGGGAGACC 320**

**He185/333_cDNA_0097 CTAGGCAAGG TGGTCCACCA ATGGGCGGAA GGAGGTTTGA TGGCCCTGGA CAAGGTGACC AGCAGATGGA TGGACGTGGA CCGAATGGTG GGCCAATGGG CGGTAGGAGG TTTGATGGAC CAGGATTCGG TGGCTTCAGA CCCGAAGGTG CAGGGAGACC 320**

**He185/333_cDNA_0098 CTAGGCAAGG TGGTGCACCA ATGGGCGGAA GGAGGTTTGA TGGCCCTGGA CAAGGTGATC AGCAGATGGA CGGACGTGGA CCGAATGGTG GGCCAATGGG CGGTAGGAGG TTTGATGGAC CAGGATTCGG TGGCTTCAGA CCCGAAGGTG CCGGGAGACC 320**

**He185/333_cDNA_0099 CTAGGCAAGG TGGTCCACCA ATGGGCGGAA GGAGGTTTGA TGGCCCTGGA CAAGGTGACC AGCAGATGGA TGGACGTGGA CCGAATGGTG GGCCAATGGG CGGTAGGAGG TTTGATGGAC CAGGATTCGG TGGCTTCAGA CCCGAAGGTG CAGGGAGACC 320**

**He185/333_cDNA_0100 ATAGGCAAGG TGGTCCACCA ATGGGTGGAA GGAGGTTTGA TGGCAATGGA CAAGGTGACC AACAGATGGC TGGACGTGAA CCGAATGGCC GGCCAGTGGG CAGTAGAAAA TTTGATGGAC CAGGATTCGG TGGCTTCAGA CCCGAAGGTG CCGGGAGACC 317**

**He185/333_cDNA_0101 CTAGGCAAGG TGGTCCACCA ATGGGCGGAA GGAGGTTTGA TGGCCCTGGA CAAGGTGACC AGCAGATGGA TGGACGTGGA CCGAATGGTG GGCCAATGGG CGGTAGGAGG TTTGATGGAC CAGGATTCGG TGGCTTCAGA CCCGAAGGTG CAGGGAGACC 320**

**He185/333_cDNA_0102 ATAGGCAAGG TGGTCCACCA ATGGGTGGAA GGAGGTTTGA TGGCAATGGA CAAGGTGACC AACAGATGGC TGGACGTGAA CCGAATGGCC GGCCAGTGGG CAGTAGAAAA TTTGATGGAC CAGGATTCGG TGGCTTCAGA CCCGAAGGTG CCGGGAGACC 317**

**He185/333_cDNA_0103 CTAGGCAAGG TGGTGCACCA ATGGGCGGAA GGAGGTTTGA TGGCCCTGGA CAAGGTGATC AGCAGATGGA CGGACGTGGA CCGAATGGTG GGCCAATGGG CGGTAGGAGG TTTGATGGAC CAGGATTCGG TGGCTTCAGA CCCGAAGGTG CCGGGAGACC 320**

**He185/333_cDNA_0104 CTAGGCAAGG TGGTGCACCA ATGGGCGGAA GGAGGTTTGA TGGCCCTGGA CAAGGTGATC AGCAGATGGA CGGACGTGGA CCGAATGGTG GGCCAATGGG CGGTAGGAGG TTTGATGGAC CAGGATTCGG TGGCTTCAGA CCCGAAGGTG CAGGGAGACC 320**

**He185/333_cDNA_0105 CTAGGCAAGG TGGTCCGCCA ATGGGCGGAA GGAGGTTTGA TGGCCCTGGA CAAGGTGACC AGCAGATGGA TGGACGTGGA CCGAATGGTG GGCCAATGGG CGGTAGGAGG TTTGATGGAC CAGGATTCGG TGGCTTCAGA CTCGAAGGTG CAGGGAGACC 320**

**He185/333_cDNA_0106 CTAGGCAAGG TGGTCCGCCA ATGGGCGGAA GGAGGTTTGA TGGCCCTGGA CAAGGTGACC AGCAGATGGA TGGACGTGGA CCGAATGGTG GGCCAATGGG CGGTAGGAGG TTTGATGGAC CAGGATTCGG TGGCTTCAGA CTCGAAGGTG CAGGGAGACC 320**

**He185/333_cDNA_0107 CTAGGCAAGG TGGTCCGCCA ATGGGCGGAA GGAGGTTTGA TGGCCCTGGA CAAGGTGACC AGCAGATGGA TGGACGTGGA CCGAATGGTG GGCCAATGGG CGGTAGGAGG TTTGATGGAC ---------- ---------- ---------- ---------- 225**

**He185/333_cDNA_0108 CTAGGCAAGG TGGTCCGCCA ATGGGCGGAA GGAGGTTTGA TGGCCCTGGA CAAGGTGACC AGCAGATGGA TGGACGTGGA CCGAATGGTG GGCCAATGGG CGGTAGGAGG TTTGATGGAC CAGGATTCGG TGGCTTCAGA CTCGAAGGTG CAGGGAGACC 320**

**He185/333_cDNA_0109 CTAGGCAAGG TGGTCCACCA ATGGGCGGAA GGAGGTTTGA TGGCCCTGGA CAAGGTGACC AACAGA-GGA T--------- ---------- ---------- ---------- ---------- ---------- ---------- ---------- ---------- 192**

**He185/333_cDNA_0110 CTAGGCAAGG TGGTCCGCCA ATGGGCGGAA GGAGGTTTGA TGGCCCTGGA CAAGGTGACC AGCAGATGGA TGGACGTGGA CCGAATGGTG GGCCAATGGG CGGTAGGAGG TTTGATGGAC CAGGATTCGG TGGCTTCAGA CTCGAAGGTG CAGGGAGACC 320**

**He185/333_cDNA_0111 CTAGGCAAGG TGGTCCGCCA ATGGGCGGAA GGAGA----- ---------- ---------- ---------- ---------- ---------- ---------- ---------- ---------- ---------- ---------- ---------- --------CC 197**

**He185/333_cDNA_0112 CTAGGCAA-- ---------- ---------- ---------- ---------- ---------- ---------- ---------- ---------- ------TGGG CGGCAGGAGG TTTGATGGAC CAGGATTCGG TGGCTTCTGA CTCGAAGGTG CAGGGAGACC 232**

13

**....|....| ....|....| ....|....| ....|....| ....|....| ....|....| ....|....| ....|....| ....|....| ....|....| ....|....| ....|....| ....|....| ....|....| ....|....| ....|....|**

12

14

15

**325 335 345 355 365 375 385 395 405 415 425 435 445 455 465 475**

**He185/333_cDNA_0001 ---------- ---------- ---------- ---------- ---------- ---------- ---------- ---------- ---------- ---------- ---------- ---------- ---------- ---------- ---------- ---------- 201**

**He185/333_cDNA_0002 TTTCTTCGGT CACGGAGGAA GGCATGCTGA TGGAGAAGGA GAAATGGAGG CTGCTCAACC AATCGGTGAT GGTCAAGGAT GGCCCGGTCG TTTCGACGGT CCTGGAAGAT TTTCCGGACG TCCTTACCCA GGCCGTGGCG GTCATCATGG ACACCACCAT 480**

**He185/333_cDNA_0003 TTTCTTCGGT CACGGAGGAA GGCATGCTGA TGGAGAAGGA GAAATGGAGG CTGCTCAACC AATCGGTGAT GGTCAAGGAT GGCCCGGTCG TTTCGATGGT CCTGGAAGAT TTTCCGGACG TCCTTACCCA GGCCGTGGCG GTCATCATGG ACACCACCAT 480**

**He185/333_cDNA_0004 TTTCTTCGGT CACGGAGGAA GGCATGCTGA TGGAGAAGGA GAAATGGAGG CTGCTCAACC AATCGGTGAT GGTCAAGGAT GGCCCGGTCG TTTCGATGGT CCTGGAAGAT TTTCCGGACG TCCTTACCCA GGCCGTGGCG GTCATCATGG ACACCACCAT 480**

**He185/333_cDNA_0005 TTTCTTCGGT CACGGAGGAA GGCATGCTGA TGGAGAAGGA GAAATGGAGG CTGCTCAACC AATCGGTGAT GGTCAAGGAT GGCCCGGTCG TTTCGATGGT CCTGGAAGAT TTTCCGGACG TCCTTACCCA GGCCGTGGCG GTCATCATGG ACACCACCAT 480**

**He185/333_cDNA_0006 ---------- ---------- ---------- ---------- ---------- ---------- ---------- ---------- ---------- ---------- ---------- -----GGACG TCCTTACCCA GGCCGTGGCG GTCATCATGG ACACCACCAT 480**

**He185/333_cDNA_0007 TTTCTTCGGT CACGGAGGAA GGCATGCTGA TGGAGAAGGA GAAATGGAGG CTGCTCAACC AACCGGTGAT GGTCAAGGAT GGCCCGGTCG TTTCGATGGT CCTGGAAGAT TTTCCGGACG TCCTTACCCA GGCCGTGGCG GTCATCATGG ACACCACCAT 480**

**He185/333_cDNA_0008 TTTCTTCGGT CACGGAGGAA GGCATGCTGA TGGAGAAGGA GAAATGGAGG CTGCTCAACC AATCGGTGAT GGTCAAGGAT GGCCCGGTCG TTTCGATGGT CCTGGAAGAT TTTCCGGACG TCCTTACCCA GGCCGTGGCG GTCATCATGG ACACCACCAT 480**

**He185/333_cDNA_0009 TTTCTTCGGT CACGGAGGAA GGCATGCTGA TGGAGAAGGA GAAATGGAGG CTGCTCAACC AATCGGTGAT GGTCAAGGAT GGCCCGGTCG TTTCGATGGT CCTGGAAGAT TTTCCGGACG TCCTTACCCA GGCCGTGGCG GTCATCATGG ACACCACCAT 480**

**He185/333_cDNA_0010 TTTCTTCGGT CACGGAGGAA GGCATGCTGA TGGAGAAGGA GAAATGGAGG CTGCTCAACC AATCGGTGAT GGTCAAGGAT GGCCCGGTCG TTTCGATGGT CCTGGAAGAT TTTCCGGACG TCCTTACCCA GGCCGTGGCG GTCATCATGG ACACCACCAT 480**

**He185/333_cDNA_0011 TTTCTTCGGT CACGGAGGAA GGCATGCTGA TGGAGAAGGA GAAATGGAGG CTGCTCAACC AATCGGTGAT GGTCAAGGAT GGCCCGGTCG TTTCGATGGT CCTGGAAGAT TTTCCGGACG TCCTTACCCA GGCCGTGGCG GTCATCATGG ACACCACCAT 480**

**He185/333_cDNA_0012 TTTCTTCGGT CACGGAGGAA TGCATGCTGA TGGAGAAGGA GAAATGGAGG TTGCTCAACC AATCGGTGAT GGTCAAGGAT GGCCCGGTCG TTTCGATGGT CCTGGAAGGT TTTCCGGACG TCCTTACCCA GGCCGTGACG GT-------- ---------- 462**

**He185/333_cDNA_0013 TTTCTTCGGT CACGGAGGAA GGCATGCTGA TGGAGAAGGA GAGATGGGGG CTGCTCAACC AATCGGTGAT GGTCAAGGAT GGCCCGGTCG TTTCGATGGT CCTGGAAGAT TTTCCGGACG TCCTTACCCA GGCCGTGGCG GTCATCATGG ACACCACCAT 480**

**He185/333_cDNA_0014 TTTCTTCGGT CAAGGAGGAA TGCATGCTGA TGGAGAAGGA GAAATGGAGG TTGCTCAACC AATCGGTGAT GGTCAAGGAT GGCCCGGTCG TTTCGATGGT CCTGGAAGAT TTTCCGGACG TCCTTACCCA GGCCGTGACG GT-------- ---------- 462**

**He185/333_cDNA_0015 TTTCTTCGGT CACGGAGGAA TGCATGCTGA TGGAGAAGGA GAAATGGAGG TTGCTCAACC AATCGGTGAT GGTCAAGGAT GGCCCGGTCG TTTCGATGGT CCTGGAAGAT TTTCCGGACG TCCTTACCCA GGCCGTGACG GT-------- ---------- 462**

**He185/333_cDNA_0016 TTTCTTCGGT CACGGAGGAA TGCATGCTGA TGGAGAAGGA GAAATGGAGG TTGCTCAACC AATCGGTGAT GGTCAAGGAT GGCCCGGTCG TTTCGATGGT CCTGGAAGAT TTTCCGGACG TCCTTACCCA GGCCGTGACG GT-------- ---------- 462**

**He185/333_cDNA_0017 TTTCTTCGGT CACGGAGGAA GGCATGCTGA TGGAGAAGGA GAAATGGAGG CTGCTCAACC AATCGGTGAT GGTCAAGGAT GGCCCGGTCG TTTCGATGGT CCTGGAAGAT TTTCCGGACA TCCTCACCCA GGCCGTGGTG GTCATCATGG ACACCACCAT 480**

**He185/333_cDNA_0018 TTTCTTCGGT CACGGAGGAA GGCATGCTGA TGGAGAAGGA GAAATGGAGG CTGCTCAACC AATCGGTGAT GGTCAAGGAT GGCCCGGTCG TTTCGATGGT CCTGGAAGAT TTTCCGGACG TCCTTACCCA GGCCGTGGCG GTCATCATGG ACACCACCAT 480**

**He185/333_cDNA_0019 TTTCTTCGGT CACGGAGGAA GGCATGCTGA TGGAGAAGGA GAAATGGAGG CTGCTCAATC AATCGGTGAT GGTCAAGGAT GGCCCGGTCG TTTCGATGGT CCTGGAAGAT TTTCCGGACG TCCTTACCCA GGCCGTGGCG GTCATCATGG ACACCACCAT 480**

**He185/333_cDNA_0020 TTTCTTCGGT CACGGAGGAA GGCATGCTGA TGGAGAAGGA GAAATGGAGG CTGCTCAACC AATCGGTGAT GGTCAAGGAT GGCCCGGTCG TTTCGATGGT CCTGGAAGAT TTTCCGGACG TCCTTACCCA GGCCGTGGCG GTCATCATGG ACACCACCAT 438**

**He185/333_cDNA_0021 TTTCTTCGGT CACGGAGGAA GGCATGCTGA TGGAGAAGGA GAAATGGAGG CTGCTCAACC AATCGGTGAT GGTCAAGGAT GGCCCGGTCG TTTCGATGGT CCTGGAAGAT TTTCCGGACG TCCTTACCCA GGCCGTGGCG GTCATCATGG ACACCACCAT 480**

**He185/333_cDNA_0022 ---------- ---------- ---------- ---------- ---------- ---------- ---------- ---------- ---------- ---------- ---------- ---------- ---------- ---------- ---------- ---------- 102**

**He185/333_cDNA_0023 TTTCTTTGGT CACGGAGGAA TGCATGCTGA TGGAGAAGGA GAAATGGAGG TTCCTCAACC AATCGGTGAT GGTCAAGGAT GGCCCGATCG TTTCGATGGT CCTCGAAGAT TTTCCGGACG TCCTTACCCA GGCCGTGGCG GT-------- ---------- 459**

**He185/333_cDNA_0024 TTTCTTCGGT CAAGGAGGAA TGCATGCTGA TGGAGAAGGA GAAATGGAGG TTGCTCAACC AATCGGTGAT GGTCAAGGAT GGCCCGGTCG TTTCGATGGT CCTGGAAGAT TTTCCGGACG TCCTTACCCA GGCCGTGACG GT-------- ---------- 462**

**He185/333_cDNA_0025 TTTCTTTGGT CACGGAGGAA TGCATGCTGA TGGAGAAGGA GAAATGGAGG TTCCTCAACC AATCGGTGAT GGTCAAGGAT GGCCCGATCG TTTCGATGGT CCTCGAAGAT TTTCCGGACG TCCTTACCCA GGCCGTGGCG GT-------- ---------- 459**

**He185/333_cDNA_0026 TTTCTTCGGT CAAGGAGGAA TGCATGCTGA TGGAGAAGGA GAAATGGAGG TTGCTCAACC AATCGGTGAT GGTCAAGGAT GGCCCGGTCG TTTCGATGGT CCTGGAAGAT TTTCCGGACG TCCTTACCCA GGCCGTGACG GT-------- ---------- 462**

**He185/333_cDNA_0027 TTTCTTTGGT CACGGAGGAA TGCATGCTGA TGGAGAAGGA GAAATGGAGG TTCCTCAACC AATCGGTGAT GGTCAAGGAT GGCCCGATCG TTTCGATGGT CCTCGAAGAT TTTCCGGACG TCCTTACCCA GGCCGTGGCG GT-------- ---------- 459**

**He185/333_cDNA_0028 TTTCTTCGGT CAAGGAGGAA TGCATGCTGA TGGAGAAGGA GAAATGGAGG TTGCTCAACC AATCGGTGAT GGTCAAGGAT GGCCCGGTCG TTTCGATGGT CCTGGAAGAT TTTCCGGACG TCCTTACCCA GGCCGTGACG GT-------- ---------- 357**

**He185/333_cDNA_0029 TTTCTTTGGT CACGGAGGAA TGCATGCTGA TGGAGAAGGA GAAATGGAGG TTCCTCAACC AATCGGTGAT GGTCAAGGAT GGCCCGATCG TTTCGATGGT CCTCGAAGAT TTTCCGGACG TCCTTACCCA GGCCGTGGCG GT-------- ---------- 459**

**He185/333_cDNA_0030 TTTCTTCGGT CAAGGAGGAA TGCATGCTGA TGGAGAAGGA GAAATGGAGG TTGCTCAACC AATCGGTGAT GGTCAAGGAT GGCCCGGTCG TTTCGATGGT CCTGGAAGAT TTTCCGGACG TCCTTACCCA GGCCGTGACG GT-------- ---------- 462**

**He185/333_cDNA_0031 TTTCTTTGGT CACGGAGGAA TGCATGCTGA TGGAGAAGGA GAAATGGAGG TTCCTCAACC AATCGGTGAT GGTCAAGGAT GGCCCGATCG TTTCGATGGT CCTCGAAGAT TTTCCGGACG TCCTTACCCA GGCCGTGGCG GT-------- ---------- 459**

**He185/333_cDNA_0032 TTTCTTCGGT CAAGGAGGAA TGCATGCTGA TGGAGAAGGA GAAATGGAGG TTGCTCAACC AATCGGTGAT GGTCAAGGAT GGCCCGGTCG TTTCGATGGT CCTGGAAGAT TTTCCGGACG TCCTTACCCA GGCCGTGACG GT-------- ---------- 357**

**He185/333_cDNA_0033 TTTCTTTGGT CACGGAGGAA TGCATGCTGA TGGAGAAGGA GAAATGGAGG TTCCTCAACC AATCGGTGAT GGTCAAGGAT GGCCCGATCG TTTCGATGGT CCTCGAAGAT TTTCCGGACG TCCTTACCCA GGCCGTGGCG GT-------- ---------- 459**

**He185/333_cDNA_0034 TTTCTTTGGT CACGGAGGAA TGCATGCTGA TGGAGAAGGA GAAATGGAGG TTCCTCAACC AATCGGTGAT GGTCAAGGAT GGCCCGATCG TTTCGATGGT CCTCGAAGAT TTTCCGGACG TCCTTACCCA GGCCGTGGCG GT-------- ---------- 459**

**He185/333_cDNA_0035 TTTCTTTGGT CATGGAGGAA GGCATGCTGA TGGAGAAGGA GAAATGGAGG CTGCTCAACC AATCGGTGAT GGTCAAGGAT GGCCCGGTCG TTTCGATGGT CCTGGAAGAT TTTCCGGACG TCCTTACCCA GGACGTGGTG GTCATCATGG ACACCACCAT 480**

**He185/333_cDNA_0036 TTTCTTCGGT CACGGAGGAA GGCATGCTGA TGGAGAAGGA GAAATGGAGG CTGCTCAACC AATCGGTGAT GGTCAAGGAT GGCCCGGTCG TTTCGATGGT CCTGGAAGAT TTTCCGGACA TCCTCACCCA GGCCGTGGTG GTCATCATGG ACACCACCAT 480**

**He185/333_cDNA_0037 TTTCTTCGGT CAAGGAGGAA TGCATGCTGA TGGAGAAGGA GAAATGGAGG TTGCTCAACC AATCGGTGAT GGCCAAGGAT GGCCCGGTCG TTTCGATGGT CCTGGAAGAT TTTCCGGACG TCCTTACCCA GGCCGTGACG GT-------- ---------- 462**

**He185/333_cDNA_0038 TTTCTTCGGT CACGGAGGAA GGCATGCTGA TGGAGAAGGA GAAATGGAGG CTGCTCAACC AATCGGTGAT GGTCAAGGAT GGCCCGGTCG TTTCGATGGT CCTGGAAGAT TTTCCGGACG TCCTTACCCA GGCCGTGGCG GTCATCATGG ACACCACCAT 480**

**He185/333_cDNA_0039 TTTCTTCGGT CACGGAGGAA GGCATGCTGA TGGAGAAGGA GAAATGGAGG CTGCTCAACC AATCGGTGAT GGTCAAGGAT GGCCCGGTCG TTTCGATGGT CCTGGAAGAT TTTCCGGACG TCCTTACCCA GGCCGTGGCG GTCATCATGG ACACCACCAT 480**

**He185/333_cDNA_0040 TTTCTTCGGT CACGGAGGAA GGCATGCAGA TGGAGAAGGA GAAATGGAGG TTCCTCAACC AATCGGTGAT GGTCAAGGAT GGCCCGGTCG TTTCGATGGT CCTGGAAGAT TTTCCGGACG TCCTTACCCA GGACGTGGTG GTCACCATGA ACACCACCAT 480**

**He185/333_cDNA_0041 TTTCTTCGGT CACGGAGGAA GGCATGCTGA TGGAGAAGGA GAAATGGAGG CTGCTCAACC AATCGGTGAT GGTCAAGGAT GGCCCGGTCG TTTCGATGGT CCTGGAAGAT TTTCCGGACG TCCTTACCCA GGCCGTGGCG GTCATCATGG ACACCACCAT 480**

**He185/333_cDNA_0042 TTTCTTCGGT CACGGAGGAA GGCATGCTGA TGGAGAAGGA GAAATGGAGG CTGCTCAACC AATCGGTGAT GGTCAAGGAT GGCCCGGTCG TTTCGATGGT CCTGGAAGAT TTTCCGGACG TCCTTACCCA GGCCGTGGCG GTCATCATGG ACACCACCAT 480**

**He185/333_cDNA_0043 TTTCTTTGGT CACGGAGGAA TGCATGCTGA TGGAGAAGGA GAAATGGAGG TTCCTCAACC AATCGGTGAT GGTCAAGGAT GGCCCGATCG TTTCGATGGT CCTCGAAGAT TTTCCGGACG TCCTTACCCA GGCCGTGGCG GT-------- ---------- 459**

**He185/333_cDNA_0044 TTTCTTCGGT CACGGAGGAA GGCATGCTGA TGGAGAAGGA GAAATGGAGG CTGCTCAACC AATCGGTGAT GGTCAAGGAT GGCCCGGTCG TTTCGATGGT CCTGGAAGAT TTTCCGGACG TCCTTACCCA GGCCGTGGCG GTCATCATGG ACACCACCAT 480**

**He185/333_cDNA_0045 TTTCTTCGGT CACGGAGGAA GGCATGCAGA TGGAGAAGGA GAAATGGAGG TTCCTCAACC AATCGGTGAT GGTCAAGGAT GGCCCGGTCG TTTCGATGGT CCTGGAAGAT TTTCCGGACG TCCTTACCCA GGACGTGGTG GTCATCATGG ACACCACCAT 480**

**He185/333_cDNA_0046 TTTCTTCGGT CACGGAGGAA GGCATGCAGA TGGAGAAGGA GAAATGGAGG TTCCTCAACC AATCGGTGAT GGTCAAGGAT GGCCCGGTCG TTTCGATGGT CCTGGAAGAT TTTCCGGACG TCCTTACCCA GGACGTGGTG GTCATCATGG ACACCACCAT 480**

**He185/333_cDNA_0047 TTTCTTCGGT CAAGGAGGAA TGCATGCTGA TGGAGAAGGA GAAATGGAGG TTGCTCAACC AATCGGTGAT GGTCAAGGAT GGCCCGGTCG TTTCGATGGT CCTGGAAGAT TTTCCGGACG TCCTTACCCA GGCCGTGACG GT-------- ---------- 462**

**He185/333_cDNA_0048 TTTCTTCGGT CACGGAGGAA GGCATGCTGA TGGAGAAGGA GAAATGGAGG CTGCTCAACC AATCGGTGAT GGTCAAGGAT GGCCCGGTCG TTTCGTTGGT CCTGGAAGAT TTTCCGGACG TCCTTACCCA GGCCGTGGCG GTCATCATGG ACACCACCAT 480**

**He185/333_cDNA_0049 TTTCTTCGGT CACGGAGGAA GGCATGCTGA TGGAGAAGGA GAAATGGAGG CTGCTCAACC AATCGGTGAT GGTCAAGGAT GGCCCGGTCG TTTCGATGGT CCTGGAAGAT TTTCCGGACG TCCTTACCCA GGCCGTGGCG GTCATCATGG ACACCACCAT 480**

**He185/333_cDNA_0050 TTTCTTCGGT CACGGAGGAA GGCATGCTGA TGGAGAAGGA GAAATGGAGG CTGCTCAACC AATCGGTGAT GGTCAAGGAT GGCCCGGTCG TTTCGATGGT CCTGGAAGAT TTTCCGGACG TCCTTACCCA GGCCGTGGCG GTCATCATGG ACACCACCAT 480**

**He185/333_cDNA_0051 TTTCTTCGGT CACGGAGGAA GGCATGCAGA TGGAGAAGGA GAAATGGAGG TTCCTCAACC AATCGGTGAT GGTCAAGGAT GGCCCGGTCG TTTCGATGGT CCTGGAAGAT TTTCCGGACG TCCTTACCCA GGACGTGGTG GTCATCATGG ACACCACCAT 480**

**He185/333_cDNA_0052 TTTCTTCGGT CACGGAGGAA GGCATGCTGA TGGAGAAGGA GAAATGGAGG CTGCTCAACC AATCGGTGAT GGTCAAGGAT GGCCCGGTCG TTTCGATGGT CCTGGAAGAT TTTCCGGACG TCCTCAC--- ---------- ---------- ---------- 366**

**He185/333_cDNA_0053 TTTCTTCGGT CACGGAGGAA GGCATGCAGA TGGAGAAGGA GAAATGGAGG TTCCTCAACC AATCGGTGAT GGTCAAGGAT GGCCCGGTCG TTTCGATGGT CCTGGAAGAT TTTCCGGACG TCCTTACCCA GGACGTGGTG GTCATCATGG ACACCACCAT 480**

**He185/333_cDNA_0054 TTTCTTTGGT CACGGAGGAA TGCATGCTGA TGGAGAAGGA GAAATGGAGG TTCCTCAACC AATCGGTGAT GGTCAAGGAT GGCCCGATCG TTTCGATGGT CCTCGAAGAT TTTCCGGACG TCCTTACCCA GGCCGTGGCG GT-------- ---------- 459**

**He185/333_cDNA_0055 TTTCTTCGGT CAAGGAGGAA TGCATGCTGA TGGAGAAGGA GAAATGGAGG TTGCTCAACC AATCGGTGAT GGTCAAGGAT GGCCCGGTCG TTTCGATGGT CCTGGAAGAT TTTCCGGACG TCCTTACCCA GGCCGTGACG GT-------- ---------- 462**

**He185/333_cDNA_0056 TTTCTTCGGT CAAGGAGGAA TGCATGCTGA TGGAGAAGGA GAAATGGAGG TTGCTCAACC AATCGGTGAT GGTCAAGGAT GGCCCGGTCG TTTCGATGGT CCTGGAAGAT TTTCCGGACG TCCTTACCCA GGCCGTGACG GT-------- ---------- 462**

**He185/333_cDNA_0057 TTTCTTCGGT CACGGAGGAA GGCATGCTGA TGGAGAAGGA GAAATGGAGA CTGCTCAACC AATCGGTGAT GGTCAAGGAT GGCCCGGTCG TTTCGATGGT CCTGGAAGAT TTTCCGGACA TCCTCACCCA GGCCGTGGTG GTCATCATGG ACACCACCAT 480**

**He185/333_cDNA_0058 ---------- ---------- ---------- ---------- ---------- ---------- ---------- ---------- ---------- ---------- ---------- ---------- ---------- ---------- ---------- ---------- 303**

**He185/333_cDNA_0059 TTTCTTCGGT CACGGAGGAA GGCATGCTGA TGGAGAAGGA GAAATGGAGG CTGCTCAACC AATCGGTGAT GGTCAAGGAT GGCCCGGTCG TTTCGATGGT CCTGGAAGAT TTTCCGGACA TCCTCACCCA GGCCGTGGTG GTCATCATGG ACACCACCAT 480**

**He185/333_cDNA_0060 TTTCTTTGGT CATGGAGGAA GGCATGCTGA TGGAGAAGGA GAAATGGAGG CTGCTCAACC AATCGGTGAT GGTCAAGGAT GGCCCGGTCG TTTCGATGGT CCTGGAAGAT TTTCCGGACG TCCTTACCCA GGACGTGGTG GTCATCATGG ACACCACCAT 480**

**He185/333_cDNA_0061 TTTCTTCGGT CACGGAGGAA GGCATGCTGA TGGAGAAGGA GAAATGGAGG CTGCTCAACC AATCGGTGAT GGTCAAGGAT GGCCCGGTCG TTTCGATGGT CCTGGAAGAT TTTCCGGACA TCCTCACCCA GGCCGTGGTG GTCATCATGG ACACCACCAT 480**

**He185/333_cDNA_0062 ---------- ---------- ---------- ---------- ---------- ---------- ---------- ---------- ---------- ---------- ---------- ---------- ---------- ---------- ---------- ---------- 216**

**He185/333_cDNA_0063 TTTCTTCGGT CACGGAGGAA GGCATGCTGA TGGAGAAGGA GAAATGGAGG CTGCTCAACC AATCGGTGAT GGTCAAGGAT GGCCCGGTCG TTTCGATGGT CCTGGAAGAT TTTCCGGACA TCCTCACCCA GGCCGTGGTG GTCATCATGG ACACCACCAT 480**

**He185/333_cDNA_0064 TTTCTTCGGT CACGGAGGAA GGCATGCTGA TGGAGAAGGA GAAATGGAGG CTGCTCAACC AATCGGTGAT GGTCAAGGAT GGCCCGGTCG TTTCGATGGT CCTGGAAGAT TTTCCGGACA TCCTCACCCA GGCCGTGGTG GTCATCATGG ACACCACCAT 480**

**He185/333_cDNA_0065 TTTCTTCGGT CACGGAGGAA GGCATGCTGA TGGAGAAGGA GAAATGGAGG CTGCTCAACC AATCGGTGAT GGTCAAGGAT GGCCCGGTCG TTTCGATGGT CCTGGAAGAT TTTACGGACA TCCTCACCCA GGTCGTGGTG GTCATCATGG ACACCACCAT 405**

**He185/333_cDNA_0066 TTTCTTCGGT CACGGAGGAA GGCATGCTGA TGGAGAAGGA GAAATGGAGG CTGCTCAACC AATCGGTGAT GGT------- ---------- ---------- ---------- ---------- ---------- ---------- --CATCATGG ACACCACCAT 411**

**He185/333_cDNA_0067 TTTCTTCGGT CACGGAGGAA GGCATGCTGA TGGAGAAGGA GAAATGGAGG CTGCTCAACC AATCGGTGAT GGTCAAGGAT GGCCCGGTCG TTTCGATGGT CCTGGAAGAT TTTCCGGACA TCCTCACCCA GGCCGTGGTG GTCATCATGG ACACCACCAT 405**

**....|....| ....|....| ....|....| ....|....| ....|....| ....|....| ....|....| ....|....| ....|....| ....|....| ....|....| ....|....| ....|....| ....|....| ....|....| ....|....|**

12

13

14

15

**325 335 345 355 365 375 385 395 405 415 425 435 445 455 465 475**

**He185/333_cDNA_0068 TTTCTTCGGT CACGGAGGAA GGCATGCAGA TGGAGAAGGA GAAATGGAGG TTCCTCAACC AATCGGTGAT GGTCAAGGAT GGCCCGGTCG TTTCGATGGT CCTGGAAGAT TTTCCGGACG TCCTTACCCA GGACGTGGTG GTCATCATGG ACACCACCAT 480**

**He185/333_cDNA_0069 TTTCTTCGGT CACGGAGGAA GGCATGCTGA TGGAGAAGGA GAAATGGAGG CTGCTCGACC AATCGGTGAT GGTCAAGGAT GGCCCGGTCG TTTCGATGGT CCTGGAAGAT TTTCCGGACG TCCTTACCCA GGCCGTGGCG GTCATCATGG ACACCACCAT 480**

**He185/333_cDNA_0070 TTTCTTCGGT CACGGAGGAA GGCATGCTGA TGGAGAAGGA GAAATGGAGG CTGCTCAACC AATCGGTGAT GGTCAAGGAT GGCCCGGTCG TTTCGATGGT CCTGGAAGAT TTTCCGGACG TCCTTACCCA GGCCGTGGCG GTCATCATGG ACACCACCAT 480**

**He185/333_cDNA_0071 TTTCTTCGGT CACGGAGGAA GGCATGCTGA TGGAGAAGGA GAAATGGAGG CTGCTCAACC AATCGGTGAT GGTCAAGGAT GGCCCGGTCG TTTCGATGGT CCTGGAAGAT TTTCCGGACG TCCTTACCCA GGCCGTGGCG GTCATCATGG ACACCACCAT 480**

**He185/333_cDNA_0072 TTTCTTCGGT CACGGAGGAA GGCATGCTGA TGGAGAAGGA GAAATGGAGA CTGCTCAACC AATCGGTGAT GGTCAAGGAT GGCCCGGTCG TTTCGATGGT CCTGGAAGAT TTTCCGGACG TCCTTACCCA GGCCGTGGCG GTCATCATGG ACACCACCAT 480**

**He185/333_cDNA_0073 TTTCTTCGGT CACGGAGGAA GGCATGCTGA TGGAGAAGGA GAAATGGAGG CTGCTCAACC AATCGGTGAT GGTCAAGGAT GGCCCGGTCG TTTCGATGGT CCTGGAAGAT TTTCCGGACG TCCTTACCCA GGCCGTGGCG GTCATCATGG ACACCACCAT 480**

**He185/333_cDNA_0074 TTTCTTCGGT CACGGAGGAA GGCATGCTGA TGGAGAAGGA GAAATGGAGG CTGCTCAACC AATCGGTGAT GGTCAAGGAT GGCCCGGTCG TTTCGATGGT CCTGGAAGAT TTTCCGGACG TCCTTACCCA GGCCGTGGCG GTCATCATGG ACACCACCAT 480**

**He185/333_cDNA_0075 TTTCTTCGGT CACGGAGGAA GGCATGCTGA TGGAGAAGGA GAAATGGAGG CTGCTCAACC AATCGGTGAT GGTCAAGGAT GGCCCGGTCG CTTCGATGGT CCTGGAAGAT TTTCCGGACA TCCTCACCCA GGCCGTGGTG GTCATCATGG ACACCACCAT 480**

**He185/333_cDNA_0076 TTTCTTCGGT CACGGAGGAA GGCATGCTGA TGGAGAAGGA GAAATGGAGG CTGCTCAACC AATCGGTGAT GGTCAAGGAT GGCCCGGTCG TTTCGATGGT CCTGGAAGAT TTTCCGGACG TCCTTACCCA GGCCGTGGCG GTCATCATGG ACGCCACCAT 480**

**He185/333_cDNA_0077 TTTCTTCGGT CAAGGAGGAA TGCATGCTGA TGGAGAAGGA GAAATGGAGG TTGCTCAACC AATCGGTGAT GGTCAAGGAT GGCCCGGTCG TTTCGATGGT CCTGGAAGAT TTTCCGGACG TCCTTACCCA GGCCGTGACG GT-------- ---------- 462**

**He185/333_cDNA_0078 TTTCTTCGGT CACGGAGGAA GGCATGCTGA TGGAGAAGGA GAAATGGAGG CTGCTCAACC AATCGGTGAT GGTCAAGGAT GGCCCGGTCG TTTCGATGGT CCTGGAAGAT TTTCCGGACG TCCTTACCCA GGCCGTGGCG GTCATCATGG ACACCACCAT 480**

**He185/333_cDNA_0079 TTTCTTCGGT CACGGAGGAA GGCATGCTGA TGGAGAAGGA GAAATGGAGG CTGCTCAACC AATCGGTGAT GGTCAAGGAT GGCCCGGTCG TTTCGATGGT CCTGGAAGAT TTTCCGGACG TCCTTACCCA GGCCGTGGCG GTCATCATGG ACACCACCAT 480**

**He185/333_cDNA_0080 TTTCTTCGGT CACGGAGGAA GGCATGCTGA TGGAGAAGGA GAAATGGAGG CTGCTCAACC AATCGGTGAT GGTCAAGGAT GGCCCGGTCG TTTCGATGGT CCTGGAAGAT TTTCCGGACG TCCTTACCCA GGCCGTGGCG GTCATCATGG ACACCACCAT 480**

**He185/333_cDNA_0081 TTTCTTTGGT CACGGAGGAA TGCATGCTGA TGGAGAAGGA GAAATGGAGG TTCCTCAACC AATCGGTGAT GGTCAAGGAT GGCCCGATCG TTTCGATGGT CCTCGAAGAT TTTCCGGACG TCCTTACCCA GGCCGTGGCG GT-------- ---------- 459**

**He185/333_cDNA_0082 TTTCTTCGGT CACGGAGGAA GGCATGCTGA TGGAGAAGGG GAAATGGAGG CTGCTCAACC AATCGGTGAT GGTCAAGGAT GGCCCGGTCG TTTCGATGGT CCTGGAAGAT TTTCCGGACG TCCTTACCCA GGCCGTGGCG GTCATCATGG ACACCACCAT 480**

**He185/333_cDNA_0083 TTTCTTTGGT CACGGAGGAA TGCATGCTGA TGGAGAAGGA GAAATGGAGG TTCCTCAACC AATCGGTGAT GGTCAAGGAT GGCCCGATCG TTTCGATGGT CCTCGAAGAT TTTCCGGACG TCCTTACCCA GGCCGTGGCG GT-------- ---------- 459**

**He185/333_cDNA_0084 TTTCTTCGGT CACGGAGGAA GGCATGCTGA TGGAGAAGGG GAAATGGAGG CTGCTCAACC AATCGGTGAT GGTCAAGGAT GGCCCGGTCG TTTCGATGGT CCTGGAAGAT TTTCCGGACG TCCTTACCCA GGCCGTGACG GT-------- ---------- 462**

**He185/333_cDNA_0085 TTTCTTCGGT CACGGAGGAA GGCATGCTGA TGGAGAAGGG GAAATGGAGG CTGCTCAACC AATCGGTGAT GGTCAAGGAT GGCCCGGTCG TTTCGATGGT CCTGGAAGAT TTTCCGGACG TCCTTACCCA GGCCGTGGCG GTCATCATGG ACACCACCAT 480**

**He185/333_cDNA_0086 TTTCTTCGGT CAAGGAGGAA TGCATGCTGA TGGAGAAGGA GAAATGGAGG TTGCTCAACC AATCGGTGAT GGTCAAGGAT GGCCCGGTCG TTTCGATGGT CCTGGAAGAT TTTCCGGACG TCCTTACCCA GGCCGTGACG GT-------- ---------- 462**

**He185/333_cDNA_0087 TTTCTTCGGT CAAGGAGGAA TGCATGCTGA TGGAGAAGGA GAAATGGAGG TTGCTCAACC AATCGGTGAT GGTCAAGGAT GGCCCGGTCG TTTCGATGGT CCTGGAAGAT TTTCCGGACG TCCTTACCCA GGCCGTGACG GT-------- ---------- 462**

**He185/333_cDNA_0088 TTTCTTCGGT CACGGAGGAA GGCATGCTGA TGGAGAAGGA GAAATGGAGG CTGCTCAACC AATCGGTGAT GGTCAAGGAT GGCCCGGTCG TTTCGATGGT CCTGGAAGAT TTTCCGGACA TCCTCACCCA GGCCGTGGTG GTCATCATGG ACACCACCAT 480**

**He185/333_cDNA_0089 TTTCTTCGGT CACGGAGGAA GGCATGCTGA TGGAGAAGGA GAAATGGAGG CTGCTCAACC AATCGGTGAT GGTCAAGGAT GGCCCGGTCG TTTCGATGGT CCTGGAAGAT TTTCCGGACA TCCTCACACA GGCCGTGGTG GTCATCATGG ACACCACCAT 480**

**He185/333_cDNA_0090 TTTCTTCGGT CAAGGAGGAA TGCATGCTGA TGGAGAAGGA GAAATGGAGG TTGCTCAACC AATCGGTGAT GGTCAAGGAT GGCCCGGTCG TTTCGATGGT CCTGGAAGAT TTTCCGGACG TCCTTACCCA GGCCGTGACG GT-------- ---------- 462**

**He185/333_cDNA_0091 TTTCTTCGGT CAAGGAGGAA TGCATGCTGA TGGAGAAGGA GAAATGGAGG TTGCTCAACC AATCGGTGAT GGTCAAGGAT GGCCCGGTCG TTTCGATGGT CCTGGAAGAT TTTCCGGACG TCCTTACCCA GGCCGTGACG GT-------- ---------- 462**

**He185/333_cDNA_0092 TTTCTTCGGT CACGGAGGAA GGCATGCTGA TGGAGAAGGG GAAATGGAGG CTGCTCAACC AATCGGTGAT GGTCAAGGAT GGCCCGGTCG TTTCGATGGT CCTGGAAGAT TTTCCGGACG TCCTTACCCA GGCCGTGGCG GTCATCATGG ACACCACCAT 480**

**He185/333_cDNA_0093 ---------- ---------- ---------- ---------- ---------- ---------- ---------- ---------- ---------- ---------- ---------- ---------- ---------- ---------- ---------- ---------- 231**

**He185/333_cDNA_0094 TTTCTTCGGT CACGGAGGAA GGCATGCTGA TGGAGAAGGA GAAATGGAGG CTGCTCAACC AATCGGTGAT GGTCAAGGAT GGCCCGGTCG TTTCGATGGT CCTGGAAGAT TTTCCGGACA TCCTCACCCA GGCCGTGGTG GTCATCATGG ACACCACCAT 480**

**He185/333_cDNA_0095 TTTCTTCGGT CACGGAGGAA GGCATGCTGA TGGAGAAGGA GAAATGGAGG CTGCTCAACC AATCGGTGAT GGTCAAGGAT GGCCCGGTCG TTTCGATGGT CCTGGAAGAT TTTCCGGACA TCCTCACCCA GGCCGTGGTG GTCATCATGG ACACCACCAT 480**

**He185/333_cDNA_0096 TTTCTTCGGT CAAGGAGGAA TGCATGCTGA TGGAGAAGGA GAAATGGAGG TTGCTCAACC AATCGGTGAT GGTCAAGGAT GGCCCGGTCG TTTCGATGGT CCTGGAAGAT TTTCCGGACG TCCTTACCCA GGCCGTGACG GT-------- ---------- 462**

**He185/333_cDNA_0097 TTTCTTCGGT CAAGGAGGAA TGCATGCTGA TGGAGAAGGA GAAATGGAGG TTGCTCAACA AATCGGTGAT GGTCAAGGAT GGCCCGGTCG TTTCGATGGT CCTGGAAGAT TTTCCGGACG TCCTTACCCA GGCCGTGACG GT-------- ---------- 462**

**He185/333_cDNA_0098 TTTCTTCGGT CACGGAGGAA GGCATGCTGA TGGAGAAGGA GAAATGGAGG CTGCTCAACC AATCGGTGAT GGTCAAGGAT GGCCCGGTCG TTTCGATGGT CCTGGAAGAT TTTCCGGACA TCCTCACCCA GGCCGTGGTG GTCATCATGG ACACCACCAT 480**

**He185/333_cDNA_0099 TTTCTTCGGT CAAGGAGGAA TGCATGCTGA TGGAGAAGGA GAAATGGAGG TTGCTCAACC AATCGGTGAT GGTCAAGGAT GGCCCGGTCG TTTCGATGGT CCTGGAAGAT TTTCCGGACG TCCTTACCCA GGCCGTGACG GT-------- ---------- 462**

**He185/333_cDNA_0100 TTTCTTTGGT CACGGAGGAA TGCATGCTGA TGGAGAAGGA GAAATGGAGG TTCCTCAACC AATCGGTGAT GGTCAAGGAT GGCCCGATCG TTTCGATGGT CCTCGAAGAT TTTCCGGACG TCCTTACCCA GGCCGTGGCG GT-------- ---------- 459**

**He185/333_cDNA_0101 TTTCTTCGGT CAAGGAGGAA TGCATGCTGA TGGAGAAGGA GAAATGGAGG TTGCTCAACC AATCGGTGAT GGTCAAGGAT GGCCCGGTCG TTTCGATGGT CCTGGAAGAT TTTCCGGACG TCCTTACCCA GGCCGTGACG GT-------- ---------- 462**

**He185/333_cDNA_0102 TTTCTTCGGT CAAGGAGGAA TGCATGCTGA TGGAGAAGGA GAAATGGAGG TTGCTCAACC AATCGGTGAT GGTCAAGGAT GGCCCGGTCG TTTCGATGGT CCTGGAAGAT TTTCCGGACG TCCTTACCCA GGCCGTGACG GT-------- ---------- 459**

**He185/333_cDNA_0103 TTTCTTCGGT TACGGAGGAA GGCATGCTGA TGGAGAAAGA GAAATGGAGG CTGCTCAACC AATCGGTGAT GGTCAAGGAT GGCCCGGTCG TTTCGATGGT CCTGGAAAAT TTTCCGGACA TCCTCACCCA GGCCGTGGTG GTCATCATGG ACACCACCAT 480**

**He185/333_cDNA_0104 TTTCTTCGGT CAAGGAGGAA TGCATGCTGA TGGAGAAGGA GAAATGGAGG TTGCTCAACC AATCGGTGAT GGTCAAGGAT GGCCCGGTCG TTTCGATGGT CCTGGAAGAT TTTCCGGACG TCCTTACCCA GGCCGTGACG GT-------- ---------- 462**

**He185/333_cDNA_0105 TTTCTTCGGT CACGGAGGAA GGCATGCTGA TGGAGAAGGA GAAATGGAGG CTGCTCAACC AATC------ ---------- ---------- ---------- ---------- ---------- ---------- ---------- ---------- ---------- 384**

**He185/333_cDNA_0106 TTTCTTCGGT CACGGAGGAA GGCATGCTGA TGGAGAAGGA GAAATGGAGG CTGCTCAACC AATCGGTGAT GGTCAAGGAT GGCCCGGTCG TTTCGATGGT CCTGGAAGAT TTTCCGGACG TCCTTACC-- ---------- ---------- ---------- 450**

**He185/333_cDNA_0107 ---------- ---------- ---------- ---------- ---------- ---------- ---------- ---------- ---------- ---------- ---------- ---------- ---------- ---------- ---------- ---------- 282**

**He185/333_cDNA_0108 TTTCTTCGGT CACGGAGGAA GGCATGCTGA TGGAGAAGGA GAAATGGAGG CTGCTCAACC AATCGGTGAT GGTCAAGGAT GGCCCGGTCG TTTCGATGGT CCTGGAAGAT TTTCCGGACG TCCTTACCCA GGCCGTGGCG GTCATCATGG ACACCACCAT 480**

**He185/333_cDNA_0109 ---------- ---------- ---------- ---------- ---------- ---------- ---------- ---------- ---------- ---------- ---------- ---------- ---------- ---------- ---------- ---------- 225**

**He185/333_cDNA_0110 TTTCTTCGGT CACGGAGGAA GGCATGCTGA TGGAGAAGGA GAAATGGAGG CTGCTCAACC AATCGGTGAT GGTCAAGGAT GGCCCGGTCG TTTCGATGGT CCTGGAAGAT TTTCCGGACG TCCTTACCCA GGCCGTGGCG GTCATCATGG ACACCACCAC 480**

**He185/333_cDNA_0111 TTTCTTCGGT CACGGAGGAA GGCATGCTGA TGGAGAAGGA GAAATGGAGG CTGCTCAACC AATCGGTGAT GGCCAAGGAT GGCCCGGTCG TTTCGATGGT CCTGGAAGAT TTTCCGGACG TCCTTACCCA GGCCGTGGCG GTCATCATGG ACACCACCAT 357**

**He185/333_cDNA_0112 TTTCTTCGGT CACGGAGGAA GGCATGCTGA TGGAGAAGGA GAAATGGAGG CTGCTCAACC AATCGGTGAT GGTCAAGGAT GGCCCGGTCG TTTCGATGGT CCTGGAAGAT TTTCCGGACG TCCTTACCCA GGCCGTGGCG GTCATCATGG ACACCACCAT 392**

18

19

17

20

**....|....| ....|....| ....|....| ....|....| ....|....| ....|....| ....|....| ....|....| ....|....| ....|....| ....|....| ....|....| ....|....| ....|....| ....|....| ....|....|**

15

16

**485 495 505 515 525 535 545 555 565 575 585 595 605 615 625 635**

**He185/333_cDNA_0001 GGTCCTCACC ATGACCAGGC CGACGAACAA TCATTTGGTC AGCAAAACGA CAGCAGCAGC GAGGAGGATG GCCGACCTCA CCGTCACCAC CACCAC---- -----CATCA TCACCATGAC CGTCATAACA AGACAGACGA CCACCATCGC CATAATCACA 352**

**He185/333_cDNA_0002 GGTCCTCACC ATGACCAGGC CGACGAACAA TCATTTGGTC AGCAAAACGA CAGCAGCAGC GAGGAGGATG GCCGACCTCA CCGTCACCAC CACCAC---- -----CATCA TCACCATGAC CGTCATAACA AGACAGACGA CCACCATCAC CATAATCACA 631**

**He185/333_cDNA_0003 GGTCCTCACC ATGACCAGGC CGACGAACAA TCATTTGGTC AGCAAAACGA CAGCAGCAGC GAGGAGGATG GCCGACCTCA CCGTCACCAC CACCAC---- -----CATCA TCACCATGAC CGTCATAACA AGACAGACGA CCACCATCAC CATAATCACA 631**

**He185/333_cDNA_0004 GGTCCTCACC ATGACCAGGC CGACGAACAA TCATTTGGTC AGCAAAACGA CAGCAGCAGC GAGGAG---G GCCGACCTCA CCGTCACCAC CACCAC---- -----CATCA TCACCATGAC CGTCATAACA AGACAGACGA CCACCATCAC CATAATCACA 628**

**He185/333_cDNA_0005 GGTCCTCACC ATGGCCAGGC CGACGAACAA TCATTTGGTC AGCAAAACGA CAGCAGCAGC GAGGAGGATG GCCGACCTCA CCGTCACCAC CACCAC---- -----CATCA TCACCATGAC CGTCATAACA AGACAGACGA CCACCATCAC CATAATCACA 631**

**He185/333_cDNA_0006 GGTCCTCACC ATGACCAGGC CGACGAACAA TCATTTGGTC AGCAAAACGA CAGCAGCAGC GAGGAGGATG GCCGACCTCA CCGTCACCAC CACCAC---- -----CATCA TCACCATGAC CGTCATAACA AGACAGGCGA CCACCATCAC CATAATCACA 427**

**He185/333_cDNA_0007 GGTCCTCACC ATGACCAGGC CGACGAACAA TCATTTGGTC AGCAAAACGA CAGCAGCAGC GAGGAGGATG GCCGACCTCA CCGTCACCAC CACCAC---- -----CATCA TCACCATGAC CGTCATAACA AGACAGACGA CCACCATCAC CATAATCACA 631**

**He185/333_cDNA_0008 GGTCCTCACC ATGACCAGGC CGACGAACAA TCATTTGGTC AGCAAAACGA CAGCAGCAGC GAGGAGGATG GCCGACCTCA CCGTCACCAC CACCAC---- -----CATCA TCACCATGAC CGTCATAACA AGACAGACGA CCACCATCAC CATAATCACA 631**

**He185/333_cDNA_0009 GGTCCTCACC ATGACCAGGC CGACGAGCAA TCATTTGGTC AGCAAAACGA CAGCAGCAGC GAGGAGGATG GCCGACCTCA CCGTCACCAC CACCAC---- -----CATCA TCACCATGAC CGTCATAACA AGACAGACGA CCACCATCAC CATAATCACA 631**

**He185/333_cDNA_0010 GGTCCTCACC ATGACCAGGC CGACGAACAA TCATTTGGTC AGCAAAACGA CAGCAGCAGC GAGGAGGATG GCCGACCTCA CCGTCACCAC CACCAC---- -----CATCA TCACCATGAC CGTCATAACA AGACAGACGA CCACCATCAC CATAATCGCA 631**

**He185/333_cDNA_0011 GGTCCTCACC ATGGCCAGGC CGACGAACAA TCATTTGGTC AGCAAAACGA CAGCAGCAGC GAGGAGGATG GCCGACCTCA CCGTCACCAC CACCAC---- -----CATCA TCACCATGAC CGTCATAACA AGACAGACGA CCACCATCAC CATAATCACA 631**

**He185/333_cDNA_0012 ---------C ATGACAAGGC TGACGAACAA TCATTTGGTC AGCAAAACGA CAGCAGCAGC GAGGAGGATG GCCGACCTCA CCGTCACCAC CACCAC---- -----CATCA TCACCATGAC CGTCATAACA AGACAGACGA CCACCATCAT CATAATCACA 604**

**He185/333_cDNA_0013 GGTCCTCACC ATGACCAGGC CGACGAACAA CCATTTGGTC AGCAAAACGA CAGCAGCAGC GAGGAGGATG GCCGACCTCA CCGTCACCAC CACCAC---- -----CATCA TCACCATGAC CGTCATAACA AGACAGACGA CCACCATCAC CATAATCACA 631**

**He185/333_cDNA_0014 ---------C ATGACAAGGC TGACGAACAA TCATTTGGTC AGCAAAACGA CAGCAGCAGC GAGGAGGATG GCCGACCTCA CCGTCACCAC CACCAC---- -----CATCA TCACCATGAC CGTCATAACA AGACAGACGA CCACCATCAC CATAATCACA 604**

**He185/333_cDNA_0015 ---------C ATGACAAGGC TGACGAACAA TCATTTGGTC AGCAAAACGA CAGCAGCAGC GAGGAGGATG GCCGACCTCA CCGTCACCAC CACCAC---- -----CATCA TCACCATGAC CGTCATAACA AGACAGACGA CCACCATCAT CATAATCACA 604**

**He185/333_cDNA_0016 ---------C ATGACAAGGC TGACGAACAA TCATTTGGTC AGCAAAACGA CGGCAGCTGC GATGAGGATG GCCGACCTCA CCGTCCCCAC CCCCCC---- -----CATCA TCACCATGAC CGTCATAACA ACACAGACGA CCACCATCAT CATAATCACC 604**

**He185/333_cDNA_0017 GGTCCTCACC ATGACCAGGC AGACGAACAA TCATTTGGTC AGCAAAACGA CAGCAGCAGC GAGGAGGATG GCCGACCTCA CCGTCACCAC CACCACCACC AC---CATCA TCACCATGAC CGTCATAACA AGACAGGCGA CCACCATCAT CATAATCACA 637**

**He185/333_cDNA_0018 GGTCCTCACC ATGACCAGGC CGACGAACAA TCATTTGGTC AGTAAAACGA CAGCAGCAGC GAGGAGGATG GCCGACCTCA CCGTCACCAC CACCAC---- -----CATCA TCACCATGAC CGTCATAACA AGACAGACGA CCACCATCAC CATAATCACA 631**

**He185/333_cDNA_0019 GGTCCTCACC ATGACCAGGC CGACGAACAA TCATTTGGTC AGCAAAACGA CAGCAGCAGC GAGGAGGATG GCCGACCTCA CCGTCACCAC CACCAC---- -----CATCA TCACCATGAC CGTCATAACA AGACAGACGA CCACCATCAC CATAATCACA 631**

**He185/333_cDNA_0020 GGTCCTCACC ATGACCAGGC CGACGAACAA TCATTTGGTC AGCAAAACGA CAGCAGCAGC GAGGAGGATG GCCGACCTCA CCGTCACCAC CACCAC---- -----CATCA TCACCATGAC CGTCATAACA AGACAGACGA CCACCATCAC CATAATCACA 589**

**He185/333_cDNA_0021 GGTCCTCACC ATGACCAGGC CGACGAACAA TCATTTGGTC AGCAAAACGA CAGCAGCAGC GAGGAGGATG GCCGACCTCA CCGTCACCAC CACCAC---- -----CATCA TCACCATGAC CGTCATAACA AGACGGACGA CCACCATCAC CATAATCACA 631**

**He185/333_cDNA_0022 ---------- ---------- ---------- ---------- ---------- ---------- ---------- ---------- ---------- ---------- ---------- ---------- ---------- ---------- ---------- ---------- 102**

**He185/333_cDNA_0023 ---------C ATGACAAGGC TGACGAACAA TCATTTGGTC AGCAAAACGA CAGCAGCAGC GAGGAGGATG GCCGACCTCA CCATCACCAC CACCAC---- -----CATCA TCACCATGAC CGTCATAACA AGACAGACGA CCACCATCAC CATAATCACA 601**

**He185/333_cDNA_0024 ---------C ATGACAAGGC TGACGAACAA TCATTTGGTC AGCAAAACGA CAGCAGCAGC GAGGAGGATG GCCGACCTCA CCGTCACCAC CGCCACCAT---- ---------- ------- ---------- ---------- ---------- ---------- 552**

**He185/333_cDNA_0025 ---------C ATGACAAGGC TGACGAACAA TCATTTGGTC AGCAAAACGA CAGCAGCAGC GAGGAGGATG GCCGACCTCA CCATCACCAC CACCAC---- -----CATCA TCACCATGAC CGTCATAACA AGACAGACGA CCACCATCAC CATAATCACA 601**

**He185/333_cDNA_0026 ---------C ATGACAAGGC TGACGAACAA TCATTTGGTC AGCAAAACGA CAGCAGCAGC GAGGAGGATG GCCGACCTCA CCGTCACCAC CACCAC---- -----CATCA TCACCATGAC CGTCATAACA AGACAGACGA CCACCATCAC CATAATCACA 604**

**He185/333_cDNA_0027 ---------C ATGACAAGGC TGACGAACAA TCATTTGGTC AGCAAAACGA CAGCAGCAGC GAGGAGGATG GCCGACCTCA CCATCACCAC CACCAC---- -----CATCA TCACCATGAC CGTCATAACA AGACAGACGA CCACCATCAC CATAATCACA 601**

**He185/333_cDNA_0028 ---------C ATGACAAGGC TGACGAACAA TCATTTGGTC AGCAAAACGA CAGCAGCAGC GAGGAGGATG GCCGACCTCA CCGTCACCAC CACCAC---- -----CATCA TCACCATGAC CGTCATAACA AGACAGACGA CCGCCATCAC CATAATCACA 499**

**He185/333_cDNA_0029 ---------C ATGACAAGGC TGACGCACAA TCATTTGGTC AGCAAAACGA CAGCAGCAGC GAGGAGGATG GCCGACCTCA CCATCACCAC CACCAC---- -----CATCA TCACCATGAC CGTCATAACA AGACAGACGA CCACCATCAC CATAATCACA 601**

**He185/333_cDNA_0030 ---------C ATGACAAGGC TGACGAACAA TCATTTGGTC AGCAAAACGA CAGCAGCAGC GAGGAGGATG GCCGACCTCA CCGTCACCAC CACCAC---- -----CATCA TCACCATGAC CGTCATAACA AGACAGACGA CCACCATCAC CATAATCACA 604**

**He185/333_cDNA_0031 ---------C ATGACAAGGC TGACGAACAA TCATTTGGTC AGCAAAACGA CAGCAGCAGC GAGGAGGATG GCCGACCTCG CCATCACCAC CACCAC---- -----CATCA TCACCATGAC CGTCATAACA AGACAGACGA CCACCATCAC CATAATCACA 601**

**He185/333_cDNA_0032 ---------C ATGACAAGGC TGACGAACAA TCATTTGGTC AGCAAAACGA CAGCAGCAGC GAGGAGGATG GCCGACCTCA CCGTCACCAC CACCAC---- -----CATCA TCACCATGAC CGTCATAACA AGACAGACGA CCACCATCAC CATAATCACA 499**

**He185/333_cDNA_0033 ---------C ATGACAAGGC TGACGAACAA TCATTTGGTC AGCAAAACGA CAGCAGCAGC GAGGAGGATG GCCGACCTCA CCATCACCAC CAC------- -----CATCA TCACCATGAC CGTCATAACA AGACAGACGA CCACCATCAC CATAATCACA 598**

**He185/333_cDNA_0034 ---------C ATGACAAGGC TGACGAACAA TCATTTGGTC AGCAAAACGA CAGCAGCAGC GAGGAGGATG GCCGACCTCA CCATCACCAC CACCAC---- -----CATCA TCACCATGAC CGTCATAACA AGACAGACGA CCACCATCAC CATAATCACA 601**

**He185/333_cDNA_0035 GGTCCTCACC ATGACCAGGC AGACGAACAA TCATTTGGTC AGCAAAACGA CAGCAGCAGC GAGGAGGATG GCCGACCTCA CCGTCATCAC CACCACCACC ACCATCATCA TCACCATGAC CGTCATAACA AGACAGACGA CCACCGTCAT CATAATCACA 640**

**He185/333_cDNA_0036 GGTCCTCACC ATGACCAGGC AGACGAACAA TCATTTGGTC AGCAAAACGA CAGCAGCAGC GAGGAGGATG GCCGACCTCA CCGTCACCAC CACCACCACC AC---CATCA TCACCATGAC CGTCATAACA AGACAGGCGA CCACCATCAT CATAATCACA 637**

**He185/333_cDNA_0037 ---------C ATGACAAGGC TGACGAACAA TCATTTGGTC AGCAAAACGA CAGCAGCAGC GAGGAGGATG GCCGACCTCA CCGTCACCAC CACCACCAC- -----CATCA TCACCATGAC CGTCATAACA AGACAGACGA CCACCATCAC CATAATCACA 607**

**He185/333_cDNA_0038 GGTCCTCACC ATGACCAGGC CGACGAACAA TCATTTGGTC AGCAAAACGA CAGCAGCAGC GAGGAGGATG GCCGACCTCA CCGTCACCAC CACCAC---- -----CATCA TCACCATGAC CGTCATAACA AGACAGACGA CCACCATCAC CATAATCACA 631**

**He185/333_cDNA_0039 GGTCCTCACC GTGACCAGGC CGACGAACAA TCATTTGGTC AGCAAAACGA CAGCAGCAGC GAGGAGGATG GCCGACCTCA CCGTCACCAC CACCAC---- -----CATCA TCACCATGAC CGTCATAACA AGACAGACGA CCACCATCAC CATAATCACA 631**

**He185/333_cDNA_0040 GGTCCTCACC ATGACCAGGC AGACGAACAA TCATTTGGTC AGCAAAACGA CAGCAGCAGC GAGGAGGATG GCCGACCTCA CCATCATCAC CACCACCACC ACCATCATCA TCACCATGAC CGCCATAACA AGACAGACGA CCACCATCAC CATAATCACA 640**

**He185/333_cDNA_0041 GGTCCTCACC GTGACCAGGC CGACGAACAA TCATTTGGCC AGCAAAACGA CAGCAGCAGC GAGGAGGATG GCCGACCTCA CCGTCACCAC CACCAC---- -----CATCA TCACCATGAC CGTCATAACA AGACAGACGA CCACCATCAC CATAATCACA 631**

**He185/333_cDNA_0042 GGTCCTCACC ATGACCAGGC CGACGAACAA TCATTTGGTC AGCAAAACGA CAGCAGCAGC GAGGAGGATG GCCGACCTCA CCGTCACCAC CACCAC---- -----CATCA TCACCATGAC CGTCATAACA AGACAGACAA CCACCATCAC CATAATCACA 631**

**He185/333_cDNA_0043 ---------C ATGACAAGGC TGACGAACAA TCATTTGGTC AGCAAAACGA CAGCAGCAGC GAGGAGGATG GCCGACCTCA CCATCACCAC CACCAC---- -----CATCA TCACCATGAC CGTCATAACA AGACAGACGA CCACCATCAC CATAATCACA 601**

**He185/333_cDNA_0044 GGTCCTCACC ATGACCAGGC CGACGGACAA TCATTTGGTC AGCAAAACGA CAGCAGCAGC GAGGAGGATG GCCGACCTCA CCGTCACCAC CACCAC---- -----CATCA TCACCATGAC CGTCATAACA AGACAGACGA CCACCATCAC CATAATCACA 631**

**He185/333_cDNA_0045 GGTCCTCACC ATGACCAGGC AGACGAACAA TCATTTGGTC AGCAAAACGA CAGCAGCAGC GAGGAGGATG GCCGACCTCA CCATCATCAC CACCACCACC ACCATCATCA TCACCATGAC CGCCATAACA AGACAGACGA CCACCATCAC CATAATCACA 640**

**He185/333_cDNA_0046 GGTCCTCACC ATGACCAGGC AGACGAACAA TCATTTGGTC AGCAAAACGA CAGCAGCAGC GAGGAGGATG GCCGACCTCA CCATCATCAC CACCACCACC ACCATCATCA TCACCATGAC CGCCATAACA AGACAGACGA CCACCATCAC CATAATCACA 640**

**He185/333_cDNA_0047 ---------C ATGACAAGGC TGACGAACAA TCATTTGGTC AGCAAAACGA CAGCAGCAGC GAGGAGGATG GCCGACCTCA CCGTCACCAC CACCAC---- -----CATCA TCACCATGAC CGTCATAACA AGACAGACGA CCACCGTCAC CATAATCGCA 604**

**He185/333_cDNA_0048 GGTCCTCACC ATGACCAGGC CGACGAACAA TCATTTGGTC AGCAAAACGA CAGCAGCAGC GAGGAGGATG ACCGACCTCA CCGTCACCAC CACCAC---- -----CATCA TCACCATGAC CGTCATAACA AGACAGACGA CCACCATCAC CATAATCACA 631**

**He185/333_cDNA_0049 GGTCCTCACC ATGACCAGGC CGACGAACAA TCATTTGGTC AGCAAAACGA CAGCAGCAGC GAGGAGGATG GCCGACCTCA CCGTCACCAC CACCAC---- -----CATCA TCATCATGAC CGTCATAACA AGACAGACGA CCACCATCAC CATAATCACA 631**

**He185/333_cDNA_0050 GGTCCTCACC ATGACCAGGC CGACGAACAA TCATTTGGTC AGCAAAACGA CAGCAGCAGC GAGGAGGATG GCCGACCTCA CCGTCACCAC CACCAC---- -----CATCA TCACCATGAC CGTCATAACA AGACAGACGA CCACCATCAC CATAATCACA 631**

**He185/333_cDNA_0051 GGTCCTCACC ATGACCAGGC AGACGAACAA TCACTTGGTC AGCAAAACGA CAGCAGCAGC GAGGAGGATG GCCGACCTCA CCATCATCAC CACCACCACC ACCATCATCA TCACCATGAC CGCCATAACA AGACAGACGA CCACCATCAC CATAATCACA 640**

**He185/333_cDNA_0052 ---------C ATGACCAGGC CGACGAACAA TCATTTGGTC AGCAAAACGA CAGCAGCAGC GAGGAGGATG GCCGACCTCA CCGTCACCAC CACCAC---- -----CATCA TCACCATGAC CGTCATAACA AGACAGACGA CCACCATCAC CATAATCACA 514**

**He185/333_cDNA_0053 GGTCCTCACC ATGACCAGGC AGACGAACAA TCATTTGGTC AGCAAAACGA CAGCAGCAGC GAGGAGGATG GCCGACCTCA CCATCATCAC CACCACCACC ACCATCATCA TCACCATGAC CGCCATAACA AGACAGACGA CCACCATCAC CAT------- 633**

**He185/333_cDNA_0054 ---------C ATGACAAGGC TGACGAACAA TCATTTGGTC AGCAAAACGA CAGCAGCAGC GAGGAGGATG GCCGACCTCA CCATCACCAC CACCAT------- --CATCA TCACCATGAC CGTCATAACA AGACAGACGA CCACCATCAC CATAATCACA 601**

**He185/333_cDNA_0055 ---------C ATGACAAGGC TGACGAACAA TCATTTGGTC AGCAAAACGA CAGCAGCAGC GAGGAGGATG GCCGACCTCA CCGTCACCAC CACCAC---- -----CATCA TCACCATGAC CGCCATAACA AGACAGACGA CCACCATCAC CATAATCACA 604**

**He185/333_cDNA_0056 ---------C ATGACAAGGC TGACGAACAA TCATTTGGTC AGCAAAACGA CAGCAGCAGC GAGGAGGATG GCCGACCTCA CCGTCACCAC CACCAC---- -----CATCA TCACCATGAC CGTCATAACA AGACAGACGA CCACCATCAC CATAATCACA 604**

**He185/333_cDNA_0057 GGTCCTCACC ATGACCAGGC AGACGAACAA TCATTTGGTC AGCAAAACGA CAGCAGCAGC GAGGAGGATG GCCGACCTCA CCGTCACCAC CACCACCACC AC---CATCA TCACCATGAC CGTCATAACA AGACAGGCGA CCACCATCAT CATAATCACA 637**

**He185/333_cDNA_0058 ---------- ---------- ---------- ---------- ---------- ---------- ---------- ---------- ---------- ---------- ---------- ---------- ---------- ---------- ---------- ---------- 303**

**He185/333_cDNA_0059 GGTCCTCACC ATGACCAGGC AGACGAACAA TCATTTGGTC AGCAAAACGA CAGCAGCAGC GAGGAGGATG GCCGACCTCA CCGTCACCAC CACCACCACC AC---CATCA TCACCATGAC CGTCATAACA AGACAGGCGA CCACCATCAT CATAATCACA 637**

**He185/333_cDNA_0060 GGTCCTCACC ATGACCAGGC AGACGAACAA TCATTTGGTC AGCAAAACGA CAGCAGCAGC GAGGAGGATG GCCGACCTCA CCGTCATCAC CACCACCACC ACCATCATCA TCACCATGAC CGTCATAACA AGACAGACGA CCACCGTCAT CATAATCACA 640**

**He185/333_cDNA_0061 GGTCCTCACC ATGACCAGGC AGACGAACAA TCATTTGGTC AGCAAAACGA CAGCAGCAGC GAGGAGGATG GCCGACCTCA CCGTCACCAC CACCACCACC AC---CATCA TCACCATGAC CGTCATAACA AGACAGACGA CCACCATCAT CATAATCACA 637**

**He185/333_cDNA_0062 ---------- --GACCAGGC AGACGAACAA TCATTTGGTC AGCAAAACGA CAGCAGCAGC GAGGAGGATG GCCGACCTCA CCGTCACCAC CACCACCACC AC---CATCA TCACCATGAC CGTCATAACA AGACAGGCGA CCACCATCAT CATAATCACA 361**

**He185/333_cDNA_0063 GGTCCTCACC ATGACCAGGC AGGCGAACAA TCATTTGGTC AGCAAAACGA CAGCAGCAGC GAGGAGGATG GCCGACCTCA CCGTCACCAC CACCACCACC AC---CATCA TCACCATGAC CGTCATAACA AGACAGGCGA CCACCATCAT CATAATCACA 637**

**He185/333_cDNA_0064 GGTCCTCACC ATGACCAGGC AGACGAACAA TCATTTGGTC AGCAAAACGA CAGCAGCAGC GAGGAGGATG GCCGACCTCA CCGTCACCAC CACCACCACC AC---CATCA TCACCATGAC CGTCATAACA AGACAGGCGA CCACCATCAT CATAATCACA 637**

**He185/333_cDNA_0065 GGTCCTCACC ATGACCAGGC AGACGAACAA TCATTTGGTC AGCAAAACGA CAGCAGCAGC GAGGAGGATG GCCGACCTCA CCGTCACCAC CACCACCACC ACCACCATCA TCACCATGAC CGTCATAACA AGACAGGCGA CCACCATCAT CATAATCACA 565**

**He185/333_cDNA_0066 GGTCCTCACC ATGACCAGGC AGACGAACAA TCATTTGGTC AGCAAAACGA CAGCAGCAGC GAGGAAGATG GCCGACCTCA CCGTCACCAC CACCACCACC AC---CATCA TCACCATGAC CGTCATAACA AGACAGGCGA CCACCATCAT CATAATCACA 568**

**He185/333_cDNA_0067 GGTCCTCACC ATGACCAGGC AGACGAACAA TCATTTGGTC AGCAAAACGA CAGCAGCAGC GAGGAGGATG GCCGACCTCA CCGTCACCAC CACCACCACC AC---CATCA TCACCATGAC CGTCATAACA AGACAGGCGA CCACCATCAT CATAATCACA 562**

**....|....| ....|....| ....|....| ....|....| ....|....| ....|....| ....|....| ....|....| ....|....| ....|....| ....|....| ....|....| ....|....| ....|....| ....|....| ....|....|**

15

18

19

20

16

17

**485 495 505 515 525 535 545 555 565 575 585 595 605 615 625 635**

**He185/333_cDNA_0068 GGTCCTCACC ATGACCAGGC AGACGAACAA TCATTTGGTC AGCAAAACGA CAGCAGCAGC GAGGAGGATG GCCGACCTCA CCATCATCAC CACCACCACC ACCATCATCA TCACCATGAC CGCCATAACA AGACAGACGA CCACCATCAC CATAATCACA 640**

**He185/333_cDNA_0069 GGTCCTCACC ATGACCAGGC CGACGAACAA TCATTTGGTC AGCAAAACGA CAGCAGCAGC GAGGAGGATG GCCGACCTCA CCGTCACCAC CACCAC---- -----CATCA TCACCATGAC CGTCATAACA AGACAGACGA CCACCATCAC CATAATCACA 631**

**He185/333_cDNA_0070 GGTCCTCACC ATGACCAGGC CGACGAACAA TCATTTGGTC AGCAAAACGA CAGCAGCAGC GAGGAGGATG GCCGACCTCA CCGTCACCAC CACCAC---- -----CATCA TCACCATGAC CGTCATAACA AGACAGACGA CCACCATCAC CATAATCACA 631**

**He185/333_cDNA_0071 GGTCCTCACC ATGACCAGGC CGACGAACAA TCATTTGGTC AGCAAAGCGA CAGCAGCAGC GAGGAGGATG GCCGACCTCA CCGTCACCAC CACCAC---- -----CATCA TCACCATGAC CGTCATAACA AGACAGACGA CCACCATCAC CATAATCACA 631**

**He185/333_cDNA_0072 GGTCCTCACC ATGACCAGGC CGACGAACAA TCATTTGGTC AGCAAAACGA CAGCAGCAGC GAGGAGGATG GCCGACCTCA CCGTCACCAC CACCAC---- -----CATCA TCACCATGAC CGTCATAACA AGACAGACGA CCACCATCAC CATAATCACA 631**

**He185/333_cDNA_0073 GGTCCTCACC ATGACCAGGC CGACGAACAA TCATTTGGTC AGCAAAACGA CAGCAGCAGC GAGGAGGGTG GCCGACCTCA CCGTCACCAC CACCAC---- -----CATCA TCACCATGAC CGTCATAACA AGACAGACGA CCACCATCAC CATAATCACA 631**

**He185/333_cDNA_0074 GGTCCTCACC ATGACCAGGC CGACGAACAA TCATTTGGTC AGCAAAACGA CAGCAGCAGC GAGGAGGATG GCCGACCTCA CCGTCACCAC CACCAC---- -----CATCA TCACTATGAC CGTCATAACA AGACAGACGA CCACCGTCAT CATAATCACA 631**

**He185/333_cDNA_0075 GGTCCTCACC ATGACCAGGC AGACGAACAA TCATTTGGTC AGCAAAACGA CAGCAGCAGC GAGGAGGATG GCCGACCTCA CCGTCACCAC CACCGCCACC AC---CATCA TCACCATGAC CGTCATAACA AGACAGGCGA CCACCATCAT CATAATCACA 637**

**He185/333_cDNA_0076 GGTCCTCACC ATGACCAGGC CGACGAACAA TCATTTGGTC AGCAAAACGA CAGCAGCAGC GAGGAGGATG GCCGACCTCA CCGTCACCAC CACCAC---- -----CATCA TCACCATGAC CGTCATAACA AGACAGACGA CCACCATCAC CATAATCACA 631**

**He185/333_cDNA_0077 ---------C ATGACAAGGC TGACGAACAA TCATTTGGTC AGAAAAACGA CAGCAGCAGC GAGGAGGATG GCCGACCTCA CCGTCACCAC CACCAC---- -----CATCA TCACCATGAC CGTCATAACA AGACAGACGA CCACCATCAC CATAATCACA 604**

**He185/333_cDNA_0078 GGTCCTCACC ATGACCAGGC CGACGAACAA TCATTTGGTC AGCAAAACGA CAGCAGCAGC GAGGAGGATG GCCGACCTCA CCGTCACCAC CACCAC---- -----CATCA TCACCATGAC CGTCATAACA AGACAGACGA CCACCATCAC CATAATCACA 631**

**He185/333_cDNA_0079 GGTCCTCGCC ATGACCAGGC CGACGAACAA TCATTTGGTC AGCAAAACGA CAGCAGCAGC GAGGAGGATG GCCGACCTCA CCGTCACCAC CACCAC---- -----CATCA TCACCATGAT CGTCATAACA AGACAGACGA CCACCATCAC CATAATCACA 631**

**He185/333_cDNA_0080 GGTCCTCACC ATGACCAGGC CGACGAACAA TCATTTGGTC AGCAAAACGA CAGCAGCAGC GAGGAGGATG GCCGACCTCA CCGTCACCAC CACCAC---- -----CATCA TCACCATGAC CGTCATAACA GGACAGACGA CCACCATCAC CATAATCACA 631**

**He185/333_cDNA_0081 ---------C ATGACAAGGC TGACGAACAA TCATTTGGTC AGCAAAACGA CAGCAGCAGC GAGGAGGATG GTCGACCTCA CCATCACCAC CACCAC---- -----CATCA TCACCATGAC CGTCATAACA AGACAGACGA CCACCATCAC CATAATCACA 601**

**He185/333_cDNA_0082 GGTCCTCACC ATGACAAGGC CGACGAACAA TCATTTGGTC AGCAAAACGA CAGCAGCAGC GAGGAGGATG GCCGACCTCA CCGTCACCAC CACCAC---- -----CATCA TCACCATGAC CGTCATAACA AGACAAACGA CCACCATCAC CATAATCACA 631**

**He185/333_cDNA_0083 ---------C ATGACAAGGC TGACGAACAA TCATTTGGTC AGCAAAACGA CAGCAGCAGC GAGGAGGATG GCCGACCTCA CCGTCACCAC CACCAC---- -----CATCA TCACCATGAC CGTCATAACA AGACAAACGA CCACCATCAC CATAATCACA 601**

**He185/333_cDNA_0084 ---------C ATGACAAGGC TGACGAACAA TCATTTGGTC AGCAAAACGA CAGCAGCAGC GAGGAGGATG GCCGACCTCA CCGTCACCAC CACCAC---- -----CATCA TCACCATGAC CGTCATAACA AGACAAACGA CCACCATCAC CATAATCACA 604**

**He185/333_cDNA_0085 GGTCCTCACC ATGACAAGGC CGACGAACAA TCATTTGGTC AGCAAAACGA CAGCAGCAGC GAGGAGGATG GCCGACCTCA CCATCATCAC CACCACCACC ACCACCATCA TCACCATGAC CGTCATAACA AGACAGACGA CCACCATCAT CATAATCACA 640**

**He185/333_cDNA_0086 ---------C ATGACAAGGC TGACGAACAA TCATTTGGTC AGCAAAACGA CAGCAGCAGC GAGGAGGATG GCCGACCTCA CCGTCACCAC CACCAC---- -----CATCA TCACCATGAC CGTCATAACA AGACAAACGA CCACCATCAC CATAATCACA 604**

**He185/333_cDNA_0087 ---------C ATGACAAGGC TGACGAACAA TCATTTGGTC AGCAAAACGA CAGCAGCAGC GAGGAGGATG GCCGACCTCA CCGTCACCAC CACCAC---- -----CATCA TCACCATGAC CGTCATAACA AGACAAACGA CCACCATCAC CATAATCACA 604**

**He185/333_cDNA_0088 GGTCCTCACC ATGACCAGGC AGACGAACAA TCATTTGGTC AGCAAAACGA CAGCAGCAGC GAGGAGGATG GCCGACCTCA CCGTCACCAC CACCACCACC AC---CATCA GCACCATGAC CGTCATAACA AGACAGGCGA CCACCATCAT CATAATCACA 637**

**He185/333_cDNA_0089 GGTCCTCACC ATGACCAGGC AGACGAACAA TCATTTGGTC AGCAAAACGA CAGCAGCAGC GAGGAGGATG GCCGACCTCA CCGTCACCAC CACCACCACC AC---CATCA TCACCATGAC CGTCATAACA AGACAGGCGA CCACCATCAT CATAATCACA 637**

**He185/333_cDNA_0090 ---------C ATGACAAGGC TGACGAACAA TCATTTGGTC AGCAAAACGA CAGCATCAGC GAGGAGGATG GCCGACCTCA CCGTCACCAC CACCAC---- -----CATCA TCACCATGAC CGTCATAACA AGACAAACGA CCACCATCAC CATAATCACA 604**

**He185/333_cDNA_0091 ---------C ATGACAAGGC TGACGAACAA TCATTTGGTC AGCAAAACGA CAGCAGCAGC GAGGAGGATG GCCGACCTCA CCGTCACCAC CACCAC---- -----CATCA TCACCATGAC CGTCATAACA AGACAAACGA CCACCATCAC CATAATCACA 604**

**He185/333_cDNA_0092 GGTCCTCACC ATGACAAGGC CGACGAACAA TCATTTGGTC AGCAAAACGA CAGCAGCAGC GAGGAGGATG GCCGACCTCA CCATCATCAC CACCACCACC ACCACCATCA TCACCATGAC CGTCATAACA AGACAGACGA CCACCATCAT CATAATCACA 640**

**He185/333_cDNA_0093 ---------- ---------- ----GAACAA TCATTTGGTC AGCAAAACGA CAGCAGCAGC GAGGAGGATG GCCGACCTCA CCATCATCAC CACCACCACC ACCACCATCA TCACCATGAC CGTCATAACA AGACAGACGA CCACCATCAT CATAATCACA 367**

**He185/333_cDNA_0094 GGTCCTCACC ATGACCAGGC AGACGAACAA TCATTTGGTC AGCAAAACGA CAGCAGCAGC GAGGAGGATG GCCGACCTCA CCGTCACCAC CACCACCACC AC---CATCA TCACCATGAC CGTCATAACA AGACAGGCGA CCACCATCAT CATAATCACA 637**

**He185/333_cDNA_0095 GGTCCTCACC ATGACCAGGC AGACGAACAA TCATTTGGTC AGCAAAACGA CAGCAGCAGC GAGGAGGATG GCCGACCTCA CCGTCACCAC CACCACCACC AC---CATCA TCACCATGAC CGTCATAACA AGACAGGCGA CCACCATCAT CATAATCACA 637**

**He185/333_cDNA_0096 ---------C ATGACAAGGC TGACGAACAA TCATTTGGTC AGCAAAACGA CAGCAGCAGC GAGGAGGATG GCCGACCTCA CCGTCACCAC CACCAC---- -----CATCA TCACCATGAC CGTCATAACA AGACAAACGA CCACCATCAT CATAATCACA 604**

**He185/333_cDNA_0097 ---------C ATGACAAGGC TGACGAACAA TCATTTGGTC AGCAAAACGA CAGCAGCAGC GAGGAGGATG GCCGACCTCA CCGTCACCAC CACCAC---- -----CATCA TCACCATGAC CGTCATAACA AGACAAACGA CCACCATCAC CATAATCACA 604**

**He185/333_cDNA_0098 GGTCCTCACC ATGACCAGGC AGACGAACAA TCATTTGGTC AGCAAAACGA CAGCAGCAGC GAGGAGGATG GCCGACCTCA CCGTCACCAC CACCACCACC AC---CATCA GCACCATGAC CGTCATAACA AGACAGGCGA CCACCATCAT CATAATCACA 637**

**He185/333_cDNA_0099 ---------C ATGACAAGGC TGACGAACAA TCATTTGGTC AGCAAAACGA CAGCAGCAGC GAGGAGGATG GCCGACCTCA CCGTCACCAC CACCAC---- -----CATCA TCACCATGAC CGTCATAACA AGACAAACGA CCACCATCAC CATAATCACA 604**

**He185/333_cDNA_0100 ---------C ATGACAAGGC TGACGAACAA TCATTTGGTC AGCAAAACGA CAGCAGCAGC GAGGAGGATG GCCGACCTCA CCATCACCAC CACCAC---- -----CATCA TCACCATGAC CGTCATAACA AGACAGACGA CCACCATCAC CATAATCACA 601**

**He185/333_cDNA_0101 ---------C ATGACAAGGC TGACGAACAA TCATTTGGTC AGCAAAACGA CAGCAGCAGC GAGGAGGATG GCCGACCTCA CCGTCACCAC CACCAC---- -----CATCA TCACCATGAC CGTCATAACA AGACAAACGA CCACCATCAC CATAATCACA 604**

**He185/333_cDNA_0102 ---------C ATGACAAGGC TGACGAACAA TCATTTGGTC AGCAAAACGA CAGCAGCAGC GAGGAGGATG GCCGACCTCA CCGTCACCAC CACCAC---- -----CATCA TCACCATGAC CGTCATAACA AGACAAACGA CCACCATCAC CATAATCACA 601**

**He185/333_cDNA_0103 GGTCCTCACC ATGACCAGGC AGACGAACAA TCATTTGGTC AGCAAAACGA CAGCAGCAGC GAGGAGGATG GCCGACCTCA CCGTCACCAC CACCACCACC AC---CATCA GCACCATGAC CGTCATAACA AGACAGGCGA CCACCATCAT CATAATCTCA 637**

**He185/333_cDNA_0104 ---------C ATGACAAGGC TGACGAACAA TCATTTGGTC AGCAAAACGA CAGCAGCAGC GAGGAGGATG GCCGACCTCA CCGTCACCAC CACCAC---- -----CATCA TCACCATGAC CGTCATAACA AGACAAACGA CCACCATCAC CATAATCACA 604**

**He185/333_cDNA_0105 ---------- ---------- ---------- --ATTTGGTC AGCAAAACGA CAGCAGCAGC GAGGAGGATG GCCGACCTCA CCGTCACCAC CACCAC---- -----CATCA TCACCATGAC CGTCATAACA AGACAGACGA CCACCGTCAT CATAATCACA 503**

**He185/333_cDNA_0106 ---------C ACGACCAGGC CGACGAACAA TCATTTGGTC AGCAAAACGA CAGCAGCAGC GAGGAGGATG GCCGACCTCA CCGTCACCAC CACCAC---- -----CATCA TCACTATGAC CGTCATAACA AGACAGACGA CCACCGTCAT CATAATCACA 590**

**He185/333_cDNA_0107 ---------- ---------- ---------- ---------- ---------- ---------- ---------- ---------- ---------- ---------- -----CATCA TCACCATGAC CGTCATAACA AGACAGACGA CCACCATCAC CATAATCACA 335**

**He185/333_cDNA_0108 GGTCCTCACC ATGACCAGGC CGACGAACAA TCATTTGGTC AGCAAAATGA CAGCAGCAGC GAGGAGGATG GCCGACCTCA CCGTCACCAC CACCAC---- -----CATCA TCACCATGAC CGTCATAACA AGACAGACGA CCACCATCAC CATAATCACA 631**

**He185/333_cDNA_0109 ---------- ---------- ---------- ---------- ---------- ---------- ---------G GCCGACCTCA CCGTCACCAC CACCAC---- -----CATCA TCACCATGAC CGTCATAACA AGACAGACGA CCACCATCAC CATAATCACA 312**

**He185/333_cDNA_0110 GGTCCTCACC ATGACCAGGC CGACGAACAA TCATTTGGTC AGCAAAACGA CAGCAGCAGC GAGGAGGATG GCCGACCTCA CCGTCACCAC CACCAC---- -----CATCA TCACCATGAC CGTCATAACA AGACAGACGA CCACCATCAC CATAATCACA 631**

**He185/333_cDNA_0111 GGTCCTCACC ATGACCAGGC CGACGAACAA TCATTTGGTC AGCAAAACGA CAGCAGCAGC GAGGAGGATG GCCGACCTCA CCGTCACCAC CACCAC---- -----CATCA TCTCCATGAC CGTCATAACA AGACAGACGA CCACCATCAC CATAATCACA 508**

**He185/333_cDNA_0112 GGTCCTCACC ATGACCAGGC CGACGAACAA TCATTTGGTC AGCAAAACGA CAGCAGCAGC GAGGAGGATG GCCGACCTCA CCGTCACCAC CACCAC---- -----CATCA TCACCATGAC CGTCATAACA AGACAGACGA CCACCATCAC CATAATCACA 543**

**....|....| ....|....| ....|....| ....|....| ....|....| ....|....| ....|....| ....|....| ....|....| ....|....| ....|....| ....|....| ....|....| ....|....| ....|....| ....|....|**

20

21

22

**645 655 665 675 685 695 705 715 725 735 745 755 765 775 785 795**

**He185/333_cDNA_0001 CCGAAGGCCA CCGCCACCAT ---------- ---------- ---------- ---------- ---------- ---------- ---------- ---------- ---------- ---------- ---------- -CATCATAAC AAGACAGAAG AGGGTGACCA 401**

**He185/333_cDNA_0002 CCGAAGGCCA CCGCCACCAT ---------- ---------- ---------- ---------- ---------- ---------- ---------- ---------- ---------- ---------- ---------- -CATCATAAC AAGACAGAAG AGGGTGACCA 680**

**He185/333_cDNA_0003 CCGAAGGCCA CCGCCACCAT ---------- ---------- ---------- ---------- ---------- ---------- ---------- ---------- ---------- ---------- ---------- -CATCATAAC AAGACAGAAG AGGGTGACCA 680**

**He185/333_cDNA_0004 CCGAAGGCCA CCGCCACCAT ---------- ---------- ---------- ---------- ---------- ---------- ---------- ---------- ---------- ---------- ---------- -CATCATAAC AAGACAGAAG AGGGTGACCA 677**

**He185/333_cDNA_0005 CCGAAGGCCA CCGCCACCAT ---------- ---------- ---------- ---------- ---------- ---------- ---------- ---------- ---------- ---------- ---------- -CATCATAAC AAGACAGAAG AGGGTGACCA 680**

**He185/333_cDNA_0006 CCGAAGGCCA CCGCCACCAT ---------- ---------- ---------- ---------- ---------- ---------- ---------- ---------- ---------- ---------- ---------- -CATCATAAC AAGACAGAAG AGGGTGACCA 476**

**He185/333_cDNA_0007 CCGAAGGCCA CCGCCACCAT ---------- ---------- ---------- ---------- ---------- ---------- ---------- ---------- ---------- ---------- ---------- -CATCATAAC AAGACAGAAG AGGGTGACCA 680**

**He185/333_cDNA_0008 CCGAAGGCCA CCGCCACCAT ---------- ---------- ---------- ---------- ---------- ---------- ---------- ---------- ---------- ---------- ---------- -CATCATAAC AAGACAGAAG AGGGTGACCA 680**

**He185/333_cDNA_0009 CCGAAGGCCA CCGCCACCAT ---------- ---------- ---------- ---------- ---------- ---------- ---------- ---------- ---------- ---------- ---------- -CATCATAAC AAGACAGAAG AGGGTGACCA 680**

**He185/333_cDNA_0010 CCGAAGGCCA CCGCCACCAT ---------- ---------- ---------- ---------- ---------- ---------- ---------- ---------- ---------- ---------- ---------- -CATCATAAC AAGACAGGAG AGGGTGACCA 680**

**He185/333_cDNA_0011 CCGAAGGCCA CCGCCACCAT ---------- ---------- ---------- ---------- ---------- ---------- ---------- ---------- ---------- ---------- ---------- -CATCATAAC AAGACAGAAG AGGGTGACCA 680**

**He185/333_cDNA_0012 CCGAAGGCCA CCGCCACCAT ---------- ---------- ---------- ---------- ---------- ---------- ---------- ---------- ---------- ---------- ---------- -CATCATAAC AAGACAGAAG AGGGTGACCA 653**

**He185/333_cDNA_0013 CCGAAGGCCA CCGCCACCAT ---------- ---------- ---------- ---------- ---------- ---------- ---------- ---------- ---------- ---------- ---------- -CATCATAAC AAGACAGAAG AGGGTGACCA 680**

**He185/333_cDNA_0014 CCGAAGGCCA CCGCCACCAT ---------- ---------- ---------- ---------- ---------- ---------- ---------- ---------- ---------- ---------- ---------- -CATCATAAC AAGACAGAAG AGGGTGACCA 653**

**He185/333_cDNA_0015 CCGAAAGCCA CCGCCACCAT ---------- ---------- ---------- ---------- ---------- ---------- ---------- ---------- ---------- ---------- ---------- -CATCATAAC AAGACAGAAG AGGGTGACCA 653**

**He185/333_cDNA_0016 CCGAAGGCCG CCGCCCCCAT ---------- ---------- ---------- ---------- ---------- ---------- ---------- ---------- ---------- ---------- ---------- -CATCATTAC AACACAGAAG AAGGTGGCCA 653**

**He185/333_cDNA_0017 CCGAAGGCCA CCGCCACCAT CATAAC---- ---------- ---------- ---------- ---------- ---------- ---------- ---------- ---------- ---------- ---------- ---------- ---------- ---------- 663**

**He185/333_cDNA_0018 CCGAAGGCCA CCGCCACCAT ---------- ---------- ---------- ---------- ---------- ---------- ---------- ---------- ---------- ---------- ---------- -CATCATAAC AAGACAGAAG AGGGTGACCA 680**

**He185/333_cDNA_0019 CCGAAGGCCA CCGCCACCAT ---------- ---------- ---------- ---------- ---------- ---------- ---------- ---------- ---------- ---------- ---------- -CATCATAAC AAGACAGAAG AGGGTGACCA 680**

**He185/333_cDNA_0020 CCGAAGGCCA CCGCCACCAT ---------- ---------- ---------- ---------- ---------- ---------- ---------- ---------- ---------- ---------- ---------- -CATCATAAC AAGACAGAAG AGGGTGACCA 638**

**He185/333_cDNA_0021 CCGAAGGCCA CCGCCACCAT ---------- ---------- ---------- ---------- ---------- ---------- ---------- ---------- ---------- ---------- ---------- -CATCATAAC AAGACAGAAG AGGGTGACCA 680**

**He185/333_cDNA_0022 ---------- ---------- ---------- ---------- ---------- ---------- ---------- ---------- ---------- ---------- ---------- ---------- ---------- ---------- ---------- ---------- 102**

**He185/333_cDNA_0023 CCGAAGGCCA CCGCCACCAG ---------- ---------- ---------- ---------- ---------- ---------- ---------- ---------- ---------- ---------- ---------- -CATCATAAC AAGACAGACG AGGGTGACCA 650**

**He185/333_cDNA_0024 ---------- ---------- ---------- ---------- ---------- ---------- ---------- ---------- ---------- ---------- ---------- ---------- ---------- -CATCATAAC AAGACAGAAG AGGGTGACCA 581**

**He185/333_cDNA_0025 CCGAAGGCCA CCGCCACCAG ---------- ---------- ---------- ---------- ---------- ---------- ---------- ---------- ---------- ---------- ---------- -CATCATAAC AAGACAGAAG AGGGTGACCA 650**

**He185/333_cDNA_0026 CCGAAGGCCA CCGCCACCAT ---------- ---------- ---------- ---------- ---------- ---------- ---------- ---------- ---------- ---------- ---------- -CATCATAAC AAGACAGAAG AGGGTGACCA 653**

**He185/333_cDNA_0027 CCGAAGGCCA CCGCCACCAG ---------- ---------- ---------- ---------- ---------- ---------- ---------- ---------- ---------- ---------- ---------- -CATCATAAC AAGACAGAAG AGGGTGACCA 650**

**He185/333_cDNA_0028 CCGAAGGCCA CCGCCACCAT ---------- ---------- ---------- ---------- ---------- ---------- ---------- ---------- ---------- ---------- ---------- -CATCATAAC AAGACAGAAG AGGGTGACCA 548**

**He185/333_cDNA_0029 CCGAAGGCCA CCGCCACCAG ---------- ---------- ---------- ---------- ---------- ---------- ---------- ---------- ---------- ---------- ---------- -CATCATAAC AAGACAGAAG AGGGTGACCA 650**

**He185/333_cDNA_0030 CCGAAGGCCA CCGCCACCAT ---------- ---------- ---------- ---------- ---------- ---------- ---------- ---------- ---------- ---------- ---------- -CATCATAAC AAGACAGAAG AGGGTGACCA 653**

**He185/333_cDNA_0031 CCGAAGGCCA CCGCCACCAG ---------- ---------- ---------- ---------- ---------- ---------- ---------- ---------- ---------- ---------- ---------- -CATCATAAC AAGACAGAAG AGGGTGACCA 650**

**He185/333_cDNA_0032 CCGAAGGCCA CCGCCACCAT ---------- ---------- ---------- ---------- ---------- ---------- ---------- ---------- ---------- ---------- ---------- -CATCATAAC AAGACAGAAG AGGGTGACCA 548**

**He185/333_cDNA_0033 CCGAAGGCCA CCGCCACCAG ---------- ---------- ---------- ---------- ---------- ---------- ---------- ---------- ---------- ---------- ---------- -CATCATAAC AAGACAGAAG AGGGTGACCA 647**

**He185/333_cDNA_0034 CCGAAGGCCA CCGCCACCAG ---------- ---------- ---------- ---------- ---------- ---------- ---------- ---------- ---------- ---------- ---------- -CATCATAAC AAGACAGAAG AGGGCGACCA 650**

**He185/333_cDNA_0035 CCGAAGGCCA CCGCCACCAC ---------- ---------- ---------- ---------- ---------- ---------- ---------- ---------- ---------- ---------- ---------- -CATCATAAC AAGACAGAAG AGGGTGACCA 689**

**He185/333_cDNA_0036 CCGAAGGCCA CCGCCACCAT CATAACAAGA CAGACGACCA CCATCATCAT AATCACACCG AAGGCCACCG CCACCATCAT AACAAGACAG ACGACCACCA TCATCATAAT CACACCGAAG GCCACCGCCA CCATCATAAC AAGACAGAAG AGGGTGACCA 797**

**He185/333_cDNA_0037 CCGAAGGCCA CCGCCACCAT ---------- ---------- ---------- ---------- ---------- ---------- ---------- ---------- ---------- ---------- ---------- -CATCATAAC AAGACAGAAG AGGGTGACCA 656**

**He185/333_cDNA_0038 CCGAAGGCCA CCGCCACCAT ---------- ---------- ---------- ---------- ---------- ---------- ---------- ---------- ---------- ---------- ---------- -CACCATAAC AAGACAGAAG AGGGTGACCA 680**

**He185/333_cDNA_0039 CCGAAGGCCA CCGCCACCAT ---------- ---------- ---------- ---------- ---------- ---------- ---------- ---------- ---------- ---------- ---------- -CATCATAAC AAGACAGAAG AGGGTGACCA 680**

**He185/333_cDNA_0040 CCGAAGGCCA CCGCCACCAT ---------- ---------- ---------- ---------- ---------- ---------- ---------- ---------- ---------- ---------- ---------- -CATCATAAC AAGACAGAAG AGGGTGACCA 689**

**He185/333_cDNA_0041 CCGAAGGCCA CCGCCACCAT ---------- ---------- ---------- ---------- ---------- ---------- ---------- ---------- ---------- ---------- ---------- -CATCATAAC AAGACAGAAG AGGGTGACCA 680**

**He185/333_cDNA_0042 CCGAAGGCCA CCGCCACCAT ---------- ---------- ---------- ---------- ---------- ---------- ---------- ---------- ---------- ---------- ---------- -CATCATAAC AAGACAGAAG AGGGTGACCA 680**

**He185/333_cDNA_0043 CCGAAGGCCA CCGCCACCAG ---------- ---------- ---------- ---------- ---------- ---------- ---------- ---------- ---------- ---------- ---------- -CATCATAAC AAGACAGAAG AGGGTGACCA 650**

**He185/333_cDNA_0044 CCGAAGGCCA CCGCCACCAT ---------- ---------- ---------- ---------- ---------- ---------- ---------- ---------- ---------- ---------- ---------- -CATCATAAC AAGACAGAAG AGGGTGACCA 680**

**He185/333_cDNA_0045 CCGAAGGCCA CCGCCACCAT ---------- ---------- ---------- ---------- ---------- ---------- ---------- ---------- ---------- ---------- ---------- -CATCATAAC AAGACAGAAG AGGGTGACCA 689**

**He185/333_cDNA_0046 CCGAAGGCCA CCGCCACCAT ---------- ---------- ---------- ---------- ---------- ---------- ---------- ---------- ---------- ---------- ---------- -CATCATAAC AAGACAGAAG AGGGTGACCA 689**

**He185/333_cDNA_0047 CCGAAGGCCA CCGCCACCAT ---------- ---------- ---------- ---------- ---------- ---------- ---------- ---------- ---------- ---------- ---------- -CATCATAAC AAGACAGAAG AGGGTGACCA 653**

**He185/333_cDNA_0048 CCGAAGGCCA CCGCCACCAT ---------- ---------- ---------- ---------- ---------- ---------- ---------- ---------- ---------- ---------- ---------- -CATCATAAC AAGACAGAAG AGGGTGACCA 680**

**He185/333_cDNA_0049 CCGAAGGCCA CCGCCACCAT ---------- ---------- ---------- ---------- ---------- ---------- ---------- ---------- ---------- ---------- ---------- -CATCATAAC AAGACAGAAG AGGGTGACCA 680**

**He185/333_cDNA_0050 CCGAAGGCCA CCGCCACCAT ---------- ---------- ---------- ---------- ---------- ---------- ---------- ---------- ---------- ---------- ---------- -CATCATAAC AAGACAGAAG AGGGTGACCA 680**

**He185/333_cDNA_0051 CCGAAGGCCA CCGCCACCAT ---------- ---------- ---------- ---------- ---------- ---------- ---------- ---------- ---------- ---------- ---------- -CATCATAAC AAGACAGAAG AGGGTGACCA 689**

**He185/333_cDNA_0052 CCGAAGGCCA CCGCCACCAT ---------- ---------- ---------- ---------- ---------- ---------- ---------- ---------- ---------- ---------- ---------- -CATCATAAC AAGACAGAAG AGGGTGACCA 663**

**He185/333_cDNA_0053 ---------- ---------- ---------- ---------- ---------- ---------- ---------- ---------- ---------- ---------- ---------- ---------- ---------- -CATCATAAC AAGACAGAAG AGGGTGACCA 662**

**He185/333_cDNA_0054 CCGAAGGCCA CCGCCACCAG ---------- ---------- ---------- ---------- ---------- ---------- ---------- ---------- ---------- ---------- ---------- -CATCATAAC AAGACAGAAG AGGGTGACCA 650**

**He185/333_cDNA_0055 CCGAAGGCCA CCGCCACCAT ---------- ---------- ---------- ---------- ---------- ---------- ---------- ---------- ---------- ---------- ---------- -CATCACAAC AAGACAGAAG AGGGTGACCA 653**

**He185/333_cDNA_0056 CCGAAGGCCA CCGCCACCAT ---------- ---------- ---------- ---------- ---------- ---------- ---------- ---------- ---------- ---------- ---------- -CATCATAAC AAGACAGAAG AGGGTGACCA 653**

**He185/333_cDNA_0057 CCGAAGGCCA CCGCCACCAT CATAACAAGA CAGACGACCA CCATCATCAT AATCACACCG AAGGCCACCG CCACCATCAT AACAAGACAG ACGACCACCA TCATCATAAT CACACCGAAG GCCACCGCCA CCATCATAAC AAGACAGAAG AGGGTGACCA 797**

**He185/333_cDNA_0058 ---------- ---------- ---------- ---------- ---------- ---------- ---------- ---------- ---------- ---------- ---------- ---------- ---------- ---------- ---------- ---------- 303**

**He185/333_cDNA_0059 CCGAAGGCCA CCGCCAC--- ---------- ---------- ---------- ---------- ---------- ---------- ---------- ---------- ---------- ---------- ---------- -CATCATAAC AAGACAGAAG AGGGTGACCA 683**

**He185/333_cDNA_0060 CCGAAGGCCA CCGCCACCAC ---------- ---------- ---------- ---------- ---------- ---------- ---------- ---------- ---------- ---------- ---------- -CATCATAAC AAGACAGAAG AGGGTGACCA 689**

**He185/333_cDNA_0061 CCGAAAGCCA CCGCCACCAT CATAACAAGA CAGACGACCA CCATCATCAT AATCACACCG AAAGCCACCG CCACCATCAT AACAAGACAG ACGACCACCA TCATCATAAT CACACCGAAG GCCACCGCCA CCATCATAAC AAGACAGAAG AGGGTGACCA 797**

**He185/333_cDNA_0062 CCGAAGGCCA CCGCCACCAT CATAACAAGA CAGACGACCA CCATCATCAT AATCACACCG AAGGCCACCG CCACCATCAT AACAAGACAG ATGACCACCA TCATCATAAT CACACCGAAG GCCACCGCCA CCATCATAAC AAGACAGAAG AGGGTGACCA 521**

**He185/333_cDNA_0063 CCGAAGGCCA CCGCCACCAT CATAACAAGA CAGACGACCA CCATCATCAT AATCACACCG AAGGCCACCG CCACCATCAT AACAAGACGG ACGACCACCA TCATCATAAT CACACCGAAG GCCACCGCCA CCATCATAAC AAGACAGAAG AGGGTGACCA 797**

**He185/333_cDNA_0064 CCGAAGGCCA CCGCCACCAT CATAACAAGA CAGACGACCA CCATCATCAT AATCACACCG AAGGCCACCG CCACCATCAT AACAAGACAG ACGACCACCA TCATCATAAT CACACCGAAG GCCACCGCCA CCATCATAAC AAGACAGAAG AGGGTGACCA 797**

**He185/333_cDNA_0065 CCGAAGGCCA CTGCCACCAT CATAACAAGA CAGACGACCA CCATCATCAT AATCACACCG AAGGCCACCG CCACCATCAT AACAAGACAG ACGACCACCA TCATCATAAT CACACCGAAG GCCACCGCCA CCATCATAAC AAGACAGAAG AGGGTGACCA 725**

**He185/333_cDNA_0066 CCGAAGGCCA CCGCCACCAT CATAACAAGA CAGACGACCA CCATCATCAT AATCACACCG AAGGCCACCG CCACCATCAT AACAAGACAG ACGACCACCA TCATCATAAT CACACCGAAG GCCACCGCCA CCATCATAAC AAGACAGAAG AGGGTGACCA 728**

**He185/333_cDNA_0067 CCGAAGGCCA CCGCCAC--- ---------- ---------- ---------- ---------- ---------- ---------- ---------- ---------- ---------- ---------- ---------- -CATCATAAC AAGACAGAAG AGGGTGACCA 608**

**....|....| ....|....| ....|....| ....|....| ....|....| ....|....| ....|....| ....|....| ....|....| ....|....| ....|....| ....|....| ....|....| ....|....| ....|....| ....|....|**

22

20

21

**645 655 665 675 685 695 705 715 725 735 745 755 765 775 785 795**

**He185/333_cDNA_0068 CCGAAGGCCA CCGCCACCAT ---------- ---------- ---------- ---------- ---------- ---------- ---------- ---------- ---------- ---------- ---------- -CATCATAAC AAGACAGAAG AGGGTGACCA 689**

**He185/333_cDNA_0069 CCGAAGGCCA CCGCCACCAT ---------- ---------- ---------- ---------- ---------- ---------- ---------- ---------- ---------- ---------- ---------- -CATCATAAC AAGACAGAAG AGGGTGACCA 680**

**He185/333_cDNA_0070 CCGAAGGCCA CCGCCACCAT ---------- ---------- ---------- ---------- ---------- ---------- ---------- ---------- ---------- ---------- ---------- -CATCATAAC AAGACAGAAG AGGGTGACCA 680**

**He185/333_cDNA_0071 CCGAAGGCCA CCGCCACCAT ---------- ---------- ---------- ---------- ---------- ---------- ---------- ---------- ---------- ---------- ---------- -CATCATAAC AAGACAGAAG AGGGTGACCA 680**

**He185/333_cDNA_0072 CCGAAGGCCA CCGCCACCAT ---------- ---------- ---------- ---------- ---------- ---------- ---------- ---------- ---------- ---------- ---------- -CATCATAAC AAGACAGAAG AGGGTGACCA 680**

**He185/333_cDNA_0073 CCGAAGGCCA CCGCCACCAT ---------- ---------- ---------- ---------- ---------- ---------- ---------- ---------- ---------- ---------- ---------- -CATCATAAC AAGACAGAAG AGGGTGACCA 680**

**He185/333_cDNA_0074 CCGAAGGCCA CCGCCACCAT ---------- ---------- ---------- ---------- ---------- ---------- ---------- ---------- ---------- ---------- ---------- -CATCATAAC AAGACAGAAG AGGGTGACCA 680**

**He185/333_cDNA_0075 CCGAAGGCCA CCGCCACCAT CATAACAAGA CAGACGACCA CCATCATCAT AATCACACCG AAGGCCACCG CCACCATCAT AACAAGACAG ACGACCACCA TCATCATAAT CACACCGAAG GCCACCGCCA CCATCATAGC AAGACAGAAG AGGGTGACCA 797**

**He185/333_cDNA_0076 CCGAAGGCCA CCGCCACCAT ---------- ---------- ---------- ---------- ---------- ---------- ---------- ---------- ---------- ---------- ---------- -CATCATAAC AAGACAGAAG AGGGTGACCA 680**

**He185/333_cDNA_0077 CCGAAGGCCA CCGCCACCAT ---------- ---------- ---------- ---------- ---------- ---------- ---------- ---------- ---------- ---------- ---------- -CATCATAAC AAGACAGAAG AGGGTGACCA 653**

**He185/333_cDNA_0078 CCGAAGGCCA CCGCCACCAT ---------- ---------- ---------- ---------- ---------- ---------- ---------- ---------- ---------- ---------- ---------- -CATCATAAC AAGACAGAAG AGGGTGACCA 680**

**He185/333_cDNA_0079 CCGAAGGCCA CCGCCACCAT ---------- ---------- ---------- ---------- ---------- ---------- ---------- ---------- ---------- ---------- ---------- -CATCATAAC AAGACAGAAG AGGGTGACCA 680**

**He185/333_cDNA_0080 CCGAAGGCCA CCGCCACCAT ---------- ---------- ---------- ---------- ---------- ---------- ---------- ---------- ---------- ---------- ---------- -CATCATAAC AAGACAGAAG AGGGTGACCA 680**

**He185/333_cDNA_0081 CCGAAGGCCA CCGCCACCAG ---------- ---------- ---------- ---------- ---------- ---------- ---------- ---------- ---------- ---------- ---------- -CATCATAAC AAGACAGAAG AGGGTGACCA 653**

**He185/333_cDNA_0082 CCGAAGGCCA CCGCCACCAT ---------- ---------- ---------- ---------- ---------- ---------- ---------- ---------- ---------- ---------- ---------- -CATCATAAC AAGACAGAAG AGGGTGACCA 680**

**He185/333_cDNA_0083 CCGAAGGCCA CCGCCACCAT ---------- ---------- ---------- ---------- ---------- ---------- ---------- ---------- ---------- ---------- ---------- -CATCATAAC AAGACAGAAG AGGGTGACCA 650**

**He185/333_cDNA_0084 CCGAAGGCCA CCGCCACCAT ---------- ---------- ---------- ---------- ---------- ---------- ---------- ---------- ---------- ---------- ---------- -CATCATAAC AAGACAGAAG AGGGTGACCA 653**

**He185/333_cDNA_0085 CCGAAGGCCA CCGCCACCAT ---------- ---------- ---------- ---------- ---------- ---------- ---------- ---------- ---------- ---------- ---------- -CATCATAAC AAGACAGAAG AGGGTGACCA 689**

**He185/333_cDNA_0086 CCGAAGGCCA CCGCCACCAT ---------- ---------- ---------- ---------- ---------- ---------- ---------- ---------- ---------- ---------- ---------- -CATCATAAC AAGACAGAAG AGGGTGACCA 653**

**He185/333_cDNA_0087 CCGAAGGCCA CCGCCACCAT ---------- ---------- ---------- ---------- ---------- ---------- ---------- ---------- ---------- ---------- ---------- -CATCATAAC AAGACAGAAG AGGGTGACCA 653**

**He185/333_cDNA_0088 CCGAAGGCCA CCGCCACCAT CATAACAAGA CAGACGACCA CCATCATCAT AATCACACCG AAGGCCACCG CCACCATCAT AACAAGACAG ACGACCACCA TCATCATAAT CACACCGAAG GCCACCGCCA CCATCATAAC AAGACAGAAG AGGGTGACCA 797**

**He185/333_cDNA_0089 CCGAAGGCCA CCGCCACCAT CATGACAAGA CAGACGACCA CCATCATCAT AATCACACCG AAGGCCACCG CCACCATCAT AACAAGACAG ACGACCACCA TCATCATAAT CACACCGAAG GCCACCGCCA CCATCATAAC AAGACAGAAG AGGGTGACCA 797**

**He185/333_cDNA_0090 CCGAAGGCCA CCGCCACCAT ---------- ---------- ---------- ---------- ---------- ---------- ---------- ---------- ---------- ---------- ---------- -CATCATAAC AAGACAGAAG AGGGTGACCA 653**

**He185/333_cDNA_0091 CCGAAGGCCA CCGCCACCAT ---------- ---------- ---------- ---------- ---------- ---------- ---------- ---------- ---------- ---------- ---------- -CATCATAAC AAGACAGAAG AGGGTGACCA 653**

**He185/333_cDNA_0092 CCGAAGGCCA CCGCCACCAT ---------- ---------- ---------- ---------- ---------- ---------- ---------- ---------- ---------- ---------- ---------- -CATCATAAC AAGACAGAAG AGGGTGACCA 689**

**He185/333_cDNA_0093 CCGAAGGCCA CCGCCACCAT ---------- ---------- ---------- ---------- ---------- ---------- ---------- ---------- ---------- ---------- ---------- -CATCATAAC AAGACAGAAG AGGGTGACCA 416**

**He185/333_cDNA_0094 CCGAAGGCCA CCGCCACCAT CATGACAAGA CAGACGACCA CCATCATCAT AATCACACCG AAGGCCACCG CCACCATCAT AACAAGACAG ACGACCACCA TCATCATAAT CACACCGAAG GCCACCGCCA CCATCATAAC AAGACAGAAG AGGGTGACCA 797**

**He185/333_cDNA_0095 CCGAAGGCCA CCGCCACCAT CATGACAAGA CAGACGACCA CCATCATCAT AATCACACCG AAGGCCACCG CCACCATCAT AACAAGACAG ACGACCACCA TCATCATAAT CACACCGAAG GCCACCGCCA CCATCATAAC AAGACAGAAG AGGGTGACCA 797**

**He185/333_cDNA_0096 CCGAAGGCCA CCGCCACCAT ---------- ---------- ---------- ---------- ---------- ---------- ---------- ---------- ---------- ---------- ---------- -CATCATAAC AAGACAGAAG AGGGTGACCA 653**

**He185/333_cDNA_0097 CCGAAGGCCA CCGCCACCAT ---------- ---------- ---------- ---------- ---------- ---------- ---------- ---------- ---------- ---------- ---------- -CATCATAAC AAGACAGAAG AGGGTGACCA 653**

**He185/333_cDNA_0098 CCGAAGGCCA CCGCCACCAT CATAACAAGA CAGACGACCA CCATCATCAT AATCACACCG AAGGCCACCG CCACCATCAT AACAAGACAG ACGACCACCA TCATCATAAT CACACCGAAG GCCACCGCCA CCATCATAAC AAGACAGAAG AGGGTGACCA 797**

**He185/333_cDNA_0099 CCGAAGGCCA CCGCCACCAT ---------- ---------- ---------- ---------- ---------- ---------- ---------- ---------- ---------- ---------- ---------- -CATCATAAC AAGACAGAAG AGGGTGACCA 653**

**He185/333_cDNA_0100 CCGAAGGCCA CCGCCACCAG ---------- ---------- ---------- ---------- ---------- ---------- ---------- ---------- ---------- ---------- ---------- -CATCATAAC AAGACAGAAG AGGGTGACCA 650**

**He185/333_cDNA_0101 CCGAAGGCCA CCGCCACCAT ---------- ---------- ---------- ---------- ---------- ---------- ---------- ---------- ---------- ---------- ---------- -CATCATAAC AAGACAGAAG AGGGTGACCA 653**

**He185/333_cDNA_0102 CCGAAGGCCA CCGCCACCAT ---------- ---------- ---------- ---------- ---------- ---------- ---------- ---------- ---------- ---------- ---------- -CATCATAAC AAGACAGAAG AGGGTGACCA 650**

**He185/333_cDNA_0103 CCGAAGGCCA CCGCCACCAT CATAACAAGA CAGACGACCA CCATCATCAT AATCACACCG AAGGCCACCG CCACCATCAT AACAAGACAG ACGACCACCA TCATCATAAT CACACTGAAA GCCACCGCCA CCATCATAAC AAGACAGAAG AGGGTGACCA 797**

**He185/333_cDNA_0104 CCGAAGGCCA CCGCCACCAT ---------- ---------- ---------- ---------- ---------- ---------- ---------- ---------- ---------- ---------- ---------- -CATCATAAC AAGACAGAAG AGGGTGACCA 653**

**He185/333_cDNA_0105 CCGAAGGCCA CCGCCACCAT ---------- ---------- ---------- ---------- ---------- ---------- ---------- ---------- ---------- ---------- ---------- -CATCATAAC AAGACAGAAG AGGGTGACCA 552**

**He185/333_cDNA_0106 CCGAAGGCCA CCGCCACCAT ---------- ---------- ---------- ---------- ---------- ---------- ---------- ---------- ---------- ---------- ---------- -CATCATAAC AAGACAGAAG AGGGTGACCA 639**

**He185/333_cDNA_0107 CCGAAGGCCA CCGCCACCAT ---------- ---------- ---------- ---------- ---------- ---------- ---------- ---------- ---------- ---------- ---------- -CATCATAAC AAGACAGAAG AGGGTGACCA 384**

**He185/333_cDNA_0108 CCGAAGGCCA CCGCCACCAT ---------- ---------- ---------- ---------- ---------- ---------- ---------- ---------- ---------- ---------- ---------- -CATCATAAC AAGACAGAAG AGGGTGAC-A 679**

**He185/333_cDNA_0109 CCGAAGGCCA CCGCCACCAT ---------- ---------- ---------- ---------- ---------- ---------- ---------- ---------- ---------- ---------- ---------- -CATCATAAC AAGACAGAAG AGGGTGACCA 361**

**He185/333_cDNA_0110 CCGAAGGCCA CCGCCACCAT ---------- ---------- ---------- ---------- ---------- ---------- ---------- ---------- ---------- ---------- ---------- -CATCATAAC AAGACAGAAG AGGGTGAC-A 679**

**He185/333_cDNA_0111 CCGAAGGCCA CCGCCACCAT ---------- ---------- ---------- ---------- ---------- ---------- ---------- ---------- ---------- ---------- ---------- -CATCATAAC AAGACAGAAG AGGGTGACCA 557**

**He185/333_cDNA_0112 CCGAAGGCCA CCGCCACCAT ---------- ---------- ---------- ---------- ---------- ---------- ---------- ---------- ---------- ---------- ---------- -CATCATAAC AAGACAGAAG AGGGTGACCA 592**

**....|....| ....|....| ....|....| ....|....| ....|....| ....|....| ....|....| ....|....| ....|....| ....|....| ....|....| ....|....| ....|....| ....|....| ....|....| ....|....|**

27

26

25

24

23

22

**805 815 825 835 845 855 865 875 885 895 905 915 925 935 945 955**

**He185/333_cDNA_0001 GGACAGACCA GAGATGAGGC CATTCCGGTT CAACCCTTT- CGGTCGCAAA CCTTTCGGAG GACGTCCATT CGGCAGACGC AACCATACCG AAGAAGGATC TCCCAGGCGC GATGGC---- ---------- ----AACCGT GGACGTTGGG ATGGGAATGA 542**

**He185/333_cDNA_0002 GGACAGACCA GAGATGAGGC CATTCCGGTT CAACCCTTT- CGGTCGCAAA CCTTTCGGAA GACGTCAATT CGGCAGACGC AACCATACCG AAGAAGGATC TCCCAGGCGC GATGGC---- ---------- ----AACCGT GGACGTTGGG ATGAGAATGA 821**

**He185/333_cDNA_0003 GGACAGACCA GAGATGAGGC CATTCCGGTT CAACCCTTT- CGGTCGCAAA CCTTTCGGAA GACGTCAATT CGGCAGACGC AACCATACCG AAGAAGGATC TCCCAGGCGC GATGGC---- ---------- ----AACCGT GGACGTTGGG ATGAGAATGA 821**

**He185/333_cDNA_0004 GGACAGACCA GAGATGAGAC CATTCCGGTT CAACCCTTT- CGGTCGCAAA CCTTTCGGAG GACGTCCATT CGGCAGACGC AACCATACCG AAGAAGGGTC TCCCAGGCGC GATGGC---- ---------- ----AACCGT GGACGTTGGG ATGAGAATGA 818**

**He185/333_cDNA_0005 GGACAGACCA GAGATGAGGC CATTCCGGTT CAACCCTTT- CGGTCGCAAA CCTTTCGGAA GACGTCCATT CGGCAGACGC AACCATACCG AAGAAGGATC TCCCAGGCGC GATGGC---- ---------- ----AACCGT GGACGTTGGG ATGAGAATGA 821**

**He185/333_cDNA_0006 GGACAGACCA GAGATGAGGC CATTCCGGTT CAACCCTTT- CGGTCGCAAA CCTTTCGGAA GACGTCAATT CGGCAGACGC AACCATACCG AAGAAGGATC TCCCAGGCGC GATGGC---- ---------- ----AACCGT GGACGTTGGG ATGAGAATGA 617**

**He185/333_cDNA_0007 GGACAGACCA GAGATGAGGC CATTCCGGTT CAACCCTTT- CGGTCGCAAA CCTTTCGGAA GACGTCAATT CGGCAGACGC AACCATACCG AAGAAGGATC TCCCAGGTGC GATGGC---- ---------- ----AACCGT GGACGTTGGG ATGAGAATGA 821**

**He185/333_cDNA_0008 GGACAGACCA GAGATGAGGC CATTCCGGTT CAACCCTTT- CGGTCGCAAA CCTTTCGGAG GACGTCCATT CGGCAGACGC AACCATACCG AAGAAGGATC TCCCAGGCGC GATGGC---- ---------- ----AACCGT GGACGTTGGG ATGAGAATGA 821**

**He185/333_cDNA_0009 GGACAGACCA GAGATGAGGC CATTCCGGTT CAACCCTTT- CGGTCGCAAA CCTTTCGGAA GACGTCCATT CGGCAGACGC GACCATACCG AAGAAGGATC TCCCAGGCGC GATGGC---- ---------- ----AACCGT GGACGTTGGG ATGAGAATGA 821**

**He185/333_cDNA_0010 GGACAGACCA GAGATGAGGC CATTCCGGTT CGACCCTTT- CGGTCGCAAA CCTTTCGGAA GACGTCCATT CGGCAGACGC AACCATACCG AAGAAGGATC TCCCAGGCGC GATGGC---- ---------- ----AACCGT GGACGTTGGG ATGAGAATGA 821**

**He185/333_cDNA_0011 GGACAGACCA GAGATGAGGC CATTCCGGTT CAACCCTTT- CGGTCGCAAA CCTTTCGGAA GACGTCAATT CGGCAGACGC AACCATACCG AAGAAGGATC TCCCAGGCGC GATGGC---- ---------- ----AACCGT GGACGTTGGG ATGAGAATGA 821**

**He185/333_cDNA_0012 GGACAGACCA GAGATGGGGC CATTCCGGTT CAACCCTTT- CGGTCGCAAG CCTTTCGGAG GACGTCCATT CGGCAGATGC AACCATACCG AAGAAGGATC TCCCAGGCGC GATGGAGATC GTCGTCCCAA TGGCAACCGT GGACGTTGGG ATGAGAATGA 812**

**He185/333_cDNA_0013 GGACAGACCA GAGATGAGGC CATTCCGGTT CAACCCTTT- CGGTCGCAAA CCTTTCGGAG GACGTCCATT CGGCAGACGC AACCATACCG AAGAAGGATC TCCCAGGCGC GATGGC---- ---------- ----AACCGT GGACGTTGGG ATGAGAATGA 821**

**He185/333_cDNA_0014 GGACAGACCA GAGATGAGGC CATTCCGGTT CAACCCTTT- CGGTCGCAAG CCTTTCGGAG GACGTCCATT CGGCAGATGC AACCATACCG AAGAAGGATC TCCCAGGCGC GATGGAGATC GTCGTCCCAA GGGCAACCGT GGACGTTGGG ATGAGAATGA 812**

**He185/333_cDNA_0015 GGACAGACCA GAGATGAGGC CATTCCGGTT CAACCCTTT- CGGTCGCAAG CCTTTCGGAG GACGTCCATT CGGCGGATGC AACCATACCG AAGAAGGATC TCCCAGGCGC GATGGAGATC GTCGTCCCAA TGGCAACCGT GGACGTTGGG ATGAGAATGA 812**

**He185/333_cDNA_0016 GGACAGACCA GAGATGAGGC CATTCCGGTT CTTCCACCT- TTTTCGCAAG CAATTCGGAG GGCGTCCATT CGGCAGATGC AGCCATCACG AAGAAGGATG TTCCACCCGC GATGGAGATC GTCGTCCCAA TGGC------ ---------G ATGAGAATGA 797**

**He185/333_cDNA_0017 ---------- ---------- ---------- ---------- -------AAG CCTTTCGGAG GACGTCCATT CGGCAGACGC AACCATACCG ATGAAGGATC TCCTAGGCGC GATGGA---- ---------- ----CACCGT GGACGTTGGA ATGAGAATGA 758**

**He185/333_cDNA_0018 GGACAGACCA GAGATGAGGC CATTCCGGTT CAACCCTTT- CGGTCGCAAA CCTTTCGGAA GACGTCAATT CGGCAGACGC AACCATACCG AAGAAGGATC TCCCAGGCGC GATGGC---- ---------- ----AACCGT GGACGTTGGG ATGAGAATGA 821**

**He185/333_cDNA_0019 GGACAGACCA GAGATGAGGC CATTCCGGTT CAACCCTTT- CGGTCGCAAA CCTTTCGGAA GACGTCAATT CGGCAGACGC AACCATACCG AAGAAGGATC CCCCAGGCGC GATGGC---- ---------- ----AACCGT GGACGTTGGG ATGAGAATGA 821**

**He185/333_cDNA_0020 GGACAGACCA GAGATGAGGC CATTCCGGTT CAACCCTTT- CGGTCGCAAA CCTTTCGGAG GACGTCCATT CGGCAGACGC AACCATACCG AAGAAGGATC TCCCAGGCGC GATGGC---- ---------- ----AACCGT GGACGTTGGG ATGAGAATGA 779**

**He185/333_cDNA_0021 GGACAGACCA GAGATGAGGC CATTCCGGTT CAACCCTTT- CGGTCGCAAA CCTTTCGGAA GACGTCCATT CGGCAGACGC AACCATACCG AAGAAGGATC TCCCAGGCGC GATGGC---- ---------- ----AACCGT GGACGTTGGG ATGAGAATGA 821**

**He185/333_cDNA_0022 ---------- ---------- ---------- ---------- ---------- ---------- ---------- ---------- ---------- ---------- ---------- ---------- ---------- ---------- GGACGTTGGG ATGAGAATGA 122**

**He185/333_cDNA_0023 GGACAGTCCA GAGATGAGGC CATTCCGGTT CAACGCTTT- CGGTCGCAAG CCTTTCGGAG GACATCCATT CGGCAGACGC AACCATACCG AAGAAGGATC TCCCAGGCGC GATGGAGATC GTCGTCCCAA TGGCAACCGT GGACGTTGGG ATGAGAATGA 809**

**He185/333_cDNA_0024 GGACAGACCA GAGATGAGGC CATTCCGGTT CAACCCTTT- CGGTCGCAAG CCTTTCGGAG GACGTCCATT CGGCAGATGC AACCATACCG AAGAAGGATC TCCCAGGCGC GATGGAGATC GTCGTCCCAA GGGCAACCGT GGACGTTGGG ATGAGAATGA 740**

**He185/333_cDNA_0025 GGACAGACCA GAGATGAGGC CATTCCGGTT CAACGCTTT- CGGTCGCAAG CCTTTCGGAG GACATCCATT CGGCAGACGC AACCATACCG AAGAAGGATC TCCCAGGCGC GATGGAGATC GTCGTCCCAA TGGCAACCGT GGACGTTGGG ATGAGAATGA 809**

**He185/333_cDNA_0026 GGACAGACCA GAGATGAGGC CATTCCGGTT CAACCCTTT- CGGTCGCAAG CCTTTCGGAG GACGTCCATT CGGCAGATGC AACCATACCG AAGAAGGATC TCCCAGGCGC GATGGAGATC GTCGTCCCAA GGGCAACCGT GGACGTTGGG ATGAGAATGA 812**

**He185/333_cDNA_0027 GGACAGACCA GAGATGAGGC CATTCCGGTT CAACGCTTT- CGGTCGCAAG CCTTTCGGAG GACATCCATT CGGCAGACGC AACCATACCG AAGAAGGATC TCCCAGGCGC GATGGAGATC GTCGTCCCAA TGGCAACCGT GGACGTTGGG ATGAGAATGA 809**

**He185/333_cDNA_0028 GGACAGACCA GAGATGAGGC CATTCCGGTT CAACCCTTT- CGGTCGCAAG CCATTCGGAG GACGTCCATT CGGCAGATGC AACCATACCG AAGAAGGATC TCCCAGGCGC GATGGAGATC GTCGTCCCAA GGGCAACCGT GGACGTTGGG ATGAGAATGA 707**

**He185/333_cDNA_0029 GGACAGACCA GAGATGAGGC CATTCCGGTT CAACGCTTT- CGGTCGCAAG CCTTTCGGAG GACATCCATT CGGCAGACGC AACCATACCG AAGAAGGATC TCCCAGGCGC GATGGAGATC GTCGTCCCAA TGGCAACCGT GGACGTTGGG ATGAGAATGA 809**

**He185/333_cDNA_0030 GGACAGACCA GAGATGAGGC CATTCCGGTT CAACCCTTT- CGGTCGCAAG CCTTTCGGAG GACGTCCATT CGGCAGATGC AACCATACCG AAGAAGGATC TCCCAGGCGC GATGGAGATC GTCGTCCCAA GGGCAACCGT GGACGTTGGG ATGAGAATGA 812**

**He185/333_cDNA_0031 GGACAGACCA GAGATGAGGC CATTCCGGTT CAACGCTTT- CGGTCGCAAG CCTTTCGGAG GACATCCATT CGGCAGACGC AACCATACCG AAGAAGGATC TCCCAGGCGC GATGGAGATC GTCGTCCCAA TGGCAACCGT GGACGTTGGG ATGAGAATGA 809**

**He185/333_cDNA_0032 GGACAGACCA GAGATGAGGC CATTCCGGTT CAACCCTTT- CGGTCGCAAG CCTTTCGGAG GACGTCCATT CGGCAGATGC AACCATACCG AAGAAGGATC TCCCAGGCGC GATGGAGATC GTCGTCCCAA GGGCAACCGT GGACGTTGGG ATGAGAATGA 707**

**He185/333_cDNA_0033 GGACAGACCA GAGATGAGGC CATTCCGGTT CAACGCTTT- CGGTCGCAAG CCTTTCGGAG GACATCCATT CGGCAGACGC AACCATACCG AAGAAGGATC TCCCAGGCGC GATGGAGATC GTCGTCCCAA TGGCAACCGT GGACGTTGGG ATGAGAATGA 806**

**He185/333_cDNA_0034 GGACAGACCA GAGATGAGGC CATTCCGGTT CAACGCTTT- CGGTCGCAAG CCTTTCGGAG GACATCCATT CGGCAGACGC AACCATACCG AAGAAGGATC TCCCAGGCGC GATGGAGATC GTCGTCCCAA TGGCAACCGT GGACGTTGGG ATGAGAATGA 809**

**He185/333_cDNA_0035 GGACAGACCA GAGATGAGGC CATTCCGGTT CAACCTTTT- CGGTCGCAAG CCTTTCGGAG AACGTCCATT CGGCAGACGC AACCATACCG AAGAAGGATC TCCTAGACGC GATGAA---- ---------- ----CACCGT GGACGTTGGG ATGAGAATGA 830**

**He185/333_cDNA_0036 GGACAGACCA GAGATGAGGC CATTCTGGTT CAACCCTTT- CGGTCGCAAG CCTTTCGGAG GACGTCCATT CGGCAGACGC AACCATACCG ATGAAGGATC TCCTAGGCGC GATGGA---- ---------- ----CACCGT GGACGTTGGA ATGAGAATGA 938**

**He185/333_cDNA_0037 GGACAGACCA GAGATGAGGC CATTCCGGTT CAACCCTTT- CGGTCGCAAG CCTTTCGGAG GACGTCCATT CGGCAGATGC AACCATACCG AAGAAGGACC TCCCAGGCGC GATGGAGATC GTCGTCCCAA GGACAACCGT GGACGTTGGG ATGAGAATGA 815**

**He185/333_cDNA_0038 GGACAGACCA GAGATGAGGC CATTCCGGTT CAACTCTTT- CGGTCACAAA CCTTTCGGAA GACGTCCATT CGGCAGACGC AACCATACCG AAGAAGGATC TCCCAGGCGC GATGGC---- ---------- ----AACCGT GGACGTTGGG ATGAGAATGA 821**

**He185/333_cDNA_0039 GGACAGACCA GAGATGAGGC CATTCCGGTT CAACCCTTT- CGGTCGCAAA CCTTTCGGAA GACGTCAATT CGGCAGACGC AACCATACCG AAGAAGGATC TCCCAGGCGC GATGGC---- ---------- ----AACCGT GGACGTTGGG ATGAGAATGA 821**

**He185/333_cDNA_0040 GGACAGACCA GAGATGAGGC CATTCCGGTT CAACCCTTT- CGGTCGCAAA CCTTTCGGAG GACGTCCATT TGGCAGACGC AACCATACCG AAGAAGGATC TCCCAGGCGC GATGGC---- ---------- ----AACCGT GGACGTTGGG ATGAGAATGA 830**

**He185/333_cDNA_0041 GGACAGACCA GAGATGAGGC CATTCCGGTT CAACCCTTT- CGGTCGCAAA CCTTTCGGAA GACGTCCATT CGGCAGACGC AACCATACCG AAGAAGGATC TCCCAGGCGC GATGGC---- ---------- ----AACCGT GGACGTTGGG ATGAGAATGA 821**

**He185/333_cDNA_0042 GGACAGACCA GAGATGAGGC CATTCCGGTT CAACCCTTT- CGGTCGCAAA CCTTTCGGAA GACGTCAATT CGGCAGACGC AACCATACCG AAGAAGGATC TCCCAGGCGC GATGGC---- ---------- ----AACCGT GGACGTTGGG ATGAGAATGA 821**

**He185/333_cDNA_0043 GGACAGACCA GAGATGAGGC CATTCCGGTT CAACGCTTT- CGGTCGCAAG CCTTTCGGAG GACATCCATT CGGCAGACGC AACCATACCG AAGAAGGATC TCCCAGGCGC GATGGAGATC GTCGTCCCAA TGGCAACCGT GGACGTTGGG ATGAGAATGA 809**

**He185/333_cDNA_0044 GGACAGACCA GAGATGAGGC CATTCCGGTT CAACCCTTT- CGGTCGCAAA CCTTTCGGAG GACGTCCATT CGGCAGACGC AACCATACCG AAGAAGGATC TCCCAGGCGC GATGGC---- ---------- ----AACCGT GGACGTTGGG ATGAGAATGA 821**

**He185/333_cDNA_0045 GGACAGACCA GAGATGAGGC CATTCCGGTT CAACCCTTT- CGGTCGCAAA CCTTTCGGAG GACGTCCATT TGGCAGACGC AACCATACCG AAGAAGGATC TCCCAGGCGC GATGGC---- ---------- ----AACCGT GGACGTTGGG ATGAGAATGA 830**

**He185/333_cDNA_0046 GGACAGACCA GAGATGAGGC CATTCCGGTT CAACCCTTT- CGGTCGCAAA CCTTTCGGAG GACGTCCATT TGGCAGACGC AACCATACCG AAGAAGGATC TCCCAGGCGC GATGGC---- ---------- ----AACCGT GGACGTTGGG TTGAGAATGA 830**

**He185/333_cDNA_0047 GGACAGACCA GAGATGAGGC CATTCCGGTT CAACCCTTT- CGGTCGCAAG CCTTTCGGAG GACGTCCATT CGGCAGATGC AACCATACCG AAGAAGGATC TCCCAGGCGC GATGGAGATC GTCGTCCCAA GGGCAACCGT GGACGTTGGG ATGAGAATGA 812**

**He185/333_cDNA_0048 GGACAGACCA GAGATGAGGC CATTCCGGTT CAACCCTTT- CGGTCGCAAA CCTTTCGGAG GACGTCCATT CGGCAGACGC AACCATACCG AAGAAGGATC TCCCAGGCGC GATGGC---- ---------- ----AACCGT GGACGTTGGG ATGAGAATGA 821**

**He185/333_cDNA_0049 GGACAGACCA GAGATGAGGC CATTCCGGTT CAACCCTTT- CGGTCGCAAA CCTTTCGGAG GACGTCCATT CGGCAGACGC AACCATACCG AAGAAGGATC TCCCAGGCGC GATGGC---- ---------- ----AACCGT GGACGTTGGG ATGAGAATGA 821**

**He185/333_cDNA_0050 GGACAGACCA GAGATGAGGC CATTCCGGTT CAACCCTTT- CGGTCGCAAA CCTTTCGGAG GACGTCCATT CGGCAGACGC AACCATACCG AAGAAGGATC TCCCAGGCGC GATGGC---- ---------- ----AACCGT GGACGTTGGG ATGAGAATGA 821**

**He185/333_cDNA_0051 GGACAGACCA GAGATGAGGC CATTCCGGTT CAACCCTTT- CGGTCGCAAA CCTTTCGGAG GACGTCCATT TGGCAGACGC AACCATACCG AAGAAGGATC TCCCAGGCGC GATGGC---- ---------- ----AACCGT GGACGTTGGG ATGAGAATGA 830**

**He185/333_cDNA_0052 GGACAGACCA GAGATGAGGC CATTCCGGTT CAACCCTTT- CGGTCGCAAA CCTTTCGGAG GACGTCCATT CGGCAGACGC AACCATACCG AAGAAGGATC TCCCAGGCGC GATGGC---- ---------- ----AACCGT GGACGTTGGG ATGAGAATGA 704**

**He185/333_cDNA_0053 GGACAGACCA GAGATGAGGC CATTCCGGTT CAACCCTTT- CGGTCGCAAA CCTTTCGGAG GACGTCCATT TGGCAGACGC AACCATACCG AAGAAGGATC TCCCAGGCGC GATGGC---- ---------- ----AACCGT GGACGTTGGG ATGAGAATGA 803**

**He185/333_cDNA_0054 GGACAGACCA GAGATGAGGC CATTCCGGTT CAACGCTTT- CGGTCGCAAG CCTTTCGGAG GACATCCATT CGGCAGACGC AACCATACCG AAGAAGGATC TCCCAGGCGC GATGGAGATC GTCGTCCCAA TGGCAACCGT GGACGTTGGG ATGAGAATGA 809**

**He185/333_cDNA_0055 GGACAGACCA GAGATGAGGC CATTCCGGTT CAACCCTTT- CGGTCGCAAG CCTTTCGGAG GACGTCCATT CGGCAGATGC AACCATACCG AAGAAGGATC TCCCAGGCGC GATGGAGATC GTCGTCCCAA GGGCAACCGT GGACGTTGGG ATGAGAATGA 812**

**He185/333_cDNA_0056 GGACAGACCA GAGATGAGGC CATTCCGGTT CAACCCTTT- CGGTCGCAAG CCTTTCGGAG GACGTCCATT CGGCAGATGC AACCATACCG AAGAAGGATC TCCCAGGCGC GATGGAGATG GTCGTCCCAA GGGCAACCGT GGACGTTGGG ATGAGAATGA 812**

**He185/333_cDNA_0057 GGACAGACCA GAGATGAGGC CATTCTGGTT CAACCCTTT- CGGTCGCAAG CCTTTCGGAG GACGTCCATT CGGCAGACGC AACCATACCG ATGAAGGATC TCCTAGGCGC GATGGA---- ---------- ----CACCGT GGACGTTGGA ATGAGAATGA 938**

**He185/333_cDNA_0058 ---------- ---------- ---------- ---------- ---------- ---------- ---------- ---------- ---------- --GAAGGATC TCCTAGGCGC GATGGA---- ---------- ----CACCTT GGACGTTGGA ATGAGAATGA 353**

**He185/333_cDNA_0059 GGACAGACCA GAGATGAGGC CATTCTGGTT CAACCCTTT- CGGTCGCAAG CCTTTCGGAG GACGTCCATT CGGCAGACGC AACCATACCG ATGAAGGATC TCCTAGGCGC GATGGA---- ---------- ----CACCGT GGACGTTGGA ATGAGAATGA 824**

**He185/333_cDNA_0060 GGACAGACCA GAGATGAGGC CATTCCGGTT CAACCTTTT- CGGTCGCAAG CCTTTCGGAG AACGTCCATT CGGCAGACGC AACCATACCG AAGAAGGATC TCCTAGACGC GATGAA---- ---------- ----CACCGT GGACGTTGGG ATGAGAATGA 830**

**He185/333_cDNA_0061 GGACAGACCA GAGATGAGGC CATTCTGGTT CAACCCTTT- CGGTCGCAAG CCTTTCGGAG GACGTCCATT CGGCAGACGC AACCATACCG ATGAAGGATC TCCTAGGCGC GATGGA---- ---------- ----CACCGT GGACGTTGGA ATGAGAATGA 938**

**He185/333_cDNA_0062 GGACAGACCA GAGATGAGGC CATTCTGGTT CAACCCTTT- CGGTCGCAAG CCTTTCGGAG GACGTCCATT CGGCAGACGC AACCATACCG ATGAAGGATC TCCTAGGCGC GATGGA---- ---------- ----CACCGT GGACGTTGGA ATGAGAATGA 662**

**He185/333_cDNA_0063 GGACAGACCA GAGATGAGGC CATTCTGGTT CAACCCTTT- CGGTCGCAAG CCTTTCGGAG GACGTCCATT CGGCAGACGC AACCATACCG ATGAAGGATC TCCTAGGCGC GATGGA---- ---------- ----CACCGT GGACGTTGGA ATGAGAATGA 938**

**He185/333_cDNA_0064 GGACAGACCA GAGATGAGGC CATTCTGGTT CAACCCTTT- CGGTCGCAAG CCTTTCGGAG GACGTCCATT CGGCAGACGC AACCATACCG ATGAAGGATC TCCTAGGCGC GATGGA---- ---------- ----CACCGT GGACGTTGGA ATGAGAATGA 938**

**He185/333_cDNA_0065 GGACAGACCA GAGATGAGGC CATTCTGGTT CAACCCTTT- CGGTCGCAAG CCTTTCGGAG GACGTCCATT CGGCAGACGC AACCATACCG ATGAAGGATC TCCTAGGCGC GATGGA---- ---------- ----CACCGT GGACGTTGGA ATGAGAATGA 865**

**He185/333_cDNA_0066 GGACAGACCA GAGATGAGGC CATTCTGGTT CAACCCTTT- CGGTCGCAAG CCTTTCGGAG GACGTCCATT CGGCAGACGC AACCATACCG ATGAAGGATC TCCTAGGCGC GATGGA---- ---------- ----CACCGT GGACGTTGGA ATGAGAATGA 869**

**He185/333_cDNA_0067 GGACAGACCA GAGATGAGGC CATTCTGGTT CAACCCTTT- CGGTCGCAAG CCTTTCGGAG GACGTCCATT CGGCAGACGC AACCATACCG ATGAAGGATC TCCTAGGCGC GATGGA---- ---------- ----CACCGT GGACGTTGGA ATGAGAATGA 749**

**....|....| ....|....| ....|....| ....|....| ....|....| ....|....| ....|....| ....|....| ....|....| ....|....| ....|....| ....|....| ....|....| ....|....| ....|....| ....|....|**

27

26

25

24

23

22

**805 815 825 835 845 855 865 875 885 895 905 915 925 935 945 955**

**He185/333_cDNA_0068 GGACAGACCA GAGATGAGGC CATTCCGGTT CAACCCTTT- CGGTCGCAAA CCTTTCGGAG GACGTCCATT TGGCAGACGC AACCATACCG AAGAAGGATC TCCCAGGTGC GATGGC---- ---------- ----AACCGT GGACGTTGGG ATGAGAATGA 830**

**He185/333_cDNA_0069 GGACAGACCA GAGATGAGGC CATTCCGGTT CAACCCTTT- CGGTCGCAAA CCTTTCTGAA GACGTCAATT CGGCAGACGC AACCATACCG AAGAAGGATC TCCCAGGCGC GATGGC---- ---------- ----AACCGT GGACGTTGGG ATGAGAATGA 821**

**He185/333_cDNA_0070 GGACAGACCA GAGATGAGGC CATTCCGGTT CAACCCTTT- CGGTCGCAAA CCTTTCGGAG GACGTCCATT CGGCAGACGC AACCATACCG AAGAAGGATC TCCCAGGCGC GATGGC---- ---------- ----AACCGT GGACGTTGGG ATGAGAATGA 821**

**He185/333_cDNA_0071 GGACAGACCA GAGATGAGGC CATTCCGGTT CAACCCTTT- CGGTCGCAAA CCTTTCGGAG GACGTCCATT CGGCAGACGC AACCATACCG AAGAAGGATC TCCCAGGCGC GATGGC---- ---------- ----AACCGT GGACGTTGGG ATGAGAATGA 821**

**He185/333_cDNA_0072 GGACAGACCA GAGATGAGGC CATTCCGGTT CAACCCTTT- CGGTCGCAAA CCTTTCGGAG GACGTCCATT CGGCAGACGC AACCATACCG AAGAAGGATC TCCCAGGCGC GATGGC---- ---------- ----AACCGT GGACGTTGGG ATGAGAATGA 821**

**He185/333_cDNA_0073 GGACAGACCA GAGATGGGGC CATTCCGGTT CAACCCTTT- CGGTCGCAAA CCTTTCGGAG GACGTCCATT CGGCAGACGC AACCATACCG AAGAAGGATC TCCCAGGCGC GATGGC---- ---------- ----AACCGT GGACGTTGGG ATGAGAATGA 821**

**He185/333_cDNA_0074 GGACAGACCA GAGATGAGGC CATTCCGGTT CAACCCTTT- CGGTCGCAAG CCTTTCGGAG GACGTCCATT CGGCAGACGC AACCATACCG AAGAAGGATC TCCCAGGCGC GATGGC---- ---------- ----AACCGT GGACGTTGGG ATGAGAATGA 821**

**He185/333_cDNA_0075 GGACAGACCA GAGATGAGGC CATTCTGGTT CAACCCTTT- CGGTCGCAAG CCTTTCGGAG GACGTCCATT CGGCAGACGC AACCATACCG ATGAAGGATC TCCTAGGCGC GATGGA---- ---------- ----CACCGT GGACGTTGGA ATGAGAATGA 938**

**He185/333_cDNA_0076 GGACAGACCA GAGATGAGGC CATTCCGGTT CAACCCTTT- CGGTCGCAAA CCTTTCGGAA GACGTCAATT CGGCAGACGC AACCATACCA AAGAAGGATC TCCCAGGCGC GATGGC---- ---------- ----AACCGT GGACGTTGGG ATGAGAATGA 821**

**He185/333_cDNA_0077 GGACAGACCA GAGATGAGGC CATTCCGGTT CAACCCTTT- CGGTCGCAAG CCTTTCGGAG GACGTCCATT CGGCAGATGC AACCATACCG AAGAAGGATC TCCCAGGCGC GATGGAGATC GTCGTCCCAA GGGCAACCGT GGACGTTGGG ATGAGAATGA 812**

**He185/333_cDNA_0078 GGACAGACCA GAGATGAGGC CATTCCGGTT CAACCCTTT- CGGTCGCAAA CCTTTCGGAG GACGTCCATT CGGCAGACGC AACCATACCG AAGAAGGATC TCCCAGGCGC GATGGC---- ---------- ----AACCGT GGACGTTGGG ATGAGAATGA 821**

**He185/333_cDNA_0079 GGACAGACCA GAGATGAGGC CATTCCGGTT CAACCCTTT- CGGTCGCAAA CCTTTCGGAG GACGTCCATT CGGCAGACGC AACCATACCG AAGAAGGATC TCCCAGGCGC GATGGC---- ---------- ----AACCGT GGACGTTGGG ATGAGAATGA 821**

**He185/333_cDNA_0080 GGACAGACCA GAGATGAGGC CATTCCGGTT CAACCCTTT- CGGTCGCAAA CCTTTCGGAA GACGTCAATT CGGCAGACGC AACCATACCG AAGAAGGATC TCCCAGGCGC GATGGC---- ---------- ----AACCGT GGACGTTGGG ATGAGAATGA 821**

**He185/333_cDNA_0081 GGACAGACCA GAGATGAGGC CATTCCGGTT CAACCCTTT- CGGTCGCAAG CCTTTCGGAG GACATCCATT CGGCAGACGC AACCATACCG AAGAAGGATC TCCCAGGCGC GATGGAGATC GTCGTCCCAA TGGCAACCGT GGACGTTGGG ATGAGAATGA 809**

**He185/333_cDNA_0082 GGACAGACCA GAGATGAGGC CATTCCGGTT CAACCCTTT- CGGTCGCAAG CCTTTCGGAG GACGTCCATT CGGCAGATGC AACCATACCG AAGAAGGATC TCCCAGGCGC GATGGAGATC GTCGTCCCAA TGGCAACCGT GGACGTTGGG ATGAGAATGA 839**

**He185/333_cDNA_0083 GGACAGACCA GAGATGAGGC CATTCCGGTT CAACCCTTT- CGGTCGCAAG CCTTTCGGAG GACGTCCATT CGGCAGATGC AACCATACCG AAGAAGGATC TCCCAGGCGC GATGGAGATC GTCGTCCCAA TGGCAACCGT GGACGTTGGG ATGAGAATGA 809**

**He185/333_cDNA_0084 GGACAGACCA GAGATGAGGC CATTCCGGTT CAACCCTTT- CGGTCGCAAG CCTTTCGGAG GACGTCCATT CGGCAGATGC AACCATACCG AAGAAGGATC TCCCAGGCGC GATGGAGATC GTCGTCCCAA TGGCAACCGT GGACGTTGGG ATGAGAATGA 812**

**He185/333_cDNA_0085 GGACAGACCA GAGATGAGGC CATTCCGGTT CAACCCTTT- CGGTCGCAAG CCTTTCGGAG GACGTCCATT CGGCAGACGC AACCATACCG AAGAAGGATC TCCCAGGCGC GATGGAGATC GTCGTCCCAA TGGCAACCGT GGACGTTGGG ATGAGAATGA 848**

**He185/333_cDNA_0086 GGACAGACCA GAGATGAGGC CATTCCGGTT CAACCCTTT- CGGTCGCAAG CCTTTCGGAG GACGTCCATT CGGCAGATGC AACCATACCG AAGAAGGATC TCCCAGGCGC GATGGAGATC GTCGTCCCAA TGGCAACCGT GGACGTTGGG ATGAGAATGA 812**

**He185/333_cDNA_0087 GGACAGACCA GAGATGAGGC CATTCCGGTT CAACCCTTT- CGGTCGCAAG CCTTTCGGAG GACATCCATT CGGCAGACGC AACCATACCG AAGAAGGATC TCCCAGGCGC GATGGAGATC GTCGTCCCAA TGGCAACCGT GGACGTTGGG ATGAGAATGA 812**

**He185/333_cDNA_0088 GGACAGACCA GAGATGAGGC CATTCTGGTT CAACCCTTT- CGGTCGCAAG CCTTTCGGAG GACGTCCATT CGGCAGACGC AACCATACCG ATGAAGGATC TCCTAGGCGC GATGGA---- ---------- ----CACCGT GGACGTTGGA ATGAGAATGA 938**

**He185/333_cDNA_0089 GGACAGACCA GAGATGAGGC CATTCTGGTT CAACCCTTT- CGGTCGCAAG CCTTTCGGAG GACGTCCATT CGGCAGACGC AACCATACCG ATGAAGGATC TCCTAGGCGC GATGGA---- ---------- ----CACCGT GGACGTTGGA ATGAGAATGA 938**

**He185/333_cDNA_0090 GGACAGACCA GAGATGAGGC CATTCCGGTT CAACCCTTT- CGGTCGCAAG CCTTTCGGAG GACGTCCATT CGGCAGATGC AACCATACCG AAGAAGGATC TCCCAGGCGC GATGGAGATC GTCGTCCCAA TGGCAACCGT GGACGTTGGG ATGAGAATGA 812**

**He185/333_cDNA_0091 GGACAGACCA GAGATGAGGC CATTCCGGTT CAACCCTTT- CGGTCGCAAG CCTTTCGGAG GACGTCCATT CGGCAGATGC AACCATACCG AAGAAGGATC TCCCAGGCGC GATGGAGATC GTCGTCCCAA TGGCAACCGT GGACGTTGGG ATGAGAATGA 812**

**He185/333_cDNA_0092 GGACAGACCA GAGATGAGGC CATTCCGGTT CAACCCTTT- CGGTCGCAAG CCTTTCGGAG GACGTCCATT CGGCAGACGC AACCATACCG AAGAAGGATC TCCCAGGCGC GATGGAGATC GTCGTCCCAA TGGCAACCGT GGACGTTGGG ATGAGAATGA 848**

**He185/333_cDNA_0093 GGACAGACCA GAGATGAGGC CATTCCGGTT CAACCCTTT- CGGTCGCAAG CCTTTCGGAG GACGTCCATT CGGCAGACGC AACCATACCG AAGAAGGATC TCCCAGGCGC GATGGAGATC GTCGTCCCAA TGGCAACCGT GGACGTTGGG ATGAGAATGA 575**

**He185/333_cDNA_0094 GGACAGACCA GAGATGAGGC CATTCTGGTT CAACCCTTT- CGGTCGCAAG CCTTTCGGAG GACGTCCATT CGGCAGACGC AACCATACCG ATGAAGGATC TCCTAGGCGC GATGGA---- ---------- ----CACCGT GGACGTTGGA ATGAGAATGA 938**

**He185/333_cDNA_0095 GGACAGACCA GAGATGAGGC CATTCTGGTT CAACCCTTT- CGGTCGCAAG CCTTTCGGAG GACGTCCATT CGGCAGACGC AACCATACCG ATGAAGGATC TCCTAGGCGC GATGGA---- ---------- ----CACCGT GGACGTTGGA ATGAGAATGA 938**

**He185/333_cDNA_0096 GGACAGACCA GAGATGAGGC CATTCCGGTT CAACCCTTT- CGGTCGCAAG CCTTTCGGAG GACGTCCATT CGGCAGACGC AACCATACCG AAGAAGGATC TCCCAGGCGC GATGGAGATC GTCGTCCCAA TGGCAACCGT GGACGTTGGG ATGAGAATGA 812**

**He185/333_cDNA_0097 GGACAGACCA GAGATGAGGC CATTCCGGTT CAACCCTTT- CGGTCGCAAG CCTTTCGGAG GACGTCCATT CGGCAGATGC AACCATACCG AAGAAGGATC TCCCAGGCGC GATGGAGATC GTCGTCCCAA TGGCAACCGT GGACGTTGGG ATGAGAATGA 812**

**He185/333_cDNA_0098 GGACAGACCA GAGATGAGGC CATTCTGGTT CAACCCTTT- CGGTCGCAAG CCTTTCGGAG GACGTCCATT CGGCAGACGC AACCATACCG ATGAAGGATC TCCTAGGCGC GATGGA---- ---------- ----CACCGT GGACGTTGGA ATGAGAATGA 938**

**He185/333_cDNA_0099 GGACAGACCA GAGATGAGGC CATTCCGGTT CAACCCTTT- CGGTCGCAAG CCTTTCGGAG GACGTCCATT CGGCAGATGC AACCATACCG AAGAAGGATC TCCCAGGCGC GATGGAGATC GTCGTCCCAA TGGCAACCGT GGACGTTGGG ATGAGAATGA 812**

**He185/333_cDNA_0100 GGACAGACCA GAGATGAGGC CATTCCGGTT CAACCCTTT- CGGTCGCAAG CCTTTCGGAG GACATCCATT CGGCAGACGC AACCATACCG AAGAAGGATC TCCCAGGCGC GATGGAGATC GTCGTCCCAA TGGCAACCGT GGACGTTGGG ATGAGAATGA 809**

**He185/333_cDNA_0101 GGACAGACCA GAGATGAGGC CATTCCGGTT CAACCCTTT- CGGTCGCAAG CCTTTCGGAG GACGTCCATT CGGCAGATGC AACCATACCG AAGAAGGATC TCCCAGGCGC GATGGAGATC GTCGTCCCAA TGGCAACCGT GGACGTTGGG ATGAGAATGA 812**

**He185/333_cDNA_0102 GGACAGACCA GAGATGAGGC CATTCCGGTT CAACCCTTT- CGGTCGCAAG CCTTTCGGAG GACGTCCATT CGGCAGATGC AACCATACCG AAGAAGGATC TCCCAGGCGC GATGGAGATC GTCGTCCCAA TGGCAACCGT GGACGTTGGG ATGAGAATGA 809**

**He185/333_cDNA_0103 GGACAGACCA GAGATGAGGC CATTCTGGTT CAACCCTTT- CGGTCGCAAG CCTTTCGGAG GACGTCCATT CGGCAGACGC AACCATACCG ATGAAGGATC TCCTAGGCGC GATGGA---- ---------- ----CACCGT GGACGTTGGA ATGAGAATGA 938**

**He185/333_cDNA_0104 GGACAGACCA GAGATGAGGC CATTCCGGTT CAACCCTTT- CGGTCGCAAG CCTTTCGGAG GACGTCCATT CGGCAGATGC AACCATACCG AAGAAGGATC TCCCAGGCGC GATGGAGATC GTCGTCCCAA TGGCAACCGT GGACGTTGGG ATGAGAATGA 812**

**He185/333_cDNA_0105 GGACAGACCA GAGATGAGGC CATTCCGGTT CAACCCTTT- CGGTCGCAAG CCTTTCGGAG GACGTCCATT CGGCAGGCGC AACCATACCG AAGAAGGATC TCCCAGGCGC GATGGC----- ---------- ---AACCGT GGATGTTGGG ATGAGAATGA 693**

**He185/333_cDNA_0106 GGACAGACCA GAGATGAGGC CATTCCGGTT CAACCCTTT- CGGTCGCAAG CCTTTCGGAG GACGTCCATT CGGCAGACGC AACCATACCG AAGAAGGATC TCCCAGGCGC GATGCC----- ---------- ---AACCGT GGACGTTGGG ATGAGAATGA 780**

**He185/333_cDNA_0107 GGACAGACCA GAGATGAGGC CATTCCGGTT CAACCCTTT- CGGTCGCAAA CCTTTCGGAG GACGTCCATT CGGCAGACGC AACCATACCG AAGAAGGATC TCCCAGGCGC GATGGC----- ---------- ---AACCGT GGACGTTGGG ATGAGAATGA 525**

**He185/333_cDNA_0108 GGACAGACCA GAGATGAGGC CATTCCGGTT CAACCCTTT- CGGTCGCAAA CCTTTCGGAA GACGTCCATT CGGCAGACGC AACCATACCG AAGAAGGATC TCCCAGGCGC GATGGC----- ---------- ---AACCGT GGACGTTGGG ATGAGAATGA 820**

**He185/333_cDNA_0109 GGACAGACCA GAGATGAGGC CATTCCGGTT CAACCCTTT- CGGTCGCAAG CCTTTCGGAG GACGTCCATT CGGCAGATGC AACCATACCG AAGAAGGATC TCCCAGGCGC GATGGAGATC GTCGTCCCAA GGGCAACCGT GGACGTTGGG ATGAGAATGA 520**

**He185/333_cDNA_0110 GGACAGACCA GAGATGAGGC CATTCCGGTT CAACCCTTT- CGGTCGCAAA CCTTTCGGAG GACGTCCATT CGGCAGACGC AACCATACCG AAGAAGGATC TCCCAGGCGC GATGGC----- ---------- ---AACCGT GGACGTTGGG ATGAGAATGA 820**

**He185/333_cDNA_0111 GGACAGACCA GAGATGAGGC CATTCCGGTT CAACCCTTTT CGGTCGCAAA CCTTTCGGAG GACGTCCATT CGGCAGACGC AACCATACCG AAGAAGGATC TCCCAGGCGC GATGGC----- ---------- ---AACCGT GGACGTTGGG ATGAGAATGA 699**

**He185/333_cDNA_0112 GGACAGACCA GAGATGAGGC CATTCCGGTT CAACCCTTT- CGGTCGCAAA CCTTTCGGAG GACGTCCATT CGGCAGACGC AACCATACCG AAGAAGGATC TCCCAGGCGC GATGGC----- ---------- ---AACCGT GGACGTTGGG ATGAGAATGA 733**

28

27

**....|....| ....|....| ....|....| ....|....| ....|....| ....|....| ....|....| ....|....| ....|....| ....|....| ....|....| ....|....| ....|..**

31

30

29

**965 975 985 995 1005 1015 1025 1035 1045 1055 1065 1075 1085**

**He185/333_cDNA_0001 AAGT------- --------G TGGAGGAAGA ACATCTTCCG ACTGAAAGCA TGACAACATC TGCAGTGCCT GATGTGGTCG AGATCGACAT CAACGAAATA GACAGCAACA TTATCCCCGA GGTGTAG 654**

**He185/333_cDNA_0002 AAGT------- --------G TGGAGGAAGA ACATCTTCCG ACTGAAAGCA TGACAACATC TGTAGTGCCT GATGTGGTCG AGATCGACAT CAACGAAATA GACAGCAACA TTATCCCCGA GGTGTAG 933**

**He185/333_cDNA_0003 AAGT------- --------G TGGAGGAAGA ACATCTTCCG ACTGAAAGCA TGACAACATC TGTAGTGCCT GATGTGGTCG AGATCGACAT CAACGAAATA GACAGCAACA TTATCCCCGA GGTGTAG 933**

**He185/333_cDNA_0004 AAGT------- --------G TGGAGGAAGA ACATCTTCCG ACTGAAAGCA TGACAACATC TGCAGTGCCT GATGTGGTCG AGATCGACAT CAACGAAATA GACAGCAACA TTATCCCCGA GGTGTAG 930**

**He185/333_cDNA_0005 AAGT------- --------G TGGAGGAAGA ACATCTTCCG ACTGAAAGCA TGACAACATC TGTAGTGCCT GATGTGGTCG AGATCGACAT CAACGAAATA GACAGCAACA TTATCCCCGA GGTGTAG 933**

**He185/333_cDNA_0006 AAGT------- --------G TGGAGGAAGA ACATCTTCCG ACTGAAAGCA TGACAACATC TGTAGTGCCT GATGTGGTCG AGATCGACAT CAACGAAATA GACAGCAACA TTATCCCCGA GGTGTAG 729**

**He185/333_cDNA_0007 AAGT------- --------G TGGAGGAAGA ACATCTTCCG ACTGAAAGCA TGACAACATC TGTAGTGCCT GATGTGGTCG AGATCGACAT CAACGAAATA GACAGCAACA TTATCCCCGA GGTGTAG 933**

**He185/333_cDNA_0008 AAGT------- --------G TGGAGGAAGA ACATCTTCCG ACTGAAAGCA TGACAACATC TGCAGTGCCT GATGTGGTCG AGATCGACAT CAACGAAATA GACAGCAACA TTATCCCCGA GGTGTAG 933**

**He185/333_cDNA_0009 AAGT------- --------G TGGAGGAAGA ACATCTTCCG ACTGAAAGCA TGACAACATC TGTAGTGCCT GATGTGGTCG AGATCGACAT CAACGAAATA GACAGCAACA TTATCCCCGA GGTGTAG 933**

**He185/333_cDNA_0010 AAGT------- --------G TGGAGGAAGA ACATCTTCCG ACTGAAAGCA TGACAACATC TGTAGTGCCT GATGTGGTCG AGATCAACAT CAACGAAATA GACAGCAACA TTATCCCCGA GGTGTAG 933**

**He185/333_cDNA_0011 AAGT------- --------G TGGAGGAAGA ACATCTTCCG ACTGAAAGCA TGACAACATC TGTAGTGCCT GATGTGGTCG AGATCGACAT CAACGAAATA GACAGCAACA TTATCCCCGA GGTGTAG 933**

**He185/333_cDNA_0012 AAGT------- --------G AGGAGGAAGA ACATCTTCCA ACTGAAAGCA TGACAACATC TGCAGTGCCT GATGTGGTCG AGATTGACAT CAAC------ ---------A TTATCCCCGA GGTGTAG 909**

**He185/333_cDNA_0013 AAGT------- --------G TGGAGGAAGA ACATCTTCCG ACTGAAAGCA TGACAACATC TGCAGTGCCT GATGTGGTCG AGATCGACAT CAACGAAATA GACAGCAACA TTATCCCCGA GGTGTAG 933**

**He185/333_cDNA_0014 AAGT------- --------G AGGAGGAAGA ACATCTTCCA ACTGAAAGCA TGACAACATC TGCAGTGCCT GATGTGGTCG AGATTGACAT CAAC------ ---------A TTATCCCCGA GGTGTAG 909**

**He185/333_cDNA_0015 AAGT------- --------G AGGAGGAAGA ACATCTTCCA ACTGAAAGCA TGACAACATC TGCAGTGCCT GATGTGGTCG AGATTGACAT CAAC------ ---------A TTATCCCCGA GGTGTAG 909**

**He185/333_cDNA_0016 AAGGGATGGG AAAGAAAATG AGGAGGAAGA ACATCTTCCA ACAGAAAGCA TGACAACATG TGCAGTGATT GATGTGATCG AAATTGACAT CAAC------ ---------A TTATCCCCGA GGTGTAG 909**

**He185/333_cDNA_0017 AAGT------- --------G AGGAGGAAGA ACATCCTCCA ACTGAAAGCA TGACAATATC CGCAGTGCCT GATGTGGTCG AGATCGACAT CAACGAAATA GACATCAACA TTATCCCCGA GGAGTAG 870**

**He185/333_cDNA_0018 AAGT------- --------G TGGAGGAAGA ACATCTTCCG ACTGAAAGCA TGACAACATC TGTAGTGCCT GATGTGGTCG AGATCGACAT CAACGAAATA GACAGCAACA TTACCCCCGA GGTGTAG 933**

**He185/333_cDNA_0019 AAGT------- --------G TGGAGGAAGA ACATCTTCCG ACTGAAAGCA TGACAACATC TGTAGTGCCT GATGTGGTCG AGATCGACAT CAACGAAATA GACAGCAACA TTATCCCCGA GGTGTAG 933**

**He185/333_cDNA_0020 AAGT------- --------G TGGAGGAAGA ACATCTTCCG ACTGAAAGCA TGACAACATC TGCAGTGCCT GATGTGGTCG AGATCGACAT CAACGAAATA GACAGCAACA TTATCCCCGA GGTGTAG 891**

**He185/333_cDNA_0021 AAGT------- --------G TGGAGGAAGA ACATCTTCCG ACTGAAAGCA TGACAACATC TGTAGTGCCT GATGTGGTCG AGATCGACAT CAACGAAATA GACAGCAACA TTATCCCCGA GGTGTAG 933**

**He185/333_cDNA_0022 ATGT------- --------G AGGAGGAAGA ACATCTTCCA ACTGAAAGCA TGACAACACC TGCAGTGCCT GATGTGGTCG AGATTGACAT CAAC------ ---------A TTATCCCCGA GGTGTAG 219**

**He185/333_cDNA_0023 AAGT------- --------G AGGAGGAAGA ACATCTTCCA ACTGAAAGCA TGACAACATC TGTAGTGCCT GATGTGGTCG AGATTGACAT CAAC------ ---------A TTATCCCCGA GGTGTAG 906**

**He185/333_cDNA_0024 AAGT------- --------G AGGAGGAAGA ACATCTTCCA ACTGAAAGCA TGACAACATC TGCAGTGCCT GATGTGGTCG AGATTGACAT CAAC------ ---------A TTATCCCCGA GGTGTAG 837**

**He185/333_cDNA_0025 AAGT------- --------G AGGAGGAAGA ACATCTTCCA ACTGAAAGCA TGACGACATC TGTAGTGCCT GATGTGGTCG AGATTGACAT CAAC------ ---------A TTATCCCCGA GGTGTAG 906**

**He185/333_cDNA_0026 AAGT------- --------G AGGAGGAAGA ACATCTTCCA ACTGAAAGCA TGACAACATC TGCAGTGCCT GATGTGGTCG AGATTGACAT CAAC------ ---------A TTATCCCCGA GGTGTAG 909**

**He185/333_cDNA_0027 AAGT------- --------G AGGAGGAAGA ACATCTTCCA ACTGAAAGCA TGACAACATC TGTAGTGCCT GATGTGGTCG AGATTGACAT CAAC------ ---------A TTATCCCCGA GGTGTAG 906**

**He185/333_cDNA_0028 AAGT------- --------G AGGAGGAAGA ACATCTTCCA ACTGAAAGCA TGACAACATC TGCAGTGCCT GATGTGGTCG AGATTGACAT GAAC------ ---------A TTATCCCCGA GGTGTAG 804**

**He185/333_cDNA_0029 AAGT------- --------G AGGAGGAAGA ACATCTTCCA ACTGAAAGCA TGACAACATC TGTAGTGCCT GATGTGGTCG AGATTGACAT CAAC------ ---------A TTATCCCCGA GGTGTAG 906**

**He185/333_cDNA_0030 AAGT------- --------G AGGAGGAAGA ACATCTTCCA ACTGAAAGCA TGACAACATC TGCAGTGCCT GACGTGGTCG AGATTGACAT CAAC------ ---------A TTATCCCCGA GGTGTAG 909**

**He185/333_cDNA_0031 AAGT------- --------G AGGAGGAAGA ACATCTTCCA ACTGAAAGCA TGACAACATC TGTAGTGCCT GATGTGGTCG AGATTGACAT CAAC------ ---------A TTATCCCCGA GGTGTAG 906**

**He185/333_cDNA_0032 AAGT------- --------G AGGAGGAAGA ACATCTTCCA ACTGAAAGCA TGACAACATC TGCAGTGCCT GATGTGGTCG AGATTGACAT CAAC------ ---------A TTATCCCCGA GGTGTAG 804**

**He185/333_cDNA_0033 AAGT------- --------G AGGAGGAAGA ACATCTTCCA ACTGAAAGCA TGACAACATC TGTAGTGCCT GATGTGGTCG AGATTGACAT CAAC------ ---------A TTATCCCCGA GGTGTAG 903**

**He185/333_cDNA_0034 AAGT------- --------G AGGAGGAAGA ACATCTTCCA ACTGAAAGCA TGACGACATC TGTAGTGCCT GATGTGGTCG AGATTGACAT CAAC------ ---------A TTATCCCCGA GGTGTAG 906**

**He185/333_cDNA_0035 AAGT------- --------G AGGAGGAAGA ACATCTTCCA ACTGAAAGCA TGACAACATC TGCAGTGCCT GATGTGGTCG AGATCGACAT CAACGAAATA GACATCAACA TTATCCCCGA GGTGTAG 942**

**He185/333_cDNA_0036 AAGT------- --------G AGGAGGAAGA ACATCTTCCA ACTGAAAGCA TGACAATATC CGCAGTGCCT GATGTGGTCG AGATCGACAT CAACGAAATA GACATCAACA TTATCCCCGA GGTGTAG 1050**

**He185/333_cDNA_0037 AAGT------- --------G AGGAGGAAGA ACATCTTCCA ACTGAAAGCA TGACAACATC TGCAGTGCCT GATGTGGTCG AGATTGACAT CAAC------ ---------A TTATCCCCGA GGTGTAG 912**

**He185/333_cDNA_0038 AAGT------- --------G TGGAGGAAGA ACATCTTCCG ACTGAAAGCA TGACAACATC TGTAGTGCCT GATGTGGTCG AGATCGACAT CAACGAAATA GACAGCAACA TTATCCCCGA GGTGTAG 933**

**He185/333_cDNA_0039 AAGT------- --------G TGGAGGAAGA ACATCTTCCG ACTGAAAGCA TGACAACATC TGTAGTGCCT GATGTGGTCG AGATCGACAT CAACGAAATA GACAGCAACA TTATCCCCGA GGTGTAG 933**

**He185/333_cDNA_0040 AAGT------- --------G AGGAGGAAGA ACATCTTCCG ACTGAAAGCA TGACAACATC TGCAGTGCCT GATGTGGTCG AGATCGACAT CAACGAAATA GACAGCAACA TTATCCCCGA GGTGTAG 942**

**He185/333_cDNA_0041 AAGT------- --------G TGGAGGAAGA ACATCTTCCG ACTGAAGGCA TGACAACATC TGTAGTGCCT GATGTGGTCG AGATCAACAT CAACGAAATA GACAGCAACA TTATCCCCGA GGTGTAG 933**

**He185/333_cDNA_0042 AGGT------- --------G TGGAGGAAGA ACATCTTCCG ACTGGAAGCA TGACAACATC TGTAGTGCCT GATGTGGTCG AGATCGACAT CAACGAAATA GACAGCAACA TTATCCCCGA GGTGTAG 933**

**He185/333_cDNA_0043 AAGT------- --------G AGGAGGAAGA ACATCTTCCA ACTGAAAGCA TGACAACATC TGTAGTGCCT GATGTGGTCG AGATCGACAT CAACGAAATA GACAGCAACA TTATCCCCGG GGTGTAG 921**

**He185/333_cDNA_0044 AAGT------- --------G TGGAGGAAGA ACATCTTCCG ACTGAAAGCA TGACAACATC TGCAGTGCCT GATGTGGTCG AGATCGACAT CAACGAAATA GACAGCAACA TTATCCCCGA GGTGTAG 933**

**He185/333_cDNA_0045 AAGT------- --------G AGGAGGAAGA ACATCTTCCG ACTGAAAGCA TGACAACATC TGCAGTGCCT GATGTGGTCG AGATCGACAT CAACGAAATA GACAGCAACA TTATCCCCGA GGTGTAG 942**

**He185/333_cDNA_0046 AAGT------- --------G AGGAGGAAGA ACATCTTCCG ACTGAAAGCA TGACAACATC TGCAGTGCCT GATGTGGTCG AGATCGACAT CAACGAAATA GACAGCAACA TTATCCCCGA GGTGTAG 942**

**He185/333_cDNA_0047 AAGT------- --------G AGGAGGAAGA ACATCTTCCA ACTGAAAGCA TGACAACATC TGCAGTGCCT GATGTGGTCG AGATTGACAT CAAC------ ---------A TTATCCCCGA GGTGTAG 909**

**He185/333_cDNA_0048 AAGT------- --------G TGGAGGAAGA ACATCTTCCG ACTGAAAGCA TGACAACATC TGCAGTGCCT GATGTGGTCG AGATCGACAT CAACGAAATA GACAGCAACA TTATCCCCGA GGTGTAG 933**

**He185/333_cDNA_0049 AAGT------- --------G TGGAGGAAGA ACATCTTCCG GCTGAAAGCA TGACAACATC TGCAGTGCCT GATGTGGTCG AGATCGACAT CAACGAAATA GACAGCAACA TTATCCCCGA GGTGTAG 933**

**He185/333_cDNA_0050 AAGT------- --------G TGGAGGAAGA ACATCTTCCG ACTGAAAGCA TGACAACATC TGCAGTGCCT GATGTGGTCG AGATCGACAT CAACGAAATA GACAGCAACA TTATCCCCGA GGTGTAG 933**

**He185/333_cDNA_0051 AAGT------- --------G AGGAGGAAGA ACATCTTCCG ACTGAAAGCA TGACAACATC TGCAGTGCCT GATGTGGTCG AGATCGACAT CAACGAAATA GACAGCAACA TTATCCCCGA GGTGTAG 942**

**He185/333_cDNA_0052 AAGT------- --------G TGGAGGAAGA ACATCTTCCG ACTGAAAGCA TGACAACATC TGCAGTGCCT GATGTGGTCG AGATCGACAT CAACGAAATA GACAGCAACA TTATCCCCGA GGTGTAG 816**

**He185/333_cDNA_0053 AAGT------- --------G AGGAGGAAGA ACATCTTCCG ACTGAAAGCA TGACAACATC TGCAGTGCCT GATGTGGTCG AGATCGACAT CAACGAAATA GACAGCAACA TTATCCCCGA GGTGTAG 915**

**He185/333_cDNA_0054 AAGT------- --------G AGGAGGAAGA ACATCTTCCA ACTGAAAGCA TGACAACATC TGTAGTGCCT GATGTGGTCG AGATTGACAT CAAC------ ---------A TTATCCCCGA GGTGTAG 906**

**He185/333_cDNA_0055 AAGT------- --------G AGGAGGAAGA ACATCTTCCA ACTGAAAGCA TGACAACATC TGCAGTGCCT GATGTGGTCG AGATTGACAT CAAC------ ---------A TTATCCCCGA GGTGTAG 909**

**He185/333_cDNA_0056 AAGT------- --------G AGGAGGAAGA ACATCTTCCA ACTGAAAGCA TGACAACATA TGCAGTACCT GATGTGGTCG AGATTGACAT CGAC------ ---------A TTGTCCCCGA GGGGTAG 909**

**He185/333_cDNA_0057 AAGT------- --------G AGGAGGAAGA ACATCTTCCA ACTGAAAGCA TGACAATATC CGCAGTGCCT GATGTGGTCG AGATCGACAT CAACGAAATA GACATCAACA TTATCCCCGA GGTGTAG 1050**

**He185/333_cDNA_0058 AAGT------- --------G AGGAGGAAGA ACATCTTCCA ACTGAAAGCA TGACAATATC CGCAGTGCCT GATGTGGTCG AGATCGACAT CAAC------ ---------- --------GA GGTGTAG 441**

**He185/333_cDNA_0059 AAGT------- --------G AGGAGGAAGA ACATCTTCCA ACTGAAAGCA TGACAATATC CGCAGTGCCT GATGTGGTCG AGATCGACAT CAACGAAATA GACATCAACA TTATCCCCGA GGTGTAG 936**

**He185/333_cDNA_0060 AAGT------- --------G AGGAGGAAGA ACATCTTCCA ACTGAAAGCA TGACAACATC TGCAGTGCCT GATGTGGTCG AGATCGACAT CAACGAAATA GACATCAACA TTATCCCCGA GGTGTAG 942**

**He185/333_cDNA_0061 AAGT------- --------G AGGAGGAAGA ACATCTTCCA ACTGAAAGCA TGACAATATC CGCAGTGCCT GATGTGGTCG AGATCGACAT CAACGAAATA GACATCAACA TTATCCCCGA GGTGTAG 1050**

**He185/333_cDNA_0062 AAGT------- --------G AGGAGGAAGA ACATCTTCCA ACTGAAAGCA TGACAATATC CGCAGTGCCT GATGTGGTCG AGATCGACAT CAACGAAATA GACATCAACA TTATCCCCGA GGTGTAG 774**

**He185/333_cDNA_0063 AAGT------- --------G AGGAGGAAGA ACATCTTCCA ACTGAAAGTA TGACAATATC CGCAGTGCCT GATGTGGTTG AGATCGACAT CAACGAAATA GACATCAACA TTATCCCCGA GGTGTAG 1050**

**He185/333_cDNA_0064 AAGT------- --------G AGGAGGAAGA ACATCTTCCA ACTGAAAGCA TGACAATATC CGCAGTGCCT GATGTGGTCG AGATCGACAT CAACGAAATA GACATCAACA TTATCCCCGA GGTGTAG 1050**

**He185/333_cDNA_0065 AAGT------- --------G AGGAGGAAGA ACATCTTCCA ACTGAAAGCA TGACAATATC CGCAGTGCCT GATGTGGTCG AGATCGACAT CAACGAAATA GACATCAACA TTATCCCCGA GGTGTAG 978**

**He185/333_cDNA_0066 AAGT------- --------G AGGAGGAAGA ACATCTTCCA ACTGAAAGCA TGACAATATC CGCAGTGCCT GATGTGGTCG AGATCGACAT CAACGAAATA GACATCAACA TTATCCCCGA GGTGTAG 981**

**He185/333_cDNA_0067 AAGC------- --------G AGGAGGAAGA ACATCTTCCA ACTGAAAGCA TGACAATATC CGCAGTGCCT GATGTGGTCG AGATCGACAT CAACGAAATA GACATCAACA TTGTCGCCGA GGTGTAG 861**

**....|....| ....|....| ....|....| ....|....| ....|....| ....|....| ....|....| ....|....| ....|....| ....|....| ....|....| ....|....| ....|..**

31

30

29

28

27

**965 975 985 995 1005 1015 1025 1035 1045 1055 1065 1075 1085**

**He185/333_cDNA_0068 AAGT------- --------G AGGAGGAAGA ACATCTTCCG ACTGAAAGTA TGACAACATC TGCAGTGCCT GATGTGGTCG AGATCGACAT CAACGAAATA GACAGCAACA TTGTCGCCGA GGTGTAG 942**

**He185/333_cDNA_0069 AAGT------- --------G TGGAGGAAGA ACATCTTCCG ACTGAAAGCA TGACAACATC TGTAGTGCCT GATGTGGTCG AGATCGACAT CAACGAAATA GACAGCAACA TTGTCGCCGA GGTGTAG 933**

**He185/333_cDNA_0070 AAGT------- --------G TGGAGGAAGA ACATCTTCCG ACTGAAAGCA TGACAACATC TGCAGTGCCT GATGTGGTCG AGATCGACAT CAACGAAATA GACAGCAACA TTGTCGCCGA GGTGTAG 933**

**He185/333_cDNA_0071 AAGT------- --------G TGGAGGAAGA ACATCTTCCG ACTGAAAGCA TGACAACATC TGCAGTGCCT GATGTGGTCG AGATCGACAT CAACGAAATA GACAGCAACA TTGTCGCCGA GGTGTAG 933**

**He185/333_cDNA_0072 AAGT------- --------G TGGAGGAAGA ACATCTTCCG ACTGAAAGCA TGACAACATC TGCAGTGCCT GATGTGGTCG AGATCGACAT CAACGAAATA GACAGCAACA TTGTCGCCGA GGTGTAG 933**

**He185/333_cDNA_0073 AAGT------- --------G TGGAGGAAGA ACATCTTCCG ACTGAAAGCA TGACAACATC TGCAGTGCCT GATGTGGTCG AGATCGACAT CAACGAAATA GACAGCAACA TTGTCGCCGA GGTGTAG 933**

**He185/333_cDNA_0074 AAGT------- --------G TGGAGGAAGA ACATCTTCCG ACTGAAAGCA TGACAACATC TGCAGTGCCT GATGTGGTCG AGATCGACAT CAACGAAATA GACAGCAACA TTGTCGCCGA GGTGTAG 933**

**He185/333_cDNA_0075 AAGT------- --------G AGGGGGAAGA ACATCTTCCA ACTGAAAGCA TGACAATATC CGCAGTGCCT GATGTGGTCG AGATCGACAT CAACGAAATA GACATCAACA TTGTCGCCGA GGTGTAG 1050**

**He185/333_cDNA_0076 AAGT------- --------G TGGAGGAAGA ACATCTTCCG ACTGAAAGCA TGACAACATC TGTAGTGCCT GATGTGGTCG AGATCGACAT CAACGAAATA GACAGCAACA TTGTCGCCGA GGTGTAG 933**

**He185/333_cDNA_0077 AAGT------- --------G AGGAGGAAGA ACATCTTCCA ACTGAAAGCA TGACAACATC TGCAGTGCCT GATGTGGTCG AGATTGACAT CAAC------ ---------A TTGTCGCCGA GGTGTAG 909**

**He185/333_cDNA_0078 AAGT------- --------G TGGAGGAAGA ACATCTTCCG ACTGAAAGCA TGACAACATC TGCAGTGCCT GATGTGGTCG AGATCGACAT CAACGAAATA GACAGCAACA TTGTCGCCGA GGTGTAG 933**

**He185/333_cDNA_0079 AAGT------- --------G TGGAGGAAGA ACATCTTCCG ACTGAAAGCA TGACAACATC TGCAGTGCCT GATGTGGTCG AGATCGACAT CAACGAAATA GACAGCAACA TTGTCGCCGA GGTGTAG 933**

**He185/333_cDNA_0080 AAGT------- --------G TGGAGGAAGA ACATCTTCCG ACTGAAAGCA TGACAACATC TGTAGTGCCT GATGTGGTCG AGATCGACAT CAACGAAATA GACAGCAACA TTGTCGCCGA GGTGTAG 933**

**He185/333_cDNA_0081 AAGT------- --------G AGGAGGAAGA ACATCTTCCA ACTGAAAGCA TGACAACATC TGTAGTGCCT GATGTGGTCG AGATTGACAT CAAC------ ---------A TTATCCCCGA GGTGTAG 906**

**He185/333_cDNA_0082 AAGT------- --------G AGGAGGAAGA ACATCTTCCA ACTGAAAGCA TGACAACATC TGCAGTGCCT GATGTGGTCG AGATTGACAT CAAC------ ---------A TTATCCCCGA GGTGTAG 936**

**He185/333_cDNA_0083 AAGT------- --------G AGGAGGAAGA ACATCTTCCA ACTGAAAGCA TGACAACATC TGCAGTGCCT GATGTGGTCG AGATTGACAT CAAC------ ---------A TTATCCCCGA GGTGTAG 906**

**He185/333_cDNA_0084 AAGT------- --------G AGGAGGAAGA ACATCTTCCA ACTGAAAGCA TGACAACATC TGCAGTGCCT GATGTGGTCG AGATTGACAT CAAC------ ---------A TTATCCCCGA GGTGTAG 909**

**He185/333_cDNA_0085 AAGT------- --------G TGGAGGAAGA ACATCTTCCG ACTGAAAGCA TGACAACATC TGCAGTGCCT GATGTGGTCG AGATCGACAT CAACGAAATA GACATCAACA TTATCCCCGA GGTGTAG 960**

**He185/333_cDNA_0086 AAGT------- --------G AGGAGGAAGA ACATCTTCCA ACTGAAAGCA TGACAACATC TGCAGTGCCT GATGTGGTCG AGATTGACAT CAAC------ ---------A TTATCCCCGA GGTGTAG 909**

**He185/333_cDNA_0087 AAGT------- --------G AGGAGGAAGA ACATCTTCCA ACTGAAAGCA TGACAACATC TGTAGTGCCT GATGTGGTCG AGATTGACAT CAAC------ ---------A TTATCCCCGA GGTGTAG 909**

**He185/333_cDNA_0088 AAGT------- --------G AGGAGGAAGA ACATCTTCCA ACTGAAAGCA TGACAATATC CGCAGTGCCT GATGTGGTCG AGATCGACAT CAACGAAATA GACATCAACA TTATCCCCGA GGTGTAG 1050**

**He185/333_cDNA_0089 AAGT------- --------G AGGAGGAAGA ACATCTTCCA ACTGAAAGCA TGACAATATC CGCAGTGCCT GATGTGGTCG AGATCGACAT CAACGAAATA GACATCAACA TTATCCCCGA GGTGTAG 1050**

**He185/333_cDNA_0090 AAGT------- --------G AGGAGGAAGA ACATCTTCCA ACTGAAAGCA TGACAACATC TGCAGTGCCT GATGTGGTCG AGATTGACAT CAAC------ ---------A TTATCCCCGA GGTGTAG 909**

**He185/333_cDNA_0091 AAGT------- --------G AGGAGGAAGA ACATCTTCCA ACTGAAAGCA TGACAACATC TGCAGTGCCT GATGTGGTCG AGATTGACAT CAAC------ ---------A TTATCCCCGA GGTGTAG 909**

**He185/333_cDNA_0092 AAGT------- --------G TGGAGGAAGA ACATCTTCCG ACTGAAAGCA TGACAACATC TGCAGTGCCT GATGTGGTCG AGATCGACAT CAACGAAATA GACATCAACA TTATCCCCGA GGTGTAG 960**

**He185/333_cDNA_0093 AAGT------- --------G TGGAGGAAGA ACATCTTCCG ACTGAAAGCA TGACAACATC TGCAGTGCCT GATGTGGTCG AGATCGACAT CAACGAAATA GACATCAACA TTATCCCCGA GGTGTAG 687**

**He185/333_cDNA_0094 AAGT------- --------G AGGAGGAAGA ACATCTTCCA ACTGAAAGCA TGACAATATC CGCAGTGCCT GATGTGGTCG AGATCGACAT CAACGAAATA GACATCAACA TTATCCCCGA GGTGTAG 1050**

**He185/333_cDNA_0095 AAGT------- --------G AGGAGGAAGA ACATCTTCCA ACTGAAAGCA TGACAATATC CGCAGTGCCT GATGTGGTCG AGATCGACAT CAACGAAATA GACATCAACA TTATCCCCGA GGTGTAG 1050**

**He185/333_cDNA_0096 AAGT------- --------G TGGAGGAAGA ACATCTTCCG ACTGAAAGCA TGACAACATC TGCAGTGCCT GATGTGGTCG AGATCGACAT CAACGAAATA GACATCAACA TTATCCCCGA GGTGTAG 924**

**He185/333_cDNA_0097 AAGT------- --------G AGGAGGAAGA ACATCTTCCA ACTGAAAGCA TGACAACATC TGCAGTGCCT GATGTGGTCG AGATTGACAT CAAC------ ---------A TTATCCCCGA GGTGTAG 909**

**He185/333_cDNA_0098 AAGT------- --------G AGGAGGAAGA ACATCTTCCA ACTGAAAGCA TGACAATATC CGCAGTGCCT GATGTGGTCG AGATCGACAT CAACGAAATA GACATCAACA TTATCCCCGA GGTGTAG 1050**

**He185/333_cDNA_0099 AAGT------- --------G AGGAGGAAGA ACATCTTCCA ACTGAAAGCA TGACAACATC AGCAGTGCCT GATGTGGTCG AGATTGACAT CAAC------ ---------A TTATCCCCGA GGTGTAG 909**

**He185/333_cDNA_0100 AAGT------- --------G AGGAGGAAGA ACATCTTCCA ACTGAAAGCA TGACAACATC TGTAGTGCCT GATGTGGTCG AGATTGACAT CAAC------ ---------A TTATCCCCGA GGTGTAG 906**

**He185/333_cDNA_0101 AAGT------- --------G AGGAGGAAGA ACATCTTCCA ACTGAAAGCA TGACAATATC CGCAGTGCCT GATGTGGTCG AGATCGACAT CAACGAAATA GACATCAACA TTATCCCCGA GGTGTAG 924**

**He185/333_cDNA_0102 AAGT------- --------G AGGAGGAAGA ACATCTTCCA ACTGAAAGCA TGACAACATC TGCAGTGCCT GATGTGGTCG AGATTGACAT CAAC------ ---------A TTATCCCCGA GGTGTAG 906**

**He185/333_cDNA_0103 AAGT------- --------G AGGAGGAAGA ACATCTTCCA ACTGAAAGCA TGACAATATC CGCAGTGCCT GATGTGGTCG AGATCGACAT CAACGAAATA GACATCAACA TTATCCCCGA GGTGTAG 1050**

**He185/333_cDNA_0104 AAGT------- --------G AGGAGGAAGA ACATCTTCCA ACTGAAAGCA TGACAACATC TGCAGTGCCT GATGTGGTCG AGATTGACAT CAAC------ ---------A TTATCCCCGA GGTGTAG 909**

**He185/333_cDNA_0105 AAGT------- --------G TGGAGGAAGA ACATCTTCCG ACTGAAAGCA TGACAACATC TGCAGTGCCT GATGTGGTCG AGATCGACAT CAACGAAATA GACAGCAACA TTATCCCCGA GGTGTAG 805**

**He185/333_cDNA_0106 AAGT------- --------G TGGAGGAAGA ACATCTTCCG ACTGAAAGCA TGACAACATC TGCAGTGCCT GATGTGGTCG AGATCGACAT CAACGAAATA GACAGCAACA TTATCCCCGA GGTGTAG 892**

**He185/333_cDNA_0107 AAGT------- --------G TGGAGGAAGA ACATCTTCCG ACTGAAAGCA TGACAACATC TGCAGTGCCT GATGTGGTCG AGATCGACAT CAACGAAATA GACAGCAACA TTATCCCCGA GGTGTAG 637**

**He185/333_cDNA_0108 AAGT------- --------G TGGAGGAAGA ACATCTTCCG ACTGAAAGCA TGACAACATC TGTAGTGCCT GATGTGGTCG AGATCGACAT CAACGAAATA GACAGCAACA TTATCCCCGA GGTGTAG 932**

**He185/333_cDNA_0109 AAGT------- --------G AGGAGGAAGA ACATCTTCCA ACTGAAAGCA TGACAACATC TGCAGTGCCT GATGTGGTCG AGATTGACAT CAAC------ ---------A TTATCCCCGA GGTGTAG 617**

**He185/333_cDNA_0110 AAGT------- --------G TGGAGGAAGA ACATCTTCCG ACTGAAAGCA TGACAACATC TGCAGTGCCT GATGTGGTTG AGATCGACAT CAACGAAATA GACAGCAACA TTATCCCCGA GGTGTAG 932**

**He185/333_cDNA_0111 AAGT------- --------G TGGAGGAAGA ACATCTTCCG ACTGAAAGCA TGACAACATC TGCAGTGCCT GATGTGGTCG AGATCGACAT CAACGAAATA GACAGCAACA TTATCCCCGA GGTGTAG 811**

**He185/333_cDNA_0112 AAGT------- --------G TGGAGGAAGA ACATCTTCCG ACTGAAAGCA TGACAACATC TGCAGTGCCT GATGTGGTCG AGATCGACAT CAACGAAATA GACGGCAACA TTATCCCCGA GGTGTAG 845**
