# Supplementary material for: Characterization of the Highly Variable Immune Response Gene Family, He185/333, in the Sea Urchin, Heliocidaris erythrogramma
Source: PLoS One. 2014 Oct 21;9(10):e62079. doi: 10.1371/journal.pone.0062079 (PMC4204807; doi:10.1371/journal.pone.0062079)
Supplement: Figure S3 — Nucleotide sequence alignment for 39 He185/333 gDNAs generated in Clustal W and BioEdit. The untranslated regions (5′UTR and 3′UTR), leader, 26 exon elements and ten intron elements (with suffix “i”) are labeled along the top and separated by vertical black lines. The start and stop codons are shaded in green and red coloured boxes, respectively and labeled along the top. The exon elements have been numbered according to the categorization system based on the 112 cDNA sequences shown in Fig. S2. The leader, which is identical to the predicted signal sequence, counts 63 nucleotides but is separated by the intron between nucleotide positions 55 and 56. (DOC) [file pone.0062079.s003.doc]

5’UTR

Leader

i1

i2

Start

**....|....| ....|....| ....|....| ....|....| ....|....| ....|....| ....|....| ....|....| ....|....| ....|....| ....|....| ....|....| ....|....| ....|....| ....|....| ....|....|**

**5 15 25 35 45 55 65 75 85 95 105 115 125 135 145 155**

**He185/333_gDNA_0001 GGGAAAGGAT AGAATTGGAG AGACCATTCA GACATGGAGT TGAAAGTGAC ACTGATCGTT ACCCTTGTGG CTGCTATTAC CATCTCAGGT AAGAAGTATT ATTATTT-GG TATTATTT-- --------GA TGAGTGGTGA AAACAAATAC CAACAAAAAC 149**

**He185/333_gDNA_0002 GGGAAAGGAT AGAATTGGAG AGACTATTCA GACATGGAGT TGAAAGTGAC ACTGATCGTT ACCCTTGTGG CTGCTATTAC CATCTCAGGT AAGAAATATT ATTATTT-GG TATTATTT-- --------GA TGAGTGGTGA AAACAAATAC CAACAAAAAC 149**

**He185/333_gDNA_0003 GGGAAAGGAT AGAATTGGAG AGACTATTCA GACATGGAGT TGAAAGTGAC ACTGATCGTT ACCCTTGTGG CTGCTATTAC CATCTCAGGT AAGAAATATT ATTATTT-GG TATTATTT-- --------GA TGAGTGGTGA AAACAAATAC CAACAAAAAC 149**

**He185/333_gDNA_0004 GAGAAAGGAT AGAATTGGAG AGACTATACA GACATGGAGT TGAAAGTGAC ACTGATCGTT GCCCTTGTGG CTGCTATTAC CATCTCAGGT AAGAAATATT ATTATTT-GG TATTATTT-- --------GA TGAGTGGTGA AAACAAATAC CAACAAAAAC 149**

**He185/333_gDNA_0005 GGGAAAGGAT AGAATTGGAG AGACTATTCA GACATGGAGT TGAAAGTGAC ACTGATCGTT ACCCTTGTGG CTGCTATTAC CATCTCAGGT AAGAAATATT ATTATTT-GG TATTATTT-- --------GA TGAGTGGTGA AAACAAATAC CAACAAAAAC 149**

**He185/333_gDNA_0006 GGGAAAGGAT AGAATTGGAG AGACTATTCA GACATGGAGT TGAAAGTGAC ACTGATCGTT ACCCTTGTGG CTGCTATTAC CATCTCAGGT AAGAAATATT ATTATTT-GG TATTATTT-- --------GA TGAGTGGTGA AAACAAATAC CAACAAAAAC 149**

**He185/333_gDNA_0007 GAGAAAGGAT AGAATTGGAG AGACTATACA GACATGGAGT TGAAAGTGAC ACTGATCGTT GCCCTTGTGG CTGCTATTAC CATCTCAGGT AAGAAATATT ATTATTT-GG TATTATTT-- --------GA TGAGTGGTGA AAACAAATAC CAACAAAAAC 149**

**He185/333_gDNA_0008 GGGAAAGGAT AGAATTGGAG AGACTATTCA GACATGGAGT TGAAAGTGAC ACTGATCGTT ACCCTTGTGG CTGCTATTAC CATCTCAGGT AAGAAATATT ATTATTT-GG TATTATTT-- --------GA TGAGTGGTGA AAACAAATAC CAACAAAAAC 149**

**He185/333_gDNA_0009 GAGAAAGGAT AGAATTGGAG AGACTATACA GACATGGAGT TGAAAGTGAC ACTGATCGTT GCCCTTGTGG CTGCTATTAC CATCTCAGGT AAGAAATATT ATTATTTTGG TATTATTTTG TATTATTTGA TGAGTGGTGA AAACAAATAC CAACAAAAAC 160**

**He185/333_gDNA_0010 GGGAAAGGAT AGAATTGGAG AGACTATTCA GACATGGAGT TGAAAGTGAC ACTGATCGTT ACCCTTGTGG CTGCTATTAC CATCTCAGGT AAGAAATATT ATTATTT-GG TATTATTT-- --------GA TGAGTGGTGA AAACAAATAC CAACAAAAAC 149**

**He185/333_gDNA_0011 GGGAAAGGAT AGAATTGGAG AGACTATTCA GACATGGAGT TGAAAGTGAC ACTGATCGTT ACCCTTGTGG CTGCTATTAC CATCTCAGGT AAGAAATATT ATTATTT-GG TATTATTT-- --------GA TGAGTGGTGA AAACAAATAC CAACAAAAAC 149**

**He185/333_gDNA_0012 GGGAAAGGAT AGAATTGGAG AGACTATTCA GACATGGAGT TGAAAGTGAC ACTGATCGTT ACCCTTGTGG CTGCTATTAC CATCTCAGGT AAGAAATATT ATTATTT-GG TATTATTT-- --------GA TGAGTGGTGA AAACAAATAC CAACAAAAAC 149**

**He185/333_gDNA_0013 GGGAAAGGAT AGAATTGGAG AGACTATTCA GACATGGAGT TGAAAGTGAC ACTGATCGTT ACCCTTGTGG CTGCTATTAC CATCTCAGGT AAGAAATATT ATTATTT-GG TATTATTT-- --------GA TGAGTGGTGA AAACAAATAC CAACAAAAAC 149**

**He185/333_gDNA_0014 GAGAAAGGAT AGAATTGGAG AGACTATACA GACATGGAGT TGAAAGTGAC ACTGATCGTT GCCATTTTGG CTGCTATTAC CATGTCAGGT AAGAAATATT ATTATTT-GG TTTTATTT-- --------GA TG-GTAGTGA AAAGAAATAC TATCAAAAAG 148**

**He185/333_gDNA_0015 GAGAAAGGAT AGAATTGGAG AGACTATACA GACATGGAGT TGAAAGTGAC ACTGATCGTT GCCCTTGTGG CTGCTATTAC CATCTCAGGT AAGAAATATT ATTATTT-GG TATTATTT-- --------GA TGAGTGGTGA AAACAAATAC CAACAAAAAC 149**

**He185/333_gDNA_0016 GAGAAAGGAT AGAATTGGAG AGACTATACA GACATGGAGT TGAAAGTGAC ACTGATCGTT GCCATTTTGG CTGCTATTAC CATGTCAGGT AAGAAATATT ATTATTT-GG TTTTATTT-- --------GA TG-GTAGTGA AAAGAAATAC TATCAAAAAG 148**

**He185/333_gDNA_0017 GAGAAAGGAT AGAATTGGAG AGACTATACA GACATGGAGT TGAAAGTGAC ACTGATCGTT GCCATTTTGG CTGCTATTAC CATGTCAGGT AAGAAATATT ATTATTT-GG TTTTATTT-- --------GA TG-GTAGTGA AAAGAAATAC TATCAAAAAG 148**

**He185/333_gDNA_0018 GAGAAAGGAT AGAATTGGAG AGACTATACA GACATGGAGT TGAAAGTGAC ACTGATCGTT GCCATTTTGG CTGCTATTAC CATGTCAGGT AAGAAATATT ATTATTT-GG TTTTATTT-- --------GA TG-GTAGTGA AAAGAAATAC TATCAAAAAG 148**

**He185/333_gDNA_0019 GAGAAAGGAT AGAATTGGAG AGACTATACA GACATGGAGT TGAAAGTGAC ACTGATCGTT GCCCTTGTGG CTGCTATTAC CATCTCAGGT AAGAAATATT ATTATTTTGG TATTATTTTG TATTATTTGA TGAGTGGTGA AAACAAATAC CAACAAAAAC 160**

**He185/333_gDNA_0020 GAGAAAGGAT AGAATTGGAG AGACTATACA GACATGGAGT TGAAAGTGAC ACTGATCGTT GCCCTTGTGG CTGCTATTAC CATCTCAGGT AAGAAATATT ATTATTTTGG TATTATTTTG TATTATTTGA TGAGTGGTGA AAACAAATAC CAACAAAAAC 160**

**He185/333_gDNA_0021 GAGAAAGGAT AGAATTGGAG AGACTATACA GACATGGAGT TGAAAGTGAC ACTGATCGTT GCCATTTTGG CTGCTATTAC CATGTCAGGT AAGAAATATT ATTATTT-GG TTTTATTT-- --------GA TG-GTAGTGA AAAGAAATAC TATCAAAAAG 148**

**He185/333_gDNA_0022 GAGAAAGGAT AGAATTGGAG AGACTATACA GACATGGAGT TGAAAGTGAC ACTGATCGTT GCCCTTGTGG CTGCTATTAC CATCTCAGGT AAGAAATATT ATTATTTTGG TATTATTTTG TATTATTTGA TGAGTGGTGA AAACAAATAC CAACAAAAAC 160**

**He185/333_gDNA_0023 GAGAAAGGAT AGAATTGGAG AGACTATACA GACATGGAGT TGAAAGTGAC ACTGATCGTT GCCATTTTGG CTGCTATTAC CATGTCAGGT AAGAAATATT ATTATTT-GG TTTTATTT-- --------GA TG-GTAGTGA AAAGAAATAC TATCAAAAAG 148**

**He185/333_gDNA_0024 GAGAAAGGAT AGAATTGGAG AGACTATACA GACATGGAGT TGAAAGTGAC ACTGATCGTT GCCCTTGTGG CTGCTATTAC CATCTCAGGT AAGAAATATT ATTATTT-GG TATTATTT-- --------GA TGAGTGGTGA AAACAAATAC CAACAAAAAC 149**

**He185/333_gDNA_0025 GAGAAAGGAT AGAATTGGAG AGACTATACA GACATGGAGT TGAAAGTGAC ACTGATCGTT GCCATTTTGG CTGCTATTAC CATGTCAGGT AAGAAATATT ATTATTT-GG TTTTATTT-- --------GA TG-GTAGTGA AAAGAAATAC TATCAAAAAG 148**

**He185/333_gDNA_0026 GAGAAAGGAT AGAATTGGAG AGACTATACA GACATGGAGT TGAAAGTGAC ACTGATCGTT GCCCTTGTGG CTGCTATTAC CATCTCAGGT AAGAAATATT ATTATTTTGG TATTATTTTG TATTATTTGA TGAGTGGTGA AAACAAATAC CAACAAAAAC 160**

**He185/333_gDNA_0027 GAGAAAGGAT AGAATTGGAG AGACTATACA GACATGGAGT TGAAAGTGAC ACTGATCGTT GCCCTTGTGG CTGCTATTAC CATCTCAGGT AAGAAATATT ATTATTT-GG TATTATTT-- --------GA TGAGTGGTGA AAACAAATAC CAACAAAAAC 149**

**He185/333_gDNA_0028 GAGAAAGGAT AGAATTGGAG AGACTATACA GACATGGAGT TGAAAGTGAC ACTGATCGTT GCCATTTTGG CTGCTATTAC CATGTCAGGT AAGAAATATT ATTATTT-GG TTTTATTT-- --------GA TG-GTAGTGA AAAGAAATAC TATCAAAAAG 148**

**He185/333_gDNA_0029 GAGAAAGGAT AGAATTGGAG AGACTATACA GACATGGAGT TGAAAGTGAC ACTGATCGTT GCCATTCTGG CTGCTATTAC CATGTCAGGT AAGAAATATT ATTATTT-GG TTTTATTT-- --------GA TG-GTAGTGA AAAGAAATAC TATCAAAAAG 148**

**He185/333_gDNA_0030 GAGAAAGGAT AGAATTGGAG AGACTATACA GACATGGAGT TGAAAGTGAC ACTGATCGTT GCCCTTGTGG CTGCTATTAC CATCTCAGGT AAGAAATATT ATTATTTTGG TATTATTTTG TATTATTTGA TGAGTGGTGA AAACAAATAC CAACAAAAAC 160**

**He185/333_gDNA_0031 GAGAAAGGAT AGAATTGGAG AGACTATACA GACATGGAGT TGAAAGTGAC ACTGATCGTT GCCCTTGTGG CTGCTATTAC CATCTCAGGT AAGAAATATT ATTATTT-GG TATTATTT-- --------GA TGAGTGGTGA AAACAAATAC CAACAAAAAC 149**

**He185/333_gDNA_0032 GAGAAAGGAT AGAATTGGAG AGACTATACA GACATGGAGT TGAAAGTGAC ACTGATCGTT GCCATTTTGG CTGCTATTAC CATGTCAGGT AAGAAATATT ATTATTT-GG TTTTATTT-- --------GA TG-GTAGTGA AAAGAAATAC TATCAAAAAG 148**

**He185/333_gDNA_0033 GAGAAAGGAT AGAATTGGAG AGACTATACA GACATGGAGT TGAAAGTGAC ACTGATCGTT GCCCTTGTGG CTGCTATTAC CATCTCAGGT AAGAAATATT ATTATTTTGG TATTATTTTG TATTATTTGA TGAGTGGTGA AAACAAATAC CAACAAAAAC 160**

**He185/333_gDNA_0034 GAGAAAGGAT AGAATTGGAG AGACTATACA GACATGGAGT TGAAAGTGAC ACTGATCGTT GCCCTTGTGG ATGCTATTAC CATCTCAGGT AAGAAATATT ATTATTTTGG TATTATTTTG TATTATTTGA TGAGTGGTGA AAACAAATAC CAACAAAAAC 160**

**He185/333_gDNA_0035 GAGAAAGGAT AGAATTGGAG AGACTATACA GACATGGAGT TGAAAGTGAC ACTGATCGTT GCCATTTTGG CTGCTATTAC CATGTCAGGT AAGAAATATT ATTATTT-GG TTTTATTT-- --------GA TG-GTAGTGA AAAGAAATAC TATCAAAAAG 148**

**He185/333_gDNA_0036 GAGAAAGGAT AGAATTGGAG AGACTATACA GACATGGAGT TGAAAGTGAC ACTGATCGTT GCCCTTGTGG CTGCTATTAC CATCTCAGGT AAGAAATATT ATTATTT-GG TATTATTT-- --------GA TGAGTGGTGA AAACAAATAC CAACAAAAAC 149**

**He185/333_gDNA_0037 GAGAAAGGAT AGAATTGGAG AGACTATACA GACATGGAGT TGAAAGTGAC ACTGATCGTT GCCCTTGTGG CTGCTATTAC CATCTCAGGT AAGAAATATT ATTATTT-GG TATTATTT-- --------GA TGAGTGGTGA AAACAAATAC CAACAAAAAC 149**

**He185/333_gDNA_0038 GAGAAAGGAT AGAATTGGAG AGACTATACA GACATGGAGT TGAAAGTGAC ACTGATCGTT GCCCTTGTGG CTGCTATTAC CATCTCAGGT AAGAAATATT ATTATTT-GG TATTATTT-- --------GA TGAGTGGTGA AAACAAATAC CAACAAAAAC 149**

**He185/333_gDNA_0039 GAGAAAGGAT AGAATTGGAG AGACTATACA GACATGGAGT TGAAAGTGAC ACTGATCGTT GCCCTTGTGG CTGCTATTAC CATCTCAGGT AAGAAATATT ATTATTTTGG TATTATTTTG TATTATTTGA TGAGTGGTGA AAACAAATAC CAACAAAAAC 160**

i3

i2

**....|....| ....|....| ....|....| ....|....| ....|....| ....|....| ....|....| ....|....| ....|....| ....|....| ....|....| ....|....| ....|....| ....|....| ....|....| ....|....|**

**165 175 185 195 205 215 225 235 245 255 265 275 285 295 305 315**

**He185/333_gDNA_0001 GCTCATGTAA GGGCAATTTT TTTACGTGAC TCTTTAAT-- ---------- ---------- ---------- ---------- ---------- ---------- ---------- ---------- ---------- ---------- ---------- ---------- 187**

**He185/333_gDNA_0002 GCTCATGTAA GGGCAATTTT TTTACGTGAC TCTTTAAT-- ---------- ---------- ---------- ---------- ---------- ---------- ---------- ---------- ---------- ---------- ---------- ---------- 187**

**He185/333_gDNA_0003 GCTCATGTAA GGGCAATTTT TTTACGTGAC TCTTTAAT-- ---------- ---------- ---------- ---------- ---------- ---------- ---------- ---------- ---------- ---------- ---------- ---------- 187**

**He185/333_gDNA_0004 GCTCATGTAA GGGCAATTTT TTTACGTGAC TCTTTAAC-- ---------- ---------- ---------- ---------- ---------- ---------- ---------- ---------- ---------- ---------- ---------- ---------- 187**

**He185/333_gDNA_0005 GCTCATGTAA GGGCAATTTT TTTACGTGAC TCTTTAAT-- ---------- ---------- ---------- ---------- ---------- ---------- ---------- ---------- ---------- ---------- ---------- ---------- 187**

**He185/333_gDNA_0006 GCTCATGTAA GGGCAATTTT TTTACGTGAC TCTTTAAT-- ---------- ---------- ---------- ---------- ---------- ---------- ---------- ---------- ---------- ---------- ---------- ---------- 187**

**He185/333_gDNA_0007 GCTCATGTAA GGGCAATTTT TTTACGTGAC TCTTTAAC-- ---------- ---------- ---------- ---------- ---------- ---------- ---------- ---------- ---------- ---------- ---------- ---------- 187**

**He185/333_gDNA_0008 GCTCATGTAA GGGCAATTTT TTTACGTGAC TCTTTAAT-- ---------- ---------- ---------- ---------- ---------- ---------- ---------- ---------- ---------- ---------- ---------- ---------- 187**

**He185/333_gDNA_0009 GCTCATGTAA GGGCAATTTT TTTACGTGAC TCTTTAAT-- ---------- ---------- ---------- ---------- ---------- ---------- ---------- ---------- ---------- ---------- ---------- ---------- 198**

**He185/333_gDNA_0010 GCTCATGTAA GGGCAATTTT TTTACGTGAC TCTTTAAT-- ---------- ---------- ---------- ---------- ---------- ---------- ---------- ---------- ---------- ---------- ---------- ---------- 187**

**He185/333_gDNA_0011 GCTCATGTAA GGGCAATTTT TTTACGTGAC TCTTTAAT-- ---------- ---------- ---------- ---------- ---------- ---------- ---------- ---------- ---------- ---------- ---------- ---------- 187**

**He185/333_gDNA_0012 GCTCATGTAA GGGCAATTTT TTTACGTGAC TCTTTAAT-- ---------- ---------- ---------- ---------- ---------- ---------- ---------- ---------- ---------- ---------- ---------- ---------- 187**

**He185/333_gDNA_0013 GCTCATGTAA GGGCAATTTT TTTACGTGAC TCTTTAAT-- ---------- ---------- ---------- ---------- ---------- ---------- ---------- ---------- ---------- ---------- ---------- ---------- 187**

**He185/333_gDNA_0014 GCTCAAGTAA AGGCAATT-T TATACGTGAC TCTTCAAT-- ---------- ---------- ---------- ---------- ---------- ---------- ---------- ---------- ---------- ---------- ---------- ---------- 185**

**He185/333_gDNA_0015 GCTCATGTAA GGGCAATTTT TTTACGTGAC TCTTTAAC-- ---------- ---------- ---------- ---------- ---------- ---------- ---------- ---------- ---------- ---------- ---------- ---------- 187**

**He185/333_gDNA_0016 GCTCAAGTAA AGGCAATT-T TATACGTGAC TCTTCAAT-- ---------- ---------- ---------- ---------- ---------- ---------- ---------- ---------- ---------- ---------- ---------- ---------- 185**

**He185/333_gDNA_0017 GCTCAAGTAA AGGCAATT-T TATACGTGAC TCTTCAAT-- ---------- ---------- ---------- ---------- ---------- ---------- ---------- ---------- ---------- ---------- ---------- ---------- 185**

**He185/333_gDNA_0018 GCTCAAGTAA AGGCAATT-T TATACGTGAC TCTTCAAT-- ---------- ---------- ---------- ---------- ---------- ---------- ---------- ---------- ---------- ---------- ---------- ---------- 185**

**He185/333_gDNA_0019 GCTCATGTAA GGGCAATTTT TTTACGTGAC TCTTTAAT-- ---------- ---------- ---------- ---------- ---------- ---------- ---------- ---------- ---------- ---------- ---------- ---------- 198**

**He185/333_gDNA_0020 GCTCATGTAA GGGCAATTTT TTTACGTGAC TCTTTAAT-- ---------- ---------- ---------- ---------- ---------- ---------- ---------- ---------- ---------- ---------- ---------- ---------- 198**

**He185/333_gDNA_0021 GCTCAAGTAA AGGCAATT-T TATACGTGAC TCTTCAAT-- ---------- ---------- ---------- ---------- ---------- ---------- ---------- ---------- ---------- ---------- ---------- ---------- 185**

**He185/333_gDNA_0022 GCTCATGTAA GGGCAATTTT TTTACGTGAC TCTTTAAT-- ---------- ---------- ---------- ---------- ---------- ---------- ---------- ---------- ---------- ---------- ---------- ---------- 198**

**He185/333_gDNA_0023 GCTCAAGTAA AGGCAATT-T TATACGTGAC TCTTCAAT-- ---------- ---------- ---------- ---------- ---------- ---------- ---------- ---------- ---------- ---------- ---------- ---------- 185**

**He185/333_gDNA_0024 GCTCATGTAA TGGCAATTGT TTTACGTGAC TCTTTAAT-- ---------- ---------- ---------- ---------- ---------- ---------- ---------- ---------- ---------- ---------- ---------- ---------- 187**

**He185/333_gDNA_0025 GCTCAAGTAA AGGCAATT-T TATACGTGAC TCTTCAAT-- ---------- ---------- ---------- ---------- ---------- ---------- ---------- ---------- ---------- ---------- ---------- ---------- 185**

**He185/333_gDNA_0026 GCTCATGTAA GGGCAATTTT TTTACGTGAC TCTTTAAT-- ---------- ---------- ---------- ---------- ---------- ---------- ---------- ---------- ---------- ---------- ---------- ---------- 198**

**He185/333_gDNA_0027 GCTCATGTAA TGGCAATTGT TTTACGTGAC TCTTTAAT-- ---------- ---------- ---------- ---------- ---------- ---------- ---------- ---------- ---------- ---------- ---------- ---------- 187**

**He185/333_gDNA_0028 GCTCAAGTAA AGGCAATT-T TATACGTGAC TCTTCAAT-- ---------- ---------- ---------- ---------- ---------- ---------- ---------- ---------- ---------- ---------- ---------- ---------- 185**

**He185/333_gDNA_0029 GCTCAAGTAA AGGCAATT-T TATACGTGAC TCTTCAAT-- ---------- ---------- ---------- ---------- ---------- ---------- ---------- ---------- ---------- ---------- ---------- ---------- 185**

**He185/333_gDNA_0030 GCTCATGTAA GGGCAATTTT TTTACGTGAC TCTTTAAT-- ---------- ---------- ---------- ---------- ---------- ---------- ---------- ---------- ---------- ---------- ---------- ---------- 198**

**He185/333_gDNA_0031 GCTCATGTAA TGGCAATTGT TTTACGTGAC TCTTTAAT-- ---------- ---------- ---------- ---------- ---------- ---------- ---------- ---------- ---------- ---------- ---------- ---------- 187**

**He185/333_gDNA_0032 GCTCAAGTAA AGGCAATT-T TATACGTGAC TCTTCAAT-- ---------- ---------- ---------- ---------- ---------- ---------- ---------- ---------- ---------- ---------- ---------- ---------- 185**

**He185/333_gDNA_0033 GCTCATGTAA GGGCAATTTT TTTACGTGAC TCTTTAAT-- ---------- ---------- ---------- ---------- ---------- ---------- ---------- ---------- ---------- ---------- ---------- ---------- 198**

**He185/333_gDNA_0034 GCTCATGTAA GGGCAATTTT TTTACGTGAC TCTTTAAT-- ---------- ---------- ---------- ---------- ---------- ---------- ---------- ---------- ---------- ---------- ---------- ---------- 198**

**He185/333_gDNA_0035 GCTCAAGTAA AGGCAATT-T TATACGTGAC TCTTCAAT-- ---------- ---------- ---------- ---------- ---------- ---------- ---------- ---------- ---------- ---------- ---------- ---------- 195**

**He185/333_gDNA_0036 GCTCATGTAA TGGCAATTGT TTTACGTGAC TCTTTAAT-- ---------- ---------- ---------- ---------- ---------- ---------- ---------- ---------- ---------- ---------- ---------- ---------- 187**

**He185/333_gDNA_0037 GCTCATGTAA GGGCAATTTT TTTACGTGAC TCTTTAAAGT AGAACACTCA AGAAAATCAC AAATATGGCA ATTAATGTAT CACTAGAAAC TGGGAAGCTT ACAGATGGCA ATCGTATAAG TTTTATGTCA ACTGCGTATA TGATGATTAT AAAAACAACA 309**

**He185/333_gDNA_0038 GCTCATGTAA GGGCAATTTT TTTACGTGAC TCTTTAAAGT AGAACACTCA AGAAAATCAC AAATATGGCA ATTAATGTAT CACTAGAAAC TGGGAAGCTT ACAGATGGCA ATCGTATAAG TTTTATGTCA ACTGCGTATA TGATGATTAT AAAAACAACA 309**

**He185/333_gDNA_0039 GCTCATGTAA GGGCAATTTT TTTACGTGAC TCTTTAAT-- ---------- ---------- ---------- ---------- ---------- ---------- ---------- ---------- ---------- ---------- ---------- ---------- 198**

**....|....| ....|....| ....|....| ....|....| ....|....| ....|....| ....|....| ....|....| ....|....| ....|....| ....|....| ....|....| ....|....| ....|....| ....|....| ....|....|**

i3

**325 335 345 355 365 375 385 395 405 415 425 435 445 455 465 475**

**He185/333_gDNA_0001 ---------- ---------- ---------- ---------- ---------- ---------- ---------- ---------- ---------- ---------- ---------- ---------- ---------- ---------- ---------- ---------- 187**

**He185/333_gDNA_0002 ---------- ---------- ---------- ---------- ---------- ---------- ---------- ---------- ---------- ---------- ---------- ---------- ---------- ---------- ---------- ---------- 187**

**He185/333_gDNA_0003 ---------- ---------- ---------- ---------- ---------- ---------- ---------- ---------- ---------- ---------- ---------- ---------- ---------- ---------- ---------- ---------- 187**

**He185/333_gDNA_0004 ---------- ---------- ---------- ---------- ---------- ---------- ---------- ---------- ---------- ---------- ---------- ---------- ---------- ---------- ---------- ---------- 187**

**He185/333_gDNA_0005 ---------- ---------- ---------- ---------- ---------- ---------- ---------- ---------- ---------- ---------- ---------- ---------- ---------- ---------- ---------- ---------- 187**

**He185/333_gDNA_0006 ---------- ---------- ---------- ---------- ---------- ---------- ---------- ---------- ---------- ---------- ---------- ---------- ---------- ---------- ---------- ---------- 187**

**He185/333_gDNA_0007 ---------- ---------- ---------- ---------- ---------- ---------- ---------- ---------- ---------- ---------- ---------- ---------- ---------- ---------- ---------- ---------- 187**

**He185/333_gDNA_0008 ---------- ---------- ---------- ---------- ---------- ---------- ---------- ---------- ---------- ---------- ---------- ---------- ---------- ---------- ---------- ---------- 187**

**He185/333_gDNA_0009 ---------- ---------- ---------- ---------- ---------- ---------- ---------- ---------- ---------- ---------- ---------- ---------- ---------- ---------- ---------- ---------- 198**

**He185/333_gDNA_0010 ---------- ---------- ---------- ---------- ---------- ---------- ---------- ---------- ---------- ---------- ---------- ---------- ---------- ---------- ---------- ---------- 187**

**He185/333_gDNA_0011 ---------- ---------- ---------- ---------- ---------- ---------- ---------- ---------- ---------- ---------- ---------- ---------- ---------- ---------- ---------- ---------- 187**

**He185/333_gDNA_0012 ---------- ---------- ---------- ---------- ---------- ---------- ---------- ---------- ---------- ---------- ---------- ---------- ---------- ---------- ---------- ---------- 187**

**He185/333_gDNA_0013 ---------- ---------- ---------- ---------- ---------- ---------- ---------- ---------- ---------- ---------- ---------- ---------- ---------- ---------- ---------- ---------- 187**

**He185/333_gDNA_0014 ---------- ---------- ---------- ---------- ---------- ---------- ---------- ---------- ---------- ---------- ---------- ---------- ---------- ---------- ---------- ---------- 185**

**He185/333_gDNA_0015 ---------- ---------- ---------- ---------- ---------- ---------- ---------- ---------- ---------- ---------- ---------- ---------- ---------- ---------- ---------- ---------- 187**

**He185/333_gDNA_0016 ---------- ---------- ---------- ---------- ---------- ---------- ---------- ---------- ---------- ---------- ---------- ---------- ---------- ---------- ---------- ---------- 185**

**He185/333_gDNA_0017 ---------- ---------- ---------- ---------- ---------- ---------- ---------- ---------- ---------- ---------- ---------- ---------- ---------- ---------- ---------- ---------- 185**

**He185/333_gDNA_0018 ---------- ---------- ---------- ---------- ---------- ---------- ---------- ---------- ---------- ---------- ---------- ---------- ---------- ---------- ---------- ---------- 185**

**He185/333_gDNA_0019 ---------- ---------- ---------- ---------- ---------- ---------- ---------- ---------- ---------- ---------- ---------- ---------- ---------- ---------- ---------- ---------- 198**

**He185/333_gDNA_0020 ---------- ---------- ---------- ---------- ---------- ---------- ---------- ---------- ---------- ---------- ---------- ---------- ---------- ---------- ---------- ---------- 198**

**He185/333_gDNA_0021 ---------- ---------- ---------- ---------- ---------- ---------- ---------- ---------- ---------- ---------- ---------- ---------- ---------- ---------- ---------- ---------- 185**

**He185/333_gDNA_0022 ---------- ---------- ---------- ---------- ---------- ---------- ---------- ---------- ---------- ---------- ---------- ---------- ---------- ---------- ---------- ---------- 198**

**He185/333_gDNA_0023 ---------- ---------- ---------- ---------- ---------- ---------- ---------- ---------- ---------- ---------- ---------- ---------- ---------- ---------- ---------- ---------- 185**

**He185/333_gDNA_0024 ---------- ---------- ---------- ---------- ---------- ---------- ---------- ---------- ---------- ---------- ---------- ---------- ---------- ---------- ---------- ---------- 187**

**He185/333_gDNA_0025 ---------- ---------- ---------- ---------- ---------- ---------- ---------- ---------- ---------- ---------- ---------- ---------- ---------- ---------- ---------- ---------- 185**

**He185/333_gDNA_0026 ---------- ---------- ---------- ---------- ---------- ---------- ---------- ---------- ---------- ---------- ---------- ---------- ---------- ---------- ---------- ---------- 198**

**He185/333_gDNA_0027 ---------- ---------- ---------- ---------- ---------- ---------- ---------- ---------- ---------- ---------- ---------- ---------- ---------- ---------- ---------- ---------- 187**

**He185/333_gDNA_0028 ---------- ---------- ---------- ---------- ---------- ---------- ---------- ---------- ---------- ---------- ---------- ---------- ---------- ---------- ---------- ---------- 185**

**He185/333_gDNA_0029 ---------- ---------- ---------- ---------- ---------- ---------- ---------- ---------- ---------- ---------- ---------- ---------- ---------- ---------- ---------- ---------- 185**

**He185/333_gDNA_0030 ---------- ---------- ---------- ---------- ---------- ---------- ---------- ---------- ---------- ---------- ---------- ---------- ---------- ---------- ---------- ---------- 198**

**He185/333_gDNA_0031 ---------- ---------- ---------- ---------- ---------- ---------- ---------- ---------- ---------- ---------- ---------- ---------- ---------- ---------- ---------- ---------- 187**

**He185/333_gDNA_0032 ---------- ---------- ---------- ---------- ---------- ---------- ---------- ---------- ---------- ---------- ---------- ---------- ---------- ---------- ---------- ---------- 185**

**He185/333_gDNA_0033 ---------- ---------- ---------- ---------- ---------- ---------- ---------- ---------- ---------- ---------- ---------- ---------- ---------- ---------- ---------- ---------- 198**

**He185/333_gDNA_0034 ---------- ---------- ---------- ---------- ---------- ---------- ---------- ---------- ---------- ---------- ---------- ---------- ---------- ---------- ---------- ---------- 198**

**He185/333_gDNA_0035 ---------- ---------- ---------- ---------- ---------- ---------- ---------- ---------- ---------- ---------- ---------- ---------- ---------- ---------- ---------- ---------- 185**

**He185/333_gDNA_0036 ---------- ---------- ---------- ---------- ---------- ---------- ---------- ---------- ---------- ---------- ---------- ---------- ---------- ---------- ---------- ---------- 187**

**He185/333_gDNA_0037 TAAATAAAAG CTCTTCTCAG CATGTGGAAT ATCCGAGGAT TCGCCAGAAA TATAGCGATG TTTTGATGTC GTCATACGAG GGATCGATCC TGCTTAAGCA GACGACGTCC TATAATGAAT GAACGTAATT GCAATGCAAT TATTTATCAA ATTATAAAGA 469**

**He185/333_gDNA_0038 TAAATAAAAG CTCTTCTCAG CATGTGGAAT ATCCGAGGAT TCGCCAGAAA TATAGCGATG TTTTGATGTC GTCATACGAG GGATCGATCC TGCTTAAGCA GACGACGTCC TATAATGAAT GAACGTAATT GCAATGCAAT TATTTATCAA ATTATAAAGA 469**

**He185/333_gDNA_0039 ---------- ---------- ---------- ---------- ---------- ---------- ---------- ---------- ---------- ---------- ---------- ---------- ---------- ---------- ---------- ---------- 198**

**....|....| ....|....| ....|....| ....|....| ....|....| ....|....| ....|....| ....|....| ....|....| ....|....| ....|....| ....|....| ....|....| ....|....| ....|....| ....|....|**

i3

**485 495 505 515 525 535 545 555 565 575 585 595 605 615 625 635**

**He185/333_gDNA_0001 ---------- ---------- ---------- ---------- ---------- ---------- ---------- ---------- ---------- ---------- ---------- ---------- ---------- ---------- ---------- ---------- 187**

**He185/333_gDNA_0002 ---------- ---------- ---------- ---------- ---------- ---------- ---------- ---------- ---------- ---------- ---------- ---------- ---------- ---------- ---------- ---------- 187**

**He185/333_gDNA_0003 ---------- ---------- ---------- ---------- ---------- ---------- ---------- ---------- ---------- ---------- ---------- ---------- ---------- ---------- ---------- ---------- 187**

**He185/333_gDNA_0004 ---------- ---------- ---------- ---------- ---------- ---------- ---------- ---------- ---------- ---------- ---------- ---------- ---------- ---------- ---------- ---------- 187**

**He185/333_gDNA_0005 ---------- ---------- ---------- ---------- ---------- ---------- ---------- ---------- ---------- ---------- ---------- ---------- ---------- ---------- ---------- ---------- 187**

**He185/333_gDNA_0006 ---------- ---------- ---------- ---------- ---------- ---------- ---------- ---------- ---------- ---------- ---------- ---------- ---------- ---------- ---------- ---------- 187**

**He185/333_gDNA_0007 ---------- ---------- ---------- ---------- ---------- ---------- ---------- ---------- ---------- ---------- ---------- ---------- ---------- ---------- ---------- ---------- 187**

**He185/333_gDNA_0008 ---------- ---------- ---------- ---------- ---------- ---------- ---------- ---------- ---------- ---------- ---------- ---------- ---------- ---------- ---------- ---------- 187**

**He185/333_gDNA_0009 ---------- ---------- ---------- ---------- ---------- ---------- ---------- ---------- ---------- ---------- ---------- ---------- ---------- ---------- ---------- ---------- 198**

**He185/333_gDNA_0010 ---------- ---------- ---------- ---------- ---------- ---------- ---------- ---------- ---------- ---------- ---------- ---------- ---------- ---------- ---------- ---------- 187**

**He185/333_gDNA_0011 ---------- ---------- ---------- ---------- ---------- ---------- ---------- ---------- ---------- ---------- ---------- ---------- ---------- ---------- ---------- ---------- 187**

**He185/333_gDNA_0012 ---------- ---------- ---------- ---------- ---------- ---------- ---------- ---------- ---------- ---------- ---------- ---------- ---------- ---------- ---------- ---------- 187**

**He185/333_gDNA_0013 ---------- ---------- ---------- ---------- ---------- ---------- ---------- ---------- ---------- ---------- ---------- ---------- ---------- ---------- ---------- ---------- 187**

**He185/333_gDNA_0014 ---------- ---------- ---------- ---------- ---------- ---------- ---------- ---------- ---------- ---------- ---------- ---------- ---------- ---------- ---------- ---------- 185**

**He185/333_gDNA_0015 ---------- ---------- ---------- ---------- ---------- ---------- ---------- ---------- ---------- ---------- ---------- ---------- ---------- ---------- ---------- ---------- 187**

**He185/333_gDNA_0016 ---------- ---------- ---------- ---------- ---------- ---------- ---------- ---------- ---------- ---------- ---------- ---------- ---------- ---------- ---------- ---------- 185**

**He185/333_gDNA_0017 ---------- ---------- ---------- ---------- ---------- ---------- ---------- ---------- ---------- ---------- ---------- ---------- ---------- ---------- ---------- ---------- 185**

**He185/333_gDNA_0018 ---------- ---------- ---------- ---------- ---------- ---------- ---------- ---------- ---------- ---------- ---------- ---------- ---------- ---------- ---------- ---------- 185**

**He185/333_gDNA_0019 ---------- ---------- ---------- ---------- ---------- ---------- ---------- ---------- ---------- ---------- ---------- ---------- ---------- ---------- ---------- ---------- 198**

**He185/333_gDNA_0020 ---------- ---------- ---------- ---------- ---------- ---------- ---------- ---------- ---------- ---------- ---------- ---------- ---------- ---------- ---------- ---------- 198**

**He185/333_gDNA_0021 ---------- ---------- ---------- ---------- ---------- ---------- ---------- ---------- ---------- ---------- ---------- ---------- ---------- ---------- ---------- ---------- 185**

**He185/333_gDNA_0022 ---------- ---------- ---------- ---------- ---------- ---------- ---------- ---------- ---------- ---------- ---------- ---------- ---------- ---------- ---------- ---------- 198**

**He185/333_gDNA_0023 ---------- ---------- ---------- ---------- ---------- ---------- ---------- ---------- ---------- ---------- ---------- ---------- ---------- ---------- ---------- ---------- 185**

**He185/333_gDNA_0024 ---------- ---------- ---------- ---------- ---------- ---------- ---------- ---------- ---------- ---------- ---------- ---------- ---------- ---------- ---------- ---------- 187**

**He185/333_gDNA_0025 ---------- ---------- ---------- ---------- ---------- ---------- ---------- ---------- ---------- ---------- ---------- ---------- ---------- ---------- ---------- ---------- 185**

**He185/333_gDNA_0026 ---------- ---------- ---------- ---------- ---------- ---------- ---------- ---------- ---------- ---------- ---------- ---------- ---------- ---------- ---------- ---------- 198**

**He185/333_gDNA_0027 ---------- ---------- ---------- ---------- ---------- ---------- ---------- ---------- ---------- ---------- ---------- ---------- ---------- ---------- ---------- ---------- 187**

**He185/333_gDNA_0028 ---------- ---------- ---------- ---------- ---------- ---------- ---------- ---------- ---------- ---------- ---------- ---------- ---------- ---------- ---------- ---------- 185**

**He185/333_gDNA_0029 ---------- ---------- ---------- ---------- ---------- ---------- ---------- ---------- ---------- ---------- ---------- ---------- ---------- ---------- ---------- ---------- 185**

**He185/333_gDNA_0030 ---------- ---------- ---------- ---------- ---------- ---------- ---------- ---------- ---------- ---------- ---------- ---------- ---------- ---------- ---------- ---------- 198**

**He185/333_gDNA_0031 ---------- ---------- ---------- ---------- ---------- ---------- ---------- ---------- ---------- ---------- ---------- ---------- ---------- ---------- ---------- ---------- 187**

**He185/333_gDNA_0032 ---------- ---------- ---------- ---------- ---------- ---------- ---------- ---------- ---------- ---------- ---------- ---------- ---------- ---------- ---------- ---------- 185**

**He185/333_gDNA_0033 ---------- ---------- ---------- ---------- ---------- ---------- ---------- ---------- ---------- ---------- ---------- ---------- ---------- ---------- ---------- ---------- 198**

**He185/333_gDNA_0034 ---------- ---------- ---------- ---------- ---------- ---------- ---------- ---------- ---------- ---------- ---------- ---------- ---------- ---------- ---------- ---------- 198**

**He185/333_gDNA_0035 ---------- ---------- ---------- ---------- ---------- ---------- ---------- ---------- ---------- ---------- ---------- ---------- ---------- ---------- ---------- ---------- 185**

**He185/333_gDNA_0036 ---------- ---------- ---------- ---------- ---------- ---------- ---------- ---------- ---------- ---------- ---------- ---------- ---------- ---------- ---------- ---------- 187**

**He185/333_gDNA_0037 GTTTGGTACA TTTAAACATG ACCTTATTTC ACACCATTCT AAAAGGGAAA GTGAACTGAT TCTGATAACT AAAACCGTTT GATTGTAAAT ACACAGGAGA AGGGTCAATG ATCAATGAAA TAAACCCCAT TTTCAAATTG AAAAGCCCTG CGAATAATAG 629**

**He185/333_gDNA_0038 GTTTGGTACA TTTAAACATG ACCTTATTTC ACACCATTCT AAAAGGGAAA GTGAACTGAT TCTGATAACT AAAACCGTTT GATTGTAAAT ACACAGGAGA AGGGTCAATG ATCAATGAAA TAAACCCCAT TTTCAAATTG AAAAGCCCTG CGAATAATAG 629**

**He185/333_gDNA_0039 ---------- ---------- ---------- ---------- ---------- ---------- ---------- ---------- ---------- ---------- ---------- ---------- ---------- ---------- ---------- ---------- 198**

**....|....| ....|....| ....|....| ....|....| ....|....| ....|....| ....|....| ....|....| ....|....| ....|....| ....|....| ....|....| ....|....| ....|....| ....|....| ....|....|**

i3

**645 655 665 675 685 695 705 715 725 735 745 755 765 775 785 795**

**He185/333_gDNA_0001 ---------- ---------- ---------- ---------- ---------- ---------- ---------- ---------- ---------- ---------- ---------- ---------- ---------- ---------- ---------- ---------- 187**

**He185/333_gDNA_0002 ---------- ---------- ---------- ---------- ---------- ---------- ---------- ---------- ---------- ---------- ---------- ---------- ---------- ---------- ---------- ---------- 187**

**He185/333_gDNA_0003 ---------- ---------- ---------- ---------- ---------- ---------- ---------- ---------- ---------- ---------- ---------- ---------- ---------- ---------- ---------- ---------- 187**

**He185/333_gDNA_0004 ---------- ---------- ---------- ---------- ---------- ---------- ---------- ---------- ---------- ---------- ---------- ---------- ---------- ---------- ---------- ---------- 187**

**He185/333_gDNA_0005 ---------- ---------- ---------- ---------- ---------- ---------- ---------- ---------- ---------- ---------- ---------- ---------- ---------- ---------- ---------- ---------- 187**

**He185/333_gDNA_0006 ---------- ---------- ---------- ---------- ---------- ---------- ---------- ---------- ---------- ---------- ---------- ---------- ---------- ---------- ---------- ---------- 187**

**He185/333_gDNA_0007 ---------- ---------- ---------- ---------- ---------- ---------- ---------- ---------- ---------- ---------- ---------- ---------- ---------- ---------- ---------- ---------- 187**

**He185/333_gDNA_0008 ---------- ---------- ---------- ---------- ---------- ---------- ---------- ---------- ---------- ---------- ---------- ---------- ---------- ---------- ---------- ---------- 187**

**He185/333_gDNA_0009 ---------- ---------- ---------- ---------- ---------- ---------- ---------- ---------- ---------- ---------- ---------- ---------- ---------- ---------- ---------- ---------- 198**

**He185/333_gDNA_0010 ---------- ---------- ---------- ---------- ---------- ---------- ---------- ---------- ---------- ---------- ---------- ---------- ---------- ---------- ---------- ---------- 187**

**He185/333_gDNA_0011 ---------- ---------- ---------- ---------- ---------- ---------- ---------- ---------- ---------- ---------- ---------- ---------- ---------- ---------- ---------- ---------- 187**

**He185/333_gDNA_0012 ---------- ---------- ---------- ---------- ---------- ---------- ---------- ---------- ---------- ---------- ---------- ---------- ---------- ---------- ---------- ---------- 187**

**He185/333_gDNA_0013 ---------- ---------- ---------- ---------- ---------- ---------- ---------- ---------- ---------- ---------- ---------- ---------- ---------- ---------- ---------- ---------- 187**

**He185/333_gDNA_0014 ---------- ---------- ---------- ---------- ---------- ---------- ---------- ---------- ---------- ---------- ---------- ---------- ---------- ---------- ---------- ---------- 185**

**He185/333_gDNA_0015 ---------- ---------- ---------- ---------- ---------- ---------- ---------- ---------- ---------- ---------- ---------- ---------- ---------- ---------- ---------- ---------- 187**

**He185/333_gDNA_0016 ---------- ---------- ---------- ---------- ---------- ---------- ---------- ---------- ---------- ---------- ---------- ---------- ---------- ---------- ---------- ---------- 185**

**He185/333_gDNA_0017 ---------- ---------- ---------- ---------- ---------- ---------- ---------- ---------- ---------- ---------- ---------- ---------- ---------- ---------- ---------- ---------- 185**

**He185/333_gDNA_0018 ---------- ---------- ---------- ---------- ---------- ---------- ---------- ---------- ---------- ---------- ---------- ---------- ---------- ---------- ---------- ---------- 185**

**He185/333_gDNA_0019 ---------- ---------- ---------- ---------- ---------- ---------- ---------- ---------- ---------- ---------- ---------- ---------- ---------- ---------- ---------- ---------- 198**

**He185/333_gDNA_0020 ---------- ---------- ---------- ---------- ---------- ---------- ---------- ---------- ---------- ---------- ---------- ---------- ---------- ---------- ---------- ---------- 198**

**He185/333_gDNA_0021 ---------- ---------- ---------- ---------- ---------- ---------- ---------- ---------- ---------- ---------- ---------- ---------- ---------- ---------- ---------- ---------- 185**

**He185/333_gDNA_0022 ---------- ---------- ---------- ---------- ---------- ---------- ---------- ---------- ---------- ---------- ---------- ---------- ---------- ---------- ---------- ---------- 198**

**He185/333_gDNA_0023 ---------- ---------- ---------- ---------- ---------- ---------- ---------- ---------- ---------- ---------- ---------- ---------- ---------- ---------- ---------- ---------- 185**

**He185/333_gDNA_0024 ---------- ---------- ---------- ---------- ---------- ---------- ---------- ---------- ---------- ---------- ---------- ---------- ---------- ---------- ---------- ---------- 187**

**He185/333_gDNA_0025 ---------- ---------- ---------- ---------- ---------- ---------- ---------- ---------- ---------- ---------- ---------- ---------- ---------- ---------- ---------- ---------- 185**

**He185/333_gDNA_0026 ---------- ---------- ---------- ---------- ---------- ---------- ---------- ---------- ---------- ---------- ---------- ---------- ---------- ---------- ---------- ---------- 198**

**He185/333_gDNA_0027 ---------- ---------- ---------- ---------- ---------- ---------- ---------- ---------- ---------- ---------- ---------- ---------- ---------- ---------- ---------- ---------- 187**

**He185/333_gDNA_0028 ---------- ---------- ---------- ---------- ---------- ---------- ---------- ---------- ---------- ---------- ---------- ---------- ---------- ---------- ---------- ---------- 185**

**He185/333_gDNA_0029 ---------- ---------- ---------- ---------- ---------- ---------- ---------- ---------- ---------- ---------- ---------- ---------- ---------- ---------- ---------- ---------- 185**

**He185/333_gDNA_0030 ---------- ---------- ---------- ---------- ---------- ---------- ---------- ---------- ---------- ---------- ---------- ---------- ---------- ---------- ---------- ---------- 198**

**He185/333_gDNA_0031 ---------- ---------- ---------- ---------- ---------- ---------- ---------- ---------- ---------- ---------- ---------- ---------- ---------- ---------- ---------- ---------- 187**

**He185/333_gDNA_0032 ---------- ---------- ---------- ---------- ---------- ---------- ---------- ---------- ---------- ---------- ---------- ---------- ---------- ---------- ---------- ---------- 185**

**He185/333_gDNA_0033 ---------- ---------- ---------- ---------- ---------- ---------- ---------- ---------- ---------- ---------- ---------- ---------- ---------- ---------- ---------- ---------- 198**

**He185/333_gDNA_0034 ---------- ---------- ---------- ---------- ---------- ---------- ---------- ---------- ---------- ---------- ---------- ---------- ---------- ---------- ---------- ---------- 198**

**He185/333_gDNA_0035 ---------- ---------- ---------- ---------- ---------- ---------- ---------- ---------- ---------- ---------- ---------- ---------- ---------- ---------- ---------- ---------- 185**

**He185/333_gDNA_0036 ---------- ---------- ---------- ---------- ---------- ---------- ---------- ---------- ---------- ---------- ---------- ---------- ---------- ---------- ---------- ---------- 187**

**He185/333_gDNA_0037 CTGGATGGTA TTCGTTCCCT CATGAAACGC CGAACATGCC CCGGTGCAAA CAGCTGAAAA TACAATGGCG TCTTATTTTG TTTACAAGTG ACTGATTCCT TTATTTTTTT AACGCTGCAT TTTTCATCAA AATAATACCA TACACGAAGT TAAGAGTGAG 789**

**He185/333_gDNA_0038 CTGGATGGTA TTCGTTCCCT CATGAAACGC CGAACATGCC CCGGTGCAAA CAGCTGAAAA TACAATGGCG TCTTATTTTG TTTACAAGTG ACTGATTCCT TTATTTTTTT AACGTTGCAT TTTTCATCAA AATAATACCA TACACGAAGT TAAGAGTGAG 789**

**He185/333_gDNA_0039 ---------- ---------- ---------- ---------- ---------- ---------- ---------- ---------- ---------- ---------- ---------- ---------- ---------- ---------- ---------- ---------- 198**

**....|....| ....|....| ....|....| ....|....| ....|....| ....|....| ....|....| ....|....| ....|....| ....|....| ....|....| ....|....| ....|....| ....|....| ....|....| ....|....|**

i3

**805 815 825 835 845 855 865 875 885 895 905 915 925 935 945 955**

**He185/333_gDNA_0001 ---------- ---------- ---------- ---------- ---------- ---------- ---------- ---------- ---------- ---------- ---------- ---------- ---------- ---------- ---------- ---------- 187**

**He185/333_gDNA_0002 ---------- ---------- ---------- ---------- ---------- ---------- ---------- ---------- ---------- ---------- ---------- ---------- ---------- ---------- ---------- ---------- 187**

**He185/333_gDNA_0003 ---------- ---------- ---------- ---------- ---------- ---------- ---------- ---------- ---------- ---------- ---------- ---------- ---------- ---------- ---------- ---------- 187**

**He185/333_gDNA_0004 ---------- ---------- ---------- ---------- ---------- ---------- ---------- ---------- ---------- ---------- ---------- ---------- ---------- ---------- ---------- ---------- 187**

**He185/333_gDNA_0005 ---------- ---------- ---------- ---------- ---------- ---------- ---------- ---------- ---------- ---------- ---------- ---------- ---------- ---------- ---------- ---------- 187**

**He185/333_gDNA_0006 ---------- ---------- ---------- ---------- ---------- ---------- ---------- ---------- ---------- ---------- ---------- ---------- ---------- ---------- ---------- ---------- 187**

**He185/333_gDNA_0007 ---------- ---------- ---------- ---------- ---------- ---------- ---------- ---------- ---------- ---------- ---------- ---------- ---------- ---------- ---------- ---------- 187**

**He185/333_gDNA_0008 ---------- ---------- ---------- ---------- ---------- ---------- ---------- ---------- ---------- ---------- ---------- ---------- ---------- ---------- ---------- ---------- 187**

**He185/333_gDNA_0009 ---------- ---------- ---------- ---------- ---------- ---------- ---------- ---------- ---------- ---------- ---------- ---------- ---------- ---------- ---------- ---------- 198**

**He185/333_gDNA_0010 ---------- ---------- ---------- ---------- ---------- ---------- ---------- ---------- ---------- ---------- ---------- ---------- ---------- ---------- ---------- ---------- 187**

**He185/333_gDNA_0011 ---------- ---------- ---------- ---------- ---------- ---------- ---------- ---------- ---------- ---------- ---------- ---------- ---------- ---------- ---------- ---------- 187**

**He185/333_gDNA_0012 ---------- ---------- ---------- ---------- ---------- ---------- ---------- ---------- ---------- ---------- ---------- ---------- ---------- ---------- ---------- ---------- 187**

**He185/333_gDNA_0013 ---------- ---------- ---------- ---------- ---------- ---------- ---------- ---------- ---------- ---------- ---------- ---------- ---------- ---------- ---------- ---------- 187**

**He185/333_gDNA_0014 ---------- ---------- ---------- ---------- ---------- ---------- ---------- ---------- ---------- ---------- ---------- ---------- ---------- ---------- ---------- ---------- 185**

**He185/333_gDNA_0015 ---------- ---------- ---------- ---------- ---------- ---------- ---------- ---------- ---------- ---------- ---------- ---------- ---------- ---------- ---------- ---------- 187**

**He185/333_gDNA_0016 ---------- ---------- ---------- ---------- ---------- ---------- ---------- ---------- ---------- ---------- ---------- ---------- ---------- ---------- ---------- ---------- 185**

**He185/333_gDNA_0017 ---------- ---------- ---------- ---------- ---------- ---------- ---------- ---------- ---------- ---------- ---------- ---------- ---------- ---------- ---------- ---------- 185**

**He185/333_gDNA_0018 ---------- ---------- ---------- ---------- ---------- ---------- ---------- ---------- ---------- ---------- ---------- ---------- ---------- ---------- ---------- ---------- 185**

**He185/333_gDNA_0019 ---------- ---------- ---------- ---------- ---------- ---------- ---------- ---------- ---------- ---------- ---------- ---------- ---------- ---------- ---------- ---------- 198**

**He185/333_gDNA_0020 ---------- ---------- ---------- ---------- ---------- ---------- ---------- ---------- ---------- ---------- ---------- ---------- ---------- ---------- ---------- ---------- 198**

**He185/333_gDNA_0021 ---------- ---------- ---------- ---------- ---------- ---------- ---------- ---------- ---------- ---------- ---------- ---------- ---------- ---------- ---------- ---------- 185**

**He185/333_gDNA_0022 ---------- ---------- ---------- ---------- ---------- ---------- ---------- ---------- ---------- ---------- ---------- ---------- ---------- ---------- ---------- ---------- 198**

**He185/333_gDNA_0023 ---------- ---------- ---------- ---------- ---------- ---------- ---------- ---------- ---------- ---------- ---------- ---------- ---------- ---------- ---------- ---------- 185**

**He185/333_gDNA_0024 ---------- ---------- ---------- ---------- ---------- ---------- ---------- ---------- ---------- ---------- ---------- ---------- ---------- ---------- ---------- ---------- 187**

**He185/333_gDNA_0025 ---------- ---------- ---------- ---------- ---------- ---------- ---------- ---------- ---------- ---------- ---------- ---------- ---------- ---------- ---------- ---------- 185**

**He185/333_gDNA_0026 ---------- ---------- ---------- ---------- ---------- ---------- ---------- ---------- ---------- ---------- ---------- ---------- ---------- ---------- ---------- ---------- 198**

**He185/333_gDNA_0027 ---------- ---------- ---------- ---------- ---------- ---------- ---------- ---------- ---------- ---------- ---------- ---------- ---------- ---------- ---------- ---------- 187**

**He185/333_gDNA_0028 ---------- ---------- ---------- ---------- ---------- ---------- ---------- ---------- ---------- ---------- ---------- ---------- ---------- ---------- ---------- ---------- 185**

**He185/333_gDNA_0029 ---------- ---------- ---------- ---------- ---------- ---------- ---------- ---------- ---------- ---------- ---------- ---------- ---------- ---------- ---------- ---------- 185**

**He185/333_gDNA_0030 ---------- ---------- ---------- ---------- ---------- ---------- ---------- ---------- ---------- ---------- ---------- ---------- ---------- ---------- ---------- ---------- 198**

**He185/333_gDNA_0031 ---------- ---------- ---------- ---------- ---------- ---------- ---------- ---------- ---------- ---------- ---------- ---------- ---------- ---------- ---------- ---------- 187**

**He185/333_gDNA_0032 ---------- ---------- ---------- ---------- ---------- ---------- ---------- ---------- ---------- ---------- ---------- ---------- ---------- ---------- ---------- ---------- 185**

**He185/333_gDNA_0033 ---------- ---------- ---------- ---------- ---------- ---------- ---------- ---------- ---------- ---------- ---------- ---------- ---------- ---------- ---------- ---------- 198**

**He185/333_gDNA_0034 ---------- ---------- ---------- ---------- ---------- ---------- ---------- ---------- ---------- ---------- ---------- ---------- ---------- ---------- ---------- ---------- 198**

**He185/333_gDNA_0035 ---------- ---------- ---------- ---------- ---------- ---------- ---------- ---------- ---------- ---------- ---------- ---------- ---------- ---------- ---------- ---------- 185**

**He185/333_gDNA_0036 ---------- ---------- ---------- ---------- ---------- ---------- ---------- ---------- ---------- ---------- ---------- ---------- ---------- ---------- ---------- ---------- 187**

**He185/333_gDNA_0037 GCAACAGACT AACATTAAGA CTGACGCTAG TCAAGTAAGA GAAATAAGTG AAGAGGAGAT GACAAAACAG GCACTGAAAA ATGTGTATAA TGTTATGGGA CATCGTCTGC TTAGACAGTA CTCGCCCCTC TTGTGTCGTC ACACATCTCA GCAGCGTTCA 949**

**He185/333_gDNA_0038 GCAACAGACT AACATTAAGA CTGACGCTAG TCAAGTAAGA GAAATAAGTG AAGAGGAGAT GACAAAACAG GCACTGAAAA ATGTGTATAA TGTTATGGGA CATCGTCTGC TTAGACAGTA CTCGCCCCTC TTGTGTCGTC ACACATCTCA GCAGCGTTCA 949**

**He185/333_gDNA_0039 ---------- ---------- ---------- ---------- ---------- ---------- ---------- ---------- ---------- ---------- ---------- ---------- ---------- ---------- ---------- ---------- 198**

**....|....| ....|....| ....|....| ....|....| ....|....| ....|....| ....|....| ....|....| ....|....| ....|....| ....|....| ....|....| ....|....| ....|....| ....|....| ....|....|**

i3

**965 975 985 995 1005 1015 1025 1035 1045 1055 1065 1075 1085 1095 1105 1115**

**He185/333_gDNA_0001 ---------- ---------- ---------- ---------- ---------- ---------- ---------- ---------- ---------- ---------- ---------- ---------- ---------- ---------- ---------- ---------- 187**

**He185/333_gDNA_0002 ---------- ---------- ---------- ---------- ---------- ---------- ---------- ---------- ---------- ---------- ---------- ---------- ---------- ---------- ---------- ---------- 187**

**He185/333_gDNA_0003 ---------- ---------- ---------- ---------- ---------- ---------- ---------- ---------- ---------- ---------- ---------- ---------- ---------- ---------- ---------- ---------- 187**

**He185/333_gDNA_0004 ---------- ---------- ---------- ---------- ---------- ---------- ---------- ---------- ---------- ---------- ---------- ---------- ---------- ---------- ---------- ---------- 187**

**He185/333_gDNA_0005 ---------- ---------- ---------- ---------- ---------- ---------- ---------- ---------- ---------- ---------- ---------- ---------- ---------- ---------- ---------- ---------- 187**

**He185/333_gDNA_0006 ---------- ---------- ---------- ---------- ---------- ---------- ---------- ---------- ---------- ---------- ---------- ---------- ---------- ---------- ---------- ---------- 187**

**He185/333_gDNA_0007 ---------- ---------- ---------- ---------- ---------- ---------- ---------- ---------- ---------- ---------- ---------- ---------- ---------- ---------- ---------- ---------- 187**

**He185/333_gDNA_0008 ---------- ---------- ---------- ---------- ---------- ---------- ---------- ---------- ---------- ---------- ---------- ---------- ---------- ---------- ---------- ---------- 187**

**He185/333_gDNA_0009 ---------- ---------- ---------- ---------- ---------- ---------- ---------- ---------- ---------- ---------- ---------- ---------- ---------- ---------- ---------- ---------- 198**

**He185/333_gDNA_0010 ---------- ---------- ---------- ---------- ---------- ---------- ---------- ---------- ---------- ---------- ---------- ---------- ---------- ---------- ---------- ---------- 187**

**He185/333_gDNA_0011 ---------- ---------- ---------- ---------- ---------- ---------- ---------- ---------- ---------- ---------- ---------- ---------- ---------- ---------- ---------- ---------- 187**

**He185/333_gDNA_0012 ---------- ---------- ---------- ---------- ---------- ---------- ---------- ---------- ---------- ---------- ---------- ---------- ---------- ---------- ---------- ---------- 187**

**He185/333_gDNA_0013 ---------- ---------- ---------- ---------- ---------- ---------- ---------- ---------- ---------- ---------- ---------- ---------- ---------- ---------- ---------- ---------- 187**

**He185/333_gDNA_0014 ---------- ---------- ---------- ---------- ---------- ---------- ---------- ---------- ---------- ---------- ---------- ---------- ---------- ---------- ---------- ---------- 185**

**He185/333_gDNA_0015 ---------- ---------- ---------- ---------- ---------- ---------- ---------- ---------- ---------- ---------- ---------- ---------- ---------- ---------- ---------- ---------- 187**

**He185/333_gDNA_0016 ---------- ---------- ---------- ---------- ---------- ---------- ---------- ---------- ---------- ---------- ---------- ---------- ---------- ---------- ---------- ---------- 185**

**He185/333_gDNA_0017 ---------- ---------- ---------- ---------- ---------- ---------- ---------- ---------- ---------- ---------- ---------- ---------- ---------- ---------- ---------- ---------- 185**

**He185/333_gDNA_0018 ---------- ---------- ---------- ---------- ---------- ---------- ---------- ---------- ---------- ---------- ---------- ---------- ---------- ---------- ---------- ---------- 185**

**He185/333_gDNA_0019 ---------- ---------- ---------- ---------- ---------- ---------- ---------- ---------- ---------- ---------- ---------- ---------- ---------- ---------- ---------- ---------- 198**

**He185/333_gDNA_0020 ---------- ---------- ---------- ---------- ---------- ---------- ---------- ---------- ---------- ---------- ---------- ---------- ---------- ---------- ---------- ---------- 198**

**He185/333_gDNA_0021 ---------- ---------- ---------- ---------- ---------- ---------- ---------- ---------- ---------- ---------- ---------- ---------- ---------- ---------- ---------- ---------- 185**

**He185/333_gDNA_0022 ---------- ---------- ---------- ---------- ---------- ---------- ---------- ---------- ---------- ---------- ---------- ---------- ---------- ---------- ---------- ---------- 198**

**He185/333_gDNA_0023 ---------- ---------- ---------- ---------- ---------- ---------- ---------- ---------- ---------- ---------- ---------- ---------- ---------- ---------- ---------- ---------- 185**

**He185/333_gDNA_0024 ---------- ---------- ---------- ---------- ---------- ---------- ---------- ---------- ---------- ---------- ---------- ---------- ---------- ---------- ---------- ---------- 187**

**He185/333_gDNA_0025 ---------- ---------- ---------- ---------- ---------- ---------- ---------- ---------- ---------- ---------- ---------- ---------- ---------- ---------- ---------- ---------- 185**

**He185/333_gDNA_0026 ---------- ---------- ---------- ---------- ---------- ---------- ---------- ---------- ---------- ---------- ---------- ---------- ---------- ---------- ---------- ---------- 198**

**He185/333_gDNA_0027 ---------- ---------- ---------- ---------- ---------- ---------- ---------- ---------- ---------- ---------- ---------- ---------- ---------- ---------- ---------- ---------- 187**

**He185/333_gDNA_0028 ---------- ---------- ---------- ---------- ---------- ---------- ---------- ---------- ---------- ---------- ---------- ---------- ---------- ---------- ---------- ---------- 185**

**He185/333_gDNA_0029 ---------- ---------- ---------- ---------- ---------- ---------- ---------- ---------- ---------- ---------- ---------- ---------- ---------- ---------- ---------- ---------- 185**

**He185/333_gDNA_0030 ---------- ---------- ---------- ---------- ---------- ---------- ---------- ---------- ---------- ---------- ---------- ---------- ---------- ---------- ---------- ---------- 198**

**He185/333_gDNA_0031 ---------- ---------- ---------- ---------- ---------- ---------- ---------- ---------- ---------- ---------- ---------- ---------- ---------- ---------- ---------- ---------- 187**

**He185/333_gDNA_0032 ---------- ---------- ---------- ---------- ---------- ---------- ---------- ---------- ---------- ---------- ---------- ---------- ---------- ---------- ---------- ---------- 185**

**He185/333_gDNA_0033 ---------- ---------- ---------- ---------- ---------- ---------- ---------- ---------- ---------- ---------- ---------- ---------- ---------- ---------- ---------- ---------- 198**

**He185/333_gDNA_0034 ---------- ---------- ---------- ---------- ---------- ---------- ---------- ---------- ---------- ---------- ---------- ---------- ---------- ---------- ---------- ---------- 198**

**He185/333_gDNA_0035 ---------- ---------- ---------- ---------- ---------- ---------- ---------- ---------- ---------- ---------- ---------- ---------- ---------- ---------- ---------- ---------- 185**

**He185/333_gDNA_0036 ---------- ---------- ---------- ---------- ---------- ---------- ---------- ---------- ---------- ---------- ---------- ---------- ---------- ---------- ---------- ---------- 187**

**He185/333_gDNA_0037 AGGAGCTTTT CAGCTGCTAT ATCGGGGGCT GTATTTTACC GTTTTATAAT TGATAATAAT CATTAATTCA AATGTTTCCA AGCTTTAAAT TTTGGATAAC CTTGTTTAAT TTATGCCATC TATGCAATAA TAAGTATTAA GCCAATATTT AAAATCATTA 1109**

**He185/333_gDNA_0038 AGGAGCTTTT CAGCTGCTAT ATCGGGGGCT GTATTTTACC GTTTTATAAT TGATAATAAT CATTAATTCA AATGTTTCCA AGCTTTAAAT TTTGGATAAC CTTGTTTAAT TTATGCCATC TATGCAATAA TAAGTATTAA GCCAATATTT AAAATCATTA 1109**

**He185/333_gDNA_0039 ---------- ---------- ---------- ---------- ---------- ---------- ---------- ---------- ---------- ---------- ---------- ---------- ---------- ---------- ---------- ---------- 198**

**....|....| ....|....| ....|....| ....|....| ....|....| ....|....| ....|....| ....|....| ....|....| ....|....| ....|....| ....|....| ....|....| ....|....| ....|....| ....|....|**

i4

i5

i3

**1125 1135 1145 1155 1165 1175 1185 1195 1205 1215 1225 1235 1245 1255 1265 1275**

**He185/333_gDNA_0001 ---------- -----GACGT GCTATTTTAT CCATGTCTAT TCCTTGATTG CTAAAACATA ATGGATATGA TTA-TTTTTT TCTTTCTTGC TTAGCTGCTA ACATTCGTGT TCATATAGAA C--------- ---------- ---------- ---------- 292**

**He185/333_gDNA_0002 ---------- -----GACGT GCTATTTTAT CCATGTCTAT TCCTTGATTG CTAAAACATA ATGAATATGA TTA--TTTTT TCTTTCTTGC TTAGCTGCTA ACATTCGTGT TGATATAGAA C--------- ---------- ---------- ---------- 291**

**He185/333_gDNA_0003 ---------- -----GACGT GCTATTTTAT CCATGTCTAT TCCTTGATTG CTAAAACATA ATGAATATGA TTA--TTTTT TCTTTCTTGC TTAGCTGCTA ACATTCGTGT TGATATAGAA C--------- ---------- ---------- ---------- 291**

**He185/333_gDNA_0004 ---------- -----GACGT GCTATTTTAT CAATGTCTAT TCCTTGATTG CTAAAACATA AAGGGTATGA TTATTTTTTT TCTTTCTTGC TTAGCTGCTA ACATTCCTGT TCATATCGAA C--------- ---------- ---------- ---------- 293**

**He185/333_gDNA_0005 ---------- -----GACGT GCTATTTTAT CCATGTCTAT TCCTTGATTG CTAAAACATA ATGGATATGA TTA-TTTTTT TCTTTCTTGC TTAGCTGCTA ACATTCGTGT TCATATAGAA C--------- ---------- ---------- ---------- 292**

**He185/333_gDNA_0006 ---------- -----GACGT GCTATTTTAT CCATGTCTAT TCCTTGATTG CTAAAACATA ATGGATATGA TTA-TTTTTT TCTTTCTTGC TTAGCTGCTA ACATTCGTGT TCATATAGAA C--------- ---------- ---------- ---------- 292**

**He185/333_gDNA_0007 ---------- -----GACGT GCTATTTTAT CAATGTCTAT TCCTTGATTG CTAAAACATA AAGGGTATGA TTATTTTTTT TCTTTCTTGC TTAGCTGCTA ACATTCCTGT TCATATCGAA C--------- ---------- ---------- ---------- 293**

**He185/333_gDNA_0008 ---------- -----GACGT GCTATTTTAT CCATGTCTAT TCCTTGATTG CTAAAACATA ATGAATATGA TTA--TTTTT TCTTTCTTGC TTAGCTGCTA ACATTCGTGT TGATATAGAA C--------- ---------- ---------- ---------- 291**

**He185/333_gDNA_0009 ---------- -----GACGT GCTATTTTAT CAATGTCTAT TCCTTGATTG CTAAAACATA AAGGATATGA TTA--TTTTT T-TTTCTTGC TTAGCTGCTA ACATTCCTGT TCATATCGAA C--------- ---------- ---------- ---------- 301**

**He185/333_gDNA_0010 ---------- -----GACGT GCTATTTTAT CCGTGTCTAT TCCTTGATTG CTAAAACATA ATGGATATGA TTA-TTTTTT TCTTTCTTGC TTAGCTGCTA ACATTCGTGT TCATATAGGA C--------- ---------- ---------- ---------- 292**

**He185/333_gDNA_0011 ---------- -----GACGT GCTATTTTAT CCATGTCTAT TCCTTGATTG CTAAAACATA ATGGATATGA TTA-TTTTTT TCTTTCTTGC TTAGCTGCTG ACATTCGTGT TCATATAGAA C--------- ---------- ---------- ---------- 292**

**He185/333_gDNA_0012 ---------- -----GACGT GCTATTTTAT CCATGTCTAT CCCTTGATTG CTAAAACATA ATGGATATGA TTA-TTTTTT TCTTTCTTGC TTAGCTGCTA ACATTCCTGT TCATATCGAA C--------- ---------- ---------- ---------- 292**

**He185/333_gDNA_0013 ---------- -----GACGT GCTATTTTAT CCATGTCTAT TCCTTGATTG CTAAAACATA ATGGATATGA TTA-TTTTTT TCTTTCTTGC TTAGCTGCTA ACATTCGTGT TCATATAGAA C--------- ---------- ---------- ---------- 292**

**He185/333_gDNA_0014 ---------- -----GACGT GCTATT--AT CCATGTCTAT TCCTTGATTG CTATAACATA ATGGATATGA TT----TTTT TCTTT-TTGC TTAGCCGCTA ACATTCCTGT TCATATCAAG CGAAAAAAAA AGAAAGAAA- ----TTTGAC CAATTCATCT 318**

**He185/333_gDNA_0015 ---------- -----GACGT GCTATTTTAT CAATGTCTAT TCCTTGATTG CTAAAACATA AAGGGTATGA TTATTTTTTT TCTTTCTTGC TTAGCTGCTA ACATTCCTGT TCATATCGAA C--------- ---------- ---------- ---------- 293**

**He185/333_gDNA_0016 ---------- -----GACGT GCTATT--AT CCATGTCTAT TCCTTGATTG CTATAACATA ATGGATATGA TT----TTTT TCTTT-TTGC TTAGCCGCTA ACATTCCTGT TCATATCAAG CGAAAAAAAA AGAAAGAAA- ----TTTGAC CAATTCATCT 318**

**He185/333_gDNA_0017 ---------- -----GACGT GCTATT--AT CCATGTCTAT TCCTTGATTG CTATAACATA ATGGATATGA TT----TTTT TCTTT-TTGC TTAGCCGCTA ACATTCCTGT TCATATCAAG CGAAAAAAAA AGAAAGAAA- ----TTTGAC CAATTCATCT 318**

**He185/333_gDNA_0018 ---------- -----GACGT GCTATT--AT CCATGTCTAT TCCTTGATTG CTATAACATA ATGGATATGA TT----TTTT TCTTT-TTGC TTAGCCGCTA ACATTCCTGT TCATATCAAG CGAAAAAAAA AGAAAGAAA- ----TTTGAC CAATTCATCT 318**

**He185/333_gDNA_0019 ---------- -----GACGT GCTATTTTAT CAATGTCTAT TCCTTGATTG CTAAAACATA AAGGATATGA TTA--TTTTT T-TTTCTTGC TTAGCTGCTA ACATTCCTGT TCATATCGAA C--------- ---------- ---------- ---------- 301**

**He185/333_gDNA_0020 ---------- -----GACGT GCTATTTTAT CAATGTCTAT TCCTTGATTG CTAAAACATA AAGGATATGA TTA--TTTTT T-TTTCTTGC TTAGCTGCTA ACATTCCTGT TCATATCGAA C--------- ---------- ---------- ---------- 301**

**He185/333_gDNA_0021 ---------- -----GACGT GCTATT--AT CCATGTCTAT TCCTTGATTG CTATAACATA ATGGATATGA TT----TTTT TCTTT-TTGC TTAGCCGCTA ACATTCCTGT TCATATCAAG CGAAAAAAAA AGAAAGAAA- ----TTTGAC CAATTCATCT 318**

**He185/333_gDNA_0022 ---------- -----GACGT GCTATTTTAT CAATGTCTAT TCCTTGATTG CTAAAACATA AAGGATATGA TTA--TTTTT T-TTTCTTGC TTAGCTGCTA ACATTCCTGT TCATATCGAA C--------- ---------- ---------- ---------- 301**

**He185/333_gDNA_0023 ---------- -----GACGT GCTATT--AT CCATGTCTAT TCCTTGATTG CTATAACATA ATGGATATGA TT----TTTT TCTTT-TTGC TTAGCCGCTA ACATTCCTGT TCATATCAAG CGAAAAAAAA AGAAAGAAA- ----TTTGAC CAATTCATCT 318**

**He185/333_gDNA_0024 ---------- -----GACGT GCTATTTTAT CCATGTCTAT TCCTTGATTG CTAAAAGATG ATGGATATGA TTA-TTTTTT TCTTTCTTGC TTAGCTGCTA ACATTCCTGT TCATATCGAG CAAAAAAAAA -GAAAGAAAT AAAATTTTAC CAATTCACTA 330**

**He185/333_gDNA_0025 ---------- -----GACGT GCTATT--AT CCATGTCTAT TCCTTGATTG CTATAACATA ATGGATATGA TT----TTTT TCTTT-TTGC TTAGCCGCTA ACATTCCTGT TCATATCAAG CGAAAAAAAA AGAAAGAAA- ----TTTGAC CAATTCATCT 318**

**He185/333_gDNA_0026 ---------- -----GACGT GCTATTTTAT CAATGTCTAT TCCTTGATTG CTAAAACATA AAGGATATGA TTA--TTTTT T-TTTCTTGC TTAGCTGCTA ACATTCCTGT TCATATCGAA C--------- ---------- ---------- ---------- 301**

**He185/333_gDNA_0027 ---------- -----GACGT GCTATTTTAT CCATGTCTAT TCCTTGATTG CTAAAAGATG ATGGATATGA TTA-TTTTTT TCTTTCTTGC TTAGCTGCTA ACATTCCTGT TCATATCGAG CAAAAAAAAA -GAAAGAAAT AAAATTTTAC CAATTCACTA 330**

**He185/333_gDNA_0028 ---------- -----GACGT GCTATT--AT CCATGTCTAT TCCTTGATTG CTATAACATA ATGGATATGA TT----TTTT TCTTT-TTGC TTAGCCGCTA ACATTCCTGT TCATATCAAG CGAAAAAAAA AGAAAGAAA- ----TTTGAC CAATTCATCT 313**

**He185/333_gDNA_0029 ---------- -----GACGT GCTATT--AT CCATGTCTAT TCCTTGATTG CTATAACATA ATGGATATGA TT----TTTT TCTTT-TTGC TTAGCCGCTA ACATTCCTGT TCATATCAAG CGAAAAAAAA AGAAAGAAA- ----TTTGAC CAATTCATCT 318**

**He185/333_gDNA_0030 ---------- -----GACGT GCTATTTTAT CAATGTCTAT TCCTTGATTG CTAAAACATA AAGGATATGA TTA--TTTTT T-TTTCTTGC TTAGCTGCTA ACATTCCTGT TCATATCGAA C--------- ---------- ---------- ---------- 301**

**He185/333_gDNA_0031 ---------- -----GACGT GCTATTTTAT CCATGTCTAT TCCTTGATTG CTAAAAGATG ATGGATATGA TTA-TTTTTT TCTTTCTTGC TTAGCTGCTA ACATTCCTGT TCATATCGAG CAAAAAAAAA -GAAAGAAAT AAAATTTTAC CAATTCACTA 330**

**He185/333_gDNA_0032 ---------- -----GACGT GCTATT--AT CCATGTCTAT TCCTTGATTG CTATAACATA ATGGATATGA TT----TTTT TCTTT-TTGC TTAGCCGCTA ACATTCCTGT TCATATCAAG CGAAAAAAAA -GAAAGAAA- ----TTTGAC CAATTCATCT 317**

**He185/333_gDNA_0033 ---------- -----GACGT GCTATTTTAT CAATGTCTAT TCCTTGATTG CTAAAACATA AAGGATATGA TTA--TTTTT T-TTTCTTGC TTAGCTGCTA ACATTCATGT TCATATCGAA C--------- ---------- ---------- ---------- 301**

**He185/333_gDNA_0034 ---------- -----GACGT GCTATTTTAT CAATGTCTAT TCCTTGATTG CTAAAACATA AAGGATATGA TTA--TTTTT T-TTTCTTGC TTAGCTGCTA ACATTCCTGT TCATATCGAA C--------- ---------- ---------- ---------- 301**

**He185/333_gDNA_0035 ---------- -----GACGT GCTATT--AT CCATGTCTAT TCCTTGATTG CTATAACATA ATGGATATGA TT----TTTT TCTTT-TTGC TTAGCCGCTA ACATTCCTGT TCATATCAAG CGAAAAAAAA AGAAAGAAA- ----TTTGAC CAATTCATCT 318**

**He185/333_gDNA_0036 ---------- -----GACGT GCTATTTTAT CCATGTCTAT TCCTTGATTG CTAAAAGATG ATGGATATGA TTA-TTTTTT TCTTTCTTGC TTAGCTGCTA ACATTCCTGT TCATATCGAG CAAAAAAAAA -GAAAGAAAT AAAATTTTAC CAATTCACTA 330**

**He185/333_gDNA_0037 GTGTTCTACT TTAACGACGT GCTATTTTAT CAATGTCTAT TCCTTGATTG CTAAAACATA AAGGGTATGA TTATTTTTTT TCTTTCTTGC TTAGCTGCTA ACATTCCTGT TCATATCGAA C--------- ---------- ---------- ---------- 1230**

**He185/333_gDNA_0038 GTGTTCTACT TTAACGACGT GCTATTTTAT CAATGTCTAT TCCTTGATTG CTAAAACATA AAGGGTATGA TTATTTTTTT TCTTTCTTGC TTAGCTGCTA ACATTCCTGT TCATATCGAA C--------- ---------- ---------- ---------- 1230**

**He185/333_gDNA_0039 ---------- -----GACGT GCTATTTTAT CAATGTCTAT TCCTTGATTG CTAAAACATA AAGGATATGA TTA-TTTTTT T--TTCTTGC TTAGCTGCTA ACATTCCTGT TCATATCGAA C--------- ---------- ---------- ---------- 301**

**....|....| ....|....| ....|....| ....|....| ....|....| ....|....| ....|....| ....|....| ....|....| ....|....| ....|....| ....|....| ....|....| ....|....| ....|....| ....|....|**

i6

i7

i8

i5

**1285 1295 1305 1315 1325 1335 1345 1355 1365 1375 1385 1395 1405 1415 1425 1435**

**He185/333_gDNA_0001 --------TT TGAATTGCAA TTGAAATATC TATTT-TTT- TT--AAAAGG TACATTAAGG TTAGTCATCT TAAGAAACTA GAAACAAAA- ---------- --------GA TCTGCACGCA GATATAATAA TATCCTATAT ATAGTTTCAT TCAGTTTCAT 421**

**He185/333_gDNA_0002 --------TT TGAATTGCAA TTGAAATATC TATTT-TTT- TT--AAAAGG TACATTAAGG TTAGTCATCT TAAGAAACTA GAAACAAAA- ---------- --------GA TCTGCACGCA GATATAATAA TATCCTATAT ATAGTTTCAT TCAGTTTCAT 420**

**He185/333_gDNA_0003 --------TT TGAATTGCAA TTGAAATATC TATTT-TTT- TT--AAAAGG TACATTAAGG TTAGTCATCT TAAGAAACTA GAAACAAAA- ---------- --------GA CCTGCACGCA GATATAATAA TATCCTATAT ATAGTTTCAT TCAGTTTCAT 420**

**He185/333_gDNA_0004 --------TT TGAATTGCAA GTGAAATATC TATTT-TTT- T---AAAAGG TACATTTAGG TTAGTCATCT TAAGAAACTA GAAAGAAAA- ---------- --------GA TCTGCACACA GATATAATAA TATCCTATAT ATAGTTTCAT TCAGTTTCAT 421**

**He185/333_gDNA_0005 --------TT TGAATTGCAA TTGAAATATC TATTT-TTT- TT--AAAAGG TACATTAAGG TTAGTCATCT TAAGAAACTA GAAACAAAA- ---------- --------GA TCTGCACGCA GATATAATAA TATCCTATAT ATAGTTTCAT TCAGTTTCAT 421**

**He185/333_gDNA_0006 --------TT TGAATTGCAA TTGAAATATC TATTT-TTT- TT--AAAAGG TACATTAAGG TTAGTCATCT TAAGAAACTA GAAACAAAA- ---------- --------GA TCTGCACGCA GATATAATAA TATCCTATAT ATAGTTTCAT TCAGTTTCAT 421**

**He185/333_gDNA_0007 --------TT TGAATTGCAA GTGAAATATC TATTT-TTT- T---AAAAGG TACATTTAGG TTAGTCATCT TAAGAAACTA GAAAGAAAA- ---------- --------GA TCTGCACACA GATATAATAA TATCCTATAT ATAGTTTCAT TCAGTTTCAT 421**

**He185/333_gDNA_0008 --------TT TGAATTGCAA TTGAAATATC TATTT-TTT- TT--AAAAGG TACATTAAGG TTAGTCATCT TAAGAAACTA GAAACAAAA- ---------- --------GA TCTGCACGCA GATATAATAA TATCCTATAT ATAGTTTCAT TCAGTTTCAT 420**

**He185/333_gDNA_0009 --------TT TGAATTGCAA TTGAAATATC TATTT-TTT- TT---AAAGG TACATTTAGG TTAGTCATCT TAAGAAACTA GAAAGAAAA- ---------- --------GA TCTGCACACA GTTATAATAA TATCCTATAT GTAGTTTCAT TCAGTTTCAT 429**

**He185/333_gDNA_0010 --------TT TGAATTGCAA TTGAAATATC TATTT-TTT- TT--AAAAGG TACATTAAGG TTAGTCATCT TAAGAAACTA GAAACAAAA- ---------- --------GA TCTGCACGCA GATATAATAA TATCCTATAT ATAGTTTCAT TCAGTTTCAT 421**

**He185/333_gDNA_0011 --------TT TGAATTGCAA TTGAAATATC TATTT-TTT- TT--AAAAGG TACATTAAGG TTAGTCATCT TAAGAAACTA GAAACAAAA- ---------- --------GA TCTGCACGCA GATATAATAA TATCCTATAT ATAGTTTCAT TCAGTTTCAT 421**

**He185/333_gDNA_0012 --------TT TGAATTGCAA TTGAAATATC TATTT-TTT- TT--AAAAGG TACATTAAGG TTAGTCATCT TAAGAAACTA GAAACAAAA- ---------- --------GA TCTGCACGCA GATATAATGA TATCCTATAT ATAGTTTCAT TCAGTTTCAT 421**

**He185/333_gDNA_0013 --------TT TGAATTGCAA TTGAAATATC TATTT-TTT- T---AAAAGG TACATTAAGG TTAGTCATCT TAAGAAACTA GAAACAAAA- ---------- --------GA TCTGCACGCA GATATAATAA TATCCTATAT ATAGTTTCAT TCAGTTTCAT 420**

**He185/333_gDNA_0014 TATCGAAATT TAAATCGCAA TTGAAATATC GACTTCTTTC TTGG--AAGG TACATTTAGG TTAGTCATCT TAAGAAACTA GAAACAAA-C TAGGAACAAG AAACAAAAGA TCTTCATACG GATATGATAA TATCCTATAT A--GTTTTAT TCAGTTTGAT 473**

**He185/333_gDNA_0015 --------TT TGAATTGCAA GTGAAATATC TATTT-TTT- T---AAAAGG TACATTTAGG TTAGTCATCT TAAGAAACTA GAAAGAAAA- ---------- --------GA TCTGCACACA GATATAATAA TATCCTATAT ATAGTTTCAT TCAGTTTCAT 421**

**He185/333_gDNA_0016 TATCGAAATT TAAATCGCAA TTGAAATATC GATTTCTTTC TTGG--AAGG TACATTTAGG TTAGTCATCT TAAGAAACTA GAAACAAA-C TAGGAACAAG AAACAAAAGA TCTTCATACG GATATGATAA TATCCTATAT A--GTTTTAT TCAGTTTGAT 473**

**He185/333_gDNA_0017 TATCGAAATT TAAATCGCAA TTGAAATATC GATTTCTTTC TTGG--AAGG TACATTTAGG TTAGTCATCT TAAGAAACTA GAAACAAA-C TAGGAACAAG AAACAAAAGA TCTTCATACG GATATGATAA TATCCTATAT A--GTTTTAT TCAGTTTGAT 473**

**He185/333_gDNA_0018 TATCGAAATT TAAATCGCAA TTGAAATATC GATTTCTTTC TTGG--AAGG TACATTTAGG TTAGTCATCT TAAGAAACTA GAAACAAA-C TAGGAACAAG AAACAAAAGA TCTTCATACG GATATGATAA TATCCTATAT A--GTTTTAT TCAGTTTGAT 473**

**He185/333_gDNA_0019 --------TT TGAATTGCAA TTGAAATATC TATTT-TTT- TT---AAAGG TACATTTAGG TTAGTCATCT TAAGAAACTA GAAAGAAAA- ---------- --------GA TCTGCACACA GTTATAATAA TATCCTATAT GTAGTTTCAT TCAGTTTCAT 429**

**He185/333_gDNA_0020 --------TT TGAATTGCAA TTGAAATATC TATTT-TTT- TT---AAAGG TACATTTAGG TTAGTCATCT TAAGAAACTA GAAAGAAAA- ---------- --------GA TCTGCACACA GTTATAATAA TATCCTATAT GTAGTTTCAT TCAGTTTCAT 429**

**He185/333_gDNA_0021 TATCGAAATT TAAATCGCAA TTGAAATATC GATTTCTTTC TTGG--AAGG TACATTTAGG TTAGTCATCT TAAGAAACTA GAAACAAA-C TAGGAACAAG AAACAAAAGA TCTTCATACG GATATGATAA TATCCTATAT A--GTTTTAT TCAGTTTGAT 473**

**He185/333_gDNA_0022 --------TT TGAATTGCAA TTGAAATATC TATTT-TTT- TT---AAAGG TACATTTAGG TTAGTCATCT TAAGAAACTA GAAAGAAAA- ---------- --------GA TCTGCACACA GTTATAATAA TATCCTATAT GTAGTTTCAT TCAGTTTCAT 429**

**He185/333_gDNA_0023 TATCGAAATT TAAATCGCAA TTGAAATATC GATTTCTTTC TTGG--AAGG TACATTTAGG TTAGTCATCT TAAGAAACTA GAAACAAA-C TAGGAACAAG AAACAAAAGA TCTTCATACG GATATGATAA TATCCTATAT A--GTTTTAT TCAGTTTGAT 473**

**He185/333_gDNA_0024 TATCGAACTT TGAATTGCAA TTGAAATATC TATTT-TTT- T---AAAAGG TACATTTAGG TTAGTAATCT TAAGAAACTA GAAACAAAA- ---------- --------GA TTTGCACACA GATATAATAA TATCCTATAT ATAGTTTCAT TCAGTTTCAT 460**

**He185/333_gDNA_0025 TATCGAAATT TAAATCGCAA TTGAAATATC GATTTCTTTC TTGG--AAGG TACATTTAGG TTAGTCATCT TAAGAAACTA GAAACAAA-C TAGGAACAAG AAACAAAAGA TCTTCATACG GATATGATAA TATCCTATAT A--GTTTTAT TCAGTTTGAT 473**

**He185/333_gDNA_0026 --------TT TGAATTGCAA TTGAAATATC TATTT-TTT- TT---AAAGG TACATTTAGG TTAGTCATCT TAAGAAACTA GAAAGAAAA- ---------- --------GA TCTGCACACA GTTATAATAA TATCCTATAT GTAGTTTCAT TCAGTTTCAT 429**

**He185/333_gDNA_0027 TATCGAACTT TGAATTGCAA TTGAAATATC TATTT-TTT- T---AAAAGG TACATTTAGG TTAGTAATCT TAAGAAACTA GAAACAAAA- ---------- --------GA TTTGCACACA GATATAATAA TATCCTATAT ATAGTTTCAT TCAGTTTCAT 466**

**He185/333_gDNA_0028 TATCGAAATT TAAATCGCAA TTGAAATATC GATTTCTTTC TTGG--AAGG TACATTTAGG TTAGTCATCT TAAGAAACTA GAAACAAA-C TAGGAACAAG AAACAAAAGA TCTTCATACG GATATGATAA TATCCTATAT A--GTTTTAT TCAGTTTGAT 473**

**He185/333_gDNA_0029 TATCGAAATT TAAATCGCAA TTGAAATATC GATTTCTTTC TTGG--AAGG TACATTTAGG TTAGTCATCT TAAGAAACTA GAAACAAA-C TAGGAACAAG AAACAAAAGA TCTTCATACG GATATGATAA TATCCTATAT A--GTTTTAT TCAGTTTGAT 473**

**He185/333_gDNA_0030 --------TT TGAATTGCAA TTGAAATATC TATTT-TTT- TT---AAAGG TACATTTAGG TTAGTCATCT TAAGAAACTA GAAAGAAAA- ---------- --------GA TCTGCACACA GTTATAATAA TATCCTATAT GTAGTTTCAT TCAGTTTCAT 429**

**He185/333_gDNA_0031 TATCGAACTT TGAATTGCAA TTGAAATATC TATTT-TTT- T---AAAAGG TACATTTAGG TTAGTAATCT TAAGAAACTA GAAACAAAA- ---------- --------GA TTTGCACACA GATATAATAA TATCCTATAT ATAGTTTCAT TCAGTTTCAT 466**

**He185/333_gDNA_0032 TATCGAAATT TAAATCGCAA TTGAAATATC GATTTCTTTC TTGG--AAGG TACATTTAGG TTAGTCATCT TAAGAAACTA GAAACAAA-C TAGGAACAAG AAACAAAAGA TCTTCATACG GATATGATAA TATCCTATAT A--GTTTTAT TCAGTTTGAT 472**

**He185/333_gDNA_0033 --------TT TGAATTGCAA TTGAAATATC TATTT-TTT- TT---AAAGG TACATTTAGG TTAGTCATCT TAAGAAACTA GAAAGAAAA- ---------- --------GA TCTGCACACA GTTATAATAA TATCCTATAT GTAGTTTCAT TCAGTTTCAT 429**

**He185/333_gDNA_0034 --------TT TGAATTGCAA TTGAAATATC TATTT-TTT- TT---AAAGG TACATTTAGG TTAGTCATCT TAAGAAACTA GAAAGAAAA- ---------- --------GA TCTGCACACA GTTATAATAA TATCCTATAT GTAGTTTCAT TCAGTTTCAT 429**

**He185/333_gDNA_0035 TATCGAAATT TAAATCGCAA TTGAAATATC GATTTCTTTC TTGG--AAGG TACATTTAGG TTAGTCATCT TAAGAA-CTA GAAAGAAAA- ---------- --------GA TCTGCACACA GATATAATAA TATCCTATAT ATAGTTTCAT TCAGTTTCAT 456**

**He185/333_gDNA_0036 TATCGAACTT TGAATTGCAA TTGAAATATC TATTT-TTT- T---AAAAGG TACATTTAGG TTAGTAATCT TAAGAAACTA GAAACAAAA- ---------- --------GA TTTGCACACA GATATAATAA TATCCTATAT ATAGTTTCAT TCAGTTTCAT 466**

**He185/333_gDNA_0037 --------TT TGAATTGCAA TTGAAATATC TATTT-TTT- ----AAAAGG TACATTTAGG TTAGTCATCT TAAGAA-CTA GAAAGAAAA- ---------- --------GA TCTGCACACA GATATAATAA TATCCTATAT ATAGTTTCAT TCAGTTTCAT 1356**

**He185/333_gDNA_0038 --------TT TGAATTGCAA TTGAAATATC TATTT-TTT- ----AAAAGG TACATTTAGG TTAGTCATCT TAAGAA-CTA GAAAGAAAA- ---------- --------GA TCTGCACACA GATATAATAA TATCCTATAT ATAGTTTCAT TCAGTTTCAT 1356**

**He185/333_gDNA_0039 --------TT TGAATTGCAA TTGAAATATC TATTT-TTT- TT---AAAGG TACATTTAGG TTAGTCATCT TAAGAAACTA GAAAGAAAA- ---------- --------GA TCTGCACACA GTTATAATAA TATCCTATAT GTAGTTTCAT TCAGTTTCAT 429**

**....|....| ....|....| ....|....| ....|....| ....|....| ....|....| ....|....| ....|....| ....|....| ....|....| ....|....| ....|....| ....|....| ....|....| ....|....| ....|....|**

i9

i10

Leader

i8

**1445 1455 1465 1475 1485 1495 1505 1515 1525 1535 1545 1555 1565 1575 1585 1595**

**He185/333_gDNA_0001 GATGCTACTG TTCTGACAGT TATTTTATTA ACAAAAGTGA GGCAAATTAT AACTTAC--- ---------- ---------- ----CATTTT GCATGACGAG AATTCATGAC TTTGCCTAAC AAATTCATTA AAATTTTCTT ACTATCTTAC AGTTCATGCG 554**

**He185/333_gDNA_0002 GATGCTACTG TTCTGACAGT TATTTTATTA ACAAAAGTGA GGCAAATTAT AACTTAC--- ---------- ---------- ----CATTTT GCATGACGAG AATTCATGAC TTTGCCTAAC AAATTCATTA AAATGTTCTT ACTATCTTAC AGTTCATGCG 543**

**He185/333_gDNA_0003 GATGCTACTG TTCTGACAGT TATTTTATTA ACAAAAGTGA GGCAAATTAT AACTTAC--- ---------- ---------- ----CATTTT GCATGACGAG AATTCATGAC TTTGCCTAAC AAATTCATTA AAATGTTCTT ACTATCTTAC AGTTCATGCG 543**

**He185/333_gDNA_0004 GATGCTACTG TTCTGACAGT TATTTTATTA ACAAAAGTGA GGCAAATTAT AAATTAC--- ---------- ---------- ----CATTTT GCATGACGAG AATTCATGAC TCTGCCTAAC AAGATCATAT AAA-TTTCTT ACTATCTTAC AGTTCATGCT 553**

**He185/333_gDNA_0005 GATGCTACTG CTCTGACAGT TATTTTATTA ACAAAAGTGA GGCAAATTAT AACTTAC--- ---------- ---------- ----CATTTT GCATGACGAG AATTCATGAC TTTGCCTAAC AAATTCATTA AAATTTTCTT ACTATCTTAC AGTTCATGCG 554**

**He185/333_gDNA_0006 GATGCTACTG TTCTGACAGT TATTTTATTA ACAAAAGTGA GGCAAATTGT AACTTAC--- ---------- ---------- ----CATTTT GCATGACGAG AATTCATGAC TTTGCCTAAC AAATTCATTA AAATTTTCTT ACTATCTTAC AGTTCATGCG 554**

**He185/333_gDNA_0007 GATGCTACTG TTCTGACAGT TATTTTATTA ACAAAAGTGA GGCAAATTAT AAATTAC--- ---------- ---------- ----CATTTT GCATGACGAG AATTCATGAC TCTGCCTAAC AAGATCATAT AAA-TTTCTT ACTATCTTAC AGTTCATGCT 553**

**He185/333_gDNA_0008 GATGCTACTG TTCTGACAGT TATTTTATTA ACAAAAGTGA GGCAAATTAT AACTTAC--- ---------- ---------- ----CATTTT GCATGACGAG AATTCATGAC TTTGCCTAAC AAATTCATTA AAATGTTCTT ACTATCTTAC AGTTCATGCG 553**

**He185/333_gDNA_0009 GATGCTACTG TTCTGACAGT TATTTTATTA ACAAAAGTGA GGCAAATTAT AAATTAC--- ---------- ---------- ----CATTTT GCATGACGAG AATTCATGAC TTTGCCTAAC AAAATCAT-A TAATTTTATT ACTATCTTAC AGTTCATGCA 561**

**He185/333_gDNA_0010 GATGCTACTG TTCTGACAGT TATTTTATTA ACAAAAGTGA GGCAAATTAT AACTTAC--- ---------- ---------- ----CATTTT GCATGACGAG AATTCATGAC TTTGCCTAAC AAATTCATTA AAATTTTCTT ACTATCTTAC AGTTCATGCG 554**

**He185/333_gDNA_0011 GATGCTACTG TTCTGACAGT TATTTTATTA ACAAAAGTGA GGCAAATTAT AACTTAC--- ---------- ---------- ----CATTTT GCATGACGAG AATTCATGAC TTTGCCTAAC AAATTCATTA AAATTTTCTT ACTATCTTAC AGTTCATGCG 554**

**He185/333_gDNA_0012 GATGCTACTG TTCTGACAGT TATTTTATTA ACAAAAGTGA GGCAAATTAT AACTTAC--- ---------- ---------- ----CATTTT GCATGACGAG AATTCATGAC TTTGCCTAAC AAATTCATTA AAATTTTCTT ACTATCTTAC AGTTCATGCG 554**

**He185/333_gDNA_0013 GATGCTACTG TTCTGACAGT TATTTTATTA ACAAAAGTGA GGCAAATTAT AACTTAC--- ---------- ---------- ----CATTTT GCATGACGAG AATTCATGAC TTTGCCTAAC AAATTCATTA AAATTTTCTT ACTATCTTAC AGTTCATGCG 554**

**He185/333_gDNA_0014 GATGCTATTG TTCTGACAGA CATTTTATCA ACAAAAGTGA GG-------T AACTTACATG AAATGACAAT TTGGAGTAAT ATGGCATTTT GCATGACGAG AATTCATGAC TTTGCCTAAC AAAATCAT-A TAATTTTATT ACTATCTTAC AGTTCATGCA 625**

**He185/333_gDNA_0015 GATGCTACTG TTCTGACAGT TATTTTATTA ACAAAAGTGA GGCAAATTAT AAATTAC--- ---------- ---------- ----CATTTT GCATGACGAG AATTCATGAC TCTGCCTAAC AAGATCATAT AAA-TTTCTT ACTATCTTAC AGTTCATGCT 533**

**He185/333_gDNA_0016 GATGCTATTG TTCTGACAGA CATTTTATCA ACAAAAGTGA GG-------T AACTTACATG AAATGACAAT TTGGAG---T ATGGCATTTT GCATGACGAG AATTCATGAC TTTGCCTAAC AAAATCAT-A TAATTTTATT ACTATCTTAC AGTTCATGCA 622**

**He185/333_gDNA_0017 GATGCTATTG TTCTGACAGA CATTTTATCA ACAAAAGTGA GG-------T AACTTACATG AAATGACAAT TTGGAGTAAT ATGGCATTTT GCATGACGAG AATTCATGAC TTTGCCTAAC AAAATCAT-A TAATTTTATT ACTATCTTAC AGTTCATGCA 625**

**He185/333_gDNA_0018 GATGCTATTG TTCTGACAGA CATTTTATCA ACAAAAGTGA GG-------T AACTTACATG AAATGACAAT TTGGAGTAAT ATGGCATTTT GCATGACGAG AATTCATGAC TTTGCCTAAC AAAATCAT-A TAATTTTATT ACTATCTTAC AGTTCATGCA 625**

**He185/333_gDNA_0019 GATGCTACTG TTCTGACAGT TATTTTATTA ACAAAAGTGA GGCAAATTAT AAATTAC--- ---------- ---------- ----CATTTT GCATGACGAG AATTCATGAC TTTGCCTAAC AAAATCAT-A TAATTTTATT ACTATCTTAC AGTTCATGCA 561**

**He185/333_gDNA_0020 GATGCTACTG TTCTGACAGT TATTTTATTA ACAAAAGTGA GGCAAATTAT AAATTAC--- ---------- ---------- ----CATTTT GCATGACGAG AATTCATGAC TTTGCCTAAC AAAATCAT-A TAATTTTATT ACTATCTTAC AGTTCATGCA 561**

**He185/333_gDNA_0021 GATGCTATTG TTCTGACAGA CATTTTATCA TCAAAAGTGA GG-------T AACTTACATG AAATGACAAT TTGGAGTAAT ATGGCATTTT GCATGACGAG AATTCATGAC TTTGCCTAAC AAAATCAT-A TAATTTTATT ACTATCTTAC AGTTCATGCA 625**

**He185/333_gDNA_0022 GATGCTACTG TTCTGACAGT TATTTTATTA ACAAAAGTGA GGCAAATTAT AAATTAC--- ---------- ---------- ----CATTTT GCATGACGAG AATTCATGAC TTTGCCTAAC AAAATCAT-A TAATTTTATT ACTATCTTAC AGTTCATGCA 561**

**He185/333_gDNA_0023 GATGCTATTG TTCTGACAGA CATTTTATCA ACAAAAGTGA GG-------T AACTTACATG AAATGACAAT TTGGAGTAAT ATGGCATTTT GCATGACGAG AATTCATGAC TTTGCCTAAC AAAATCAT-A TAATTTTATT ACTATCTTAC AGTTCATGCA 625**

**He185/333_gDNA_0024 GATGCTACTG TTCTGACAGT TATTTTATTA ACAAAAGTGA GGCAAATTAT AAATTAC--- ---------- ---------- ----CATTTT GCATGACGAG AATTCATGAC TTTGCCTAAC AAAATCAT-A TAATTTTCTT ACTATCTTAC AGTTCATGCG 598**

**He185/333_gDNA_0025 GATGCTATTG TTCTGACAGA CATTTTATCA ACAAAAGTGA GG-------T AACTTACATG AAATGACAAT TTGGAGTAAT ATGGCATTTT GCATGACGAG AATTCATGAC TTTGCCTAAC AAAATCAT-A TAATTTTATT ACTATCTTAC AGTTCATGCA 625**

**He185/333_gDNA_0026 GATGCTACTG TTCTGACAGT TATTTTATTA ACAAAAGTGA GGCAAATTAT AAATTAC--- ---------- ---------- ----CATTTT GCATGACGAG AATTCATGAC TTTGCCTAAC AAAATCAT-A TAATTTTATT ACTATCTTAC AGTTCATGCA 561**

**He185/333_gDNA_0027 GATGCTACTG TTCTGACAGT TATTTTATTA ACAAAAGTGA GGCAAATTAT AAATTAC--- ---------- ---------- ----CATTTT GCATGACGAG AATTCATGAC TTTGCCTAAC AAAATCAT-A TAATTTTCTT ACTATCTTAC AGTTCATGCG 598**

**He185/333_gDNA_0028 GATGCTATTG TTCTGACAGA CATTTTATCA ACAAAAGTGA GG-------T AACTTACATG AAATGACAAT TTGGAGTAAT ATGGCATTTT GCATGACGAG AATTCATGAC TTTGCCTAAC AAAATCAT-A TAATTTTATT ACTATCTTAC AGTTCATGCA 625**

**He185/333_gDNA_0029 GATGCTATTG TTCTGACAGA CATTTTATCA ACAAAAGTGA GG-------T AACTTACATG AAATGACAAT TTGGAGTAAT ATGGCATTTT GCATGACGAG AATTCATGAC TTTGCCTAAC AAAATCAT-A TAATTTTATT ACTATCTTAC AGTTCATGCA 625**

**He185/333_gDNA_0030 GATGCTACTG TTCTGACAGT TATTTTATTA ACAAAAGTGA GGCAAATTAT AAATTAC--- ---------- ---------- ----CATTTT GCATGACGAG AATTCATGAC TTTGCCTAAC AAAATCAT-A TAATTTTATT ACTATCTTAC AGTTCATGCA 561**

**He185/333_gDNA_0031 GATGCTACTG TTCTGACAGT TATTTTATTA ACAAAAGTGA GGCAAATTAT AAATTAC--- ---------- ---------- ----CATTTT GCATGACGAG AATTCATGAC TTTGCCTAAC AAAATCAT-A TAATTTTCTT ACTATCTTAC AGTTCATGCG 598**

**He185/333_gDNA_0032 GATGCTATTG TTCTGACAGA CATTTTATCA ACAAAAGTGA GG-------T AACTTACATG AAATGACAAT TTGGAGTAAT ATGGCATTTT GCATGACGAG AATTCATGAC TTTGCCTAAC AAAATCAT-A TAATTTTATT ACTATCTTAC AGTTCATGCA 624**

**He185/333_gDNA_0033 GATGCTACTG TTCTGACAGT TATTTTATTA ACAAAAGTGA GGCAAATTAT AAATTAC--- ---------- ---------- ----CATTTT GCATGACGAG AATTCATGAC TTTGCCTAAC AAAATCAT-A TAATTTTATT ACTATCTTAC AGTTCATGCA 561**

**He185/333_gDNA_0034 GATGCTACTG TTCTGACAGT TATTTTATTA ACAAAAGTGA GGCAAATTAT AAATTAC--- ---------- ---------- ----CATTTT GCATGACGAG AATTCATGAC TTTGCCTAAC AAAATCAT-A TAATTTTATT ACTATCTTAC AGTTCATGCA 561**

**He185/333_gDNA_0035 GATGCTACTG TTCTGACAGT TATTTTATTA ACAAAAGTGA GGCAAATTAT AAATTAC--- ---------- ---------- ----CATTTT GCATGACGAG AATTCATGAC TTTGCCTAAC AAAATCAT-A TAATTTTCTT ACTATCTTAC AGTTCATGCG 588**

**He185/333_gDNA_0036 GATGCTACTG TTCTGACAGT TATTTTATTA ACAAAAGTGA GGCAAATTAT AAATTAC--- ---------- ---------- ----CATTTT GCATGACGAG AATTCATGAC TTTGCCTAAC AAAATCAT-A TAATTTTATT ACTATCTTAC AGTTCATGCA 598**

**He185/333_gDNA_0037 GATGCTACTG TTCTGACAGT TATTTTATTA ACAAAAGTGA GGCAAATTAT AAATTAC--- ---------- ---------- ----CATTTT GCATGACGAG AATTCATGAC TTTGCCTAAC AAAATCAT-A TAATTTTCTT ACTATCTTAC AGTTCATGCG 1488**

**He185/333_gDNA_0038 GATGCTACTG TTCTGACAGT TATTTTATTA ACAAAAGTGA GGCAAATTAT AAATTAC--- ---------- ---------- ----CATTTT GCATGACGAG AATTCATGAC TTTGCCTAAC AAAATCAT-A TAATTTTCTT ACTATCTTAC AGTTCATGCG 1488**

**He185/333_gDNA_0039 GATGCTACTG TTCTGACAGT TATTTTATTA ACAAAAGTGA GGCAAATTAT AAATTAC--- ---------- ---------- ----CATTTT GCATGACGAG AATTCATGAC TTTGCCTAAC AAAATCAT-A TAATTTTATT ACTATCTTAC AGTTCATGCA 561**

4

6

5

**....|....| ....|....| ....|....| ....|....| ....|....| ....|....| ....|....| ....|....| ....|....| ....|....| ....|....| ....|....| ....|....| ....|....| ....|....| ....|....|**

1

2

3

7

**1605 1615 1625 1635 1645 1655 1665 1675 1685 1695 1705 1715 1725 1735 1745 1755**

**He185/333_gDNA_0001 CAAAGAGAAC CGGGAGGAAG AGGAAATGGC AGAGAGAGGG GACAAGGTCG CTTCGGAGGA AGGCCAGGAT CTGAT---AG ATCCCAAATG ATGGGTGGAC CTAGGCAAGG TGGTCCGCCA ATGGGCGGAA GGAGGTTTGA TGGCCCTGGA CAAGGTGACC 711**

**He185/333_gDNA_0002 CAAAGAGAAC CGGGAGGAAG AGGAAATGGC AGAGAGAGGG GACAAGGTCG CTTCGGAGGA AGGCCAGGAT CTGAT---AG ATCCCAAATG ATGGGTGGAC CTAGGCAAGG TGGTCCGCCA ATGGGCGGAA GGAGGTTTGA TGGCCCTGGA CAAGGTGACC 710**

**He185/333_gDNA_0003 CAAAGAGAAC CGGGAGGAAG AGGAAATGGC AGAGAGAGGG GACAAGGTCG CTTCGGAGGA AGGCCAGGAT CTGAT---AG ATCCCAAATG ATGGGTGGAC CTAGGCAAGG TGGTCCGCCA ATGGGCGGAA GGAGGTTTGA TGGCCCTGGA CAAGGTGACC 710**

**He185/333_gDNA_0004 CAAAGAGCAC AAGGAGGAAG AGGATATGGC AGAAAGAGGG GACAAGGTCG CTTCGGAGGA AGTCCAGGAT CTGAT---AG ACCCCAAATG ACGGGTGGAC CTAGGCAAGG TGGTCCACCA ATGGGCGGAA GGAGGTTTGA TAGCCCTGGA CAAGGTGACC 710**

**He185/333_gDNA_0005 CAAAGAGAAC CGGGAGGAAG AGGAAATGGC AGAGAGAGGG GACAAGGTCG CTTCGGAGGA AGGCCAGGAT CTGAT---AG ATCCCAAATG ATGGGTGGAC CTAGGCAAGG TGGTCCGCCA ATGGGCGGAA GGAGGTTTGA TGGCCCTGGA CAAGGTGACC 711**

**He185/333_gDNA_0006 CAAAGAGAAC CGGGAGGAAG AGGAAATGGC AGAGAGAGGG GACAAGGTCG CTTCGGAGGA AGGCCAGGAT CTGAT---AG ATCCCAAATG ATGGGTGGAC CTAGGCAAGG TGGTCCGCCA ATGGGCGGAA GGAGGTTTGA TGGCCCTGGA CAAGGTGACC 711**

**He185/333_gDNA_0007 CAAAGAGCAC GAGGAGGAAG AGGATATGGC AGAAAGAGGG GACAAGGTCG CTTCGGAGGA AGTCCAGGAT CTGAT---AG ACCCCAAATG ACGGGTGGAC CTAGGCAAGG TGGTCCACCA ATGGGCGGAA GGAGGTTT-- ---------- ---------- 688**

**He185/333_gDNA_0008 CAAAGAGAAC CGGGAGGAAG AGGAAATGGC AGAGAGAGGG GACAAGGTCG CTTCGGAGGA AGGCCAGGAT CTGAT---AG ATCCCAAATG ATGGGTGGAC CTAGGCAAGG TGGTCCGCCA ATGGGCGGAA GGAGGTTTGA TGGCCCTGGA CAAGGTGACC 710**

**He185/333_gDNA_0009 CAAAGAGGCC GGGGCGGAAG AGGAAATGGC AGAGAGAGGG GACAAGGTCG CTTCGGAGGA AGGCCAGGAT CC------AG ACCCCAAATG ATGGGTGGAC ATAGGCAAGG TGGTCCACCA ATGGGTGGAA GGAGGTTTGA TGGCAATGGA CAAGGTGACC 715**

**He185/333_gDNA_0010 CAAAGAGAAC CGGGAGGAAG AGGAAATGGC AGAGAGAGGG GACAAGGTCG CTTCGGAGGA AGGCCAGGAT CTGAT---AG ATCCCAAATG ATGGGTGGAC CTAGGCAAGG TGGTCCGCCA ATGGGCGGAA GGAGGTTTGA TGGCCCTGGA CAAGGTGACC 711**

**He185/333_gDNA_0011 CAAAGAGAAC CGGGAGGAAG AGGAAATGGC AGAGAGAGGG GACAAGGTCG CTTCGGAGGA AGGCCAGGAT CTGAT---AG ATCCCAAATG ATGGGTGGAC CTAGGCAAGG TGGTCCGCCA ATGGGCGGAA GGAGGTTTGA TGGCCCTGGA CAAGGTGACC 711**

**He185/333_gDNA_0012 CAAAGAGAAC CGGGAGGAAG AGGAGATGGC AGAGAGAGGG GACAAGGTCG CTTCGGAGGA AGGCCAGGAT CTGAT---AG ATCCCAAATG ATGGGTGGAC CTAGGCAAGG TGGTCCGCCA ATGGGCGGAA GGAGGTTTGA TGGCCCTGGA CAAGGTGACC 711**

**He185/333_gDNA_0013 CAAAGAGAAC CGGGAGGAAG AGGAAATGGC AGAGAGAGGG GACAAGGTCG CTTCGGAGGA AGGCCAGGAT CTGAT---AG ATCCCAAATG ATGGGTGGAC CTAGGCAAGG TGGTCCGCCA ATGGGCGGAA GGAGGTTTGA TGGCCCTGGA CAAGGTGACC 710**

**He185/333_gDNA_0014 CAAAGAGACC GGGGCGGAAG AGGAAATGGC AGAGAGAGGG GACAAGGTCG CTTTGGAGGA AGGCCAAGAT CTGATGATAG ACCCCAAATG ATGGGTGGAC ATAGGCAAGG TGGTCCACCA ATGGGCGGAA GGAGGTTTGA TGTCCCTGGA CAAGGTGACC 785**

**He185/333_gDNA_0015 CAAAGAGCAC GGGGAGGAAG AGGATATGGC AGAAAGAGGG GACAAGGTCG CTTCGGAGGA AGTCCAGGAT CTGAT---AG ACCCCAAATG ACGGGTGGAC CTAGGCAAGG TGGTCCACCA ATGGGCGGAA GGAGGTTT-- ---------- ---------- 688**

**He185/333_gDNA_0016 CAAAGAGACC GGGGCGGAAG AGGAAATGGC AGAGAGAGGG GACAAGGTCG CTTTGGAGGA AGGCCAAGAT CTGATGATAG ACCCCAAATG ATGGGTGGAC ATAGGCAAGG TGGTCCACCA ATGGGCGGAA GGAGGTTTGA TGTCCCTGGA CAAGGTGACC 782**

**He185/333_gDNA_0017 CAAAGAGACC GGGGCGGAAG AGGAAATGGC AGAGAGAGGG GACAAGGTCG CTTTGGAGGA AGGCCAAGAT CTGATGATAG ACCCCAAATG ATGGGTGGAC ATAGGCAAGG TGGTCCACCA ATGGGCGGAA GGAGGTTTGA TGTCCCTGGA CAAGGTGACC 785**

**He185/333_gDNA_0018 CAAAGAGACC GGGGCGGAAG AGGAAATGGC AGAGAGAGGG GACAAGGTCG CTTTGGAGGA AGGCCAAGAT CTGATGATAG ACCCCAAATG ATGGGTGGAC ATAGGCAAGG TGGTCCACCA ATGGGCGGAA GGAGGTTTGA TGTCCCTGGA CAAGGTGACC 785**

**He185/333_gDNA_0019 CAAAGAGGCC GGGGCGGAAG AGGAAATGGC AGAGAGAGGG GACAAGGTCG CTTCGGAGGA AGGCCAGGAT CC------AG ACCCCAAATG ATGGGTGGAC ATAGGCAAGG TGGTCCACCA ATGGGTGGAA GGAGGTTTGA TGGCAATGGA CAAGGTGACC 715**

**He185/333_gDNA_0020 CAAAGAGGCC GGGGCGGAAG AGGAAATGGC AGAGAGAGGG GACAAGGTCG CTTCGGAGGA AGGCCAGGAT CC------AG ACCCCAAATG ATGGGTGGAC ATAGGCAAGG TGGTCCACCA ATGGGTGGAA GGAGGTTTGA TGGCAATGGA CAAGGTGACC 715**

**He185/333_gDNA_0021 CAAAGAGACC GGGGCGGAAG AGGAAATGGC AGAGAGAGGG GACAAGGTCG CTTTGGAGGA AGGCCAAGAT CTGATGATAG ACCCCAAATG ATGGGTGGAC ATAGGCAAGG TGGTCCACCA ATGGGCGGAA GGAGGTTTGA TGTCCCTGGA CAAGGTGACC 785**

**He185/333_gDNA_0022 CAAAGAGGCC GGGGCGGAAG AGGAAATGGC AGAGAGAGGG GACAAGGTCG CTTCGGAGGA AGGCCAGGAT CC------AG ACCCCAAATG ATGGGTGGAC ATAGGCAAGG TGGTCCACCA ATGGGTGGAA GGAGGTTTGA TGGCAATGGA CAAGGTGACC 715**

**He185/333_gDNA_0023 CAAAGAGACC GGGGCGGAAG AGGAAATGGC AGAGAGAGGG GACAAGGTCG CTTTGGAGGA AGGCCAAGAT CTGATGATAG ACCCCAAATG ATGGGTGGAC ATAGGCAAGG TGGTCCACCA ATGGGCGGAA GGAGGTTTGA TGTCCCTGGA CAAGGTGACC 785**

**He185/333_gDNA_0024 CAAAGAGAAC CGGGAGGAAG AGGAAATGGC AGAGAGAGGG GACAAGGTCG CTTCAGAGGA AGGCCAAGAT CTGATGATAG ACCCCAAATG ATGGGTGGAC ATAGGCAAGG TGGTCCACCA ATGGGCGGAA GGAGGTTTGA TGTCCCTGGA CAAGGTGACC 758**

**He185/333_gDNA_0025 CAAAGAGACC GGGGCGGAAG AGGAAATGGC AGAGAGAGGG GACAAGGTCG CTTTGGAGGA AGGCCAAGAT CTGATGATAG ACCCCAAATG ATGGGTGGAC ATAGGCAAGG TGGTCCACCA ATGGGCGGAA GGAGGTTTGA TGTCCCTGGA CAAGGTGACC 785**

**He185/333_gDNA_0026 CAAAGAGGCC GGGGCGGAAG AGGAAATGGC AGAGAGAGGG GACAAGGTCG CTTCGGAGGA AGGCCAGGAT CC------AG ACCCCAAATG ATGGGTGGAC ATAGGCAAGG TGGTCCACCA ATGGGTGGAA GGAGGTTTGA TGGCAATGGA CAAGGTGACC 715**

**He185/333_gDNA_0027 CAAAGAGAAC CGGGAGGAAG AGGAAATGGC AGAGAGAGGG GACAAGGTCG CTTCAGAGGA AGGCCAGGAT CTGAT---AG ATCCCAAATG ATGGGTGGAC CTAGGCAAGG TGGTCCACCA ATGGGCGGAA GGAGGTTTGA TGGCCCTGGA CAAGGTGACC 755**

**He185/333_gDNA_0028 CAAAGAGACC GGGGCGGAAG AGGAAATGGC AGAGAGAGGG GACAAGGTCG CTTTGGAGGA AGGCCAAGAT CTGATGATAG ACCCCAAATG ATGGGTGGAC ATAGGCAAGG TGGTCCACCA ATGGACGGAA GGAGGTTTGA TGTCCCTGGA CAAGGTGACC 785**

**He185/333_gDNA_0029 CAAAGAGACC GGGGCGGAAG AGGAAATGGC AGAGAGAGGG GACAAGGTCG CTTTGGAGGA AGGCCAAGAT CTGATGATAG ACCCCAAATG ATGGGTGGAC ATAGGCAAGG TGGTCCACCA ATGGGCGGAA GGAGGTTTGA TGTCCCTGGA CAAGGTGACC 785**

**He185/333_gDNA_0030 CAAAGAGGCC GGGGCGGAAG AGGAAATGGC AGAGAGAGGG GACAAGGTCG CTTCGGAGGA AGGCCAGGAT CC------AG ACCCCAAATG ATGGGTGGAC ATAGGCAAGG TGGTCCACCA ATGGGTGGAA GGAGGTTTGA TGGCAATGGA CAAGGTGACC 715**

**He185/333_gDNA_0031 CAAAGAGAAC CGGGAGGAAG AGGAAATGGC AGAGAGAGGG GACAAGGTCG CTTCAGAGGA AGGCCAGGAT CTGAT---AG ATCCCAAATG ATGGGTGGAC CTAGGCAAGG TGGTCCACCA ATGGGCGGAA GGAGGTTTGA TGGCCCTGGA CAAGGTGACC 755**

**He185/333_gDNA_0032 CAAAGAGACC GGGGCGGAAG AGGAAATGGC AGAGAGAGGG GACAAGGTCG CTTTGGAGGA AGGCCAAGAT CTGATGATAG ACCCCAAATG ATGGGTGGAC ATAGGCAAGG TGGTCCACCA ATGGGCGGAA GGAGGTTTGA TGTCCCTGGA CAAGGTGACC 784**

**He185/333_gDNA_0033 CAAAGAGGCC GGGGCGGAAG AGGAAATGGC AGAGAGAGGG GACAAGGTCG CTTCGGAGGA AGGCCAGGAT CC------AG ACCCCAAATG ATGGGTGGAC ATAGGCAAGG TGGTCCACCA ATGGGTGGAA GGAGGTTTGA TGGCAATGGA CAAGGTGACC 715**

**He185/333_gDNA_0034 CAAAGAGGCC GGGGCGGAAG AGGAAATGGC AGAGAGAGGG GACAAGGTCG CTTCGGAGGA AGGCCAGGAT CC------AG ACCCCAAATG ATGGGTGGAC ATAGGCAAGG TGGTCCACCA ATGGGTGGAA GGAGGTTTGA TGGCAATGGA CAAGGTGACC 715**

**He185/333_gDNA_0035 CAAAGAGAAC CGGGAGGAAG AGGAAATGGC AGAGAGAGGG GACAAGGTCG CTTCAGAGGA AGGCCAGGAT CTGAT---AG ATCCCAAATG ATGGGTGGAC CTAGGCAAGG TGGTCCACCA TTGGGCGGAA GGAGGTTTGA TGGCCCTGGA CAAGGTGACC 745**

**He185/333_gDNA_0036 CAAAGAGACC GGGGCGGAAG AGGAAATGGC AGAGAGAGGG GACAAGGTCG CTTTGGAGGA AGGCCAAGAT CTGATGATAG ACCCCAAATG ATGGGTGGAC ATAGGCAAGG TGGTCCACCA ATGGGCGGAA GGAGGTTTGA TGTCCCTGGA CAAGGTGACC 758**

**He185/333_gDNA_0037 CAAAGAGAAC CGGGAGGAAG AGGAAATGGC AGAGAGAGGG GACAAGGTCG CTTCAGAGGA AGGCCAGGAT CTGAT---AG ATCCCAAATG ATGGGTGGAC CTAGGCAAGG TGGTCCACCA ATGGGCGGAA GGAGGTTTGA TGGCCCTGGA CAAGGTGACC 1645**

**He185/333_gDNA_0038 CAAAGAGAAC CGGGAGGAAG AGGAAATGGC AGAGAGAGGG GACAAGGTCG CTTCAGAGGA AGGCCAGGAT CTGAT---AG ATCCCAAATG ATGGGTGGAC CTAGGCAAGG TGGTCCACCA ATGGGCGGAA GGAGGTTTGA TGGCCCTGGA CAAGGTGACC 1645**

**He185/333_gDNA_0039 CAAAGAGGCC GGGGCGGAAG AGGAAATGGC AGAGAGAGGG GACAAGGTCG CTTCGGAGGA AGGCCAGGAT CC------AG ACCCCAAATG ATGGGTGGAC ATAGGCAAGG TGGTCCACCA ATGGGTGGAA GGAGGTTTGA TGGCAATGGA CAAGGTGACC 715**

8

9

11

**....|....| ....|....| ....|....| ....|....| ....|....| ....|....| ....|....| ....|....| ....|....| ....|....| ....|....| ....|....| ....|....| ....|....| ....|....| ....|....|**

12

7

10

**1765 1775 1785 1795 1805 1815 1825 1835 1845 1855 1865 1875 1885 1895 1905 1915**

**He185/333_gDNA_0001 AGCAGATGGA TGGACGTGGA CCGAATGGTG GGCCAATGGG CGGTAGGAGG TTTGATGGAC CAGGATTCGG TGGCTTCAGA CTCGAAGGTG CAGGGAGACC TTTCTTCGGT CACGGAGGAA GGCATGCTGA TGGAGAAGGA GAAATGGAGG CTGCTCAACC 871**

**He185/333_gDNA_0002 AGCAGATGGA TGGACGTGGA CCGAATGGTG GGCCAATGGG CGGTAGGAGG TTTGATGGAC CAGGATTCGG TGGCTTCAGA CTCGAAGGTG CAGGGAGACC CTTCTTCGGT CACGGAGGAA GGCATGCTGA TGGAGAAGGA GAAATGGAGG CTGCTCAACC 870**

**He185/333_gDNA_0003 AGCAGATGGA TGGACGTGGA CCGAATGGTG GGCCAATGGG CGGTAGGAGG TTTGATGGAC CAGGATTCGG TGGCTTCAGA CTCGAAGGTG CAGGGAGACC TTTCTTCGGT CACGGAGGAA GGCATGCTGA TGGAGAAGGA GAAATGGAGG CTGCTCAACC 870**

**He185/333_gDNA_0004 AACAGATGGA TGGACGTGGA CCGAATGGTG GGCCAATGGG CGGTAGGAGG TTTGATGGAC CAGGATTCGG TGGCTTCAGA CCCGAAGGTG CAGGGAGACC TTTCTTCGGT CAAGGAGGAA TGCATGCTGA TGGAGAAGGA GAAATGGAGG TTGCTCAACC 870**

**He185/333_gDNA_0005 AGCAGATGGA TGGACGTGGA CCGAATGGTG GGCCAATGGG CGGTAGGAGG TTTGATGGAC CAGGATTCGG TGGCTTCAGA CTCGAAGGTG CAGGGAGACC TTTCTTCGGT CACGGAGGAA GGCATGCTGA TGGAGAAGGA GAAATGGAGG CTGCTCAACC 871**

**He185/333_gDNA_0006 AGCAGATGGA TGGACGTGGA CCGAATGGTG GGCCAATGGG CGGTAGGAGG TTTGATGGAC CAGGATTCGG TGGCTTCAGA CTCGAAGGTG CAGGGAGACC TTTCTTCGGT CACGGAGGAA GGCATGCTGA TGGAGAAGGA GAAATGGAGG CTGCTCAACC 870**

**He185/333_gDNA_0007 ---------- ---------- ---------- ---------- ---------- ---------- ---------- ---------- ---GAAGGTG CAGGGAGACC TTTCTTCGGT CAAGGAGGAA TGCATGCTGA TGGAGAAGGA GAAATGGAGG TTGCTCAACC 765**

**He185/333_gDNA_0008 AGCAGATGGA TGGACGTGGA CCGAATGGTG GGCCAATGGG CGGTAGGAGG TTTGATGGAC CAGGATTCGG TGGCTTCAGA CTCGAAGGTG CAGGGAGACC TTTCTTCGGT CACGGAGGAA GGCATGCTGA TGGAGAAGGA GAAATGGAGG CTGCTCAACC 870**

**He185/333_gDNA_0009 AACAGATGGC TGGACGTGAA CCGAATGGCC GGCCAGTGGG CAGTAGAAAA TTTGATGGAC CAGGATTCGG TGGCTTCAGA CCCGAAGGTG CCGGGAGACC TTTCTTTGGT CACGGAGGAA TGCATGCTGA TGGAGAAGGA GAAATGGAGG TTCCTCAACC 875**

**He185/333_gDNA_0010 AGCAGATGGA TGGACGTGGA CCGAATGGTG GGCCAATGGG CGGTAGGAGG TTTGATGGAC CAGGATTCGG TGGCTTCAGA CTCGAAGGTG CAGGGAGACC TTTCTTCGGT CACGGAGGAA GGCATGCTGA TGGAGAAGGA GAAATGGAGG CTGCTCAACC 871**

**He185/333_gDNA_0011 AGCAGATGGA TGGACGTGGA CCGAATGGTG GGCCAATGGG CGGTAGGAGG TTTGATGGAC CAGGATTCGG TGGCTTCAGA CTCGAAGGTG CAGGGAGACC TTTCTTCGGT CACGGAGGAA GGCATGCTGA TGGAGAAGGA GAAATGGAGG CTGCTCAACC 871**

**He185/333_gDNA_0012 AGCAGATGGA TGGACGTGGA CCGAATGGTG GGCCAATGGG CGGTAGGAGG TTTGATGGAC CAGGATTCGG TGGCTTCAGA CTCGAAGGTG CAGGGAGACC TTTCTTCGGT CACGGAGGAA GGCATGCTGA TGGAGAAGGA GAAATGGAGG CTGCTCAACC 871**

**He185/333_gDNA_0013 AGCAGATGGA TGGACGTGGA CCGAATGGTG GGCCAATGGG CGGTAGGAGG TTTGATGGAC CAGGATTCGG TGGCTTCAGA CTCGAAGGTG CAGGGAGACC TTTCTTCGGT CACGGAGGAA GGCATGCTGA TGGAGAAGGA GAAATGGAGG CTGCTCAACC 870**

**He185/333_gDNA_0014 AAAAGATGGC TGGACGTGAA CCAAATGGCC GGCCAGTGGG CAGTAGAAAA TTTGATGGAC CAGGA-TCGG TGGCTTCAGA CCCGAAGGTG CCGGGAGACC TTTCTTTGGT CATGGAGGAA GGCATGCTGA TGGAGAAGGA GAAATGGAGG CTGCTCAACC 944**

**He185/333_gDNA_0015 ---------- ---------- ---------- ---------- ---------- ---------- ---------- ---------- ---GAAGGTG CAGGGAGACC TTTCTTCGGT CAAGGAGGAA TGCATGCTGA TGGAGAAGGA GAAATGGAGG TTGCTCAACC 765**

**He185/333_gDNA_0016 AAAAGATGGC TGGACGTGAA CCAAATGGCC GGCCAGTGGG CAGTAGAAAA TTTGATGGAC CAGGATTCGG TGGCTTCAGA CCCGAAGGTG CCGGGAGACC TTTCTTTGGT CATGGAGGAA GGCATGCTGA TGGAGAAGGA GAAATGGAGG CTGCTCAACC 942**

**He185/333_gDNA_0017 AAAAGATGGC TGGACGTGAA CCAAATGGCC GGCCAGTGGG CAGTAGAAAA TTTGATGGAC CAGGATTCGG TGGCTTCAGA CCCGAAGGTG CCGGGAGACC TTTCTTTGGT CATGGAGGAA GGCATGCTGA TGGAGAAGGA GAAATGGAGG CTGCTCAACC 945**

**He185/333_gDNA_0018 AAAAGATGGC TGGACGTGAA CCAAATGGCC GGCCAGTGGG CAGTAGAAAA TTTGATGGAC CAGGATTCGG TGGCTTCAGA CCCGAAGGTG CCGGGAGACC TTTCTTTGGT CATGGAGGAA GGCATGCTGA TGGAGAAGGA GAAATGGAGG CTGCTCAACC 945**

**He185/333_gDNA_0019 AACAGATGGC TGGACGTGAA CCGAATGGCC GGCCAGTGGG CAGTAGAAAA TTTGATGGAC CAGGATTCGG TGGCTTCAGA CCCGAAGGTG CCGGGAGACC TTTCTTTGGT CACGGAGGAA TGCATGCTGA TGGAGAAGGA GAAATGGAGG TTCCTCAACC 875**

**He185/333_gDNA_0020 AACAGATGGC TGGACGTGAA CCGAATGGCC GGCCAGTGGG CAGTAGAAAA TTTGATGGAC CAGGATTCGG TGGCTTCAGA CCCGAAGGTG CCGGGAGACC TTTCTTTGGT CACGGAGGAA TGCATGCTGA TGGAGAAGGA GAAATGGAGG TTCCTCAACC 875**

**He185/333_gDNA_0021 AAAAGATGGC TGGACGTGAA CCAAATGGCC GGCCAGTGGG CAGTAGAAAA TTTGATGGAC CAGGATTCGG TGGCTTCAGA CCCGAAGGTG CCGGGAGACC TTTCTTTGGT CATGGAGGAA GGCATGCTGA TGGAGAAGGA GAAATGGAGG CTGCTCAACC 945**

**He185/333_gDNA_0022 AACAGATGGC TGGACGTGAA CCGAATGGCC GGCCAGTGGG CAGTAGAAAA TTTGATGGAC CAGGATTCGG TGGCTTCAGA CCCGAAGGTG CCGGGAGACC TTTCTTTGGT CACGGAGGAA TGCATGCTGA TGGAGAAGGA GAAATGGAGG TTCCTCAACC 875**

**He185/333_gDNA_0023 AAAAGATGGC TGGACGTGAA CCAAATGGCC GGCCAGTGGG CAGTAGAAAA TTTGATGGAC CAGGATTCGG TGGCTTCAGA CCCGAAGGTG CCGGGAGACC TTTCTTTGGT CATGGAGGAA GGCATGCTGA TGGAGAAGGA GAAATGGAGG CTGCTCAACC 945**

**He185/333_gDNA_0024 AAAAGATGGC TGGACGTGAA CCAAATGGCC GGCCAGTGGG CAGTAGAAAA TTTGATGGAC CAGGATTCGG TGGCTTCAGA CCCGAAGGTG CCGGGAGACC TTTCTTTGGT CATGGAGGAA GGCATGCTGA TGGAGAAGGA GAAATGGAGG CTGCTCAACC 918**

**He185/333_gDNA_0025 AAAAGATGGC TGGACGTGAA CCAAATGGCC GGCCAGTGGG CAGTAGAAAA TTTGATGGAC CAGGATTCGG TGGCTTCAGA CCCGAAGGTG CCGGGAGACC TTTCTTTGGT CATGGAGGAA GGCATGCTGA TGGAGAAGGA GAAATGGAGG CTGCTCAACC 945**

**He185/333_gDNA_0026 AACAGATGGC TGGACGTGAA CCGAATGGCC GGCCAGTGGG CAGTAGAAAA TTTGATGGAC CAGGATTCGG TGGCTTCAGA CCCGAAGGTG CCGGGAGACC TTTCTTTGGT CACGGAGGAA TGCATGCTGA TGGAGAAGGA GAAATGGAGG TTCCTCAACC 875**

**He185/333_gDNA_0027 AGCAGATGGA TGGACGTGGA CCGAATGGTG GGCCAATGGG CGGTAGGAGG TTTGATGGAC CAGGATTCGG TGGCTTCAGA CCCGAAGGTG CAGGGAGACC TTTCTTCGGT CACGGAGGAA GGCATGCTGA TGGAGAAGGG GAAATGGAGG TTCCTCAACC 915**

**He185/333_gDNA_0028 AAAAGATGGC TGGACGTGAA CCAAATGGCC GGCCAGTGGG CAGTAGAAAA TTTGATGGAC CAGGATTCGG TGGCTTCAGA CCCGAAGGTG CCGGGAGACG TTTCTTTGGT CATGGAGGAA GGCATGCTGA TGGAGAAGGA GAAATGGAGG CTGCTCAACC 945**

**He185/333_gDNA_0029 AAAAGATGGC TGGACGTGAA CCAAATGGCC GGCCAGTGGG CAGTAGAAAA TTTGATGGAC CAGGATTCGG TGGCTTCAGA CCCGAAGGTG CCGGGAGACC TTTCTTTGGT CATGGAGGAA GGCATGCTGA TGGAGAAGGA GAAATGGAGG CTGCTCAACC 945**

**He185/333_gDNA_0030 AACAGATGGC TGGACGTGAA CCGAATGGCC GGCCAGTGGG CAGTAGAAAA TTTGATGGAC CAGGATTCGG TGGCTTCAGA CCCGAAGGTG CCGGGAGACC TTTCTTTGGT CACGGAGGAA TGCATGCTGA TGGAGAAGGA GAAATGGAGG TTCCTCAACC 875**

**He185/333_gDNA_0031 AGCAGATGGA TGGACGTGGA CCGAATGGTG GGCCAATGGG CGGTAGGAGG TTTGATGGAC CAGGATTCGG TGGCTTCAGA CCCGAAGGTG CAGGGAGACC TTTCTTCGGT CACGGAGGAA GGCATGCTGA TGGAGAAGGG GAAATGGAGG CTGCTCAACC 915**

**He185/333_gDNA_0032 AAAAGATGGC TGGACGTGAA CCAAATGGCC GGCCAGTGGG CAGTAGAAAA TTTGATGGAC CAGGATTCGG TGGCTTCAGA CCCGAAGGTG CCGGGAGACC TTTCTTTGGT CATGGAGGAA GGCATGCTGA TGGAGAAGGA GAAATGGAGG CTGCTCAACC 944**

**He185/333_gDNA_0033 AACAGATGGC TGGACGTGAA CCGAATGGCC GGCCAGTGGG CAGTAGAAAA TTTGATGGAC CAGGATTCGG TGGCTTCAGA CCCGAAGGTG CCGGGAGACC TTTCTTTGGT CACGGAGGAA TGCATGCTGA TGGAGAAGGA GAAATGGAGG TTCCTCAACC 875**

**He185/333_gDNA_0034 AACAGATGGC TGGACGTGAA CCGAATGGCC GGCCAGTGGG CAGTAGAAAA TTTGATGGAC CAGGATTCGG TGGCTTCAGA CCCGAAGGTG CCGGGAGACC TTTCTTTGGT CACGGAGGAA TGCATGCTGA TGGAGAAGGA GAAATGGAGG TTCCTCAACC 875**

**He185/333_gDNA_0035 AGCAGATGGA TGGACGTGGA CCGAATGGTG GGCCAATGGG CGGTAGGAGG TTTGATGGAC CAGGATTCGG TGGCTTCAGA CCCGAAGGTG CAGGGAGACC TTTCTTCGGT CAAGGAGGAA TGCATGCTGA TGGAGAAGGA GAAATGGAGG TTGCTCAACC 905**

**He185/333_gDNA_0036 AAAAGATGGC TGGACGTGAA CCAAATGGCC GGCCAGTGGG CAGTAGAAAA TTTGATGGAC CAGGATTCGG TGGCTTCAGA CCCGAAGGTG CCGGGAGACC TTTCTTTGGT CATGGAGGAA GGCATGCTGA TGGAGAAGGA GAAATGGAGG CTGCTCAACC 918**

**He185/333_gDNA_0037 AGCAGATGGA TGGACGTGGA CCGAATGGTG GGCCAATGGG CGGTAGGAGG TTTGATGGAC CAGGATTCGG TGGCTTCAGA CCCGAAGGTG CAGGGAGACC TTTCTTCGGT CAAGGAGGAA TGCATGCTGA TGGAGAAGGA GAAATGGAGG TTGCTCAACC 1805**

**He185/333_gDNA_0038 AGCAGATGGA TGGACGTGGA CCGAATGGTG GGCCAATGGG CGGTAGGAGG TTTGATGGAC CAGGATTCGG TGGCTTCAGA CCCGAAGGTG CAGGGAGACC TTTCTTCGGT CAAGGAGGAA TGCATGCTGA TGGAGAAGGA GAAATGGAGG TTGCTCAACC 1805**

**He185/333_gDNA_0039 AACAGATGGC TGGACGTGGA CCGAATGGCC GGCCAGTGGG CAGTAGAAAA TTTGATGGAC CAGGATTCGG TGGCTTCAGA CCCGAAGGTG CCGGGAGACC TTTCTTTGGT CACGGAGGAA TGCATGCTGA TGGAGAAGGA GAAATGGAGG TTCCTCAACC 875**

16

15

14

17

**....|....| ....|....| ....|....| ....|....| ....|....| ....|....| ....|....| ....|....| ....|....| ....|....| ....|....| ....|....| ....|....| ....|....| ....|....| ....|....|**

13

12

**1925 1935 1945 1955 1965 1975 1985 1995 2005 2015 2025 2035 2045 2055 2065 2075**

**He185/333_gDNA_0001 AATCGGTGAT GGTCAAGGAT GGCCCGGTCG TTTCGATGGT CCTGGAAGAT TTTCCGGACG TCCTTACCCA GGCCGTGGCG GTCATCATGG ACACCACCAT GGTCCTCACC ATGACCAGGC CGACGATCAA TCATTTGGTC AGCAAAACGA CAGCAGCAGC 1031**

**He185/333_gDNA_0002 AATCGGTGAT GGTCAAGGAT GGCCCGGTCG TTTCGATGGT CCTGGAAGAT TTTCCGGACG TCCTTACCCA GGCCGTGGCG GTCATCATGG ACACCACCAT GGTCCTCACC ATGACCAGGC CGACGAACAA TCATTTGGTC AGCAAAACGA CAGCAGCAGC 1038**

**He185/333_gDNA_0003 AATCGGTGAT GGTCAAGGAT GGCCCGGTCG TTTCGATGGT CCTGGAAGAT TTTCCGGACG TCCTTACCCA GGCCGTGGCG GTCATCATGG ACACCACCAT GGTCCTCACC ATGACCAGGC CGACGAACAA TCATTTGGTC AGCAAAACGA CAGCAGCAGC 1038**

**He185/333_gDNA_0004 AATCGGTGAT GGTCAAGGAT GGCCCGGTCG TTTCGATGGT CCTGGAAGAT TTTCCGGACG TCCTTACCCA GGCCGTGACG GT-------- ---------- ---------C ATGACAAGGC TGACGAACAA TCATTTGGTC AGCAAAACGA CAGCAGCAGC 1003**

**He185/333_gDNA_0005 AATCGGTGAT GGTCAAGGAT GGCCCGGTCG TTTCGATGGT CCTGGAAGAT TTTCCGGACG TCCTTACCCA GGCCGTGGCG GTCATCATGG ACACCACCAT GGTCCTCACC ATGACCAGGC CGACGAACAA TCATTTGGTC AGCAAAACGA CAGCAGCAGC 1031**

**He185/333_gDNA_0006 AATCGGTGAT GGTCAAGGAT GGCCCGGTCG TTTCGATGGT CCTGGAAGAT TTTCCGGACG TCCTTACCCA GGCCGTGGCG GTCATCATGG ACACCACCAT GGTCCTCACC ATGACCAGGC CGACGAACAA TCATTTGGTC AGCAAAACGA CAGCAGCAGC 1031**

**He185/333_gDNA_0007 AATCGGTGAT GGTCAAGGAT GGCCCGGTCG TTTCGATGGT CCTGGAAGAT TTTCCGGACG TCCTTACCCA GGCCGTGACG GT-------- ---------- ---------C ATGACAAGGC TGACGAACAA TCATTTGGTC AGCAAAACGA CAGCAGCAGC 898**

**He185/333_gDNA_0008 AATCGGTGAT GGTCAAGGAT GGCCCGGTCG TTTCGATGGT CCTGGAAGAT TTTCCGGACG TCCTTACCCA GGCCGTGGCG GTCATCACGG ACACCACCAT GGTCCTCACC AGGACCAGGC CGACGAACAA TCATTTGGTC AGCAAAACGA CAGCAGCAGC 1030**

**He185/333_gDNA_0009 AATCGGTGAT GGTCAAGGAT GGCCCGATCG TTTCGATGGT CCTCGAAGAT TTTCCGGACG TCCTTACCCA GGCCGTGGCG GT-------- ---------- ---------C ATGACAAGGC TGACGAACAA TCATTTGGTC AGCAAAACGA CAGCAGCAGC 1008**

**He185/333_gDNA_0010 AATCGGTGAT GGTCAAGGAT GGCCCGGTCG TTTCGATGGT CCTGGAAGAT TTTCCGGACG TCCTTACCCA GGCCGTGGCG GTCATCATGG ACACCACCAT GGTCCTCACC ATGACCAGGC CGACGAACAA TCATTTGGTC AGCAAAACGA CAGCAGCAGC 1031**

**He185/333_gDNA_0011 AATCGGTGAT GGTCAAGGAT GGCCCGGTCG TTTCGATGGT CCTGGAAGAT TTTCCGGACG TCCTTACCCA GGCCGTGGCG GTCATCATGG ACACCACCAT GGTCCTCACC ATGACCAGGC CGACGAACAA TCATTTGGTC AGCAAAACGA CAGCAGCAGC 1031**

**He185/333_gDNA_0012 AATCGGTGAT GGTCAAGGAT GGCCCGGTCG TTTCGATGGT CCTGGAAGAT TTTCCGGACG TCCTTACCCA GGCCGTGGCG GTCATCATGG ACACCACCAT GGTCCTCACC ATGACCAGGC CGACGAACAA TCATTTGGTC AGCAAAACGA CAGCAGCAGC 1031**

**He185/333_gDNA_0013 AATCGGTGAT GGTCAAGGAT GGCCCGGTCG TTTCGATGGT CCTGGAAGAT TTTCCGGACG TCCTTACCCA GGCCGTGGCG GTCATCATGG ACACCACCAT GGTCCTCACC ATGACCAGGC CGACGAACAA TCATTTGGTC AGCAAAACGA CAGCAGCAGC 1030**

**He185/333_gDNA_0014 AATCGGTGAT GGTCAAGGAT GGCCCGGTCG TTTCGATGGT CCTGGAAGAT TTTCCAGACG TCCTTACCCA GGACGTGGTG GTCATCATGG ACACCACCAT GGTCCTCGCC ATGACCAGGC AGACGAACAA TCAACTGGTC AGCAAAACGA CAGCAGCAGC 1104**

**He185/333_gDNA_0015 AATCGGTGAT GGTCAAGGAT GGCCCGGTCG TTTCGATGGT CCTGGAAGAT TTTCCGGACG TCCTTACCCA GGCCGTGACG GT-------- ---------- ---------C ATGACAAGGC TGACGAACAA TCATTTGGTC AGCAAAACGA CAGCAGCAGC 898**

**He185/333_gDNA_0016 AATCGGTGAT GGTCAAGGAT GGCCCGGTCG TTTCGATGGT CCTGGAAGAT TTTCCAGACG TCCTTACCCA GGACGTGGTG GTCATCATGG ACACCACCAT GGTCCTCGCC ATGACCAGGC AGACGAACAA TCAACTGGTC AGCAAAACGA CAGCAGCAGC 1102**

**He185/333_gDNA_0017 AATCGGTGAT GGTCAAGGAT GGCCCGGTCG TTTCGATGGT CCTGGAAGAT TTTCCAGACG TCCTTACTCA GGACGTGGTG GTCATCATGG ACACCACCAT GGTCCTCGCC ATGACCAGGC AGACGAACAA TCAACTGGTC AGCAAAACGA CAGCAGCAGC 1105**

**He185/333_gDNA_0018 AATCGGTGAT GGTCAAGGAT GGCCCGGTCG TTTCGATGGT CCTGGAAGAT TTTCCAGACG TCCTTACACA GGACGTGGTG GTCATCATGG ACACCACCAT GGTCCTCGCC ATGACCAGGC AGACGAACAA TCAACTGGTC AGCAAAACGA CAGCAGCAGC 1105**

**He185/333_gDNA_0019 AATCGGTGAT GGTCAAGGAT GGCCCGATCG TTTCGATGGT CCTCGAAGAT TTTCCGGACG TCCTTACCCA GGCCGTGGCG GT-------- ---------- ---------C ATGACAAGGC TGACGAACAA TCATTTGGTC AGCAAAACGA CAGCAGCAGC 1008**

**He185/333_gDNA_0020 AATCGGTGAT GGTCAAGGAT GGCCCGATCG TTTCGATGGT CCTCGAAGAT TTTCCGGACG TCCTTACCCA GGCCGTGGCG GT-------- ---------- ---------C ATGACAAGGC TGACGAACAA TCATTTGGTC AGCAAAACGA CAGCAGCAGC 1008**

**He185/333_gDNA_0021 AATCGGTGAT GGTCAAGGAT GGCCCGGTCG TTTCGATGGT CCTGGAAGAT TTTCCAGACG TCCTTACCCA GGACGTGGTG GTCATCATGG ACACCACCAT GGTCCTCGCC ATGACCAGGC AGACGAACAA TCAACTGGTC AGCAAAACGA CAGCAGCAGC 1105**

**He185/333_gDNA_0022 AATCGGTGAT GGTCAAGGAT GGCCCGATCG CTTCGATGGT CCTCGAAGAT TTTCCGGACG TCCTTACCCA GGCCGTGGCG GT-------- ---------- ---------C ATGACAAGGC TGACGAACAA TCATTTGGTC AGCAAAACGA CAGCAGCAGC 1008**

**He185/333_gDNA_0023 AATCGGTGAT GGTCAAGGAT GGCCCGGTCG TTTCGATGGT CCTGGAAGAT TTTCCAGACG TCCTTACCCA GGACGTGGTG GTCATCATGG ACACCACCAT GGTCCTCGCC ATGACCAGGC AGACGAACAA TCAACTGGTC AGCAAAACGA CAGCAGCAGC 1105**

**He185/333_gDNA_0024 AATCGGTGAT GGTCAAGGAT GGCCCGGTCG TTTCGATGGT CCTGGAAGAT TTTCCAGACG TCCTTACCCA GGACGTGGTG GTCATCATGG ACACCACCAT GGTCCTCGCC ATGACCAGGC AGACGAACAA TCAACTGGTC AGCAAAACGA CAGCAGCAGC 1878**

**He185/333_gDNA_0025 AATCGGTGAT GGTCAAGGAT GGCCCGGTCG TTTCGATGGT CCTGGAAGAT ATTCCAGACG TCCTTACCCA GGACGTGGTG GTCATCATGG ACACCACCAT GGTCCTCGCC ATGACCAGGC AGACGAACAA TCAACTGGTC AGCAAAACGA CAGCAGCAGC 1105**

**He185/333_gDNA_0026 AATCGGTGAT GGTCAAGGAT GGCCCGATCG TTTCGATGGT CCTCGAAGAT TTTCCGGACG TCCTTACCCA GGCCGTGGCG GT-------- ---------- ---------C ATGACAAGGC TGACGAACAA TCATTTGGTC AGCAAAACGA CAGCAGCAGC 1008**

**He185/333_gDNA_0027 AATCGGTGAT GGTCAAGGAT GGCCCGGTCG TTTCGATGGT CCTGGAAGAT TTTCCGGACG TCCTTACCCA GGCCGTGGCG GTCATCATGG ACACCACCAT GGTCCTCACC ATGACAAGGC CGACGAACAA TCATTTGGTC AGCAAAACGA CAGCAGCAGC 1075**

**He185/333_gDNA_0028 AATCGGTGAT GGTCAAGGAT GGCCCGGTCG TTTCGATGGT CCTGGAAGAT TTTCCAGACG TCCTTACCCA GGACGTGGTG GTCATCATGG ACACCACCAT GGTCCTCGCC ATGACCAGGC AGACGAACAA TCAACTGGTC AGCAAAACGA CAGCAGCAGC 1105**

**He185/333_gDNA_0029 AATCGGTGAT GGTCAAGGAT GGCCCGGTCG TTTCGATGGT CCTGGAAGAT TTTCCAGACG TCCTTACCCA GGACGTGGTG GTCATCATGG ACACCACCAT GGTCCTCGCC ATGACCAGGC AGACGAACAA TCAACTGGTC AGCAAAACGA CAGCAGCAGC 1105**

**He185/333_gDNA_0030 AATCGGTGAT GGTCAAGGAT GGCCCGATCG TTTCGATGGT CCTCGAAGAT TTTCCGGACG TCCTTACCCA GGCCGTGGCG GT-------- ---------- ---------C ATGACAAGGC TGACGAACAA TCATTTGGTC AGCAAAACGA CAGCAGCAGC 1008**

**He185/333_gDNA_0031 AATCGGTGAT GGTCAAGGAT GGCCCGGTCG TTTCGATGGT CCTGGAAGAT TTTCCGGACG TCCTTACCCA GGCCGTGGCG GTCATCATGG ACACCACCAT GGTCCTCACC ATGACAAGGC CGACGAACAA TCATTTGGTC AGCAAAACGA CAGCAGCAGC 1075**

**He185/333_gDNA_0032 AATCGGTGAT GGTCAAGGAT GGCCCGGTCG TTTCGATGGT CCTGGAAGAT TTTCCAGACG TCCTTACCCA GGACGTGGTG GTCATCATGG ACACCACCAT GGTCCTCGCC ATGACCAGGC AGACGAACAA TCAACTGGTC AGCAAAACGA CAGCAGCAGC 1104**

**He185/333_gDNA_0033 AATCGGTGAT GGTCAAGGAT GGCCCGATCG TTTCGATGGT CCTCGAAGAT TTTCCGGACG TCCTTACCCA GGCCGTGGCG GT-------- ---------- ---------C ATGACAAGGC TGACGAACAA TCATTTGGTC AGCAAAACGA CAGCAGCAGC 1008**

**He185/333_gDNA_0034 AATCGGTGAT GGTCAAGGAT GGCCCGATCG TTTCGATGGT CCTCGAAGAT TTTCCGGACG TCCTTACCCA GGCCGTGGCG GT-------- ---------- ---------C ATGACAAGGC TGACGAACAA TCATTTGGTC AGCAAAACGA CAGCAGCAGC 1008**

**He185/333_gDNA_0035 AATCGGTGAT GGTCAAGGAT GGCCCGGTCG TTTCGATGGT CCTGGAAGAT TTTCCGGACG TCCTTACCCA GGCCGTGACG GT-------- ---------- ---------C ATGACAAGGC TGACGAACAA TCATTTGGTC AGCAAAACGA CAGCAGCAGC 1038**

**He185/333_gDNA_0036 AATCGGTGAT GGTCAAGGAT GGCCCGGTCG TTTCGATGGT CCTGGAAGAT TTTCCAGACG TCCTTACCCA GGACGTGGTG GTCATCATGG ACACCACCAT GGTCCTCGCC ATGACCAGGC AGACGAACAA TCAACTGGTC AGCAAAACGA CAGCAGCAGC 1078**

**He185/333_gDNA_0037 AATCGGTGAT GGTCAAGGAT GGCCCGGTCG TTTCGATGGT CCTGGAAGAT TTTCCGGACG TCCTTACCCA GGCCGTGACG GT-------- ---------- ---------C ATGACAAGGC TGACGAACAA TCATTTGGTC AGCAAAACGA CAGCAGCAGC 1938**

**He185/333_gDNA_0038 AATCGGTGAT GGTCAAGGAT GGCCCGGTCG TTTCGATGGT CCTGGAAGAT TTTCCGGACG TCCTTACCCA GGCCGTGACG GT-------- ---------- ---------C ATGACAAGGC TGACGAACAA TCATTTGGTC AGCAAAACGA CAGCAGCAGC 1938**

**He185/333_gDNA_0039 AATCGGTGAT GGTCAAGGAT GGCCCGATCG TTTCGATGGT CCTCGAAGAT TTTCCGGACG TCCTTACCCA GGCCGTGGCG GT-------- ---------- ---------C ATGACAAGGC TGACGAACAA TCATTTGGTC AGCAAAACGA CAGCAGCAGC 1008**

19

17

18

**....|....| ....|....| ....|....| ....|....| ....|....| ....|....| ....|....| ....|....| ....|....| ....|....| ....|....| ....|....| ....|....| ....|....| ....|....| ....|....|**

20

21

**2085 2095 2105 2115 2125 2135 2145 2155 2165 2175 2185 2195 2205 2215 2225 2235**

**He185/333_gDNA_0001 GAGGAGGATG GCCGACCTCA CCGTCACCAC CACCAC---- -----CATCA TCACCATGAC CGTCATAACA AGACAGACGA CCACCATCAC CATAATCACA CCGAAGGCCA CCGCCACCAT ---------- ---------- ---------- ---------- 1142**

**He185/333_gDNA_0002 GAGGAGGATG GCCGACCTCA CCGTCACCAC CACCAC---- -----CATCA TCACCATGAC CGTCATAACA AGACAGACGA CCACCATCAC CATAATCACA CCGAAGGCCA CCGCCACCAT ---------- ---------- ---------- ---------- 1141**

**He185/333_gDNA_0003 GAGGAGGATG GCCGACCTCA CCGTCACCAC CACCAC---- -----CATCA TCACCATGAC CGTCATAACA AGACAGACGA CCACCATCAC CATAATCACA CCGAAGGCCA CCGCCACCAT ---------- ---------- ---------- ---------- 1141**

**He185/333_gDNA_0004 GAGGAGGATG GCCGACCTCA CCGTCACCAC CACCAC---- -----CATCA TCACCATGAC CGTCATAACA AGACAGACGA CCACCATCAC CATAATCACA CCGAAGGCCA CCGCCACCAT ---------- ---------- ---------- ---------- 1114**

**He185/333_gDNA_0005 GAGGAGGATG GCCGACCTCA CCGTCACCAC CACCAC---- -----CATCA TCACCATGAC CGTCATAACA AGACAGACGA CCACCATCAC CATAATCACA CCGAAGGCCA CCGCCACCAT ---------- ---------- ---------- ---------- 1142**

**He185/333_gDNA_0006 GAGGAGGATG GCCGACCTCA CCGTCACCAC CACCAC---- -----CATCA TCACCATGAC CGTCATAACA AGACAGACGA -CACCATCAC CATAATCACA CCGAAGGCCA CCGCCACCAT ---------- ---------- ---------- ---------- 1141**

**He185/333_gDNA_0007 GAGGAGGATG GCCGACCTCA CCGTCACCAC CACCAC---- -----CATCA TCACCATGAC CGTCATAACA AGACAGACGA CCACCATCAC CATAATCACA CCGAAGGCCA CCGCCACCAT ---------- ---------- ---------- ---------- 1009**

**He185/333_gDNA_0008 GAGGAGGATG GCCGACCTCA CCGTCACCAC CACCAC---- -----CATCA TCACCATGAC CGTCATAACA AGACAGACGA CCACCATCAC CATAATCACA CCGAAGGCCA CCGCCACCAT ---------- ---------- ---------- ---------- 1141**

**He185/333_gDNA_0009 GAGGAGGATG GCCGACCTCA CCATCACCAC CACCAC---- -----CATCA TCACCATGAC CGTCATAACA AGACAGACGA CCACCATCAC CATAATCACA CCGAAGGCCA CCGCCACCAG ---------- ---------- ---------- ---------- 1119**

**He185/333_gDNA_0010 GAGGAGGATG GCCGACCTCA CCGTCACCAC CACCAC---- -----CATCA TCACCATGAC CGTCATAACA AGACAGACGA CCACCATCAC CATAATCACA CCGAAGGCCA CCGCCACCAT ---------- ---------- ---------- ---------- 1142**

**He185/333_gDNA_0011 GAGGAGGATG GCCGACCTCA CCGTCACCAC CACCAC---- -----CATCA TCACCATGAC CGTCATAACA AGACAGACGA CCACCATCAC CATAATCACA CCGAAGGCCA CCGCCACCAT ---------- ---------- ---------- ---------- 1142**

**He185/333_gDNA_0012 GAGGAGGATG GCCGACCTCA CCGTCACCAC CACCAC---- -----CATCA TCACCATGAC CGTCATAACA AGACAGACGA CCACCGTCAT CATAATCACA TCGAAGGCCA CCGCCACCAT ---------- ---------- ---------- ---------- 1142**

**He185/333_gDNA_0013 GAGGAGGATG GCCGACCTCA CCGTCACCAC CACCAC---- -----CATCA TCACCATGAC CGTCATAACA AGACAGACGA CCACCATCAC CATAATCACA CCGAAGGCCA CCGCCACCAT ---------- ---------- ---------- ---------- 1141**

**He185/333_gDNA_0014 GAGGAGGATG GCCGACCTCA CCGTCACCAC CACCAT---- -----CATCA TCACCATGAC CGTCATAACA AGACAGACGA CCACCATCAC CATAATCACA CCGAAGGCCA CCGCCACCAT ---------- ---------- ---------- ---------- 1215**

**He185/333_gDNA_0015 GAGGAGGATG GCCGACCTCA CCGTCACCAC CACCAC---- -----CATCA TCACCATGAC CGTCATAACA AGACAGACGA CCACCATCAC CATAATCACA CCGAAGGCCA CCGCCACCAT ---------- ---------- ---------- ---------- 1009**

**He185/333_gDNA_0016 GAGGAGGATG GCCGACCTCA CCGTCACCAC CACCAT---- -----CATCA TCACCATGAC CGTCATAACA AGACAGACGA CCACCATCAC CATAATCACA CCGAAGGCCA CCGCCACCAT ---------- ---------- ---------- ---------- 1713**

**He185/333_gDNA_0017 GAGGAGGATG GCCGACCTCA CCGTCACCAC CACCAT---- -----CATCA TCACCATGAC CGTCATAACA AGACAGACGA CCACCATCAC CATAATCACA CCGAAGGCCA CCGCCACCAT ---------- ---------- ---------- ---------- 1216**

**He185/333_gDNA_0018 GAGGAGGATG GCCGACCTCA CCGTCACCAC CACCAT---- -----CATCA TCACCATGAC CGTCATAACA AGACAGACGA CCACCATCAC CATAATCACA CCGAAGGCCA CCGCCACCAT ---------- ---------- ---------- ---------- 1216**

**He185/333_gDNA_0019 GAGGAGGATG GCCGACCTCA CCATCACCAC CACCAC---- -----CATCA TCACCATGAC CGTCATAACA AGACAGACGA CCACCATCAC CATAATCACA CCGAAGGCCA CCGCCACCAG ---------- ---------- ---------- ---------- 1119**

**He185/333_gDNA_0020 GAGGAGGATG GCCGACCTCA CCATCACCAC CACCAC---- -----CATCA TCACCATGAC CGTCATAACA AGACAGACGA CCACCATCAC CATAATCACA CCGAAGGCCA CCGCCACCAT ---------- ---------- ---------- ---------- 1119**

**He185/333_gDNA_0021 GAGGAGGATG GCCGACCTCA CCGTCACCAC CACCAT---- -----CATCA TCACCATGAC CGTCATAACA AGACAGACGA CCACCATCAC CATAATCACA CCGAAGGCCA CCGCCACCAT ---------- ---------- ---------- ---------- 1216**

**He185/333_gDNA_0022 GAGGAGGATG GCCGACCTCA CCATCACCAC CACCAC---- -----CATCA TCACCATGAC CGTCATAACA AGACAGACGA CCACCATCAC CATAATCACA CCGAAGGCCA CCGCCACCAG ---------- ---------- ---------- ---------- 1119**

**He185/333_gDNA_0023 GAGGAGGATG GCCGACCTCA CCGTCACCAC CACCAT---- -----CATCA TCACCATGAC CGTCATAACA AGACAGACGA CCACCATCAC CATAATCACA CCGAAGGCCA CCGCCACCAT ---------- ---------- ---------- ---------- 1216**

**He185/333_gDNA_0024 GAGGAGGATG GCCGACCTCA CCGTCACCAC CACCAT---- -----CATCA TCACCATGAC CGTCATAACA AGACAGACGA CCACCATCAC CATAATCACA CCGAAGGCCA CCGCCACCAT ---------- ---------- ---------- ---------- 1189**

**He185/333_gDNA_0025 GAGGAGGATG GCCGACCTCA CCGTCACCAC CACCAT---- -----CATCA TCACCATGAC CGTCATAACA AGACAGACGA CCACCATCAC CATAATCACA CCGAAGGCCA CCGCCACCAT ---------- ---------- ---------- ---------- 1216**

**He185/333_gDNA_0026 GAGGAGGATG GCCGACCTCA CCATCACCAC CACCAC---- -----CATCA TCACCATGAC CGTCATAACA AGACAGACGA CCACCATCAC CATAATCACA CCGAAGGCCA CCGCCACCAG ---------- ---------- ---------- ---------- 1119**

**He185/333_gDNA_0027 GAGGAGGATG GCCGACCTCA CCATCATCAC CACCACCACC ACCACCATCA TCACCATGAC CGTCATAACA AGACAGACGA CCACCATCAT CATAATCACA CCGAAGGCCA CCGCCACCAT ---------- ---------- ---------- ---------- 1195**

**He185/333_gDNA_0028 GAGGAGGATG GCCGACCTCA CCGTCACCAC CACCAT---- -----CATCA TCACCATGAC CGTCATAACA AGACAGACGA CCACCATCAC CATAATCACA CCGAAGGCCA CCGCCACCAT ---------- ---------- ---------- ---------- 1216**

**He185/333_gDNA_0029 GAGGAGGATG GCCGACCTCA CCGTCACCAC CACCAT---- -----CATCA TCACCATGAC CGTCATAACA AGACAGACGA CCACCATCAC CATAATCACA CCGAAGGCCA CCGCCACCAT ---------- ---------- ---------- ---------- 1216**

**He185/333_gDNA_0030 GAGGAGGATG GCCGACCTCA CCATCACCAC CACCAC---- -----CATCA TCACCATGAC CGTCATAACA AGACAGACGA CCACCATCAC CATAATCACA CCGAAGGCCA CCGCCACCAG ---------- ---------- ---------- ---------- 1119**

**He185/333_gDNA_0031 GAGGAGGATG GCCGACCTCA CCATCATCAC CACCACCACC ACCACCATCA TCACCATGAC CGTCATAACA AGACAGACGA CCACCATCAT CATAATCACA CCGAAGGCCA CCGCCACCAT ---------- ---------- ---------- ---------- 1195**

**He185/333_gDNA_0032 GAGGAGGATG GCCGACCTCA CCGTCACCAC CACCAT---- -----CATCA TCACCATGAC CGTCATAACA AGACAGACGA CCACCATCAC CATAATCACA CCGAAGGCCA CCGCCACCAT ---------- ---------- ---------- ---------- 1215**

**He185/333_gDNA_0033 GAGGAGGATG GCCGACCTCA CCATCACCAC CACCAC---- -----CATCA TCACCATGAC CGTCATAACA AGACAGACGA CCACCATCAC CATAATCACA CCGAAGGCCA CCGCCACCAG ---------- ---------- ---------- ---------- 1119**

**He185/333_gDNA_0034 GAGGAGGATG GCCGACCTCA CCATCACCAC CACCAC---- -----CATCA TCACCATGAC CGTCATAACA AGACAGACGA CCACCATCAC CATAATCACA CCGAAGGCCA CCGCCACCAG ---------- ---------- ---------- ---------- 1119**

**He185/333_gDNA_0035 GAGGAGGATG GCCGACCTCA CCGTCACCAC CACCAC---- -----CATCA TCACCATGAC CGTCATAACA AGACAAACGA CCACCATCAC CATAATCACA CCGAAGGCCA CCGCCACCAT ---------- ---------- ---------- ---------- 1149**

**He185/333_gDNA_0036 GAGGAGGATG GCCGACCTCA CCGTCACCAC CACCAT---- -----CATCA TCACCATGAC CGTCATAACA AGACAGACGA CCACCATCAC CATAATCACA CCGAAGGCCA CCGCCACCAT ---------- ---------- ---------- ---------- 1189**

**He185/333_gDNA_0037 GAGGAGGATG GCCGACCTCA CCGTCACCAC CACCAC---- -----CATCA TCACCATGAC CGTCATAACA AGACAAACGA CCACCATCAC CATAATCACA CCGAAGGCCA CCGCCACCAT ---------- ---------- ---------- ---------- 2049**

**He185/333_gDNA_0038 GAGGAGGATG GCCGACCTCA CCGTCACCAC CACCAC---- -----CATCA TCACCATGAC CGTCATAACA AGACAAACGA CCACCATCAC CATAATCACA CCGAAGGCCA CCGCCACCAT ---------- ---------- ---------- ---------- 2049**

**He185/333_gDNA_0039 GAGGAGGATG GCCGACCTCA CCATCACCAC CACCAC---- -----CATCA TCACCATGAC CGTCATAACA AGACAGACGA CCACCATCAC CATAATCACA CCGAAGGCCA CCGCCACCAG ---------- ---------- ---------- ---------- 1119**

**....|....| ....|....| ....|....| ....|....| ....|....| ....|....| ....|....| ....|....| ....|....| ....|....| ....|....| ....|....| ....|....| ....|....| ....|....| ....|....|**

22

23

21

**2245 2255 2265 2275 2285 2295 2305 2315 2325 2335 2345 2355 2365 2375 2385 2395**

**He185/333_gDNA_0001 ---------- ---------- ---------- ---------- ---------- ---------- ---------- -CATCATAAC AAGACAGAAG AGGGTGACCA GGACAGACCA GAGATGAGGC CATTCCGGTT CAACCCTTTC GGTCGCAAAC CTTTCGGAAG 1231**

**He185/333_gDNA_0002 ---------- ---------- ---------- ---------- ---------- ---------- ---------- -CATCATAAC AAGACAGAAG AGGGTGACCA GGACAGACCA GAGATGAGGC CATTCCGGTT CAACCCTTTC GGTCGCAAAC CTTTCGGAGG 1230**

**He185/333_gDNA_0003 ---------- ---------- ---------- ---------- ---------- ---------- ---------- -CATCATAAC AAGACAGAAG AGGGTGACCA GGACAGACCA GAGATGAGGC CATTCCGGTT CAACCCTTTC GGTCGCAAAC CTTTCGGAGG 1230**

**He185/333_gDNA_0004 ---------- ---------- ---------- ---------- ---------- ---------- ---------- -CATCATAAC AAGACAGAAG AGGGTGACCA GGACAGACCA GAGATGAGGC CATTCCGGTT CAACCCTTTC GGTCGCAAGC CTTTCGGAGG 1203**

**He185/333_gDNA_0005 ---------- ---------- ---------- ---------- ---------- ---------- ---------- -CATCATAAC AAGACAGAAG AGGGTGACCA GGACAGACCA GAGATGAGGC CATTCCGGTT CAACCCTTTC GGTCGCAAAC CTTTCGGAAG 1231**

**He185/333_gDNA_0006 ---------- ---------- ---------- ---------- ---------- ---------- ---------- -CATCATAAC AAGACAGAAG AGGGTGACCA GGACAGACCA GAGATGAGGC CATTCCGGTT CAACCCTTTC GGTCGCAAAC CTTTCGGAAG 1230**

**He185/333_gDNA_0007 ---------- ---------- ---------- ---------- ---------- ---------- ---------- -CATCATAAC AAGACAGAAG AGGGTGACCA GGACAGACCA GAGATGAGGC CATTCCGGTT CAACCCTTTC GGTCGCAAGC CTTTCGGAGG 1098**

**He185/333_gDNA_0008 ---------- ---------- ---------- ---------- ---------- ---------- ---------- -CATCATAAC AAGACAGAAG AGGGTGACCA GGACAGACCA GAGATGAGGC CATTCCGGTT CAACCCTTTC GGTCGCAAAC CTTTCGGAGG 1230**

**He185/333_gDNA_0009 ---------- ---------- ---------- ---------- ---------- ---------- ---------- -CATCATAAC AAGACAGAAG AGGGTGACCA GGACAGACCA GAGATGAGGC CATTCCGGTT CAACGCTTTC GGTCGCAAGC CTTTCGGAGG 1208**

**He185/333_gDNA_0010 ---------- ---------- ---------- ---------- ---------- ---------- ---------- -CATCATAAC AAGACAGAAG AGGGTGACCA GGACAGACCA GAGATGAGGC CATTCCGGTT CAACCCTTTC GGTCGCAAAC CTTTCGGAAG 1231**

**He185/333_gDNA_0011 ---------- ---------- ---------- ---------- ---------- ---------- ---------- -CATCATAAC AAGACAGAAG AGGGTGACCA GGACAGACCA GAGATGAGGC CATTCCGGTT CAACCCTTTC GGTCGCAAAC CTTTCGGAAG 1231**

**He185/333_gDNA_0012 ---------- ---------- ---------- ---------- ---------- ---------- ---------- -CATCATAAC AAGACAGAAG AGGGTGACCA GGACAGACCA GAGATGAGGC CATTCCGGTT CAACCCTTTC GGTCGCAAGC CTTTCGGAGG 1231**

**He185/333_gDNA_0013 ---------- ---------- ---------- ---------- ---------- ---------- ---------- -CATCATAAC AAGACAGAAG AGGGTGACCA GGACAGACCA GAGATGAGGC CATTCCGGTT CAACCCTTTC GGTCGCAAAC CTTTCGGAAG 1230**

**He185/333_gDNA_0014 ---------- ---------- ---------- ---------- ---------- ---------- ---------- -CATCATAAC AAGACAGAAG AGGGTGACCA GGACAGACCA GAGATGAGGC CATTCCGGTT CAACCCTTTC GGTCGCAAGC CTTTCGGAGG 1304**

**He185/333_gDNA_0015 ---------- ---------- ---------- ---------- ---------- ---------- ---------- -CATCATAAC AAGACAGAAG AGGGTGACCA GGACAGACCA GAGATGAGGC CATTCCGGTT CAACCCTTTC GGTCGCAAGC CTTTTGGAGG 1098**

**He185/333_gDNA_0016 ---------- ---------- ---------- ---------- ---------- ---------- ---------- -CATCATAAC AAGACAGAAG AGGGTGACCA GGACAGACCA GAGATGAGGC CATTCCGGTT CAACCCTTTC GGTCGCAAGC CTTTCGGAGG 1302**

**He185/333_gDNA_0017 ---------- ---------- ---------- ---------- ---------- ---------- ---------- -CATCATAAC AAGACAGAAG AGGGTGACCA GGACAGACCA GAGATGAGGC CATTCCGGTT CAACCCTTTC GGTCGCAAGC CTTTCGGAGG 1305**

**He185/333_gDNA_0018 ---------- ---------- ---------- ---------- ---------- ---------- ---------- -CATCATAAC AAGACAGAAG AGGGTGACCA GGACAGACCA GAGATGAGGC CATTCCGGTT CAACCCTTTC GGTCGCAAGC CTTTCGGAGG 1305**

**He185/333_gDNA_0019 ---------- ---------- ---------- ---------- ---------- ---------- ---------- -CATCATAAC AAGACAGAAG AGGGTGACCA GGACAGACCA GAGATGAGGC CATTCCGGTT CAACCCTTTC GGTCGCAAGC CTTTCGGAGG 1208**

**He185/333_gDNA_0020 ---------- ---------- ---------- ---------- ---------- ---------- ---------- -CATCATAAC AAGACAGAAG AGGGTGACCA GGACAGACCA GAGATGAGGC CATTCCGGTT CAACCCTTTC GGTCGCAAGC CTTTCGGAGG 1208**

**He185/333_gDNA_0021 ---------- ---------- ---------- ---------- ---------- ---------- ---------- -CATCATAAC AAGACAGAAG AGGGTGACCA GGACAGACCA GAGATGAGGC CATTCCGGTT CAACCCTTTC GGTCGCAAGC CTTTCGGAGG 1305**

**He185/333_gDNA_0022 ---------- ---------- ---------- ---------- ---------- ---------- ---------- -CATCATAAC AAGACAGAAG AGGGTGACCA GGACAGACCA GAGATGAGGC CATTCCGGTT CAACCCTTTC GGTCGCAAGC CTTTCGGAGG 1208**

**He185/333_gDNA_0023 ---------- ---------- ---------- ---------- ---------- ---------- ---------- -CATCATAAC AAGACAGAAG AGGGTGACCA GGACAGACCA GAGATGAGGC CATTCCGGTT CAACCCTTTC GGTCGCAAGC CTTTCGGAGG 1305**

**He185/333_gDNA_0024 ---------- ---------- ---------- ---------- ---------- ---------- ---------- -CATCATAAC AAGACAGAAG AGGGTGACCA GGACAGACCA GAGATGAGGC CATTCCGGTT CAACCCTTTC GGTCGCAAGC CTTTCGGAGG 1278**

**He185/333_gDNA_0025 ---------- ---------- ---------- ---------- ---------- ---------- ---------- -CATCATAAC AAGACAGAAG AGGGTGACCA GGACAGACCA GAGATGAGGC CATTCCGGTT CAACCCTTTC GGTCGCAAGC CTTTCGGAGG 1305**

**He185/333_gDNA_0026 ---------- ---------- ---------- ---------- ---------- ---------- ---------- -CATCATAAC AAGACAGAAG AGGGTGACCA GGACAGACCA GAGATGAGGC CATTCCGGTT CAACCCTTTC GGTCGCAAGC CTTTCGGAGG 1208**

**He185/333_gDNA_0027 ---------- ---------- ---------- ---------- ---------- ---------- ---------- -CATCATAAC AAGACAGAAG AGGGTGACCA GGACAGACCA GAGATGAGGC CATTCCGGTT CAACCCTTTC GGTCGCAAGC CTTTCGGAGG 1284**

**He185/333_gDNA_0028 ---------- ---------- ---------- ---------- ---------- ---------- ---------- -CATCATAAC AAGACAGAAG AGGGTGACCA GGACAGACCA GAGATGAGGC CATTCCGGTT CAACCCTTTC GGTCGCAAGC CTTTCGGAGG 1305**

**He185/333_gDNA_0029 ---------- ---------- ---------- ---------- ---------- ---------- ---------- -CATCATAAC AAGACAGAAG AGGGTGACCA GGACAGACCA GAGATGAGGC CATTCCGGTT CAACCCTTTC GGTCGCAAGC CTTTCGGAGG 1305**

**He185/333_gDNA_0030 ---------- ---------- ---------- ---------- ---------- ---------- ---------- -CATCATAAC AAGACAGAAG AGGGTGACCA GGACAGACTA GAGATGAGGC CATTCCGGTT CAACCCTTTC GGTCGCAAGC CTTTCGGAGG 1208**

**He185/333_gDNA_0031 ---------- ---------- ---------- ---------- ---------- ---------- ---------- -CATCATAAC AAGACAGAAG AGGGTGACCA GGACAGACCA GAGATGAGGC CATTCCGGTT CAACCCTTTC GGTCGCAAGC CTTTCGGAGG 1284**

**He185/333_gDNA_0032 ---------- ---------- ---------- ---------- ---------- ---------- ---------- -CATCATAAC AAGACAGAAG AGGGTGACCA GGACAGACCA GAGATGAGGC CATTCCGGTT CAACCCTTTC GGTCGCAAGC CTTTCGGAGG 1304**

**He185/333_gDNA_0033 ---------- ---------- ---------- ---------- ---------- ---------- ---------- -CATCATAAC AAGACAGAAG AGGGTGACCA GGACAGACCA GAGATGAGGC CATTCCGGTT CAACCCTTTC GGTCGCAAGC CTTTCGGAGG 1208**

**He185/333_gDNA_0034 ---------- ---------- ---------- ---------- ---------- ---------- ---------- -CATCATAAC AAGACAGAAG AGGGTGACCA GGACAGACCA GAGATGAGGC CATTCCGGTT CAACCCTTTC GGTCGCAAGC CTTTCGGAGG 1208**

**He185/333_gDNA_0035 ---------- ---------- ---------- ---------- ---------- ---------- ---------- -CATCATAAC AAGACAGAAG AGGGTGACCA GGACAGACCA GAGATGAGGC CATTCCGGTT CAACCCTTTC GGTCGCAAGC CTTTCGGAGG 1238**

**He185/333_gDNA_0036 ---------- ---------- ---------- ---------- ---------- ---------- ---------- -CATCATAAC AAGACAGAAG AGGGTGACCA GGACAGACCA GAGATGAGGC CATTCCGGTT CAACCCTTTC GGTCGCAAGC CTTTCGGAGG 1278**

**He185/333_gDNA_0037 ---------- ---------- ---------- ---------- ---------- ---------- ---------- -CATCATAAC AAGACAGAAG AGGGTGACCA GGACAGACCA GAGATGAGGC CATTCCGGTT CAACCCTTTC GGTCGCAAGC CTTTCGGAGG 2138**

**He185/333_gDNA_0038 ---------- ---------- ---------- ---------- ---------- ---------- ---------- -CATCATAAC AAGACAGAAG AGGGTGACCA GGACAGACCA GAGATGAGGC CATTCCGGTT CAACCCTTTC GGTCGCAAGC CTTTCGGAGG 2138**

**He185/333_gDNA_0039 ---------- ---------- ---------- ---------- ---------- ---------- ---------- -CATCATAAC AAGACAGAAG AGGGTGACCA GGACAGACCA GAGATGAGGC CATTCCGGTT CAACCCTTTC GGTCGCAAGC CTTTCGGAGG 1208**

**....|....| ....|....| ....|....| ....|....| ....|....| ....|....| ....|....| ....|....| ....|....| ....|....| ....|....| ....|....| ....|....| ....|....| ....|....| ....|....|**

24

25

26

27

28

29

23

**2405 2415 2425 2435 2445 2455 2465 2475 2485 2495 2505 2515 2525 2535 2545 2555**

**He185/333_gDNA_0001 ACGTCCATTC GGCAGACGCA ACCATACCGA AGAAGGATCT CCCAGGCGCG ATGGC----- ---------- ---AACCGTG GACGTTGGGA TGAGAATGAA AGT------- --------GT GGAGGAAGAA CATCTTCCGA CTGAAAGCAT GACAACATCT 1358**

**He185/333_gDNA_0002 ACGTCCATTC GGCAGACGCA ACCATACCGA AGAAGGATCT CCCAGGCGCG ATGGC----- ---------- ---AACCGTG GACGTTGGGA TGAGAATGAA AGT------- --------GT GGAGGAAGAA CATCTTCCGA CTGAAAGCAT GACAACATCT 1357**

**He185/333_gDNA_0003 GCGTCCATTC GGCAGACGCA ACCATACCGA AGAAGGATCT CCCAGGCGCG ATGGC----- ---------- ---AACCGTG GACGTTGGGA TGAGAATGAA AGT------- --------GT GGAGGAAGAA CATCTTCCGA CTGAAAGCAT GACAACATCT 1357**

**He185/333_gDNA_0004 ACGTCCATTC GGCAGATGCA ACCATACCGA AGAAGGATCT CCCAGGCGCG ATGGAGATCG TCGTCCCAAG GGCAACCGTG GACGTTGGGA TGAGAATGAA AGT------- --------GA GGAGGAAGAA CATCTTCCAA CTGAAAGCAT GACAACATCT 1348**

**He185/333_gDNA_0005 ACGTCAATTC GGCAGACGCA ACCATACCGA AGAAGGATCT CCCAGGCGCG ATGGC----- ---------- ---AACCGTG GACGTTGGGA TGAGAATGAA AGT------- --------GT GGAGGAAGAA CATCTTCCGA CTGAAAGCAT GACAACATCT 1358**

**He185/333_gDNA_0006 ACGTCAATTC GGCAGACGCA ACCATACCGA AGAAGGATCT CCCAGGCGCG ATGGC----- ---------- ---AACCGTG GACGTTGGGA TGAGAATGAA AGT------- --------GT GGAGGAAGAA CATCTTCCGA CTGAAAGCAT GACAACATCT 1356**

**He185/333_gDNA_0007 ACGTCCATTC GGCAGATGCA ACCATACCGA AGAAGGATCT CCCAGGCGCG ATGGAGATCG TCGTCCCAAG GGCAACCGTG GACGTTGGGA TGAGAATGAG AGT------- --------GA GGAGGAAGAA CATCTTCCAA CTGAAAGCAT GACAACATCT 1243**

**He185/333_gDNA_0008 ACGTCCATTC GGCAGACGCA ACCATACCGA AGAAGGATCT CCCAGGCGCG ATGGC----- ---------- ---AACCGTG GACGTTGGGA TGAGAATGAA AGT------- --------GT GGAGGAAGAA CATCTTCCGA CTGAAAGCAT GACAACATCT 1357**

**He185/333_gDNA_0009 ACATCCATTC GGCAGACGCA ACCATACCGA AGAAGGATCT CCCAGGCGCG ATGGAGATCG TCGTCCCAAT GGCAACCGTG GACGTTGGGA TGAGAATGAA AGT------- --------GA GGAGGAAGAA CATCTTCCAA CTGAAAGCAT GACAACATCT 1353**

**He185/333_gDNA_0010 ACGTCAATTC GGCAGACGCA ACCATACCGA AGAAGGATCT CCCAGGCGCG ATGGC----- ---------- ---AACCGTG GACGTTGGGA TGAGAATGAA AGT------- --------GT GGAGGAAGAA CATCTTCCGA CTGAAAGCAT GACAACATCT 1358**

**He185/333_gDNA_0011 ACGTCCATTC GGCAGACGCA ACCATACCGA AGAAGGATCT CCCAGGCGCG ATGGC----- ---------- ---AACCGTG GACGTTGGGA TGAGAATGAA AGT------- --------GT GGAGGAAGAA CATCTTCCGA CTGAAAGCAT GACAACATCT 1358**

**He185/333_gDNA_0012 ACGTCCATTC GGCAGACGCA ACCATACCGA AGAAGGATCT CCCAGGCGCG ATGGC----- ---------- ---AACCGTG GACGTTGGGA TGAGAATGAA AGT------- --------GT GGAGGAAGAA CATCTTCCGA CTGAAAGCAT GACAACATCT 1358**

**He185/333_gDNA_0013 ACGTCCATTC GGCAGACGCA ACCATACCGA AGAAGGATCT CCCAGGCGCG ATGGC----- ---------- ---AACCGTG GACGTTGGGA TGAGAATGAA AGT------- --------GT GGAGGAAGAA CATCTTCCGA CTGAAAGCAT GACAACATCT 1357**

**He185/333_gDNA_0014 ACGTCCATTC GACAGACGCA ACCATACCGA AGAAGGATCT CCTAGGCGCG ATGGC----- ---------- ---AACCGTG GACGTTGGGA TGAGAATGAA AGT------- --------GA GGAGGAAGAA CATCTTCCAA CTGAAAGCAT GACAACATCT 1431**

**He185/333_gDNA_0015 ACGTCCATTC GGCAGATGCA ACCATACCGA AGAAGGATCT CCCAGGCGCG ATGGAGATCG TCGTCCCAAG GGCAACCATG GACGTTGGGA TGAGAATGAA AGT------- --------GA GGAGGAAGAA CATCTTCCAA CTGAAAGCAT GACAACATCT 1243**

**He185/333_gDNA_0016 ACGTCCATTC GACAGACGCA ACCATACCGA AGAAGGATCT CCTAGGCGCG ATGGC----- ---------- ---AACCGTG GACGTTGGGA TGAGAATGAA AGT------- --------GA GGAGGAAGAA CATCTTCCAA CTGAAAGCAT GACAACATCT 1429**

**He185/333_gDNA_0017 ACGTCCATTC GACAGACGCA ACCATACCGA AGAAGGATCT CCTAGGCGCG ATGGC----- ---------- ---AACCGTG GACGTTGGGA TGAGAATGAA AGT------- --------GA GGAGGAAGAA CATCTTCCAA CTGAAAGCAT GACAACATCT 1432**

**He185/333_gDNA_0018 ACGTCCATTC GACAGACGCA ACCATACCGA AGAAGGATCT CCTAGGCGCG ATGGC----- ---------- ---AACCGTG GACGTTGGGA TGAGAATGAA AGT------- --------GA GGAGGAAGAA CATCTTCCAA CTGAAAGCAT GACAACATCT 1432**

**He185/333_gDNA_0019 ACATCCATTC GGCAGACGCA ACCATACCGA AGAAGGATCT CCCAGGCGCG ATGGAGATCG TCGTCCCAAT GGCAACCGTG GACGTTGGGA TGAGAATGAA AGT------- --------GA GGAGGAAGAA CATCTTCCAA CTGAAAGCAT GACAACATCT 1353**

**He185/333_gDNA_0020 ACGTCCATTC GACAGACGCA ACCATACCGA AGAAGGATCT CCTAGGCGCG ATGGC----- ---------- ---AACCGTG GACGTTGGGA TGAGAATGAA AGT------- --------GA GGAGGAAGAA CATCTTCCAA CTGAAAGCAT GACAACATCT 1335**

**He185/333_gDNA_0021 ACGTCCATTC GACAGACGCA ACCATACCGA AGAAGGATCT CCTAGGCGCG ATGGC----- ---------- ---AACCGTG GACGTTGGGA TGAGAATGAA AGT------- --------GA GGAGGAAGAA CATCTTCCAA CTGAAAGCAT GACAACATCT 1432**

**He185/333_gDNA_0022 ACATCCATTC GGCAGACGCA ACCATACCGA AGAAGGATCT CCCAGGCGCG ATGGAGATCG TCGTCCCAAT GGCAACCGTG GACGTTGGGA TGAGAATGAA AGT------- --------GA GGAGGAAGAA CATCTTCCAA CTGAAAGCAT GACAACATCT 1353**

**He185/333_gDNA_0023 ACGTCCATTC GACAGACGCA ACCATACCGA AGAAGGATCT CCTAGGCGCG ATGGC----- ---------- ---AACCGTG GACGTTGGGA TGAGAATGAA AGT------- --------GA GGAGGAAGAA CATCTTCCAA CTGAAAGCAT GACAACATCT 1432**

**He185/333_gDNA_0024 ACGTCCATTC GACAGACGCA ACCATACCGA AGAAGGATCT CCTAGGCGCG ATGGC----- ---------- ---AACCGTG GACGTTGGGA TGAGAATGAA AGT------- --------GA GGAGGAAGAA CATCTTCCAA CTGAAAGCAT GACAACATCT 1405**

**He185/333_gDNA_0025 ACGTCCATTC GACAGACGCA ACCATACCGA AGAAGGATCT CCTAGGCGCG ATGGC----- ---------- ---AACCGTG GACGTTGGGA TGAGAATGAA AGT------- --------GA GGAGGAAGAA CATCTTCCAA CTGAAAGCAT GACAACATCT 1432**

**He185/333_gDNA_0026 ACGTCCATTC GACAGACGCA ACCATACCGA AGAAGGATCT CCTAGGCGCG ATGGC----- ---------- ---AACCGTG GACGTTGGGA TGAGAATGAA AGT------- --------GA GGAGGAAGAA CATCTTCCAA CTGAAAGCAT GACAACATCT 1335**

**He185/333_gDNA_0027 ACGTCCATTC GGCAGACGCA ACCATACCGA AGAAGGATCT CCCAGGCGCG ATGGAGATCG TCGTCCCAAT GGCAACCGTG GACGTTGGGA TGAGAATGAA AGT------- --------GT GGAGGAAGAA CATCTTCCGA CTGAAAGCAT GACAACATCT 1429**

**He185/333_gDNA_0028 ACGTCCATTC GACAGACGCA ACCATACCGA AGAAGGATCT CCTAGGCGCG ATGGC----- ---------- ---AACCGTG GACGTTGGGA TGAGAATGAA AGT------- --------GA GGAGGAAGAA CATCTTCCAA CTGAAAGCAT GACAACATCT 1432**

**He185/333_gDNA_0029 ACGTCCATTC GACAGACGCA ACCATACCGA AGAAGGATCT CCTAGGCGCG ATGGC----- ---------- ---AACCGTG GACGTTGGGA TGAGAATGAA AGT------- --------GA GGAGGAAGAA CATCTTCCAA CTGAAAGCAT GACAACATCT 1432**

**He185/333_gDNA_0030 ACATCCATTC GGCAGACGCA ACCATACCGA AGAAGGATCT CCCAGGCGCG ATGGAGATCG TCGTCCCAAT GGCAACCGTG GACGTTGGGA TGAGAATGAA AGT------- --------GA GGAGGAAGAA CATCTTCCAA CTGAAAGCAT GACAACATCT 1354**

**He185/333_gDNA_0031 ACGTCCATTC GGCAGACGCA ACCATACCGA AGAAGGATCT CCCAGGCGCG ATGGAGATCG TCGTCCCAAT GGCAACCGTG GACGTTGGGA TGAGAATGAA AGT------- --------GT GGAGGAAGAA CATCTTCCGA CTGAAAGCAT GACAACATCT 1429**

**He185/333_gDNA_0032 ACGTCCATTC GACAGACGCA ACCATACCGA AGAAGGATCT CCTAGGCGCG ATGGC----- ---------- ---AACCGTG GACGTTGGGA TGAGAATGAA AGT------- --------GA GGAGGAAGAA CATCTTCCAA CTGAAAGCAT GACAACATCT 1431**

**He185/333_gDNA_0033 ACATCCATTC GGCAGACGCA ACCATACCGA AGAAGGATCT CCCAGGCGCG ATGGAGATCG TCGTCCCAAT GGCAACCGTG GACGTTGGGA TGAGAATGAA AGT------- --------GA GGAGGAAGAA CATCTTCCAA CTGAAAGCAT GACAACATCT 1352**

**He185/333_gDNA_0034 ACATCCATTC GGCAGACGCA ACCATACCGA AGAAGGATCT CCCAGGCGCG ATGGAGATCG TCGTCCCAAT GGCAACCGTG GACGTTGGGA TGAGAATGAA AGT------- --------GA GGAGGAAGAA CATCTTCCAA CTGAAAGCAT GACAACATCT 1353**

**He185/333_gDNA_0035 ACGTCCATTC GGCAGATGCA ACCATACCGA AGAAGGATCT CCCAGGCGCG ATGGAGATCG TCGTCCCAAT GGCAACCGTG GACGTTGGGA TGAGAATGAA AGT------- --------GA GGAGGAAGAA CATCTTCCAA CTGAAAGCAT GACAACATCT 1383**

**He185/333_gDNA_0036 ACGTCCATTC GACAGACGCA ACCATACCGA AGAAGGATCT CCTAGGCGCG ATGGC----- ---------- ---AACCGTG GACGTTGGGA TGAGAATGAA AGT------- --------GA GGAGGAAGAA CATCTTCCAA CTGAAAGCAT GACAACATCT 1405**

**He185/333_gDNA_0037 ACGTCCATTC GGCAGATGCA ACCATACCGA AGAAGGATCT CCCAGGCGCG ATGGAGATCG TCGTCCCAAT GGCAACCGTG GACGTTGGGA TGAGAATGAA AGT------- --------GA GGAGGAAGAA CATCTTCCAA CTGAAAGCAT GACAACATCT 2283**

**He185/333_gDNA_0038 ACGTCCATTC GGCAGATGCA ACCATACCGA AGAAGGATCT CCCAGGCGCG ATGGAGATCG TCGTCCCAAT GGCAACCGTG GACGTTGGGA TGAGAATGAA AGT------- --------GA GGAGGAAGAA CATCTTCCAA CTGAAAGCAT GACAACATCT 2283**

**He185/333_gDNA_0039 ACATCCATTC GGCAGACGCA ACCATACCGA AGAAGGATCT CCCAGGCGCG ATGGAGATCG TCGTCCCAAT GGCAACCGTG GACGTTGGGA TGAGAATGAA AGT------- --------GA GGAGGAAGAA CATCTTCCAA CTGAAAGCAT GACAACATCT 1353**

Stop

3’UTR

**....|....| ....|....| ....|....| ....|....| ....|....| ....|....| ....|....| ....|....| ....|....| ....|....| ....|....| ....|....| ....|....| ....|....| ....|....| ....|....|**

30

31

29

**2565 2575 2585 2595 2605 2615 2625 2635 2645 2655 2665 2675 2685 2695 2705 2715**

**He185/333_gDNA_0001 GTAGTGCCTG ATGTGGTCGA GATCGACATC AACGAAATAG ACAGCAACAT TATCCCCGAG GTGTAGAATT TATCAAGACA ATTACATTCC TGAAAG---- ---------- ---------- ---------- ---------- ---------- ---------- 1454**

**He185/333_gDNA_0002 GCAGTGCCTG ATGTGGTCGA GATCGACATC AACGAAATAG ACAGCAACAT TATCCCCGAG GTGTAGAATT TATCAAGACA ATTACATTCC TGAAAG---- ---------- ---------- ---------- ---------- ---------- ---------- 1453**

**He185/333_gDNA_0003 GCAGTGCCTG ATGTGGTCGA GATCGACATC AACGAAATAG ACAGCGACAT TATCCCCGAG GTGTAGAATT TATCAAGACA ATTACATTCC TGAAAG---- ---------- ---------- ---------- ---------- ---------- ---------- 1453**

**He185/333_gDNA_0004 GCAGTGCCTG ATGTGGTCGA GATTGACATC AAC------- --------AT TATCCCCGAG GTGTAGAATT TATCAAGACA ATTACATTCC TGAAAG---- ---------- ---------- ---------- ---------- ---------- ---------- 1429**

**He185/333_gDNA_0005 GTAGTGCCTG ATGTGGTCGA GATCGACATC AACGAAATAG ACAGCAACAT TATCCCCGAG GTGTAGAATT TATCAAGACA ATTACATTCC TGAAAG---- ---------- ---------- ---------- ---------- ---------- ---------- 1454**

**He185/333_gDNA_0006 GTAGTGCCTG ATGTGGTCGA GATCGACATC AACGAAATAG ACAGCAACAT TATCCCCGAG GTGTAGAATT TATCAAGACA ATTACATTCC TGAAAG---- ---------- ---------- ---------- ---------- ---------- ---------- 1453**

**He185/333_gDNA_0007 GCAGTGCCTG ATGTGGTCGA GATTGACATG AAC------- --------AT TATCCCCGAG GTGTAGAATT TATCAAGACA ATTACATTCC TGAAAG---- ---------- ---------- ---------- ---------- ---------- ---------- 1324**

**He185/333_gDNA_0008 GCAGTGCCTG ATGTGGTCGA GATCGACATC AACGAAATAG ACAGCAACAT TATCCCCGAG GTGTAGAATT TATCAAGACA ATTACATTCC TGAAAG---- ---------- ---------- ---------- ---------- ---------- ---------- 1453**

**He185/333_gDNA_0009 GTAGTGCCTG ATGTGGTCGA GATTGACATC AAC------- --------AT TATCCCCGAG GTGTAGAATT TATCAAGACA ATTACATTCC TGAAAG---- ---------- ---------- ---------- ---------- ---------- ---------- 1434**

**He185/333_gDNA_0010 GTAGTGCCTG ATGTGGTCGA GATCGACATC AACGAAATAG ACAGCAACAT TATCCCCGAG GTGTAGAATT TATCAAGACA ATTACATTCC TGAAAG---- ---------- ---------- ---------- ---------- ---------- ---------- 1454**

**He185/333_gDNA_0011 GTAGTGCCTG ATGTGGTCGA GATCGACATC AACGAAATAG ACAGCAACAT TATCCCCGAG GTGTAGAATT TATCAAGACA ATTACATTCC TGAAAG---- ---------- ---------- ---------- ---------- ---------- ---------- 1454**

**He185/333_gDNA_0012 GCAGTGCCTG ATGTGGTCGA GATCGACATC AACGAAATAG ACAGCAACAT TATCCCCGAG GTGTAGAATT TATCAAGACA ATTACATTCC TGAAAG---- ---------- ---------- ---------- ---------- ---------- ---------- 1454**

**He185/333_gDNA_0013 GTAGTGCCTG ATGTGGTCGA GATCGACATC AACGAAATAG ACAGCAACAT TATCCCCGAG GTGTAGAATT TATCAAGACA ATTACATTCC TGAAAG---- ---------- ---------- ---------- ---------- ---------- ---------- 1453**

**He185/333_gDNA_0014 GCAGTGCCTG ATGTGGTCGA GATCGACATC AACGAAATAG ACATCAACAT TATCCCCGAG GTGTAGAATT TATCAAGACA ATTACATTCC TGAAAG---- ---------- ---------- ---------- ---------- ---------- ---------- 1527**

**He185/333_gDNA_0015 GCAGTGCCTG ATGTGGTCGA GATTGACATC AAC------- --------AT TATCCCCGAG GTGTAGAATT TATCAAGACA ATTACATTCC TGAAAG---- ---------- ---------- ---------- ---------- ---------- ---------- 1324**

**He185/333_gDNA_0016 GCAGTGCCTG ATGTGGTCGA GATCGACATC AACGAAATAG ACATCAACAT TATCCCCGAG GTGTAGAATT TATCAAGACA ATTACATTCC TGAAAGTAAA CAATTGTCAC TGATGTCTTT TCCATCAAAA ATATGCGTGC AAAGAACATA ATATCTTTGA 1589**

**He185/333_gDNA_0017 GCAGTGCCTG ATGTGGTCGA GATCGACATC AACGAAATAG ACATCAACAT TATCCCCGAG GTGTAGAATT TATCAAGACA ATTACATTCC TGAAAGTAAA CAATTGTCAC TGATGTCTTT TCCATCAAAA ATATGCGTGC AAAGAACATA ATATCTTTGA 1592**

**He185/333_gDNA_0018 GCAGTGCCTG ATGTGGTCGA GATCGACATC AACGAAATAG ACATCAACAT TATCCCCGAG GTGTAGAATT TATCAAGACA ATTACATTCC TGAAAGTAAA CAATTGTCAC TGATGTCTTT TCCATCAAAA ATATGCGTGC AAAGAACATA ATATCTTTGA 1592**

**He185/333_gDNA_0019 GTAGTGCCTG ATGTGGTCGA GATTGACATC AAC------- --------AT TATCCCCGAG GTGTAGAATT TATCAAGACA ATTACATTCC TGAAAGTAAA CAATAGTCAC TGATGTCTTT TCCATCATAT ATATGCCTGC AAAGAACATG ATATCTTTGA 1498**

**He185/333_gDNA_0020 GCAGTGCCTG ATGTGGTCGA GATCGACATC AACGAAATAG ACATCAACAT TATCCCCGAG GTGTAGAATT TATCAAGACA ATTACATTCC TGAAAGTAAA CAATTGTCAC TGATGTCTTT TCCATCAAAA ATATGCGTGC AAAGAACATA ATATCTTTGA 1495**

**He185/333_gDNA_0021 GCAGTGCCTG ATGTGGTCGA GATCGACATC AACGAAATAG ACATCAACAT TATCCCCGAG GTGTAGAATT TATCAAGACA ATTACATTCC TGAAAGTAAA CAATTGTCAC TGATGTCTTT TCCATCAAAA ATATGCGTGC AAAGAACATA ATATCTTTGA 1592**

**He185/333_gDNA_0022 GTAGTGCCTG ATGTGGTCGA GATTGACATC AAC------- --------AT TATCCCCGAG GTGTAGAATT TATCAAGACA ATTACATTCC TGAAAGTAAA CAATAGTCAC TGATGTCTTT TCCATCATAT ATATGCCTGC AAAGAACATG ATATCTTTGA 1498**

**He185/333_gDNA_0023 GCAGTGCCTG ATGTGGTCGA GATCGACATC AACGAAATAG ACATCAACAT TATCCCCGAG GTGTAGAATT TATCAAGACA ATTACATTCC TGAAAGTAAA CAATTGTCAC TGATGTCTTT TCCATCAAAA ATATGCGTGC AAAGAACATA ATATCTTTGA 1592**

**He185/333_gDNA_0024 GCAGTGCCTG ATGTGGTCGA GATCGACATC AACGAAATAG ACATCAACAT TATCCCCGAG GTGTAGAATT TATCAAGACA ATTACATTCC TGAAAGTAAA CAATTGTCAC TGATGTCTTT TCCATCAAAA ATATGCGTGC AAAGAACATA ATATCTTTGA 1565**

**He185/333_gDNA_0025 GCAGTGCCTG ATGTGGTCGA GATCGACATC AACGAAATAG ACATCAACAT TATCCCCGAG GTGTAGAATT TATCAAGACA ATTACATTCC TGAAAGTAAA CAATTGTCAC TGATGTCTTT TCCATCAAAA ATATGCGTGC AAAGAACATA ATATCTTTGA 1592**

**He185/333_gDNA_0026 GCAGTGCCTG ATGTGGTCGA GATCGACATC AACGAAATAG ACATCAACAT TATCCCCGAG GTGTAGAATT TATCAAGACA ATTACATTCC TGAAAGTAAA CAATTGTCAC TGATGTCTTT TCCATCAAAA ATATGCGTGC AAAGAACATA ATATCTTTGA 1495**

**He185/333_gDNA_0027 GCAGTGCCTG ATGTGGTCGA GATCGACATC AACGAAATAG ACATCAACAT TATCCCCGAG GTGTAGAATT TATCAAGACA ATTACATTCC TGAAAGTAAA CAATTTTCAC TGATGTCTTT TCCATCAAAA ATATGCCTGC AAAGAACATA ATATCTTTGA 1588**

**He185/333_gDNA_0028 GCAGTGCCTG ATGTGGTCGA GATCGACATC AACGAAATAG ACATCAACAT TATCCCCGAG GTGTAGAATT TATCAAGACA ATTACATTCC TGAAAGTAAA CAATTGTCAC TGATGTCTTT TCCATCAAAA ATATGCGTGC AAAGAACATA ATATCTTTGA 1592**

**He185/333_gDNA_0029 GCAGTGCCTG ATGTGGTCGA GATCGACATC AACGAAATAG ACATCAACAT TATCCCCGAG GTGTAGAATT TATCAAGACA ATTACATTCC TGAAAGTAAA CAATTGTCAC TGATGTCTTT TCCATCAAAA ATATGCGTGC AAAGAACATA ATATCTTTGA 1592**

**He185/333_gDNA_0030 GTAGTGCCTG ATGTGGTCGA GATTGACATC AAC------- --------AT TATCCCCGAG GTGTAGAATT TATCAAGACA ATTACATTCC TGAAAGTAAA CAATAGTCAC TGATGTCTTT TCCATCATAT ATATGCCTGC AAAGAACATG ATATCTTTGA 1498**

**He185/333_gDNA_0031 GCAGTGCCTG ATGTGGTCGA GATCGACATC AACGAAATAG ACATCAACAT TATCCCCGAG GTGTAGAATT TATCAAGACA ATTACATTCC TGAAAGTAAA CAATAGTCAC TGATGTCTTT TCCATCATAT ATATGCCTGC AAAGAACATG ATATCTTTGA 1589**

**He185/333_gDNA_0032 GCAGTGCCTG ATGTGGTCGA GATCGACATC AACGAAATAG ACATCAACAT TATCCCCGAG GTGTAGAATT TATCAAGACA ATTACATTCC TGAAAGTAAA CAATTGTCAC TGATGTCTTT TCCATCAAAA ATATGCGTGC AAAGAACATA ATATCTTTGA 1591**

**He185/333_gDNA_0033 GTAGTGCCTG ATGTGGTCGA GATTGACATC AAC------- --------AT TATCCCCGAG GTGTAGAATT TATCAAGACA ATTACATTCC TGAAAGTAAA CAATAGTCAC TGATGTCTTT TCCATCATAT ATATGCCTGC AAAGAACATG ATATCTTTGA 1497**

**He185/333_gDNA_0034 GTAGTGCCTG ATGTGGTCGA GATTGACATC AAC------- --------AT TATCCCCGAG GTGTAGAATT TATCAAGACA ATTACATTCC TGAAAGTAAA CAATAGTCAC TGATGTCTTT TCCATCATAT ATATGCCTGC AAAGAACATG ATATCTTTGA 1498**

**He185/333_gDNA_0035 GCAGTGCCTG ATGTGGTCGA GATTGACATC AAC------- --------AT TATCCCCGAG GTGTAGAATT TATCAAGACA ATTACATTCC TGAAAGTAAA CAATAGTCAC TGATGTCTTT TCCATCAAAA ATATGCGTGC AAAGAACATA ATATCTTTGA 1528**

**He185/333_gDNA_0036 GCAGTGCCTG ATGTGGTCGA GATCGACATC AACGAAATAG ACATCAACAT TATCCCCGAG GTGTAGAATT TATCAAGACA ATTACATTCC TGAAAGTAAA CAATTGTCAC TGATGTCTTT TCCATCAAAA ATATGCGTGC AAAGAACATA ATATCTTTGA 1565**

**He185/333_gDNA_0037 GCAGTGCCTG ATGTGGTCGA GATTGACATC AAC------- --------AT TATCCCCGAG GTGTAGAATT TATCAAGACA ATTACATTCC TGAAAGTAAA CAATAGTCAC TGATGTCTTT TCCATCATAT ATATGCCTGC AAAGAACATA ATATCTTTGA 2428**

**He185/333_gDNA_0038 GCAGTGCCTG ATGTGGTCGA GATTGACATC AAC------- --------AT TATCCCCGAG GTGTAGAATT TATCAAGACA ATTACATTCC TGAAAGTAAA CAATAGTCAC TGATGTCTTT TCCATCATAT ATATGCCTGC AAAGAACATA ATATCTTTGA 2428**

**He185/333_gDNA_0039 GTAGTGCCTG ATGTGGTCGA GATTGACATC AAC------- --------AT TATCCCCGAG GTGTAGAATT TATCAAGACA ATTACATTCC TGAAAGTAAA CAATAGTCAC TGATGTCTTT TCCATCATAT ATATGCCTGC AAAGAACATG ATATCTTTGA 1498**

**....|....| .**

3’UTR

**2725**

**He185/333_gDNA_0001 ---------- - 1454**

**He185/333_gDNA_0002 ---------- - 1453**

**He185/333_gDNA_0003 ---------- - 1453**

**He185/333_gDNA_0004 ---------- - 1429**

**He185/333_gDNA_0005 ---------- - 1454**

**He185/333_gDNA_0006 ---------- - 1453**

**He185/333_gDNA_0007 ---------- - 1324**

**He185/333_gDNA_0008 ---------- - 1453**

**He185/333_gDNA_0009 ---------- - 1434**

**He185/333_gDNA_0010 ---------- - 1454**

**He185/333_gDNA_0011 ---------- - 1456**

**He185/333_gDNA_0012 ---------- - 1454**

**He185/333_gDNA_0013 ---------- - 1453**

**He185/333_gDNA_0014 ---------- - 1527**

**He185/333_gDNA_0015 ---------- - 1324**

**He185/333_gDNA_0016 GGTAGACGGG A 1600**

**He185/333_gDNA_0017 GGTAGACGGG A 1603**

**He185/333_gDNA_0018 GGTAGACGGG A 1603**

**He185/333_gDNA_0019 CGTACAAAGG A 1509**

**He185/333_gDNA_0020 GGTAGACGGG A 1506**

**He185/333_gDNA_0021 GGTAGACGGG A 1603**

**He185/333_gDNA_0022 CGTACAAAGG A 1509**

**He185/333_gDNA_0023 GGTAGACGGG A 1603**

**He185/333_gDNA_0024 GGTAGACGGG A 1576**

**He185/333_gDNA_0025 GGTAGACGGG A 1603**

**He185/333_gDNA_0026 GGTAGACGGG A 1506**

**He185/333_gDNA_0027 GGTAGACGGG A 1600**

**He185/333_gDNA_0028 GGTAGACGGG A 1603**

**He185/333_gDNA_0029 GGTAGACGGG A 1603**

**He185/333_gDNA_0030 CGTACAAAGG A 1509**

**He185/333_gDNA_0031 CGTACAAAGG A 1600**

**He185/333_gDNA_0032 GGTAGACGGG A 1602**

**He185/333_gDNA_0033 CGTACAAAGG A 1509**

**He185/333_gDNA_0034 CGTACAAAGG A 1509**

**He185/333_gDNA_0035 GGTAGACGGG A 1539**

**He185/333_gDNA_0036 GGTAGACGGG A 1576**

**He185/333_gDNA_0037 CGTACAAAGG A 2439**

**He185/333_gDNA_0038 CGTACAAAGG A 2439**

**He185/333_gDNA_0039 CGTACAAAGG A 1509**
