# Supplementary material for: Characterization of the Highly Variable Immune Response Gene Family, He185/333, in the Sea Urchin, Heliocidaris erythrogramma
Source: PLoS One. 2014 Oct 21;9(10):e62079. doi: 10.1371/journal.pone.0062079 (PMC4204807; doi:10.1371/journal.pone.0062079)
Supplement: Figure S4 — Alignment of 112 deduced He185/333 polypeptides generated in Clustal W and BioEdit. The polypeptide sequences were deduced from the cDNA sequences shown in Fig. S2. The 26 elements are numbered along the top and separated by vertical black lines. Four types of repeats with tandem or interspersed-incomplete structures are highlighted in differently coloured boxes and labeled along the top. Also indicated along the top are glycine- and histidine-rich regions (orange and magenta arrows, respectively), predicted O-linked and N-linked glycosylation sites (red and blue triangles, respectively), as well as predicted serine, threonine and tyrosine phosphorylations (red, blue and green lightning bolts, respectively). (DOC) [file pone.0062079.s004.doc]

Leader

1

2

3

4

5

8

10

7

12

13

14

15

6

9

11

Repeat 1

Repeat 1

Repeat 1

**....|....| ....|....| ....|....| ....|....| ....|....| ....|....| ....|....| ....|....| ....|....| ....|....| ....|....| ....|....| ....|....| ....|....| ....|....| ....|....|**

**5 15 25 35 45 55 65 75 85 95 105 115 125 135 145 155**

**He185/333_cDNA_0001 MELKVTLIVT LVAAITISVH AQREPGGRGN GRERGQGRFG GRPGSDRSQM MGGPRQGGPP MGGRRFD--- ---------- ---------- ---------- ---------- ---------- ---------- ---------- ---------- ---------- 67**

**He185/333_cDNA_0002 MELKVTLIVT LVAAITISVH AQREPGGRGN GRERGQGRFG GRPGSDRSQM MGGPRQGGPP MGGRRFDGPG QGDQQMDGRG PNGGPMGGRR FDGPGFGGFR LEGAGRPFFG HGGRHADGEG EMEAAQPIGD GQGWPGRFDG PGRFSGRPYP GRGGHHGHHH 160**

**He185/333_cDNA_0003 MELKVTLIVT LVAAITISVH AQREPGGRGN GRERGQGRFG GRPGSDRSQM MGGPRQGGPP MGGRRFDGPG QGDQRMDGRG PNGGPMGGRR FDGPGFGGFR LEGAGRPFFG HGGRHADGEG EMEAAQPIGD GQGWPGRFDG PGRFSGRPYP GRGGHHGHHH 160**

**He185/333_cDNA_0004 MELKVTLIVT LVAAITISVH AQREPGGRGN GRERGQGRFG GRPGSDRSQM MGGPRQGGPP MGGRRFDGPG QGDQQMDGRG PNGGPMGGRR FDGPGFGGFR LEGAGRPFFG HGGRHADGEG EMEAAQPIGD GQGWPGRFDG PGRFSGRPYP GRGGHHGHHH 160**

**He185/333_cDNA_0005 MELKVTLIVT LVAAITISVH AQREPGGRGN GRERGQGRFG GRPGSGRSQM MGGPRQGGPP MGGRRFDGPG QGDQQMDGRG PNGGPMGGRR FDGPGFGGFR LEGAGRPFFG HGGRHADGEG EMEAAQPIGD GQGWPGRFDG PGRFSGRPYP GRGGHHGHHH 160**

**He185/333_cDNA_0006 MELKVTLIVT LVAAITISVH AQREPGGRGN GRERGQGRFG GRPGSDRSQM MGGPRQGGPP MGGRRFDGPG QGDQQMD--- ---------- ---------- ---------- ---------- ---------- ---------- -----GRPYP GRGGHHGHHH 92**

**He185/333_cDNA_0007 MELKVTLIVT LVAAITISVH AQREPGGRGN GRERGQGRFG GRPGSDRSQM MGGPRQGGPP MGGRRFDGPG QGDQQMDGRG PNGGPMGGRR FDGPGFGGFR LEGAGRSFFG HGGRHADGEG EMEAAQPTGD GQGWPGRFDG PGRFSGRPYP GRGGHHGHHH 160**

**He185/333_cDNA_0008 MELKVTLIVT LVAAITISVH AQREPGGRGN GRERGQGRFG GRPGSDRSQM MGGPRQGGPP MGGRRFDGPG QGDRQMDGRG PNGGPMGGRR FDGPGFGGFR LEGAGRPFFG HGGRHADGEG EMEAAQPIGD GQGWPGRFDG PGRFSGRPYP GRGGHHGHHH 160**

**He185/333_cDNA_0009 MELKVTLIVT LVAAITISVH AQREPGGRGN GRERGQGRFG GRPGSDRSQM MGGPRQGGPP MGGRRFDGPG QGDQQMDGRG PNGGPMGGRR FDGPGFGGFR LEGAGRPFFG HGGRHADGEG EMEAAQPIGD GQGWPGRFDG PGRFSGRPYP GRGGHHGHHH 160**

**He185/333_cDNA_0010 MELKVTLIVT LVAAITISVH AQREPGGRGN GRERGQGRFG GRPGSDRSQM MGGPRQGGPP MGGRRFDGPG QGDQQMDGRG PNGGPMGGRR FDGPGFGGFR LEGAGRPFFG HGGRHADGEG EMEAAQPIGD GQGWPGRFDG PGRFSGRPYP GRGGHHGHHH 160**

**He185/333_cDNA_0011 MELKVTLIVT LVAAITISVH AQREPGGRGN GRERGQGRFG GRPGSDRSQM MGGPRQGGPP MGGRRFDGPG QGDQQMDGRG PNGGPMGGRR FDGPGFGGFR LEGAGRPFFG HGGRHADGEG EMEAAQPIGD GQGWPGRFDG PGRFSGRPYP GRGGHHGHHH 160**

**He185/333_cDNA_0012 MELKVTLIVA FVTAITISVH AQRARGGRRN GRERGQGRFG GSPGSDRPQM TGGPRQGGPP MGGRRFDGPG QGDQQMDGRG PNGGPMGGRR FDGPGFGGFR PEGAGRPFFG HGGMHADGEG EMEVAQPIGD GQGWPGRFDG PGRFSGRPYP GRDG------ 154**

**He185/333_cDNA_0013 MELKVTQIVT LVAAITISIH AQREPGGRGN GRERGQGRFG GRPGSDRSQM MGGPRQGGPP MGGRRFDGPG QGDQQMDGRG PNGGPMGGRR FDGPGFGGFR LEGAGRPFFG HGGRHADGEG EMGAAQPIGD GQGWPGRFDG PGRFSGRPYP GRGGHHGHHH 160**

**He185/333_cDNA_0014 MELKVTLIVA LVAAITISVH AQRARGGRGY GRKRGQGRFG GSPGSDRPQM TGGPRQGGPP MGGRRFDGPG QGDQQMDGRG PNGGPMGGRR FDGPGFGGFR PEGAGRPFFG QGGMHADGEG EMEVAQPIGD GQGWPGRFDG PGRFSGRPYP GRDG------ 154**

**He185/333_cDNA_0015 MELKVTLIVA LVTAITISVH AQRARGGRRN GRERGQGRFG GSPGSDRPQM TGGPRQGGPP MGGRRFDGPG QGDQQMDGRG PNGGPMGGRR FDGPGFGGFR PEGAGRPFFG HGGMHADGEG EMEVAQPIGD GQGWPGRFDG PGRFSGRPYP GRDG------ 154**

**He185/333_cDNA_0016 MELKVTLIVA LVTAITISVH AQRARGGRRN GRVRGQGRFG GSPGSDRSQM TGGPRQGGPP MGGRRCDGPG QGDQQMDGRG PNGGPMGGRR FDGPGFGGFR PEGAGRPFFG HGGMHADGEG EMEVAQPIGD GQGWPGRFDG PGRFSGRPYP GRDG------ 154**

**He185/333_cDNA_0017 MELKVTLIVT LVAAITISVH AQREPGGRGN GRERGQGRFG GRPGSDRSQM MGGPRQGGPP MGGRRFDGPG QGDQQMDGRG PNGGPMGGRR FDGPGFGGFR LEGAGRPFFG HGGRHADGEG EMEAAQPIGD GQGWPGRFDG PGRFSGHPHP GRGGHHGHHH 160**

**He185/333_cDNA_0018 MELKVTLIVT LVAAITISVH AQREPGGRGN GRERGQGRFG GRPGSDRSQM MGGPRQGGPP MGGRRFDGPG QGDQQMDGRG PNGGPMGGRR FDGPGFSGFR LEGAGRPFFG HGGRHADGEG EMEAAQPIGD GQGWPGRFDG PGRFSGRPYP GRGGHHGHHH 160**

**He185/333_cDNA_0019 MELKVTLIVT LVAAITISVH AQREPGGRGN GRERGQGRFG GRPGSDRSQM MGGPRQGGPP MGGRRFDGPG QGDQQMDGRG PNGGPMGGRR FDGPGFGGFR LEGAGRPFFG HGGRHADGEG EMEAAQSIGD GQGWPGRFDG PGRFSGRPYP GRGGHHGHHH 160**

**He185/333_cDNA_0020 MELKVTLIVT LVAAITISVH AQREPGG--- ---------- -RPGSDRSQM MGGPRQGGPP MGGRRFDGPG QGDQQMDGRG PNGGPMGGRR FDGPGFGGFR LEGAGRPFFG HGGRHADGEG EMEAAQPIGD GQGWPGRFDG PGRFSGRPYP GRGGHHGHHH 146**

**He185/333_cDNA_0021 MELKVTLIVT LVAAITISVH AQREPGGRGN GRERGQGRFG GRPGSDRSQM MGGPRQGGPP MGGRRFDGPG QGDQQMDGRG PNGGPMGGRR FDGPGFGGFR LEGAGRPFFG HGGRHADGEG EMEAAQPIGD GQGWPGRFDG PGRFSGRPYP GRGGHHGHHH 160**

**He185/333_cDNA_0022 MELKVTLIVA LVTAITISVH AQRARGGRRN GRES------ ---------- ---------- ---------- ---------- ---------- ---------- ---------- ---------- ---------- ---------- ---------- ---------- 34**

**He185/333_cDNA_0023 MELKVTLIVA LVAAITISVH AQRGRGGRGN GKERGQGRFG GRPGS-RPQM MGGHRQGGPP MGGRRFDGNG QGDQQMAGRE PNGRPVGSRR FDGPGFGGFR PEGAGRPFFG HGGMHADGEG EMEVPQPIGD GQGWPDRFDG PRRFSGRPYP GRGG------ 153**

**He185/333_cDNA_0024 MELKVTLIVA LVAAITISVH AQRARGGRGY GRKRGQGRFG GSPGSDRPQM TGGPRQGGPP MGGRRFDGPG QGDQQMDGRG PNGGPMGGRR FDGPGSGGFR PEGAGRPFFG QGGMHADGEG EMEVAQPIGD GQGWPGRFDG PGRFSGRPYP GRDG------ 154**

**He185/333_cDNA_0025 MELKVTLIVA LVAAITISVH AQRGRGGRGN GRERGQGRFG GRPGS-RPQM MGGHRQGGPP MGGRRFDGNG QGDQQMAGRE PNGRPVGSRK FDGPGFGGFR PEGAGRPFFG HGGMHADGEG EMEVPQPIGD GQGWPDRFDG PRRFSGRPYP GRGG------ 153**

**He185/333_cDNA_0026 MELKVTLIVA LVAAITISVH AQRARGGRGY GRKRGQGRFG GSPGSDRPQM TGGPRQGGPP MGGRRFDGPG QGDQQMDGRG PNGGPMGGRR FDGPGFGGFR PEGAGRPFFG QGGMHADGEG EMEVAQPIGD GQGWPGRFDG PGRFSGRPYP GRDG------ 154**

**He185/333_cDNA_0027 MELKVTLIVA LVAAITISVH AQRGRGGRGN GKERGQGRFG GRPGS-RPQM MGGHRQGGPP MGGRRFDGNG QGDQQMAGRE PNGRPVGSRK FDGPGFGGFR PEGAGRPFFG HGGMHADGEG EMEVPQPIGD GQGWPDRFDG PRRFSGRPYP GRGG------ 153**

**He185/333_cDNA_0028 MELKVTLIVA LVAAITISVH AQRARGGRGY GRKRGQGRFG GSPGSDRPQM TGGPRQGGPP MGGRRF---- ---------- ---------- ---------- -EGAGRPFFG QGGMHADGEG EMEVAQPIGD GQGWPGRFDG PGRFSGRPYP GRDG------ 119**

**He185/333_cDNA_0029 MELKVTLIVA LVAAITISVH AQRGRGGRGN GRERGQGRFG GRPGS-RPQM MGGHRQGGPP MGGRRFDGNG QGDQQMAGRE PNGRPVGSRK FDGPGFGGFR PEGAGRPFFG HGGMHADGEG EMEVPQPIGD GQGWPDRFDG PRRFSGRPYP GRGG------ 153**

**He185/333_cDNA_0030 MELKVTLIVA LVAAITISVH AQRARGGRGY GGKRGQGRFG GSPGSDRPQM TGGPRQGGPP MGGRRFDGPG QGDQQMDGRG PNGGPMGGRR FDGPGFGGFR PEGAGRLFFG QGGMHADGEG EMEVAQPIGD GQGWPGRFDG PGRFSGRPYP GRDG------ 154**

**He185/333_cDNA_0031 MELKVTLIVA LVAAITISVH AQRGRGGRGN GKERGQGRFG GRPGS-RPQM MGGHRQGGPP MGGRRFDGNG QGDQQMAGRE PNGRPVGSRK FDGPGFGGFR PEGAGRPFFG HGGMHADGEG EMEVPQPIGD GQGWPDRFDG PRRFSGRPYP GRGG------ 153**

**He185/333_cDNA_0032 MELKVTLIVA LVAAITISVH AQRARGGRGY GRKRGQGRFG GSPGSDRPQM TGGPRQGGPP MGGRRF---- ---------- ---------- ---------- -EGAGRPFFG QGGMHADGEG EMEVAQPIGD GQGWPGRFDG PGRFSGRPYP GRDG------ 119**

**He185/333_cDNA_0033 MELKVTLIVA LVAAITISVH AQRGRGGRGN GRERGQGRFG GRPGS-RPQM MGGHRQGGPP MGGRRFDGNG QGDQQMAGRE PNGRPVGSRK FDGPGFGGFR PEGAGRPFFG HGGMHADGEG EMEVPQPIGD GQGWPDRFDG PRRFSGRPYP GRGG------ 153**

**He185/333_cDNA_0034 MELKVTLIVA LVAAITISVH AQRGRGGRGN GRERGQGRFG GRPGS-RPQM MGGHRQGGPP MGGRRFDGNG QGDQQMAGRE PNGRPVGSRK FDGPGFGGFR PEGAGRPFFG HGGMHADGEG EMEVPQPIGD GQGWPDRFDG PRRFSGRPYP GRGG------ 153**

**He185/333_cDNA_0035 MELKGTLIVA ILAAITMSVH AQRDRGGRGN ARERGQGRFG GRPGSNRPQM MGGHRQGGPP MGGRRFDGPG QGDQQMAGRE PNGRPVGSRK FDGPGFGGFR PEGAGRPFFG HGGRHADGEG EMEAAQPIGD GQGWPGRFDG PGRFSGRPYP GRGGHHGHHH 160**

**He185/333_cDNA_0036 MELKVTLIVA LVAAITISAH AQRERGGRGN GRERGQGRFG GRPGSDRSQM TGGPRQGGAP MGGRRFDGPG QGDQQMDGRG PNGGPMGGRR FDGPGFGGFR PEGAGRPFFG HGGRHADGEG EMEAAQPIGD GQGWPGRFDG PGRFSGHPHP GRGGHHGHHH 160**

**He185/333_cDNA_0037 MELKVTLIVA LVAAITISVH AQRARGGRGY GRKRGQGRFG GSPGPDRPQM TGGPRQGGPP MGGRRFDGPG QGDQQMDGRG PNGGPMGGRR FDGPGFGGFR PEGAGRPFFG QGGMHADGEG EMEVAQPIGD GQGWPGRFDG PGRFSGRPYP GRDG------ 154**

**He185/333_cDNA_0038 MELKVTLIVT LVAAITISVH AQREPGGRGN GRERGQGRFG GRPGSDRSQM MGGPRQGGPP MGGRRFDGPG QGDQQMDGRG PNGGPMGGRR FDGPGFGGFR LEGAGRPFFG HGGRHADGEG EMEAAQPIGD GQGWPGRFDG PGRFSGRPYP GRGGHHGHHH 160**

**He185/333_cDNA_0039 MELKVTLIVT LVAAITISVH AQREPGGRGN GRERGQGRFG GRPGSDRSQM MGGPRQGGPP MGGRRFDGPG QGDQQMDGRG PNGGPMGGRR FDGPGFGGFR LEGAGRPFFG HGGRHADGEG EMEAAQPIGD GQGWPGRFDG PGRFSGRPYP GRGGHHGHHH 160**

**He185/333_cDNA_0040 MELKVTLIVA LVAAITISVH AQREPGGRGN GRERGQGRFR GRPGSDRSQM MGGPRQGGPP MGGRRFDGPG QGDQQMDGRG PNGGPMGGRR FDGPGFGGFR PEGAGRPFFG HGGRHADGEG EMEVPQPIGD GQGWPGRFDG PGRFSGRPYP GRGGHHEHHH 160**

**He185/333_cDNA_0041 MELKVTLIVT LVAAITISVH AQREPGGRGN GRERGQGRFG GRPGSDRSQM MGGPRQGGPP MGGRRFDGPG QGDQQMDGRG PNGGPMGGRR FDGPGFGGFR LEGAGRPFFG HGGRHADGEG EMEAAQPIGD GQGWPGRFDG PGRFSGRPYP GRGGHHGHHH 160**

**He185/333_cDNA_0042 MELKVTLIVT LVAAITISVH AQREPGGRGN GRERGQGRFG GRPGSDRSQM MGGPRQGGPP MGGRRFDGPG QGDQQMDGRG PNGGPMGGRR FDGPGFGGFR LEGAGRPFFG HGGRHADGEG EMEAAQPIGD GQGWPGRFDG PGRFSGRPYP GRGGHHGHHH 160**

**He185/333_cDNA_0043 MELKVTLIVA LVAAITISVH AQRGRGGRGN GKERGQGRFG GRPGS-RPQM MGGHRQGGPP MGGRRFDGNG QGDQQMAGRE PNGRPVGSRK FDGPGFGGFR PEGAGRPFFG HGGMHADGEG EMEVPQPIGD GQGWPDRFDG PRRFSGRPYP GRGG------ 153**

**He185/333_cDNA_0044 MELKVTLIVT LVAAITISVH AQREPGGRGN GRERGQGRFG GRPGSDRSQM MGGPRQGGPP MGGRRFDGPG QGDQQMDGRG PNGGPMGGRR FDGPGFGGFR LEGAGRPFFG HGGRHADGEG EMEAAQPIGD GQGWPGRFDG PGRFSGRPYP GRGGHHGHHH 160**

**He185/333_cDNA_0045 MELKVTLIVA LVAAITISVH AQREPGGRGN GRERGQGRFR GRPGSDRSQM MG*------- ---------- ---------- ---------- ---------- ---------- ---------- ---------- ---------- ---------- ---------- 52**

**He185/333_cDNA_0046 MELKVTLIVA LVAAITISVH AQREPGGRGN GRERGQGRFR GRPGSDRSQM MGGPRQGGPP MGGRRFDGPG QGDQQMDGRG PNGGPMGGRR FDGPGFGGFR PEGAGRPFFG HGGRHADGEG EMEVPQPIGD GQGWPGRFDG PGRFSGRPYP GRGGHHGHHH 160**

**He185/333_cDNA_0047 MELKVTLIVA LVAAITISVH AQRARGGRGY GRKRGQARFG GSPGSDRPQM TGGPRQGGPP MGGRRFDGPG QGDQQMDGRG PNGGPMGGRR FDGPGFGGFR PEGAGRPFFG QGGMHADGEG EMEVAQPIGD GQGWPGRFDG PGRFSGRPYP GRDG------ 154**

**He185/333_cDNA_0048 MELKVTLIVT LVAAITISVH AQREPGGRGN GRERGQGRFG GRPGSDRSQM MGGPRQGGPP MGGRRFDGPG QGDQLMDGRG PNGGPMGGRR FDGPGFGGFR LEGAGRPFFG HGGRHADGEG EMEAAQPIGD GQGWPGRFVG PGRFSGRPYP GRGGHHGHHH 160**

**He185/333_cDNA_0049 MELKVTLIVT LVAAITISVH AQREPGGRGN GRERGQGRFG GRPGSDRSQM MGGPRQGGPP MGGRRFDGPG QGDQQMDGRG PNGGPMGGRR FDGPGFGGFR LEGAGRPFFG HGGRHADGEG EMEAAQPIGD GQGWPGRFDG PGRFSGRPYP GRGGHHGHHH 160**

**He185/333_cDNA_0050 MELKVTLIVT LVAAITIAVH AQREPGGRGN GRERGQGRFG GRPGSDRSQM MGGPRQGGPP MGGRRFDGPG QGDQQMDGRG PNGGPMGGRR FDGPGFGGFR LEGAGRPFFG HGGRHADGEG EMEAAQPIGD GQGWPGRFDG PGRFSGRPYP GRGGHHGHHH 160**

**He185/333_cDNA_0051 MELKVTLIVA LVAAITISVH AQREPGGRGN GRERGQGRFR GRPGSDRSQM MGGPRQGGPP MGGRRFDGPG QGDQQMDGRG PNGGPMGGRR FDGPGFGGFR PEGAGRPFFG HGGRHADGEG EMEVPQPIGD GQGWPGRFDG PGRFSGRPYP GRGGHHGHHH 160**

**He185/333_cDNA_0052 MELKVTLIVT LVAAITISVH AQREPGGRGN GRERGQGRFG GRPGSDRSQM MGGPRQGGPP MGGRRF---- ---------- ---------- -DGPGFGGFR LEGAGRPFFG HGGRHADGEG EMEAAQPIGD GQGWPGRFDG PGRFSGRPH- ---------- 122**

**He185/333_cDNA_0053 MELKVTLIVA LVAAITISVH AQREPGGRGN GRERGQGRFR GRPGSDRSQM MGGPRQGGPP MGGRRFDGPG QGDQQMDGRG PNGGPMGGRR FDGPGFGGFR PEGAGRPFFG HGGRHADGEG EMEVPQPIGD GQGWPGRFDG PGRFSGRPYP GRGGHHGHHH 160**

**He185/333_cDNA_0054 MELKVTLIVA LVAAITISVH AQRGRGGRGN GKERGQGRFG GRPGS-RPQM MGGHRQGGPP MGGRRFDGNG QGDQQMAGRE PNGRPVGSRK FDGPGFGGFR PEGAGRPFFG HGGMHADGEG EMEVPQPIGD GQGWPDRFDG PRRFSGRPYP GRGG------ 153**

**He185/333_cDNA_0055 MELKVTLIVA LVAAITISVH AQRARGGRGY GRRRGQGRFG GSPGSDRPQM TGGPRQGGPP MGGRRFDGPG QGDQQMDGRG PNGGPMGGRR FDGPGFGGFR PEGAGRPFFG QGGMHADGEG EMEVAQPIGD GQGWPGRFDG PGRFSGRPYP GRDG------ 154**

**He185/333_cDNA_0056 MELKVTLIVA LVAAITISVH AQRARGGRGY GRKRGQGRFG GSPGFDRPQM TGGPRQGGPP MGGRRFDGPG QGDQQMDGRG PNGGPMGGRR FDGPGFGGFR PEGAGRPFFG QGGMHADGEG EMEVAQPIGD GQGWPGRFDG PGRFSGRPYP GRDG------ 154**

**He185/333_cDNA_0057 MELKVTLIVA LVAAITISAH AQRERGGRGN GRERRQGRFG GRPGSDRSQM MGGPRQGGAP MGGRRFDGPG QGDQQMDGRG PNGGPMGGRR FDGPGFGGFR PEGAGRPFFG HGGRHADGEG EMETAQPIGD GQGWPGRFDG PGRFSGHPHP GRGGHHGHHH 160**

**He185/333_cDNA_0058 MELKVTLIVA LVAAITISAH AQRERGGRGN GRERRQGRFG GRPGSDRSQM MGGPRQGGAP MGGRRFDGPG QGDQQMDGRG PNGGPMGGRR FDGPGFGGFR P--------- ---------- ---------- ---------- ---------- ---------- 101**

**He185/333_cDNA_0059 MELKVTLIVA LVAAITISAH AQRERGGRGN GRERGQGRFG GRPGSDRSQM MGGPRQGGAP MGGRRFDGPG QGDQQMDGRG PNGGPMGGRR FDGPGFGGFR PEGAGRPFFG HGGRHADGEG EMEAAQPIGD GQGWPGRFDG PGRFSGHPHP GRGGHHGHHH 160**

**He185/333_cDNA_0060 MELKVTLIVA ILAAITMSVH AQRDRGGRGN ARERGQGRFG GRPGSNRPQM MGGHRQGGPP MGGRRFDGPG QGDQQMAGRE PNGRPVGSRK FDGPGFGGFR PEGAGRPFFG HGGRHADGEG EMEAAQPIGD GQGWPGRFDG PGRFSGRPYP GRGGHHGHHH 160**

**He185/333_cDNA_0061 MELKVTLIVA LVAAITISAH AQRERGGRGN GRERGQGRFG GRPGSDRSQM MGGPRQGGAP MGGRRFDGPG QGDQQMDGRG PNGGPMGGRR FDGPGFGGFR PEGAGRPFFG HGGRHADGEG EMEAAQPIGD GQGWPGRFDG PGRFSGHPHP GRGGHHGHHH 160**

**He185/333_cDNA_0062 MELKVTLIVA LVAAITISAH AQRERGGRGN GRERGQGRFG GRPGSDRSQM MGGPRQGGAP MGGRRFDGPG QG-------- ---------- ---------- ---------- ---------- ---------- ---------- ---------- ---------- 72**

**He185/333_cDNA_0063 MELKVTLIVA LVAAITISAH AQRERGGRGN GRERGQGRFG GRPGSDRSQM MGGPRQGGAP MGGRRFDGPG QGDQQMDGRG PNGGPMGGRR FDGPGFGGFR PEGAGRPFFG HGGRHADGEG EMEAAQPIGD GQGWPGRFDG PGRFSGHPHP GRGGHHGHHH 160**

4

10

12

15

1

3

14

5

8

13

7

9

11

Leader

Repeat 1

Repeat 1

2

6

Repeat 1

**....|....| ....|....| ....|....| ....|....| ....|....| ....|....| ....|....| ....|....| ....|....| ....|....| ....|....| ....|....| ....|....| ....|....| ....|....| ....|....|**

**5 15 25 35 45 55 65 75 85 95 105 115 125 135 145 155**

**He185/333_cDNA_0068 MELKVTLIVA LVAAITISVH AQREPGGRGN GRERGQGRFR GRPGSDRSQV MGGPRQGGPP MGGRRFDGPG QGDQQMDGRG PNGGPMGGRR FDGPGFGGFR PEGAGRPFFG HGGRHADGEG EMEVPQPIGD GQGWPGRFDG PGRFSGRPYP GRGGHHGHHH 160**

**He185/333_cDNA_0069 MELKVTLIVT LVAAITTSVH AQREPGGRGN GRERGQGRFG GRP*------ ---------- ---------- ---------- ---------- ---------- ---------- ---------- ---------- ---------- ---------- ---------- 43**

**He185/333_cDNA_0070 MELKVTLIVT LVAAITISVR AQREPGGRGN GRERGQGRFG GRPGSDRSQM MGGPRQGGPP MGGRRFDGPG QGDQQMDGRG PNGGPMGGRR FDGPGFGGFR LEGAGRPFFG HGGRHADGEG EMEAAQPIGD GQGWPGRFDG PGRFSGRPYP GRGGHHGHHH 160**

**He185/333_cDNA_0071 MELKVTLIVT LVAAITISVH AQREPGGRGN GRERGQGRFG GRPGSDRSQM MGGPRQGGPP MGGRRFDGPG QGDQQMDGRG PNGGPMGGRR FDGPGFGGFR LEGAGRPFFG HGGRHADGEG EMEAAQPIGD GQGWPGRFDG PGRFSGRPYP GRGGHHGHHH 160**

**He185/333_cDNA_0072 MELKVTPIVT LVAAITISVH AQREPGGRGN GRERGQGRFG GRPGSDRSQM MGGPRQGGPP MGGRRFDGPG QGDQQMDGRG PNGGPIGGRR FDGPGFGGFR LEGAGRPFFG HGGRHADGEG EMETAQPIGD GQGWPGRFDG PGRFSGRPYP GRGGHHGHHH 160**

**He185/333_cDNA_0073 MELKVTLIVT LVAAITISVH AQREPGGRGN GRERGQGRFG GRPGSDRSQM MGGPRQGGPP MGGRRFDGPG QGDQRMDGRG PNGGPMGGRR FDGPGFDGFR LEGAGRPFFG HGGRHADGEG EMEAAQPIGD GQGWPGRFDG PGRFSGRPYP GRGGHHGHHH 160**

**He185/333_cDNA_0074 MELKVTLIVT LVAAITISVH AQREPGGRGN GRERGQGRFG GRPGSDRSQM MGGPRQGGPP MGGRRFDGPG QGDQQMDGRG PNGGPMGGRG FDGPGFGGFR LEGAGRPFFG HGGRHADGEG EMEAAQPIGD GQGWPGRFDG PGRFSGRPYP GRGGHHGHHH 160**

**He185/333_cDNA_0075 MELKATLIVA LVAAITISAH AQGERGGRGN GRERGQGRFG GRPGSDRSQM MGGPRQGGAP MGGRRFDGPG QGDQQMDGRG PNGGPMGGRR FDGPGFGGFR PEGAGRPFFG HGGRHADGEG EMEAAQPIGD GQGWPGRFDG PGRFSGHPHP GRGGHHGHHH 160**

**He185/333_cDNA_0076 MELKVTLIVT LVAAITISVH AQREPGGRGN GRERGQGRFG GRPGSDRSQM MGGPRQGGPP MGGRRFDGPG QGDQQMDGRG PNGGPMGGGR FDGPGFGGFR LEGAGRPFFG HGGRHADGEG EMEAAQPIGD GQGWPGRFDG PGRFSGRPYP GRGGHHGRHH 160**

**He185/333_cDNA_0077 MELKVTLIVA LVAAITISVH AQRARGGRGY GRKRGQGRFG GSPGSDRPQM TGGPRQGGPP MGGRRFDGPG QGDQQMDGRG PNGGPMGGRR FGGPGFGGFR PEGAGRPFFG QGGMHADGEG EMEVAQPIGD GQGWPGRFDG PGRFSGRPYP GRDG------ 154**

**He185/333_cDNA_0078 MELKVTLIVT LVAAITISVH AQREPGGRGN GRERGQGRFG GRPGSDRSQM MGGPRQGGPP MGGRRFDGPG QGDQQMDGRG PNGGPMGGRR FDGPGFGGFR LEGAGRPFFG HGGRHADGEG EMEAAQPIGD GQGWPGRFDG PGRFSGRPYP GRGGHHGHHH 160**

**He185/333_cDNA_0079 MELKVTLIVT LVAAITISVH AQREPGGRGN GRERGQGRFG GRPGSDRSQM MGGPRQGGPP MGGRRFDGPG QGDQQMDGRG PNGGPMGGRR FDGPGFGGFR LEGAGRPFFG HGGRHADGEG EMEAAQPIGD GQGWPGRFDG PGRFSGRPYP GRGGHHGHHH 160**

**He185/333_cDNA_0080 MELKVTLIVT LVAAITISVH AQREPGGRGN GRERGQGRFG GRPGSDRSQM MGGPRQGGPP MGGRRFDGPG QGDQQMDGRG PNGGPMGGRR FDGPGFGGFR LEGAGRPFFG HGGRHADGEG EMEAAQPIGD GQGWPGRFDG PGRFSGRPYP GRGGHHGHHH 160**

**He185/333_cDNA_0081 MELKVTLIVA LVAAITISVH AQRGRGGRGN GRERGQGRFG GRPGS-RPQM MGGHRQGGPP MGGRRFDGNG QGDQQMAGRE PNGRPVGSRK FDGPGFGGFR PEGAGRPFFG HGGMHADGEG EMEVPQPIGD GQGWPDRFDG PRRFSGRPYP GRGG------ 153**

**He185/333_cDNA_0082 MELKVTLIVA LVAAITISVH AQREPGGRGN GRERGQGRFR GRPGSDRSQM MGGPRQGGPP MGGRRFDGPG QGDQQMDGRG PNGGPMGGRR FDGPGFGGFR PEGAGRPFFG HGGRHADGEG EMEAAQPIGD GQGWPGRFDG PGRFSGRPYP GRGGHHGHHH 160**

**He185/333_cDNA_0083 MELKVTLIVA LVAAITISVH AQRGRGGRGN GRERGQGRFG GRPGS-RPQM MGGHRQGGPP MGGRRFDGNG QGDQQMAGRE PNGRPVGSRK FDGPGFGGFR PEGAGRPFFG HGGMHADGEG EMEVPQPIGD GQGWPDRFDG PRRFSGRPYP GRGG------ 153**

**He185/333_cDNA_0084 MELKVTLIVA LVAAITISVH AQREPGGRGN GRERGQGRFR GRPGSDRSQM MGGPRQGGPP MGGRRFDGPG QGDQQMDGRG PNGGPMGGRR FDGPGFGGFR PEGAGRPFFG HGGRHADGEG EMEAAQPIGD GQGWPGRFDG PGRFSGRPYP GRDG------ 154**

**He185/333_cDNA_0085 MELKVTLIVA LVAAITISVH AQREPGGRGN GRERGQGRFR GRPGSDRSQM MGGPRQGGPP MGGRRFDGPG QGDQQMDGRG PNGGPMGGRR FDGPGFGGFR PEGAGRPFFG HGGRHADGEG EMEAAQPIGD GQGWPGRFDG PGRFSGRPYP GRGGHHGHHH 160**

**He185/333_cDNA_0086 MELKVTLIVA LVAAITISVH AQREPGGRGN GRERGQGRFR GRPGSDRSQM MGGPRQGGPP MGGRRFDGPG QGDQQMDGRG PNGGPMGGRR FDGPGFGGFR PEGAGRPFFG QGGMHADGEG EMEVAQPIGD GQGWPGRFDG PGRFSGRPYP GRDG------ 154**

**He185/333_cDNA_0087 MELKVTLIVA LVAAITISVH AQREPGGRGN GRERGQGRFR GRPGSDRSQM MGGPRQGGPP MGGRRFDGPG QGDQQMDGRG PNGGPMGGRR FDGPGFGGFR PEGAGRPFFG QGGMHADGEG EMEVAQPIGD GQGWPGRFDG PGRFSGRPYP GRDG------ 154**

**He185/333_cDNA_0088 MELKVTLIVA LVAAITISAH AQRERGGRGN GRERGQGRFG GRPGSDRSQM MGGPRQGGAP MGGRRFDGPG QGDQQMDGRG PNGGPMGGRR FDGPGFGGFR PEGAGRPFFG HGGRHADGEG EMEAAQPIGD GQGWPGRFDG PGRFSGHPHP GRGGHHGHHH 160**

**He185/333_cDNA_0089 MELKVTLIVA LVAAITISAH AQRERGGRGN GRERGQGRFG GRPGSDRSQR MGGPRQGGAP MGGRRFDGPG QGDQQMDGRG PNGGPMGGRR FDGPGFGGFR PEGAGRPFFG HGGRHADGEG EMEAAQPIGD GQGWPGRFDG PGRFSGHPHT GRGGHHGHHH 160**

**He185/333_cDNA_0090 MELKVTLIVA LVAAITISVH AQREPGGRGN GRERGQGRFR GRPGSDRSQM MGGPRQGGPP MGGRRFDGPG QGDQQMDGRG PNGGPMGGRR FDGPGFGGFR PEGAGRPFFG QGGMHADGEG EMEVAQPIGD GQGWPGRFDG PGRFSGRPYP GRDG------ 154**

**He185/333_cDNA_0091 MELKVTLIVA LVAAITISVH AQREPGGRGN GRERGQGRFR GRPGSDRSQM MGGPRQGGPP MGGRRFDGPG QGDQQMDGRG PNGGPMGGRR FDVPGFGGFR PEGAGRPFFG QGGMHADGEG EMEVAQPIGD GQGWPGRFDG PGRFSGRPYP GRDG------ 154**

**He185/333_cDNA_0092 MELKGTLIVA LVAAITISVH AQREPGGRGN GRERGQGRFR GRPGSDRSQM MGGPRQGGPP MGGRRFDGPG QGDQQMDGRG PNGGPMGGRR FDGPGFGGFR PEGAGRPFFG HGGRHADGEG EMEAAQPIGD GQGWPGRFDG PGRFSGRPYP GRGGHHGHHH 160**

**He185/333_cDNA_0093 MELKVTLIVA LVAAITISVH AQREPGGRGN GRERGQGRFR GRPGSDRSQM MGGPRQGGPP MGGRRFDGPG QGDQQMD--- ---------- ---------- ---------- ---------- ---------- ---------- ---------- ---------- 77**

**He185/333_cDNA_0094 MELKVTLIVA LVAAITISVH AQREPGGRGN GRERGQGRFR GRPGSDRSQM MGGPRQGGPP MGGRRFDGPG QGDQQMDGRG PNGGPMGGRR FDGPGFGGFR PEGAGRPFFG HGGRHADGEG EMEAAQPIGD GQGWPGRFDG PGRFSGHPHP GRGGHHGHHH 160**

**He185/333_cDNA_0095 MELKVTLIVA LVAAITISAH AQRERGGRGN GRERGQGRFG GRPGSDRSQR MGGPRQGGAP MGGRRFDGPG QGDQQMDGRG PNGGPMGGRR FDGPGFGGFR PEGAGRPFFG HGGRHADGEG EMEAAQPIGD GQGWPGRFDG PGRFSGHPHP GRGGHHGHHH 160**

**He185/333_cDNA_0096 MELKVTLIVA LVAAITISVH AQREPGGRGN GRERGQGRFR GRPGSDRSQM MGGPRQGGPP MGGRRFDGPG QGDQQMDGRG PNGGPMGGRR FDGPGFGGFR PEGAGRPFFG QGGMHADGEG EMEVAQPIGD GQGWPGRFDG PGRFSGRPYP GRDG------ 154**

**He185/333_cDNA_0097 MELKVTLIVA LVAAITISVH AQREPGGRGN GRERGQGRFR GRPGSDRSQM MGGPRQGGPP MGGRRFDGPG QGDQQMDGRG PNGGPMGGRR FDGPGFGGFR PEGAGRPFFG QGGMHADGEG EMEVAQQIGD GQGWPGRFDG PGRFSGRPYP GRDG------ 154**

**He185/333_cDNA_0098 MELKVTLIVA LVAAITISAH AQRERGGRGN GRERGQGRFG GRPGSDRSQM MGGPRQGGAP MGGRRFDGPG QGDQQMDGRG PNGGPMGGRR FDGPGFGGFR PEGAGRPFFG HGGRHADGEG EMEAAQPIGD GQGWPGRFDG PGRFSGHPHP GRGGHHGHHH 160**

**He185/333_cDNA_0099 MELKVTLIVA LVAAITISVH AQREPGGRGN GRERGQGRFR GRPGSDRSQM MGGPRQGGPP MGGRRFDGPG QGDQQMDGRG PNGGPMGGRR FDGPGFGGFR PEGAGRPFFG QGGMHADGEG EMEVAQPIGD GQGWPGRFDG PGRFSGRPYP GRDG------ 154**

**He185/333_cDNA_0100 MELKVTLIVA LVAAITISVH AQRGRGGRGN GRERGQGRFG GRPGS-RPQM MGGHRQGGPP MGGRRFDGNG QGDQQMAGRE PNGRPVGSRK FDGPGFGGFR PEGAGRPFFG HGGMHADGEG EMEVPQPIGD GQGWPDRFDG PRRFSGRPYP GRGG------ 153**

**He185/333_cDNA_0101 MELKVTLIVA LVAAITISVH AQREPGGRGN GRERGQGRFR GRPGSDRSQM MGGPRQGGPP MGGRRFDGPG QGDQQMDGRG PNGGPMGGRR FDGPGFGGFR PEGAGRPFFG QGGMHADGEG EMEVAQPIGD GQGWPGRFDG PGRFSGRPYP GRDG------ 154**

**He185/333_cDNA_0102 MELKVTLIVA LVAAITISVH AQRGRGGRGN GRERGQGRFG GRPGS-RPQM MGGHRQGGPP MGGRRFDGNG QGDQQMAGRE PNGRPVGSRK FDGPGFGGFR PEGAGRPFFG QGGMHADGEG EMEVAQPIGD GQGWPGRFDG PGRFSGRPYP GRDG------ 153**

**He185/333_cDNA_0103 MELKVTLIVA LVAAITISAH AQRERGGRGN GRERGQGRFG GRPGSDRSQM MGGPRQGGAP MGGRRFDGPG QGDQQMDGRG PNGGPMGGRR FDGPGFGGFR PEGAGRPFFG YGGRHADGER EMEAAQPIGD GQGWPGRFDG PGKFSGHPHP GRGGHHGHHH 160**

**He185/333_cDNA_0104 MELKVTLIVA LVAAITISAH AQRERGGRGN GRERGQGRFG GRPGSDRSQM MGGPRQGGAP MGGRRFDGPG QGDQQMDGRG PNGGPMGGRR FDGPGFGGFR PEGAGRPFFG QGGMHADGEG EMEVAQPIGD GQGWPGRFDG PGRFSGRPYP GRDG------ 154**

**He185/333_cDNA_0105 MELKVTLIVT LVAAITISVH AQREPGGRGN GRERGQGRFG GRPGSDRSQM MGGPRQGGPP MGGRRFDGPG QGDQQMDGRG PNGGPMGGRR FDGPGFGGFR LEGAGRPFFG HGGRHADGEG EMEAAQPI-- ---------- ---------- -------IWS 131**

**He185/333_cDNA_0106 MELKVTLIVT LVAAITISVH AQREPGGRGN GRERGQGRFG GRPGSDRSQM MGGPRQGGPP MGGRRFDGPG QGDQQMDGRG PNGGPMGGRR FDGPGFGGFR LEGAGRPFFG HGGRHADGEG EMEAAQPIGD GQGWPGRFDG PGRFSGRPYP RPGRRTIIWS 160**

**He185/333_cDNA_0107 MELKVTLIVT LVAAITISVH AQREPGGRGN GRERGQGRFG GRPGSDRSQM MGGPRQGGPP MGGRRFDGPG QGDQQMDGRG PNGGPMGGRR FDGPSSP*-- ---------- ---------- ---------- ---------- ---------- ---------- 97**

**He185/333_cDNA_0108 MELKVTLIVT LVAAITISVH AQREPGGRGN GRERGQGRFG GRPGSDRSQM MGGPRQGGPP MGGRRFDGPG QGDQQMDGRG PNGGPMGGRR FDGPGFGGFR LEGAGRPFFG HGGRHADGEG EMEAAQPIGD GQGWPGRFDG PGRFSGRPYP GRGGHHGHHH 160**

**He185/333_cDNA_0109 MELKVTLIVA LVAAITISVH AQRARGGRGY GRKRGQGRFG GSPGSDRPQM TGGPRQGGPP MGGRRFDGPG QGDQQRMADL TVTTTTIITM TVITRQTTTI TIITPKATAT IIITRQKRVT RTDQR*---- ---------- ---------- ---------- 125**

**He185/333_cDNA_0110 MELKVTLIVT LVAAITISVH AQREPGGRGN GRERGQGRFG GRPGSDRSQM MGGPRQGGPP MGGRRFDGPG QGDQQMDGRG PNGGPMGGRR FDGPGFGGFR LEGAGRPFFG HGGRHADGEG EMEAAQPIGD GQGWPGRFDG PGRFSGRPYP GRGGHHGHHH 160**

**He185/333_cDNA_0111 MELKVTLIVT LVAAITISVH AQREPGGRGN GRERGQGRFG GRPGSDRSQM MGGPRQGGPP MGGRR----- ---------- ---------- ---------- ------PFFG HGGRHADGEG EMEAAQPIGD GQGWPGRFDG PGRFSGRPYP GRGGHHGHHH 119**

**He185/333_cDNA_0112 MELKVTLIVT LVAAITISVH AQREPGGRGN GRERGQGRFG GRPGSDRSQM MGGPRQWAAG GLMDQDSVAS DSKVQGDLSS VTEEGMLMEK EKWRLLNQSV MVKDGPVVSM VLEDFPDVLT QAVAVIMDTT MVLTMTRPTN NHLVSKTTAA ARRMADLTVT 160**

16

23

25

26

27

15

19

20

21

22

24

17

18

Repeat 2

Repeat 2

Repeat 2

Repeat 2

Repeat 2

Repeat 3

**....|....| ....|....| ....|....| ....|....| ....|....| ....|....| ....|....| ....|....| ....|....| ....|....| ....|....| ....|....| ....|....| ....|....| ....|....| ....|....|**

**165 175 185 195 205 215 225 235 245 255 265 275 285 295 305 315**

**He185/333_cDNA_0001 GPHHDQADEQ SFGQQNDSSS EEDGRPHRHH HH---HHHHD RHNKTDDHHR HNHTEGHRHH ---------- ---------- ---------- -------HHN KTEEGDQDRP EMRPFRFNPF GRKPFGGRPF GRRNHTEEGS PRRDG----- -NRGRWDGNE 181**

**He185/333_cDNA_0002 GPHHDQADEQ SFGQQNDSSS EEDGRPHRHH HH---HHHHD RHNKTDDHHH HNHTEGHRHH ---------- ---------- ---------- -------HHN KTEEGDQDRP EMRPFRFNPF GRKPFGRRQF GRRNHTEEGS PRRDG----- -NRGRWDENE 274**

**He185/333_cDNA_0003 GPHHDQADEQ SFGQQNDSSS EEDGRPHRHH HH---HHHHD RHNKTDDHHH HNHTEGHRHH ---------- ---------- ---------- -------HHN KTEEGDQDRP EMRPFRFNPF GRKPFGRRQF GRRNHTEEGS PRRDG----- -NRGRWDENE 274**

**He185/333_cDNA_0004 GPHHDQADEQ SFGQQNDSSS EE-GRPHRHH HH---HHHHD RHNKTDDHHH HNHTEGHRHH ---------- ---------- ---------- -------HHN KTEEGDQDRP EMRPFRFNPF GRKPFGGRPF GRRNHTEEGS PRRDG----- -NRGRWDENE 273**

**He185/333_cDNA_0005 GPHHGQADEQ SFGQQNDSSS EEDGRPHRHH HH---HHHHD RHNKTDDHHH HNHTEGHRHH ---------- ---------- ---------- -------HHN KTEEGDQDRP EMRPFRFNPF GRKPFGRRPF GRRNHTEEGS PRRDG----- -NRGRWDENE 274**

**He185/333_cDNA_0006 GPHHDQADEQ SFGQQNDSSS EEDGRPHRHH HH---HHHHD RHNKTGDHHH HNHTEGHRHH ---------- ---------- ---------- -------HHN KTEEGDQDRP EMRPFRFNPF GRKPFGRRQF GRRNHTEEGS PRRDG----- -NRGRWDENE 206**

**He185/333_cDNA_0007 GPHHDQADEQ SFGQQNDSSS EEDGRPHRHH HH---HHHHD RHNKTDDHHH HNHTEGHRHH ---------- ---------- ---------- -------HHN KTEEGDQDRP EMRPFRFNPF GRKPFGRRQF GRRNHTEEGS PRCDG----- -NRGRWDENE 274**

**He185/333_cDNA_0008 GPHHDQADEQ SFGQQNDSSS EEDGRPHRHH HH---HHHHD RHNKTDDHHH HNHTEGHRHH ---------- ---------- ---------- -------HHN KTEEGDQDRP EMRPFRFNPF GRKPFGGRPF GRRNHTEEGS PRRDG----- -NRGRWDENE 274**

**He185/333_cDNA_0009 GPHHDQADEQ SFGQQNDSSS EEDGRPHRHH HH---HHHHD RHNKTDDHHH HNHTEGHRHH ---------- ---------- ---------- -------HHN KTEEGDQDRP EMRPFRFNPF GRKPFGRRPF GRRDHTEEGS PRRDG----- -NRGRWDENE 274**

**He185/333_cDNA_0010 GPHHDQADEQ SFGQQNDSSS EEDGRPHRHH HH---HHHHD RHNKTDDHHH HNRTEGHRHH ---------- ---------- ---------- -------HHN KTGEGDQDRP EMRPFRFDPF GRKPFGRRPF GRRNHTEEGS PRRDG----- -NRGRWDENE 274**

**He185/333_cDNA_0011 GPHHGQADEQ SFGQQNDSSS EEDGRPHRHH HH---HHHHD RHNKTDDHHH HNHTEGHRHH ---------- ---------- ---------- -------HHN KTEEGDQDRP EMRPFRFNPF GRKPFGRRQF GRRNHTEEGS PRRDG----- -NRGRWDENE 274**

**He185/333_cDNA_0012 ---HDKADEQ SFGQQNDSSS EEDGRPHRHH HH---HHHHD RHNKTDDHHH HNHTEGHRHH ---------- ---------- ---------- -------HHN KTEEGDQDRP EMGPFRFNPF GRKPFGGRPF GRCNHTEEGS PRRDGDRRPN GNRGRWDENE 271**

**He185/333_cDNA_0013 GPHHDQADEQ PFGQQNDSSS EEDGRPHRHH HH---HHHHD RHNKTDDHHH HNHTEGHRHH ---------- ---------- ---------- -------HHN KTEEGDQDRP EMRPFRFNPF GRKPFGGRPF GRRNHTEEGS PRRDG----- -NRGRWDENE 274**

**He185/333_cDNA_0014 ---HDKADEQ SFGQQNDSSS EEDGRPHRHH HH---HHHHD RHNKTDDHHH HNHTEGHRHH ---------- ---------- ---------- -------HHN KTEEGDQDRP EMRPFRFNPF GRKPFGGRPF GRCNHTEEGS PRRDGDRRPK GNRGRWDENE 271**

**He185/333_cDNA_0015 ---HDKADEQ SFGQQNDSSS EEDGRPHRHH HH---HHHHD RHNKTDDHHH HNHTESHRHH ---------- ---------- ---------- -------HHN KTEEGDQDRP EMRPFRFNPF GRKPFGGRPF GGCNHTEEGS PRRDGDRRPN GNRGRWDENE 271**

**He185/333_cDNA_0016 ---HDKADEQ SFGQQNDGSC DEDGRPHRPH PP---HHHHD RHNNTDDHHH HNHPEGRRPH ---------- ---------- ---------- -------HHY NTEEGGQDRP EMRPFRFFHL FRKQFGGRPF GRCSHHEEGC STRDGDRRPN G-----DENE 266**

**He185/333_cDNA_0017 GPHHDQADEQ SFGQQNDSSS EEDGRPHRHH HHHH-HHHHD RHNKTGDHHH HNHTEGHRHH HN-------- ---------- ---------- ---------- ---------- ---------- --KPFGGRPF GRRNHTDEGS PRRDG----- -HRGRWNENE 253**

**He185/333_cDNA_0018 GPHHDQADEQ SFGQ*----- ---------- ---------- ---------- ---------- ---------- ---------- ---------- ---------- ---------- ---------- ---------- ---------- ---------- ---------- 174**

**He185/333_cDNA_0019 GPHHDQADEQ SFGQQNDSSS EEDGRPHRHH HH---HHHHD RHNKTDDHHH HNHTEGHRHH ---------- ---------- ---------- -------HHN KTEEGDQDRP EMRPFRFNPF GRKPFGRRQF GRRNHTEEGS PRRDG----- -NRGRWDENE 274**

**He185/333_cDNA_0020 GPHHDQADEQ SFGQQNDSSS EEDGRPHRHH HH---HHHHD RHNKTDDHHH HNHTEGHRHH ---------- ---------- ---------- -------HHN KTEEGDQDRP EMRPFRFNPF GRKPFGGRPF GRRNHTEEGS PRRDG----- -NRGRWDENE 260**

**He185/333_cDNA_0021 GPHHDQADEQ SFGQQNDSSS EEDGRPHRHH HH---HHHHD RHNKTDDHHH HNHTEGHRHH ---------- ---------- ---------- -------HHN KTEEGDQDRP EMRPFRFNPF GRKPFGRRPF GRRNHTEEGS PRRDG----- -NRGRWDENE 274**

**He185/333_cDNA_0022 ---------- ---------- ---------- ---------- ---------- ---------- ---------- ---------- ---------- ---------- ---------- ---------- ---------- ---------- ---------- ---GRWDENE 41**

**He185/333_cDNA_0023 ---HDKADEQ SFGQQNDSSS EEDGRPHHHH HH---HHHHD RHNKTDDHHH HNHTEGHRHQ ---------- ---------- ---------- -------HHN KTDEGDQDSP EMRPFRFNAF GRKPFGGHPF GRRNHTEEGS PRRDGDRRPN GNRGRWDENE 270**

**He185/333_cDNA_0024 ---HDKADEQ SFGQQNDSSS EEDGRPHRHH RHH------- ---------- ---------- ---------- ---------- ---------- -------HHN KTEEGDQDRP EMRPFRFNPF GRKPFGGRPF GRCNHTEEGS PRRDGDRRPK GNRGRWDENE 247**

**He185/333_cDNA_0025 ---HDKADEQ SFGQQNDSSS EEDGRPHHHH HH---HHHHD RHNKTDDHHH HNHTEGHRHQ ---------- ---------- ---------- -------HHN KTEEGDQDRP EMRPFRFNAF GRKPFGGHPF GRRNHTEEGS PRRDGDRRPN GNRGRWDENE 270**

**He185/333_cDNA_0026 ---HDKADEQ SFGQQNDSSS EEDGRPHRHH HH---HHHHD RHNKTDDHHH HNHTEGHRHH ---------- ---------- ---------- -------HHN KTEEGDQDRP EMRPFRFNPF GRKPFGGRPF GRCNHTEEGS PRRDGDRRPK GNRGRWDENE 271**

**He185/333_cDNA_0027 ---HDKADEQ SFGQQNDSSS EEDGRPHHHH HH---HHHHD RHNKTDDHHH HNHTEGHRHQ ---------- ---------- ---------- -------HHN KTEEGDQDRP EMRPFRFNAF GRKPFGGHPF GRRNHTEEGS PRRDGDRRPN GNRGRWDENE 270**

**He185/333_cDNA_0028 ---HDKADEQ SFGQQNDSSS EEDGRPHRHH HH---HHHHD RHNKTDDRHH HNHTEGHRHH ---------- ---------- ---------- -------HHN KTEEGDQDRP EMRPFRFNPF GRKPFGGRPF GRCNHTEEGS PRRDGDRRPK GNRGRWDENE 236**

**He185/333_cDNA_0029 ---HDKADAQ SFGQQNDSSS EEDGRPHHHH HH---HHHHD RHNKTDDHHH HNHTEGHRHQ ---------- ---------- ---------- -------HHN KTEEGDQDRP EMRPFRFNAF GRKPFGGHPF GRRNHTEEGS PRRDGDRRPN GNRGRWDENE 270**

**He185/333_cDNA_0030 ---HDKADEQ SFGQQNDSSS EEDGRPHRHH HH---HHHHD RHNKTDDHHH HNHTEGHRHH ---------- ---------- ---------- -------HHN KTEEGDQDRP EMRPFRFNPF GRKPFGGRPF GRCNHTEEGS PRRDGDRRPK GNRGRWDENE 271**

**He185/333_cDNA_0031 ---HDKADEQ SFGQQNDSSS EEDGRPRHHH HH---HHHHD RHNKTDDHHH HNHTEGHRHQ ---------- ---------- ---------- -------HHN KTEEGDQDRP EMRPFRFNAF GRKPFGGHPF GRRNHTEEGS PRRDGDRRPN GNRGRWDENE 270**

**He185/333_cDNA_0032 ---HDKADEQ SFGQQNDSSS EEDGRPHRHH HH---HHHHD RHNKTDDHHH HNHTEGHRHH ---------- ---------- ---------- -------HHN KTEEGDQDRP EMRPFRFNPF GRKPFGGRPF GRCNHTEEGS PRRDGDRRPK GNRGRWDENE 236**

**He185/333_cDNA_0033 ---HDKADEQ SFGQQNDSSS EEDGRPHHHH H----HHHHD RHNKTDDHHH HNHTEGHRHQ ---------- ---------- ---------- -------HHN KTEEGDQDRP EMRPFRFNAF GRKPFGGHPF GRRNHTEEGS PRRDGDRRPN GNRGRWDENE 269**

**He185/333_cDNA_0034 ---HDKADEQ SFGQQNDSSS EEDGRPHHHH HH---HHHHD RHNKTDDHHH HNHTEGHRHQ ---------- ---------- ---------- -------HHN KTEEGDQDRP EMRPFRFNAF GRKPFGGHPF GRRNHTEEGS PRRDGDRRPN GNRGRWDENE 270**

**He185/333_cDNA_0035 GPHHDQADEQ SFGQQNDSSS EEDGRPHRHH HHHHHHHHHD RHNKTDDHRH HNHTEGHRHH ---------- ---------- ---------- -------HHN KTEEGDQDRP EMRPFRFNLF GRKPFGERPF GRRNHTEEGS PRRDE----- -HRGRWDENE 277**

**He185/333_cDNA_0036 GPHHDQADEQ SFGQQNDSSS EEDGRPHRHH HHHH-HHHHD RHNKTGDHHH HNHTEGHRHH HNKTDDHHHH NHTEGHRHHH NKTDDHHHHN HTEGHRHHHN KTEEGDQDRP EMRPFWFNPF GRKPFGGRPF GRRNHTDEGS PRRDG----- -HRGRWNENE 313**

**He185/333_cDNA_0037 ---HDKADEQ SFGQQNDSSS EEDGRPHRHH HHH--HHHHD RHNKTDDHHH HNHTEGHRHH ---------- ---------- ---------- -------HHN KTEEGDQDRP EMRPFRFNPF GRKPFGGRPF GRCNHTEEGP PRRDGDRRPK DNRGRWDENE 272**

**He185/333_cDNA_0038 GPHHDQADEQ SFGQQNDSSS EEDGRPHRHH HH---HHHHD RHNKTDDHHH HNHTEGHRHH ---------- ---------- ---------- -------HHN KTEEGDQDRP EMRPFRFNSF GHKPFGRRPF GRRNHTEEGS PRRDG----- -NRGRWDENE 274**

**He185/333_cDNA_0039 GPHRDQADEQ SFGQQNDSSS EEDGRPHRHH HH---HHHHD RHNKTDDHHH HNHTEGHRHH ---------- ---------- ---------- -------HHN KTEEGDQDRP EMRPFRFNPF GRKPFGRRQF GRRNHTEEGS PRRDG----- -NRGRWDENE 274**

**He185/333_cDNA_0040 GPHHDQADEQ SFGQQNDSSS EEDGRPHHHH HHHHHHHHHD RHNKTDDHHH HNHTEGHRHH ---------- ---------- ---------- -------HHN KTEEGDQDRP EMRPFRFNPF GRKPFGGRPF GRRNHTEEGS PRRDG----- -NRGRWDENE 277**

**He185/333_cDNA_0041 GPHRDQADEQ SFGQQNDSSS EEDGRPHRHH HH---HHHHD RHNKTDDHHH HNHTEGHRHH ---------- ---------- ---------- -------HHN KTEEGDQDRP EMRPFRFNPF GRKPFGRRPF GRRNHTEEGS PRRDG----- -NRGRWDENE 274**

**He185/333_cDNA_0042 GPHHDQADEQ SFGQQNDSSS EEDGRPHRHH HH---HHHHD RHNKTDNHHH HNHTEGHRHH ---------- ---------- ---------- -------HHN KTEEGDQDRP EMRPFRFNPF GRKPFGRRQF GRRNHTEEGS PRRDG----- -NRGRWDENE 274**

**He185/333_cDNA_0043 ---HDKADEQ SFGQQNDSSS EEDGRPHHHH HH---HHHHD RHNKTDDHHH HNHTEGHRHQ ---------- ---------- ---------- -------HHN KTEEGDQDRP EMRPFRFNAF GRKPFGGHPF GRRNHTEEGS PRRDGDRRPN GNRGRWDENE 270**

**He185/333_cDNA_0044 GPHHDQADGQ SFGQQNDSSS EEDGRPHRHH HH---HHHHD RHNKTDDHHH HNHTEGHRHH ---------- ---------- ---------- -------HHN KTEEGDQDRP EMRPFRFNPF GRKPFGGRPF GRRNHTEEGS PRRDG----- -NRGRWDENE 274**

**He185/333_cDNA_0045 ---------- ---------- ---------- ---------- ---------- ---------- ---------- ---------- ---------- ---------- ---------- ---------- ---------- ---------- ---------- ---------- 52**

**He185/333_cDNA_0046 GPHHDQADEQ SFGQQNDSSS EEDGRPHHHH HHHHHHHHHD RHNKTDDHHH HNHTEGHRHH ---------- ---------- ---------- -------HHN KTEEGDQDRP EMRPFRFNPF GRKPFGGRPF GRRNHTEEGS PRRDG----- -NRGRWVENE 277**

**He185/333_cDNA_0047 ---HDKADEQ SFGQQNDSSS EEDGRPHRHH HH---HHHHD RHNKTDDHRH HNRTEGHRHH ---------- ---------- ---------- -------HHN KTEEGDQDRP EMRPFRFNPF GRKPFGGRPF GRCNHTEEGS PRRDGDRRPK GNRGRWDENE 271**

**He185/333_cDNA_0048 GPHHDQADEQ SFGQQNDSSS EEDDRPHRHH HH---HHHHD RHNKTDDHHH HNHTEGHRHH ---------- ---------- ---------- -------HHN KTEEGDQDRP EMRPFRFNPF GRKPFGGRPF GRRNHTEEGS PRRDG----- -NRGRWDENE 274**

**He185/333_cDNA_0049 GPHHDQADEQ SFGQQNDSSS EEDGRPHRHH HH---HHHHD RHNKTDDHHH HNHTEGHRHH ---------- ---------- ---------- -------HHN KTEEGDQDRP EMRPFRFNPF GRKPFGGRPF GRRNHTEEGS PRRDG----- -NRGRWDENE 274**

**He185/333_cDNA_0050 GPHHDQADEQ SFGQQNDSSS EEDGRPHRHH HH---HHHHD RHNKTDDHHH HNHTEGHRHH ---------- ---------- ---------- -------HHN KTEEGDQDRP EMRPFRFNPF GRKPFGGRPF GRRNHTEEGS PRRDG----- -NRGRWDENE 274**

**He185/333_cDNA_0051 GPHHDQADEQ SLGQQNDSSS EEDGRPHHHH HHHHHHHHHD RHNKTDDHHH HNHTEGHRHH ---------- ---------- ---------- -------HHN KTEEGDQDRP EMRPFRFNPF GRKPFGGRPF GRRNHTEEGS PRRDG----- -NRGRWDENE 277**

**He185/333_cDNA_0052 ---HDQADEQ SFGQQNDSSS EEDGRPHRHH HH---HHHHD RHNKTDDHHH HNHTEGHRHH ---------- ---------- ---------- -------HHN KTEEGDQDRP EMRPFRFNPF GRKPFGGRPF GRRNHTEEGS PRRDG----- -NRGRWDENE 235**

**He185/333_cDNA_0053 GPHHDQADEQ SFGQQNDSSS EEDGRPHHHH HHHHHHHHHD RHNKTDDHHH H--------- ---------- ---------- ---------- -------HHN KTEEGDQDRP EMRPFRFNPF GRKPFGGRPF GRRNHTEEGS PRRDG----- -NRGRWDENE 268**

**He185/333_cDNA_0054 ---HDKADEQ SFGQQNDSSS EEDGRPHHHH HH---HHHHD RHNKTDDHHH HNHTEGHRHQ ---------- ---------- ---------- -------HHN KTEEGDQDRP EMRPFRFNAF GRKPFGGHPF GRRNHTEEGS PRRDGDRRPN GNRGRWDENE 270**

**He185/333_cDNA_0055 ---HDKADEQ SFGQQNDSSS EEDGRPHRHH HH---HHHHD RHNKTDDHHH HNHTEGHRHH ---------- ---------- ---------- -------HHN KTEEGDQDRP EMRPFRFNPF GRKPFGGRPF GRCNHTEEGS PRRDGDRRPK GNRGRWDENE 271**

**He185/333_cDNA_0056 ---HDKADEQ SFGQQNDSSS EEDGRPHRHH HH---HHHHD RHNKTDDHHH HNHTEGHRHH ---------- ---------- ---------- -------HHN KTEEGDQDRP EMRPFRFNPF GRKPFGGRPF GRCNHTEEGS PRRDGDGRPK GNRGRWDENE 271**

**He185/333_cDNA_0057 GPHHDQADEQ SFGQQNDSSS EEDGRPHRHH HHHH-HHHHD RHNKTGDHHH HNHTEGHRHH HNKTDDHHHH NHTEGHRHHH NKTDDHHHHN HTEGHRHHHN KTEEGDQDRP EMRPFWFNPF GRKPFGGRPF GRRNHTDEGS PRRDG----- -HRGRWNENE 313**

**He185/333_cDNA_0058 ---------- ---------- ---------- ---------- ---------- ---------- ---------- ---------- ---------- ---------- ---------- ---------- ---------- -------EGS PRRDG----- -HLGRWNENE 118**

**He185/333_cDNA_0059 GPHHDQADEQ SFGQQNDSSS EEDGRPHRHH HHHH-HHHHD RHNKTGDHHH HNHTEGHRH- ---------- ---------- ---------- -------HHN KTEEGDQDRP EMRPFWFNPF GRKPFGGRPF GRRNHTDEGS PRRDG----- -HRGRWNENE 275**

**He185/333_cDNA_0060 GPHHDQADEQ SFGQQNDSSS EEDGRPHRHH HHHHHHHHHD RHNKTDDHRH HNHTEGHRHH ---------- ---------- ---------- -------HHN KTEEGDQDRP EMRPFRFNLF GRKPFGERPF GRRNHTEEGS PRRDE----- -HRGRWDENE 277**

**He185/333_cDNA_0061 GPHHDQADEQ SFGQQNDSSS EEDGRPHRHH HHHH-HHHHD RHNKTDDHHH HNHTESHRHH HNKTDDHHHH NHTESHRHHH NKTDDHHHHN HTEGHRHHHN KTEEGDQDRP EMRPFWFNPF GRKPFGGRPF GRRNHTDEGS PRRDG----- -HRGRWNENE 313**

**He185/333_cDNA_0062 ----DQADEQ SFGQQNDSSS EEDGRPHRHH HHHH-HHHHD RHNKTGDHHH HNHTEGHRHH HNKTDDHHHH NHTEGHRHHH NKTDDHHHHN HTEGHRHHHN KTEEGDQDRP EMRPFWFNPF GRKPFGGRPF GRRNHTDEGS PRRDG----- -HRGRWNENE 221**

**He185/333_cDNA_0063 GPHHDQAGEQ SFGQQNDSSS EEDGRPHRHH HHHH-HHHHD RHNKTGDHHH HNHTEGHRHH HNKTDDHHHH NHTEGHRHHH NKTDDHHHHN HTEGHRHHHN KTEEGDQDRP EMRPFWFNPF GRKPFGGRPF GRRNHTDEGS PRRDG----- -HRGRWNENE 313**

16

18

20

17

19

**....|....| ....|....| ....|....| ....|....| ....|....| ....|....| ....|....| ....|....| ....|....| ....|....| ....|....| ....|....| ....|....| ....|....| ....|....| ....|....|**

23

27

15

21

22

14

25

26

Repeat 2

Repeat 2

Repeat 2

Repeat 2

Repeat 3

Repeat 2

**165 175 185 195 205 215 225 235 245 255 265 275 285 295 305 315 He185/333_cDNA_0064 GPHHDQADEQ SFGQQNDSSS EEDGRPHRHH HHHH-HHHHD RHNKTGDHHH HNHTEGHRHH HNKTDDHHHH NHTEGHRHHH NKTDDHHHHN HTEGHRHHHN KTEEGDQDRP EMRPFWFNPF GRKPFGGRPF GRRNHTDEGS PRRDG----- -HRGRWNENE 313**

**He185/333_cDNA_0065 GPHHDQADEQ SFGQQNDSSS EEDGRPHRHH HHHHHHHHHD RHNKTGDHHH HNHTEGHCHH HNKTDDHHHH NHTEGHRHHH NKTDDHHHHN HTEGHRHHHN KTEEGDQDRP EMRPFWFNPF GRKPFGGRPF GRRNHTDEGS PRRDG----- -HRGRWNENE 289**

**He185/333_cDNA_0066 GPHHDQADEQ SFGQQNDSSS EEDGRPHRHH HHHH-HHHHD RHNKTGDHHH HNHTEGHRHH HNKTDDHHHH NHTEGHRHHH NKTDDHHHHN HTEGHRHHHN KTEEGDQDRP EMRPFWFNPF GRKPFGGRPF GRRNHTDEGS PRRDG----- -HRGRWNENE 290**

**He185/333_cDNA_0067 GPHHDQADEQ SFGQQNDSSS EEDGRPHRHH HHHH-HHHHD RHNKTGDHHH HNHTEGHRH- ---------- ---------- ---------- -------HHN KTEEGDQDRP EMRPFWFNPF GRKPFGGRPF GRRNHTDEGS PRRDG----- -HRGRWNENE 250**

**He185/333_cDNA_0068 GPHHDQADEQ SFGQQNDSSS EEDGRPHHHH HHHHHHHHHD RHNKTDDHHH HNHTEGHRHH ---------- ---------- ---------- -------HHN KTEEGDQDRP EMRPFRFNPF GRKPFGGRPF GRRNHTEEGS PRCDG----- -NRGRWDENE 277**

**He185/333_cDNA_0069 ---------- ---------- ---------- ---------- ---------- ---------- ---------- ---------- ---------- ---------- ---------- ---------- ---------- ---------- ---------- ---------- 43**

**He185/333_cDNA_0070 GPHHDQADEQ SFGQQNDSSS EEDGRPHRHH HH---HHHHD RHNKTDDHHH HNHTEGHRHH ---------- ---------- ---------- -------HHN KTEEGDQDRP EMRPFRFNPF GRKPFGGRPF GRRNHTEEGS PRRDG----- -NRGRWDENE 274**

**He185/333_cDNA_0071 GPHHDQADEQ SFGQQSDSSS EEDGRPHRHH HH---HHHHD RHNKTDDHHH HNHTEGHRHH ---------- ---------- ---------- -------HHN KTEEGDQDRP EMRPFRFNPF GRKPFGGRPF GRRNHTEEGS PRRDG----- -NRGRWDENE 274**

**He185/333_cDNA_0072 GPHHDQADEQ SFGQQNDSSS EEDGRPHRHH HH---HHHHD RHNKTDDHHH HNHTEGHRHH ---------- ---------- ---------- -------HHN KTEEGDQDRP EMRPFRFNPF GRKPFGGRPF GRRNHTEEGS PRRDG----- -NRGRWDENE 274**

**He185/333_cDNA_0073 GPHHDQADEQ SFGQQNDSSS EEGGRPHRHH HH---HHHHD RHNKTDDHHH HNHTEGHRHH ---------- ---------- ---------- -------HHN KTEEGDQDRP EMGPFRFNPF GRKPFGGRPF GRRNHTEEGS PRRDG----- -NRGRWDENE 274**

**He185/333_cDNA_0074 GPHHDQADEQ SFGQQNDSSS EEDGRPHRHH HH---HHHYD RHNKTDDHRH HNHTEGHRHH ---------- ---------- ---------- -------HHN KTEEGDQDRP EMRPFRFNPF GRKPFGGRPF GRRNHTEEGS PRRDG----- -NRGRWDENE 274**

**He185/333_cDNA_0075 GPHHDQADEQ SFGQQNDSSS EEDGRPHRHH HRHH-HHHHD RHNKTGDHHH HNHTEGHRHH HNKTDDHHHH NHTEGHRHHH NKTDDHHHHN HTEGHRHHHS KTEEGDQDRP EMRPFWFNPF GRKPFGGRPF GRRNHTDEGS PRRDG----- -HRGRWNENE 313**

**He185/333_cDNA_0076 GPHHDQADEQ SFGQQNDSSS EEDGRPHRHH HH---HHHHD RHNKTDDHHH HNHTEGHRHH ---------- ---------- ---------- -------HHN KTEEGDQDRP EMRPFRFNPF GRKPFGRRQF GRRNHTKEGS PRRDG----- -NRGRWDENE 274**

**He185/333_cDNA_0077 ---HDKADEQ SFGQKNDSSS EEDGRPHRHH HH---HHHHD RHNKTDDHHH HNHTEGHRHH ---------- ---------- ---------- -------HHN KTEEGDQDRP EMRPFRFNPF GRKPFGGRPF GRCNHTEEGS PRRDGDRRPK GNRGRWDENE 271**

**He185/333_cDNA_0078 GPHHDQADEQ SFGQQNDSSS EEDGRPHRHH HH---HHHHD RHNKTDDHHH HNHTEGHRHH ---------- ---------- ---------- -------HHN KTEEGDQDRP EMRPFRFNPF GRKPFGGRPF GRRNHTEEGS PRRDG----- -NRGRWDENE 274**

**He185/333_cDNA_0079 GPRHDQADEQ SFGQQNDSSS EEDGRPHRHH HH---HHHHD RHNKTDDHHH HNHTEGHRHH ---------- ---------- ---------- -------HHN KTEEGDQDRP EMRPFRFNPF GRKPFGGRPF GRRNHTEEGS PRRDG----- -NRGRWDENE 274**

**He185/333_cDNA_0080 GPHHDQADEQ SFGQQNDSSS EEDGRPHRHH HH---HHHHD RHNRTDDHHH HNHTEGHRHH ---------- ---------- ---------- -------HHN KTEEGDQDRP EMRPFRFNPF GRKPFGRRQF GRRNHTEEGS PRRDG----- -NRGRWDENE 274**

**He185/333_cDNA_0081 ---HDKADEQ SFGQQNDSSS EEDGRPHHHH HH---HHHHD RHNKTDDHHH HNHTEGHRHQ ---------- ---------- ---------- -------HHN KTEEGDQDRP EMRPFRFNPF GRKPFGGHPF GRRNHTEEGS PRRDGDRRPN GNRGRWDENE 270**

**He185/333_cDNA_0082 GPHHDKADEQ SFGQQNDSSS EEDGRPHRHH HH---HHHHD RHNKTNDHHH HNHTEGHRHH ---------- ---------- ---------- -------HHN KTEEGDQDRP EMRPFRFNPF GRKPFGGRPF GRCNHTEEGS PRRDGDRRPN GNRGRWDENE 280**

**He185/333_cDNA_0083 ---HDKADEQ SFGQQNDSSS EEDGRPHRHH HH---HHHHD RHNKTNDHHH HNHTEGHRHH ---------- ---------- ---------- -------HHN KTEEGDQDRP EMRPFRFNPF GRKPFGGRPF GRCNHTEEGS PRRDGDRRPN GNRGRWDENE 270**

**He185/333_cDNA_0084 ---HDKADEQ SFGQQNDSSS EEDGRPHRHH HH---HHHHD RHNKTNDHHH HNHTEGHRHH ---------- ---------- ---------- -------HHN KTEEGDQDRP EMRPFRFNPF GRKPFGGRPF GRCNHTEEGS PRRDGDRRPN GNRGRWDENE 271**

**He185/333_cDNA_0085 GPHHDKADEQ SFGQQNDSSS EEDGRPHHHH HHHHHHHHHD RHNKTDDHHH HNHTEGHRHH ---------- ---------- ---------- -------HHN KTEEGDQDRP EMRPFRFNPF GRKPFGGRPF GRRNHTEEGS PRRDGDRRPN GNRGRWDENE 283**

**He185/333_cDNA_0086 ---HDKADEQ SFGQQNDSSS EEDGRPHRHH HH---HHHHD RHNKTNDHHH HNHTEGHRHH ---------- ---------- ---------- -------HHN KTEEGDQDRP EMRPFRFNPF GRKPFGGRPF GRCNHTEEGS PRRDGDRRPN GNRGRWDENE 271**

**He185/333_cDNA_0087 ---HDKADEQ SFGQQNDSSS EEDGRPHRHH HH---HHHHD RHNKTNDHHH HNHTEGHRHH ---------- ---------- ---------- -------HHN KTEEGDQDRP EMRPFRFNPF GRKPFGGHPF GRRNHTEEGS PRRDGDRRPN GNRGRWDENE 271**

**He185/333_cDNA_0088 GPHHDQADEQ SFGQQNDSSS EEDGRPHRHH HHHH-HQHHD RHNKTGDHHH HNHTEGHRHH HNKTDDHHHH NHTEGHRHHH NKTDDHHHHN HTEGHRHHHN KTEEGDQDRP EMRPFWFNPF GRKPFGGRPF GRRNHTDEGS PRRDG----- -HRGRWNENE 313**

**He185/333_cDNA_0089 GPHHDQADEQ SFGQQNDSSS EEDGRPHRHH HHHH-HHHHD RHNKTGDHHH HNHTEGHRHH HDKTDDHHHH NHTEGHRHHH NKTDDHHHHN HTEGHRHHHN KTEEGDQDRP EMRPFWFNPF GRKPFGGRPF GRRNHTDEGS PRRDG----- -HRGRWNENE 313**

**He185/333_cDNA_0090 ---HDKADEQ SFGQQNDSIS EEDGRPHRHH HH---HHHHD RHNKTNDHHH HNHTEGHRHH ---------- ---------- ---------- -------HHN KTEEGDQDRP EMRPFRFNPF GRKPFGGRPF GRCNHTEEGS PRRDGDRRPN GNRGRWDENE 271**

**He185/333_cDNA_0091 ---HDKADEQ SFGQQNDSSS EEDGRPHRHH HH---HHHHD RHNKTNDHHH HNHTEGHRHH ---------- ---------- ---------- -------HHN KTEEGDQDRP EMRPFRFNPF GRKPFGGRPF GRCNHTEEGS PRRDGDRRPN GNRGRWDENE 271**

**He185/333_cDNA_0092 GPHHDKADEQ SFGQQNDSSS EEDGRPHHHH HHHHHHHHHD RHNKTDDHHH HNHTEGHRHH ---------- ---------- ---------- -------HHN KTEEGDQDRP EMRPFRFNPF GRKPFGGRPF GRRNHTEEGS PRRDGDRRPN GNRGRWDENE 283**

**He185/333_cDNA_0093 --------EQ SFGQQNDSSS EEDGRPHHHH HHHHHHHHHD RHNKTDDHHH HNHTEGHRHH ---------- ---------- ---------- -------HHN KTEEGDQDRP EMRPFRFNPF GRKPFGGRPF GRRNHTEEGS PRRDGDRRPN GNRGRWDENE 192**

**He185/333_cDNA_0094 GPHHDQADEQ SFGQQNDSSS EEDGRPHRHH HHHH-HHHHD RHNKTGDHHH HNHTEGHRHH HDKTDDHHHH NHTEGHRHHH NKTDDHHHHN HTEGHRHHHN KTEEGDQDRP EMRPFWFNPF GRKPFGGRPF GRRNHTDEGS PRRDG----- -HRGRWNENE 313**

**He185/333_cDNA_0095 GPHHDQADEQ SFGQQNDSSS EEDGRPHRHH HHHH-HHHHD RHNKTGDHHH HNHTEGHRHH HDKTDDHHHH NHTEGHRHHH NKTDDHHHHN HTEGHRHHHN KTEEGDQDRP EMRPFWFNPF GRKPFGGRPF GRRNHTDEGS PRRDG----- -HRGRWNENE 313**

**He185/333_cDNA_0096 ---HDKADEQ SFGQQNDSSS EEDGRPHRHH HH---HHHHD RHNKTNDHHH HNHTEGHRHH ---------- ---------- ---------- -------HHN KTEEGDQDRP EMRPFRFNPF GRKPFGGRPF GRRNHTEEGS PRRDGDRRPN GNRGRWDENE 271**

**He185/333_cDNA_0097 ---HDKADEQ SFGQQNDSSS EEDGRPHRHH HH---HHHHD RHNKTNDHHH HNHTEGHRHH ---------- ---------- ---------- -------HHN KTEEGDQDRP EMRPFRFNPF GRKPFGGRPF GRCNHTEEGS PRRDGDRRPN GNRGRWDENE 271**

**He185/333_cDNA_0098 GPHHDQADEQ SFGQQNDSSS EEDGRPHRHH HHHH-HQHHD RHNKTGDHHH HNHTEGHRHH HNKTDDHHHH NHTEGHRHHH NKTDDHHHHN HTEGHRHHHN KTEEGDQDRP EMRPFWFNPF GRKPFGGRPF GRRNHTDEGS PRRDG----- -HRGRWNENE 313**

**He185/333_cDNA_0099 ---HDKADEQ SFGQQNDSSS EEDGRPHRHH HH---HHHHD RHNKTNDHHH HNHTEGHRHH ---------- ---------- ---------- -------HHN KTEEGDQDRP EMRPFRFNPF GRKPFGGRPF GRCNHTEEGS PRRDGDRRPN GNRGRWDENE 271**

**He185/333_cDNA_0100 ---HDKADEQ SFGQQNDSSS EEDGRPHHHH HH---HHHHD RHNKTDDHHH HNHTEGHRHQ ---------- ---------- ---------- -------HHN KTEEGDQDRP EMRPFRFNPF GRKPFGGHPF GRRNHTEEGS PRRDGDRRPN GNRGRWDENE 270**

**He185/333_cDNA_0101 ---HDKADEQ SFGQQNDSSS EEDGRPHRHH HH---HHHHD RHNKTNDHHH HNHTEGHRHH ---------- ---------- ---------- -------HHN KTEEGDQDRP EMRPFRFNPF GRKPFGGRPF GRCNHTEEGS PRRDGDRRPN GNRGRWDENE 271**

**He185/333_cDNA_0102 ---HDKADEQ SFGQQNDSSS EEDGRPHRHH HH---HHHHD RHNKTNDHHH HNHTEGHRHH ---------- ---------- ---------- -------HHN KTEEGDQDRP EMRPFRFNPF GRKPFGGRPF GRCNHTEEGS PRRDGDRRPN GNRGRWDENE 270**

**He185/333_cDNA_0103 GPHHDQADEQ SFGQQNDSSS EEDGRPHRHH HHHH-HQHHD RHNKTGDHHH HNLTEGHRHH HNKTDDHHHH NHTEGHRHHH NKTDDHHHHN HTESHRHHHN KTEEGDQDRP EMRPFWFNPF GRKPFGGRPF GRRNHTDEGS PRRDG----- -HRGRWNENE 313**

**He185/333_cDNA_0104 ---HDKADEQ SFGQQNDSSS EEDGRPHRHH HH---HHHHD RHNKTNDHHH HNHTEGHRHH ---------- ---------- ---------- -------HHN KTEEGDQDRP EMRPFRFNPF GRKPFGGRPF GRCNHTEEGS PRRDGDRRPN GNRGRWDENE 271**

**He185/333_cDNA_0105 AKRQQQRGGW PTSPSPPPPS SP*------- ---------- ---------- ---------- ---------- ---------- ---------- ---------- ---------- ---------- ---------- ---------- ---------- ---------- 153**

**He185/333_cDNA_0106 AKRQQQRGGW PTSPSPPPPS SL*------- ---------- ---------- ---------- ---------- ---------- ---------- ---------- ---------- ---------- ---------- ---------- ---------- ---------- 182**

**He185/333_cDNA_0107 ---------- ---------- ---------- ---------- ---------- ---------- ---------- ---------- ---------- ---------- ---------- ---------- ---------- ---------- ---------- ---------- 97**

**He185/333_cDNA_0108 GPHHDQADEQ SFGQQNDSSS EEDGRPHRHH HHHHHH---D RHNKTDDHHH HNHTEGHRHH ---------- ---------- ---------- -------HHN KTEEGDRTDQ R*-------- ---------- ---------- ---------- ---------- 231**

**He185/333_cDNA_0109 ---------- ---------- ---------- ---------- ---------- ---------- ---------- ---------- ---------- ---------- ---------- ---------- ---------- ---------- ---------- ---------- 125**

**He185/333_cDNA_0110 GPHHDQADEQ SFGQQNDSSS EEDGRPHRHH HHHHHH---D RHNKTDDHHH HNHTEGHRHH ---------- ---------- ---------- -------HHN KTEEGDRTDQ R*-------- ---------- ---------- ---------- ---------- 231**

**He185/333_cDNA_0111 GPHHDQADEQ SFGQQNDSSS EEDGRPHRHH HHHHLH---D RHNKTDDHHH HNHTEGHRHH ---------- ---------- ---------- -------HHN KTEEGDQDRP EMRPFRFNPF RSQTFRRTSI RQTQPYRRRI SQARWQPWTL G*-------- 230**

**He185/333_cDNA_0112 TTTIITMTVI TRQTTTITII TPKATATIII TRQKRVT--- ---------- ---------- ---------- ---------- ---------- ---------- ------RTDQ R*-------- ---------- ---------- ---------- ---------- 202**

28

29

30

31

Repeat 4

**....|....| ....|....| ....|....| ....|....| ..**

**325 335 345 355**

**He185/333_cDNA_0001 -----SVEEE HLPTESMTTS AVPDVVEIDI NEIDSNIIPE V* 217**

**He185/333_cDNA_0002 -----SVEEE HLPTESMTTS VVPDVVEIDI NEIDSNIIPE V* 310**

**He185/333_cDNA_0003 -----SVEEE HLPTESMTTS VVPDVVEIDI NEIDSNIIPE V* 310**

**He185/333_cDNA_0004 -----SVEEE HLPTESMTTS AVPDVVEIDI NEIDSNIIPE V* 309**

**He185/333_cDNA_0005 -----SVEEE HLPTESMTTS VVPDVVEIDI NEIDSNIIPE V* 310**

**He185/333_cDNA_0006 -----SVEEE HLPTESMTTS VVPDVVEIDI NEIDSNIIPE V* 242**

**He185/333_cDNA_0007 -----SVEEE HLPTESMTTS VVPDVVEIDI NEIDSNIIPE V* 310**

**He185/333_cDNA_0008 -----SVEEE HLPTESMTTS AVPDVVEIDI NEIDSNIIPE V* 310**

**He185/333_cDNA_0009 -----SVEEE HLPTESMTTS VVPDVVEIDI NEIDSNIIPE V* 310**

**He185/333_cDNA_0010 -----SVEEE HLPTESMTTS VVPDVVEINI NEIDSNIIPE V* 310**

**He185/333_cDNA_0011 -----SVEEE HLPTESMTTS VVPDVVEIDI NEIDSNIIPE V* 310**

**He185/333_cDNA_0012 -----SEEEE HLPTESMTTS AVPDVVEIDI N-----IIPE V* 302**

**He185/333_cDNA_0013 -----SVEEE HLPTESMTTS AVPDVVEIDI NEIDSNIIPE V* 310**

**He185/333_cDNA_0014 -----SEEEE HLPTESMTTS AVPDVVEIDI N-----IIPE V* 302**

**He185/333_cDNA_0015 -----SEEEE HLPTESMTTS AVPDVVEIDI N-----IIPE V* 302**

**He185/333_cDNA_0016 RDGKENEEEE HLPTESMTTC AVIDVIEIDI N-----IIPE V* 302**

**He185/333_cDNA_0017 -----SEEEE HPPTESMTIS AVPDVVEIDI NEIDINIIPE E* 289**

**He185/333_cDNA_0018 ---------- ---------- ---------- ---------- -- 174**

**He185/333_cDNA_0019 -----SVEEE HLPTESMTTS VVPDVVEIDI NEIDSNIIPE V* 310**

**He185/333_cDNA_0020 -----SVEEE HLPTESMTTS AVPDVVEIDI NEIDSNIIPE V* 296**

**He185/333_cDNA_0021 -----SVEEE HLPTESMTTS VVPDVVEIDI NEIDSNIIPE V* 310**

**He185/333_cDNA_0022 -----CEEEE HLPTESMTTP AVPDVVEIDI N-----IIPE V* 72**

**He185/333_cDNA_0023 -----SEEEE HLPTESMTTS VVPDVVEIDI N-----IIPE V* 301**

**He185/333_cDNA_0024 -----SEEEE HLPTESMTTS AVPDVVEIDI N-----IIPE V* 278**

**He185/333_cDNA_0025 -----SEEEE HLPTESMTTS VVPDVVEIDI N-----IIPE V* 301**

**He185/333_cDNA_0026 -----SEEEE HLPTESMTTS AVPDVVEIDI N-----IIPE V* 302**

**He185/333_cDNA_0027 -----SEEEE HLPTESMTTS VVPDVVEIDI N-----IIPE V* 301**

**He185/333_cDNA_0028 -----SEEEE HLPTESMTTS AVPDVVEIDM N-----IIPE V* 267**

**He185/333_cDNA_0029 -----SEEEE HLPTESMTTS VVPDVVEIDI N-----IIPE V* 301**

**He185/333_cDNA_0030 -----SEEEE HLPTESMTTS AVPDVVEIDI N-----IIPE V* 302**

**He185/333_cDNA_0031 -----SEEEE HLPTESMTTS VVPDVVEIDI N-----IIPE V* 301**

**He185/333_cDNA_0032 -----SEEEE HLPTESMTTS AVPDVVEIDI N-----IIPE V* 267**

**He185/333_cDNA_0033 -----SEEEE HLPTESMTTS VVPDVVEIDI N-----IIPE V* 300**

**He185/333_cDNA_0034 -----SEEEE HLPTESMTTS VVPDVVEIDI N-----IIPE V* 301**

**He185/333_cDNA_0035 -----SEEEE HLPTESMTTS AVPDVVEIDI NEIDINIIPE V* 313**

**He185/333_cDNA_0036 -----SEEEE HLPTESMTIS AVPDVVEIDI NEIDINIIPE V* 349**

**He185/333_cDNA_0037 -----SEEEE HLPTESMTTS AVPDVVEIDI N-----IIPE V* 303**

**He185/333_cDNA_0038 -----SVEEE HLPTESMTTS VVPDVVEIDI NEIDSNIIPE V* 310**

**He185/333_cDNA_0039 -----SVEEE HLPTESMTTS VVPDVVEIDI NEIDSNIIPE V* 310**

**He185/333_cDNA_0040 -----SEEEE HLPTESMTTS AVPDVVEIDI NEIDSNIIPE V* 313**

**He185/333_cDNA_0041 -----SVEEE HLPTEGMTTS VVPDVVEINI NEIDSNIIPE V* 310**

**He185/333_cDNA_0042 -----GVEEE HLPTGSMTTS VVPDVVEIDI NEIDSNIIPE V* 310**

**He185/333_cDNA_0043 -----SEEEE HLPTESMTTS VVPDVVEIDI NEIDSNIIPG V* 306**

**He185/333_cDNA_0044 -----SVEEE HLPTESMTTS AVPDVVEIDI NEIDSNIIPE V* 310**

**He185/333_cDNA_0045 ---------- ---------- ---------- ---------- -- 52**

**He185/333_cDNA_0046 -----SEEEE HLPTESMTTS AVPDVVEIDI NEIDSNIIPE V* 313**

**He185/333_cDNA_0047 -----SEEEE HLPTESMTTS AVPDVVEIDI N-----IIPE V* 302**

**He185/333_cDNA_0048 -----SVEEE HLPTESMTTS AVPDVVEIDI NEIDSNIIPE V* 310**

**He185/333_cDNA_0049 -----SVEEE HLPAESMTTS AVPDVVEIDI NEIDSNIIPE V* 310**

**He185/333_cDNA_0050 -----SVEEE HLPTESMTTS AVPDVVEIDI NEIDSNIIPE V* 310**

**He185/333_cDNA_0051 -----SEEEE HLPTESMTTS AVPDVVEIDI NEIDSNIIPE V* 313**

**He185/333_cDNA_0052 -----SVEEE HLPTESMTTS AVPDVVEIDI NEIDSNIIPE V* 271**

**He185/333_cDNA_0053 -----SEEEE HLPTESMTTS AVPDVVEIDI NEIDSNIIPE V* 304**

**He185/333_cDNA_0054 -----SEEEE HLPTESMTTS VVPDVVEIDI N-----IIPE V* 301**

**He185/333_cDNA_0055 -----SEEEE HLPTESMTTS AVPDVVEIDI N-----IIPE V* 302**

**He185/333_cDNA_0056 -----SEEEE HLPTESMTTY AVPDVVEIDI D-----IVPE G* 302**

**He185/333_cDNA_0057 -----SEEEE HLPTESMTIS AVPDVVEIDI NEIDINIIPE V* 349**

**He185/333_cDNA_0058 -----SEEEE HLPTESMTIS AVPDVVEIDI N--------E V* 146**

**He185/333_cDNA_0059 -----SEEEE HLPTESMTIS AVPDVVEIDI NEIDINIIPE V* 311**

**He185/333_cDNA_0060 -----SEEEE HLPTESMTTS AVPDVVEIDI NEIDINIIPE V* 313**

**He185/333_cDNA_0061 -----SEEEE HLPTESMTIS AVPDVVEIDI NEIDINIIPE V* 349**

**He185/333_cDNA_0062 -----SEEEE HLPTESMTIS AVPDVVEIDI NEIDINIIPE V* 257**

**He185/333_cDNA_0063 -----SEEEE HLPTESMTIS AVPDVVEIDI NEIDINIIPE V* 349**

28

29

30

31

Repeat 4

**....|....| ....|....| ....|....| ....|....| ..**

**325 335 345 355**

**He185/333_cDNA_0064 -----SEEEE HLPTESMTIS AVPDVVEIDI NEIDINIIPE V* 349**

**He185/333_cDNA_0065 -----SEEEE HLPTESMTIS AVPDVVEIDI NEIDINIIPE V* 325**

**He185/333_cDNA_0066 -----SEEEE HLPTESMTIS AVPDVVEIDI NEIDINIIPE V* 226**

**He185/333_cDNA_0067 -----SEEEE HLPTESMTIS AVPDVVEIDI NEIDINIVAE V* 286**

**He185/333_cDNA_0068 -----SEEEE HLPTESMTTS AVPDVVEIDI NEIDSNIVAE V* 313**

**He185/333_cDNA_0069 ---------- ---------- ---------- ---------- -- 43**

**He185/333_cDNA_0070 -----SVEEE HLPTESMTTS AVPDVVEIDI NEIDSNIVAE V* 310**

**He185/333_cDNA_0071 -----SVEEE HLPTESMTTS AVPDVVEIDI NEIDSNIVAE V* 310**

**He185/333_cDNA_0072 -----SVEEE HLPTESMTTS AVPDVVEIDI NEIDSNIVAE V* 310**

**He185/333_cDNA_0073 -----SVEEE HLPTESMTTS AVPDVVEIDI NEIDSNIVAE V* 310**

**He185/333_cDNA_0074 -----SVEEE HLPTESMTTS AVPDVVEIDI NEIDSNIVAE V* 310**

**He185/333_cDNA_0075 -----SEGEE HLPTESMTIS AVPDVVEIDI NEIDINIVAE V* 349**

**He185/333_cDNA_0076 -----SVEEE HLPTESMTTS VVPDVVEIDI NEIDSNIVAE V* 310**

**He185/333_cDNA_0077 -----SEEEE HLPTESMTTS AVPDVVEIDI N-----IVAE V* 302**

**He185/333_cDNA_0078 -----SVEEE HLPTESMTTS AVPDVVEIDI NEIDSNIVAE V* 310**

**He185/333_cDNA_0079 -----SVEEE HLPTESMTTS AVPDVVEIDI NEIDSNIVAE V* 310**

**He185/333_cDNA_0080 -----SVEEE HLPTESMTTS VVPDVVEIDI NEIDSNIVAE V* 310**

**He185/333_cDNA_0081 -----SEEEE HLPTESMTTS VVPDVVEIDI N-----IIPE V* 301**

**He185/333_cDNA_0082 -----SEEEE HLPTESMTTS AVPDVVEIDI N-----IIPE V* 311**

**He185/333_cDNA_0083 -----SEEEE HLPTESMTTS AVPDVVEIDI N-----IIPE V* 301**

**He185/333_cDNA_0084 -----SEEEE HLPTESMTTS AVPDVVEIDI N-----IIPE V* 302**

**He185/333_cDNA_0085 -----SVEEE HLPTESMTTS AVPDVVEIDI NEIDINIIPE V* 319**

**He185/333_cDNA_0086 -----SEEEE HLPTESMTTS AVPDVVEIDI N-----IIPE V* 302**

**He185/333_cDNA_0087 -----SEEEE HLPTESMTTS VVPDVVEIDI N-----IIPE V* 302**

**He185/333_cDNA_0088 -----SEEEE HLPTESMTIS AVPDVVEIDI NEIDINIIPE V* 349**

**He185/333_cDNA_0089 -----SEEEE HLPTESMTIS AVPDVVEIDI NEIDINIIPE V* 349**

**He185/333_cDNA_0090 -----SEEEE HLPTESMTTS AVPDVVEIDI N-----IIPE V* 302**

**He185/333_cDNA_0091 -----SEEEE HLPTESMTTS AVPDVVEIDI N-----IIPE V* 302**

**He185/333_cDNA_0092 -----SVEEE HLPTESMTTS AVPDVVEIDI NEIDINIIPE V* 319**

**He185/333_cDNA_0093 -----SVEEE HLPTESMTTS AVPDVVEIDI NEIDINIIPE V* 228**

**He185/333_cDNA_0094 -----SEEEE HLPTESMTIS AVPDVVEIDI NEIDINIIPE V* 349**

**He185/333_cDNA_0095 -----SEEEE HLPTESMTIS AVPDVVEIDI NEIDINIIPE V* 349**

**He185/333_cDNA_0096 -----SVEEE HLPTESMTTS AVPDVVEIDI NEIDINIIPE V* 307**

**He185/333_cDNA_0097 -----SEEEE HLPTESMTTS AVPDVVEIDI N-----IIPE V* 302**

**He185/333_cDNA_0098 -----SEEEE HLPTESMTIS AVPDVVEIDI NEIDINIIPE V* 349**

**He185/333_cDNA_0099 -----SEEEE HLPTESMTTS AVPDVVEIDI N-----IIPE V* 302**

**He185/333_cDNA_0100 -----SEEEE HLPTESMTTS VVPDVVEIDI N-----IIPE V* 301**

**He185/333_cDNA_0101 -----SEEEE HLPTESMTIS AVPDVVEIDI NEIDINIIPE V* 307**

**He185/333_cDNA_0102 -----SEEEE HLPTESMTTS AVPDVVEIDI N-----IIPE V* 301**

**He185/333_cDNA_0103 -----SEEEE HLPTESMTIS AVPDVVEIDI NEIDINIIPE V* 349**

**He185/333_cDNA_0104 -----SEEEE HLPTESMTTS AVPDVVEIDI N-----IIPE V* 302**

**He185/333_cDNA_0105 ---------- ---------- ---------- ---------- -- 153**

**He185/333_cDNA_0106 ---------- ---------- ---------- ---------- -- 182**

**He185/333_cDNA_0107 ---------- ---------- ---------- ---------- -- 97**

**He185/333_cDNA_0108 ---------- ---------- ---------- ---------- -- 231**

**He185/333_cDNA_0109 ---------- ---------- ---------- ---------- -- 125**

**He185/333_cDNA_0110 ---------- ---------- ---------- ---------- -- 231**

**He185/333_cDNA_0111 ---------- ---------- ---------- ---------- -- 230**

**He185/333_cDNA_0112 ---------- ---------- ---------- ---------- -- 202**
