# Supplementary material for: Characterization of the Highly Variable Immune Response Gene Family, He185/333, in the Sea Urchin, Heliocidaris erythrogramma
Source: PLoS One. 2014 Oct 21;9(10):e62079. doi: 10.1371/journal.pone.0062079 (PMC4204807; doi:10.1371/journal.pone.0062079)
Supplement: Figure S5 — Repeats found in 104 translated He185/333 cDNA sequences, presented as sequence logos and linear sequences. The figure shows repeat types and maximum copy number (left column), as well as sequence variations within repeats (central column) and the structure of each repeat type (right column). For example, there are up to three copies of the type 1 repeat (1.1, 1.2 and 1.3) in He185/333 deduced polypeptides. Sequence variations within repeats are depicted as sequence logos and as plain text. Sequence logos were generated using the software, Geneious (Geneious v5.4, http://www.geneious.com). The size of each letter within the sequence logos is proportional to the frequency of that residue at the specific position in the He185/333 alignment. For example, the first position of repeat 1.1 is G (glycine), with a value of 1, because it is invariant at that position amongst the 104 He185/333 sequences. In the plain text below the logos variant amino acids at specific positions are indicated in brackets. (DOCX) [file pone.0062079.s005.docx]

| **Type** | **Sequence** | **Structure** |
| --- | --- | --- |
| 1.1 | GG(P/A)PMGGRR(F/C)DG(P/N)G 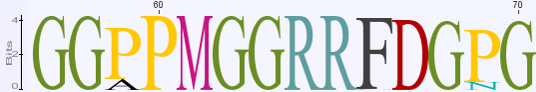 | Interspersed, incomplete |
| 1.2 | G(G/R)P(M/V/I)G(G/S)(R/G)(R/K)FD(G/V)PG 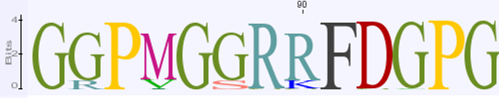 |  |
| 1.3 | 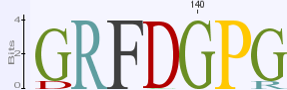 (G/D)RF(D/V)GP(G/R) |  |
| 2.1 | HN(K/N/R)T(D/G/N)(D/N)(H/R)(H/R)(H/R)H N(H/R/L) (T/P) E(G/S)(H/R)(R/C)(H/P)(H/Q) 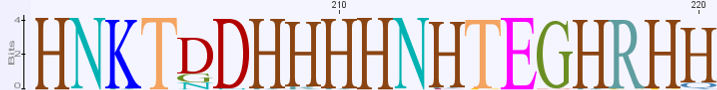 | Tandem,  Interspersed, incomplete |
| 2.2 | 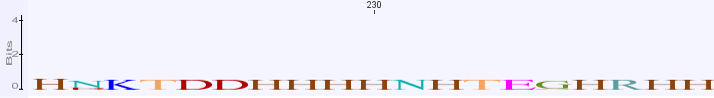 H(N/D)KTDDHHHHNH(T/S)EGHRHH |  |
| 2.3 | HNKTDDHHHHNHTE(G/S)HRHH 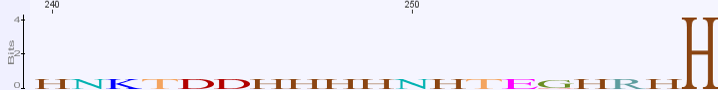 |  |
| 2.4 | HNKTEE 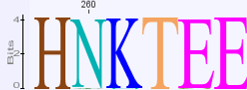 |  |
| 2.5 | (N/S/D)H(T/H)(E/D/K)E 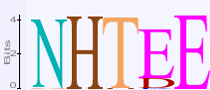 |  |
| 3.1 | PF(R/W)F(N/F/D) 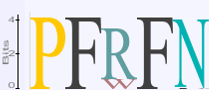 | Tandem |
| 3.2 | (P/A/L/S/H)(F/L)(G/F)(R/H)K 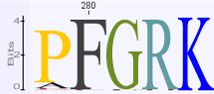 |  |
| 3.3 | 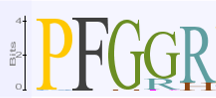 (P/Q)FG(G/R/E)(R/H) |  |
| **Type** | **Sequence** | **Structure** |
| 3.4 | (P/Q)FG(R/G)(R/C) 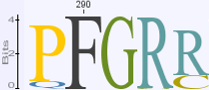 | Tandem |
| 4.1 | 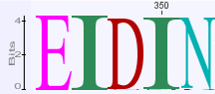 EI(D/N)(I/M)(N/D) | Tandem |
| 4.2 | EID(S/I)N 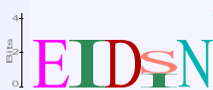 |  |
