# Supplementary material for: Characterization of the Highly Variable Immune Response Gene Family, He185/333, in the Sea Urchin, Heliocidaris erythrogramma
Source: PLoS One. 2014 Oct 21;9(10):e62079. doi: 10.1371/journal.pone.0062079 (PMC4204807; doi:10.1371/journal.pone.0062079)
Supplement: Figure S6 — Comparison of the four types of repeats from He185/333 translated sequences with five Sp185/333 repeat types (15). The number of copies of each type of repeat varies between the two species and among sequences within one species. For example, type 1 repeat is present as two complete tandem repeats and one interspersed incomplete repeat in H. erythrogramma but as up to four tandem repeats in S. purpuratus. In He185/333, type 3 repeat appears as four tandem copies and is homologous to a portion of Sp185/333 type 5 repeat. Repeat type 5 is present up to three times in Sp185/333. Similarly, the last four residues of S. purpuratus repeat type 4 (GDQD) are not part of the homologous repeat sequence in He185/333. Most, but not all, He185/333 repeats had homologues amongst Sp185/333 sequences, as some repeats were unique to each species. For example, although the type 4 repeat sequence in He185/333 was homologous to a sequence stretch in Sp185/333, the latter was not repeated in Sp185/333. Finally, repeat type 2 of H. erythrogramma is present up to five times (four complete in tandem plus one incomplete repeats). The homologous sequences in Sp185/333 are composed of types 2, 3 and 4. (DOCX) [file pone.0062079.s006.docx]

10 20 30 40

....|....|....|....|....|....|....|....|....|.....

He185/333_repeat-type_1 QMMGGPRQGGPPMGGRRFDGPGQGDQQMDGRGPNGGPMGGRRFDGPGFGG

Type 1

Type 1

(2 of up to 3 copies)

Sp185/333_repeat_type_1 PPMGGPRQDGGPMGGRRFDGPGFGTPQMDGRRQNGGPMGGRRFDGPRFGG

Type 1

Type 1

(2 of up to 4 copies)

10 20 30 40 50 60

....|....|....|....|....|....|....|....|....|....|....|....|....|..

He185/333_repeat_type_2 HNKTGDHHHHNHTEGHRHHHNKTDDHHHHNHTEGHRHHHNKTDDHHHHNHTEGHRHHHNKTEEGDQD

(4 of up to 5 copies)

Type 2

Type 2

Type 2

Type 2

(incomplete)

Sp185/333_repeat_types_2,3,4 NHTEGHQGH NETGDHPH RHHNKTGDGDQD

Type 4

Type 3

Type 2

(1 each of up to 5, 4, 2

copies, respectively)

10 20

....|....|....|....|....|...

He185/333_repeat_type_3 DRP--EMRPFRFNPFGRKPFGGRPFGRR

(4 of up to 4 copies)

Type 3

Sp185/333_repeat_type_5 DRPMFGMRPFRFNPFGRKPFGGRPFDRR

(1 of up to 3 copies)

Type 5

10

....|....|

He185/333_repeat_type_5 EIDINEIDIN

(2 of up to 2 copies)

Type 4

Sp185/333_no_repeat EIAVNEEDIN
