# Supplementary material for: Characterization of the Highly Variable Immune Response Gene Family, He185/333, in the Sea Urchin, Heliocidaris erythrogramma
Source: PLoS One. 2014 Oct 21;9(10):e62079. doi: 10.1371/journal.pone.0062079 (PMC4204807; doi:10.1371/journal.pone.0062079)
Supplement: Table S1 — Genbank accession numbers of He185/333 and Sp185/333 sequences. (DOC) [file pone.0062079.s007.doc]

Supporting Information table S1. Genbank accession numbers of *He185/333* and *Sp185/333* sequences.

| Genbank accession | Sequence name in Genbank file | Sequence name for phylogenetic analysis (figure 7 in main text) | Sequence name for diversity analysis (figure 8 in main text) |
| --- | --- | --- | --- |
| JQ780210 | He185/333_cDNA_0001 | HE009 | HE_185_cDNA_POOL_9 |
| JQ780211 | He185/333_cDNA_0002 |  | HE_185_cDNA_POOL_10 |
| JQ780212 | He185/333_cDNA_0003 |  | HE_185_cDNA_POOL_11 |
| JQ780213 | He185/333_cDNA_0004 |  | HE_185_cDNA_POOL_12 |
| JQ780214 | He185/333_cDNA_0005 | HE013 | HE_185_cDNA_POOL_13 |
| JQ780215 | He185/333_cDNA_0006 |  | HE_185_cDNA_POOL_14 |
| JQ780216 | He185/333_cDNA_0007 |  | HE_185_cDNA_POOL_15 |
| JQ780217 | He185/333_cDNA_0008 |  | HE_185_cDNA_POOL_16 |
| JQ780218 | He185/333_cDNA_0009 | HE017 | HE_185_cDNA_POOL_17 |
| JQ780219 | He185/333_cDNA_0010 |  | HE_185_cDNA_POOL_18 |
| JQ780220 | He185/333_cDNA_0011 |  | HE_185_cDNA_POOL_19 |
| JQ780221 | He185/333_cDNA_0012 |  | HE_185_cDNA_POOL_20 |
| JQ780222 | He185/333_cDNA_0013 | HE021 | HE_185_cDNA_POOL_21 |
| JQ780223 | He185/333_cDNA_0014 |  | HE_185_cDNA_POOL_22 |
| JQ780224 | He185/333_cDNA_0015 |  | HE_185_cDNA_POOL_23 |
| JQ780225 | He185/333_cDNA_0016 |  | HE_185_cDNA_POOL_24 |
| JQ780226 | He185/333_cDNA_0017 | HE025 | HE_185_cDNA_POOL_25 |
| JQ780227 | He185/333_cDNA_0018 |  | HE_185_cDNA_POOL_26 |
| JQ780228 | He185/333_cDNA_0019 |  | HE_185_cDNA_POOL_27 |
| JQ780229 | He185/333_cDNA_0020 |  | HE_185_cDNA_POOL_28 |
| JQ780230 | He185/333_cDNA_0021 | HE029 | HE_185_cDNA_POOL_29 |
| JQ780231 | He185/333_cDNA_0022 |  | HE_185_cDNA_POOL_30 |
| JQ780232 | He185/333_cDNA_0023 |  | HE_185_cDNA_POOL_31 |
| JQ780233 | He185/333_cDNA_0024 |  | HE_185_cDNA_POOL_32 |
| JQ780234 | He185/333_cDNA_0025 | HE033 | HE_185_cDNA_POOL_33 |
| JQ780235 | He185/333_cDNA_0026 |  | HE_185_cDNA_POOL_34 |
| JQ780236 | He185/333_cDNA_0027 |  | HE_185_cDNA_POOL_35 |
| JQ780237 | He185/333_cDNA_0028 |  | HE_185_cDNA_POOL_36 |
| JQ780238 | He185/333_cDNA_0029 | HE037 | HE_185_cDNA_POOL_37 |
| JQ780239 | He185/333_cDNA_0030 |  | HE_185_cDNA_POOL_38 |
| JQ780240 | He185/333_cDNA_0031 |  | HE_185_cDNA_POOL_39 |
| JQ780241 | He185/333_cDNA_0032 |  | HE_185_cDNA_POOL_40 |
| JQ780242 | He185/333_cDNA_0033 | HE041 | HE_185_cDNA_POOL_41 |
| JQ780243 | He185/333_cDNA_0034 |  | HE_185_cDNA_POOL_42 |
| JQ780244 | He185/333_cDNA_0035 |  | HE_185_cDNA_POOL_43 |
| JQ780245 | He185/333_cDNA_0036 |  | HE_185_cDNA_POOL_44 |
| JQ780246 | He185/333_cDNA_0037 | HE045 | HE_185_cDNA_POOL_45 |
| JQ780247 | He185/333_cDNA_0038 |  | HE_185_cDNA_POOL_46 |
| JQ780248 | He185/333_cDNA_0039 |  | HE_185_cDNA_POOL_47 |
| JQ780249 | He185/333_cDNA_0040 |  | HE_185_cDNA_POOL_48 |
| JQ780250 | He185/333_cDNA_0041 | HE049 | HE_185_cDNA_POOL_49 |
| JQ780251 | He185/333_cDNA_0042 |  | HE_185_cDNA_POOL_50 |
| Genbank accession | Sequence name in Genbank file | Sequence name for phylogenetic analysis (figure 7 in main text) | Sequence name for diversity analysis (figure 8 in main text) |
| JQ780252 | He185/333_cDNA_0043 |  | HE_185_cDNA_POOL_51 |
| JQ780253 | He185/333_cDNA_0044 |  | HE_185_cDNA_POOL_52 |
| JQ780254 | He185/333_cDNA_0045 |  | HE_185_cDNA_POOL_53 |
| JQ780255 | He185/333_cDNA_0046 | HE054 | HE_185_cDNA_POOL_54 |
| JQ780256 | He185/333_cDNA_0047 |  | HE_185_cDNA_POOL_55 |
| JQ780257 | He185/333_cDNA_0048 |  | HE_185_cDNA_POOL_56 |
| JQ780258 | He185/333_cDNA_0049 | HE057 | HE_185_cDNA_POOL_57 |
| JQ780259 | He185/333_cDNA_0050 |  | HE_185_cDNA_POOL_58 |
| JQ780260 | He185/333_cDNA_0051 |  | HE_185_cDNA_POOL_59 |
| JQ780261 | He185/333_cDNA_0052 |  | HE_185_cDNA_POOL_60 |
| JQ780262 | He185/333_cDNA_0053 | HE061 | HE_185_cDNA_POOL_61 |
| JQ780263 | He185/333_cDNA_0054 |  | HE_185_cDNA_POOL_62 |
| JQ780264 | He185/333_cDNA_0055 |  | HE_185_cDNA_POOL_63 |
| JQ780265 | He185/333_cDNA_0056 |  | HE_185_cDNA_POOL_64 |
| JQ780266 | He185/333_cDNA_0057 | HE065 | HE_185_cDNA_POOL_65 |
| JQ780267 | He185/333_cDNA_0058 |  | HE_185_cDNA_POOL_66 |
| JQ780268 | He185/333_cDNA_0059 |  | HE_185_cDNA_POOL_67 |
| JQ780269 | He185/333_cDNA_0060 |  | HE_185_cDNA_POOL_68 |
| JQ780270 | He185/333_cDNA_0061 | HE069 | HE_185_cDNA_POOL_69 |
| JQ780271 | He185/333_cDNA_0062 |  | HE_185_cDNA_POOL_70 |
| JQ780272 | He185/333_cDNA_0063 |  | HE_185_cDNA_POOL_71 |
| JQ780273 | He185/333_cDNA_0064 |  | HE_185_cDNA_POOL_72 |
| JQ780274 | He185/333_cDNA_0065 | HE073 | HE_185_cDNA_POOL_73 |
| JQ780275 | He185/333_cDNA_0066 |  | HE_185_cDNA_POOL_74 |
| JQ780276 | He185/333_cDNA_0067 |  | HE_185_cDNA_POOL_75 |
| JQ780277 | He185/333_cDNA_0068 |  | HE_185_cDNA_POOL_76 |
| JQ780278 | He185/333_cDNA_0069 |  | HE_185_cDNA_POOL_77 |
| JQ780279 | He185/333_cDNA_0070 | HE078 | HE_185_cDNA_POOL_78 |
| JQ780280 | He185/333_cDNA_0071 |  | HE_185_cDNA_POOL_79 |
| JQ780281 | He185/333_cDNA_0072 |  | HE_185_cDNA_POOL_80 |
| JQ780282 | He185/333_cDNA_0073 | HE081 | HE_185_cDNA_POOL_81 |
| JQ780283 | He185/333_cDNA_0074 |  | HE_185_cDNA_POOL_82 |
| JQ780284 | He185/333_cDNA_0075 |  | HE_185_cDNA_POOL_83 |
| JQ780285 | He185/333_cDNA_0076 |  | HE_185_cDNA_POOL_84 |
| JQ780286 | He185/333_cDNA_0077 | HE085 | HE_185_cDNA_POOL_85 |
| JQ780287 | He185/333_cDNA_0078 |  | HE_185_cDNA_POOL_86 |
| JQ780288 | He185/333_cDNA_0079 |  | HE_185_cDNA_POOL_87 |
| JQ780289 | He185/333_cDNA_0080 |  | HE_185_cDNA_POOL_88 |
| JQ780290 | He185/333_cDNA_0081 |  | HE_185_cDNA_POOL_102 |
| JQ780291 | He185/333_cDNA_0082 |  | HE_185_cDNA_POOL_100 |
| JQ780292 | He185/333_cDNA_0083 |  | HE_185_cDNA_POOL_103 |
| JQ780293 | He185/333_cDNA_0084 |  | HE_185_cDNA_POOL_109 |
| JQ780294 | He185/333_cDNA_0085 |  | HE_185_cDNA_POOL_95 |
| JQ780295 | He185/333_cDNA_0086 |  | HE_185_cDNA_POOL_106 |
| Genbank accession | Sequence name in Genbank file | Sequence name for phylogenetic analysis (figure 7 in main text) | Sequence name for diversity analysis (figure 8 in main text) |
| JQ780296 | He185/333_cDNA_0087 |  | HE_185_cDNA_POOL_107 |
| JQ780297 | He185/333_cDNA_0088 |  | HE_185_cDNA_POOL_92 |
| JQ780298 | He185/333_cDNA_0089 |  | HE_185_cDNA_POOL_90 |
| JQ780299 | He185/333_cDNA_0090 |  | HE_185_cDNA_POOL_110 |
| JQ780300 | He185/333_cDNA_0091 |  | HE_185_cDNA_POOL_111 |
| JQ780301 | He185/333_cDNA_0092 |  | HE_185_cDNA_POOL_96 |
| JQ780302 | He185/333_cDNA_0093 |  | HE_185_cDNA_POOL_112 |
| JQ780303 | He185/333_cDNA_0094 |  | HE_185_cDNA_POOL_94 |
| JQ780304 | He185/333_cDNA_0095 | HE089 | HE_185_cDNA_POOL_89 |
| JQ780305 | He185/333_cDNA_0096 |  | HE_185_cDNA_POOL_98 |
| JQ780306 | He185/333_cDNA_0097 |  | HE_185_cDNA_POOL_105 |
| JQ780307 | He185/333_cDNA_0098 | HE093 | HE_185_cDNA_POOL_93 |
| JQ780308 | He185/333_cDNA_0099 |  | HE_185_cDNA_POOL_108 |
| JQ780309 | He185/333_cDNA_0100 |  | HE_185_cDNA_POOL_99 |
| JQ780310 | He185/333_cDNA_0101 | HE097 | HE_185_cDNA_POOL_97 |
| JQ780311 | He185/333_cDNA_0102 |  | HE_185_cDNA_POOL_101 |
| JQ780312 | He185/333_cDNA_0103 |  | HE_185_cDNA_POOL_91 |
| JQ780313 | He185/333_cDNA_0104 |  | HE_185_cDNA_POOL_104 |
| JQ780314 | He185/333_cDNA_0105 |  | HE_185_cDNA_POOL_7 |
| JQ780315 | He185/333_cDNA_0106 |  | HE_185_cDNA_POOL_8 |
| JQ780316 | He185/333_cDNA_0107 |  | HE_185_cDNA_POOL_6 |
| JQ780317 | He185/333_cDNA_0108 | HE005 | HE_185_cDNA_POOL_5 |
| JQ780318 | He185/333_cDNA_0109 |  | HE_185_cDNA_POOL_4 |
| JQ780319 | He185/333_cDNA_0110 |  | HE_185_cDNA_POOL_2 |
| JQ780320 | He185/333_cDNA_0111 | HE001 | HE_185_cDNA_POOL_1 |
| JQ780321 | He185/333_cDNA_0112 |  | HE_185_cDNA_POOL_3 |
| JQ780171 | He185/333_gDNA_0001 |  |  |
| JQ780172 | He185/333_gDNA_0002 |  |  |
| JQ780173 | He185/333_gDNA_0003 |  |  |
| JQ780174 | He185/333_gDNA_0004 |  |  |
| JQ780175 | He185/333_gDNA_0005 |  |  |
| JQ780176 | He185/333_gDNA_0006 |  |  |
| JQ780177 | He185/333_gDNA_0007 |  |  |
| JQ780178 | He185/333_gDNA_0008 |  |  |
| JQ780179 | He185/333_gDNA_0009 |  |  |
| JQ780180 | He185/333_gDNA_0010 |  |  |
| JQ780181 | He185/333_gDNA_0011 |  |  |
| JQ780182 | He185/333_gDNA_0012 |  |  |
| JQ780183 | He185/333_gDNA_0013 |  |  |
| JQ780184 | He185/333_gDNA_0014 |  |  |
| JQ780185 | He185/333_gDNA_0015 |  |  |
| JQ780186 | He185/333_gDNA_0016 |  |  |
| JQ780187 | He185/333_gDNA_0017 |  |  |
| JQ780188 | He185/333_gDNA_0018 |  |  |
| Genbank accession | Sequence name in Genbank file | Sequence name for phylogenetic analysis (figure 7 in main text) | Sequence name for diversity analysis (figure 8 in main text) |
| JQ780189 | He185/333_gDNA_0019 |  |  |
| JQ780190 | He185/333_gDNA_0020 |  |  |
| JQ780191 | He185/333_gDNA_0021 |  |  |
| JQ780192 | He185/333_gDNA_0022 |  |  |
| JQ780193 | He185/333_gDNA_0023 |  |  |
| JQ780194 | He185/333_gDNA_0024 |  |  |
| JQ780195 | He185/333_gDNA_0025 |  |  |
| JQ780196 | He185/333_gDNA_0026 |  |  |
| JQ780197 | He185/333_gDNA_0027 |  |  |
| JQ780198 | He185/333_gDNA_0028 |  |  |
| JQ780199 | He185/333_gDNA_0029 |  |  |
| JQ780200 | He185/333_gDNA_0030 |  |  |
| JQ780201 | He185/333_gDNA_0031 |  |  |
| JQ780202 | He185/333_gDNA_0032 |  |  |
| JQ780203 | He185/333_gDNA_0033 |  |  |
| JQ780204 | He185/333_gDNA_0034 |  |  |
| JQ780205 | He185/333_gDNA_0035 |  |  |
| JQ780206 | He185/333_gDNA_0036 |  |  |
| JQ780207 | He185/333_gDNA_0037 |  |  |
| JQ780208 | He185/333_gDNA_0038 |  |  |
| JQ780209 | He185/333_gDNA_0039 |  |  |
| DQ183104.1 |  | SP001 | SP_185_cDNA_1 |
| EF065996.1 |  |  | SP_185_cDNA_2 |
| EF066327.1 |  |  | SP_185_cDNA_3 |
| EF066267.1 |  |  | SP_185_cDNA_4 |
| EF066232.1 |  |  | SP_185_cDNA_5 |
| EF066235.1 |  |  | SP_185_cDNA_6 |
| EF066253.1 |  |  | SP_185_cDNA_7 |
| EF066277.1 |  |  | SP_185_cDNA_8 |
| EF066236.1 |  |  | SP_185_cDNA_9 |
| EF066248.1 |  | SP010 | SP_185_cDNA_10 |
| EF066259.1 |  |  | SP_185_cDNA_11 |
| EF066257.1 |  |  | SP_185_cDNA_12 |
| EF066252.1 |  |  | SP_185_cDNA_13 |
| EF066241.1 |  |  | SP_185_cDNA_14 |
| EF066234.1 |  |  | SP_185_cDNA_15 |
| EF066242.1 |  |  | SP_185_cDNA_16 |
| EF066294.1 |  |  | SP_185_cDNA_17 |
| EF066288.1 |  |  | SP_185_cDNA_18 |
| EF066210.1 |  | SP019 | SP_185_cDNA_19 |
| EF066285.1 |  |  | SP_185_cDNA_20 |
| EF066286.1 |  |  | SP_185_cDNA_21 |
| EF066322.1 |  |  | SP_185_cDNA_22 |
| EF066291.1 |  |  | SP_185_cDNA_23 |
| Genbank accession | Sequence name in Genbank file | Sequence name for phylogenetic analysis (figure 7 in main text) | Sequence name for diversity analysis (figure 8 in main text) |
| DQ183149.1 |  |  | SP_185_cDNA_24 |
| EF066326.1 |  |  | SP_185_cDNA_25 |
| EF066297.1 |  |  | SP_185_cDNA_26 |
| EF066218.1 |  |  | SP_185_cDNA_27 |
| EF066216.1 |  | SP028 | SP_185_cDNA_28 |
| EF066220.1 |  |  | SP_185_cDNA_29 |
| EF066217.1 |  |  | SP_185_cDNA_30 |
| EF066219.1 |  |  | SP_185_cDNA_31 |
| EF066273.1 |  |  | SP_185_cDNA_32 |
| EF065825.1 |  |  | SP_185_cDNA_33 |
| EF065877.1 |  |  | SP_185_cDNA_34 |
| EF066268.1 |  |  | SP_185_cDNA_35 |
| EF066159.1 |  |  | SP_185_cDNA_36 |
| EF066228.1 |  | SP037 | SP_185_cDNA_37 |
| EF066275.1 |  |  | SP_185_cDNA_38 |
| EF066272.1 |  |  | SP_185_cDNA_39 |
| EF066271.1 |  |  | SP_185_cDNA_40 |
| EF066110.1 |  |  | SP_185_cDNA_41 |
| DQ183104.1 |  |  | SP_185_cDNA_42 |
| EF066278.1 |  |  | SP_185_cDNA_43 |
| EF066090.1 |  |  | SP_185_cDNA_44 |
| EF066284.1 |  |  | SP_185_cDNA_45 |
| EF066230.1 |  | SP046 | SP_185_cDNA_46 |
| EF066095.1 |  |  | SP_185_cDNA_47 |
| EF065994.1 |  |  | SP_185_cDNA_48 |
| EF066156.1 |  |  | SP_185_cDNA_49 |
| EF066138.1 |  |  | SP_185_cDNA_50 |
| EF066069.1 |  |  | SP_185_cDNA_51 |
| EF066153.1 |  |  | SP_185_cDNA_52 |
| EF066142.1 |  |  | SP_185_cDNA_53 |
| EF066157.1 |  |  | SP_185_cDNA_54 |
| EF066107.1 |  | SP055 | SP_185_cDNA_55 |
| EF066150.1 |  |  | SP_185_cDNA_56 |
| EF066312.1 |  |  | SP_185_cDNA_57 |
| EF066317.1 |  |  | SP_185_cDNA_58 |
| EF066325.1 |  |  | SP_185_cDNA_59 |
| EF066295.1 |  |  | SP_185_cDNA_60 |
| EF066313.1 |  |  | SP_185_cDNA_61 |
| EF066290.1 |  |  | SP_185_cDNA_62 |
| EF066320.1 |  |  | SP_185_cDNA_63 |
| EF066276.1 |  | SP064 | SP_185_cDNA_64 |
| EF066225.1 |  |  | SP_185_cDNA_65 |
| EF066270.1 |  |  | SP_185_cDNA_66 |
| EF066224.1 |  |  | SP_185_cDNA_67 |
| Genbank accession | Sequence name in Genbank file | Sequence name for phylogenetic analysis (figure 7 in main text) | Sequence name for diversity analysis (figure 8 in main text) |
| EF066239.1 |  |  | SP_185_cDNA_68 |
| EF066280.1 |  |  | SP_185_cDNA_69 |
| EF066244.1 |  |  | SP_185_cDNA_70 |
| EF066240.1 |  |  | SP_185_cDNA_71 |
| EF066226.1 |  |  | SP_185_cDNA_72 |
| EF066231.1 |  | SP073 | SP_185_cDNA_73 |
| EF066269.1 |  |  | SP_185_cDNA_74 |
| EF066245.1 |  |  | SP_185_cDNA_75 |
| EF066283.1 |  |  | SP_185_cDNA_76 |
| EF065781.1 |  |  | SP_185_cDNA_77 |
| EF065777.1 |  |  | SP_185_cDNA_78 |
| EF065741.1 |  |  | SP_185_cDNA_79 |
| EF065723.1 |  |  | SP_185_cDNA_80 |
| EF065773.1 |  |  | SP_185_cDNA_81 |
| EF065720.1 |  |  | SP_185_cDNA_82 |
| EF065721.1 |  |  | SP_185_cDNA_83 |
| EF065732.1 |  | SP083 | SP_185_cDNA_84 |
| EF065727.1 |  |  | SP_185_cDNA_85 |
| EF065772.1 |  |  | SP_185_cDNA_86 |
| EF065746.1 |  |  | SP_185_cDNA_87 |
| EF065731.1 |  |  | SP_185_cDNA_88 |
| EF065735.1 |  |  | SP_185_cDNA_89 |
| EF065726.1 |  |  | SP_185_cDNA_90 |
| EF065765.1 |  |  | SP_185_cDNA_91 |
| EF065730.1 |  | SP091 | SP_185_cDNA_92 |
| EF065768.1 |  |  | SP_185_cDNA_93 |
| EF065759.1 |  |  | SP_185_cDNA_94 |
| EF065729.1 |  |  | SP_185_cDNA_95 |
| EF065740.1 |  |  | SP_185_cDNA_96 |
| EF065719.1 |  |  | SP_185_cDNA_97 |
| EF065747.1 |  |  | SP_185_cDNA_98 |
| EF065724.1 |  |  | SP_185_cDNA_99 |
| EF065733.1 |  |  | SP_185_cDNA_100 |
| EF065763.1 |  | SP100 | SP_185_cDNA_101 |
| EF065742.1 |  |  | SP_185_cDNA_102 |
| EF065736.1 |  |  | SP_185_cDNA_103 |
| EF065728.1 |  |  | SP_185_cDNA_104 |
| EF065749.1 |  |  | SP_185_cDNA_105 |
| EF065783.1 |  |  | SP_185_cDNA_106 |
| EF065784.1 |  |  | SP_185_cDNA_107 |
| EF065782.1 |  |  | SP_185_cDNA_108 |
| EF065787.1 |  |  | SP_185_cDNA_109 |
| EF065790.1 |  | SP109 | SP_185_cDNA_110 |
| EF065786.1 |  |  | SP_185_cDNA_111 |
| Genbank accession | Sequence name in Genbank file | Sequence name for phylogenetic analysis (figure 7 in main text) | Sequence name for diversity analysis (figure 8 in main text) |
| EF065791.1 |  |  | SP_185_cDNA_112 |
| EF065789.1 |  |  | SP_185_cDNA_113 |
| EF065753.1 |  |  | SP_185_cDNA_114 |
| EF065750.1 |  |  | SP_185_cDNA_115 |
| EF065767.1 |  |  | SP_185_cDNA_116 |
| EF065758.1 |  |  | SP_185_cDNA_117 |
| EF065766.1 |  |  | SP_185_cDNA_118 |
| EF065752.1 |  | SP118 | SP_185_cDNA_119 |
| EF065769.1 |  |  | SP_185_cDNA_120 |
| EF065737.1 |  |  | SP_185_cDNA_121 |
| EF065744.1 |  |  | SP_185_cDNA_122 |
| EF065743.1 |  |  | SP_185_cDNA_123 |
| EF066112.1 |  |  | SP_185_cDNA_124 |
| EF066045.1 |  |  | SP_185_cDNA_125 |
| EF066119.1 |  |  | SP_185_cDNA_126 |
| EF066050.1 |  |  | SP_185_cDNA_127 |
| EF066051.1 |  | SP127 | SP_185_cDNA_128 |
| EF066052.1 |  |  | SP_185_cDNA_129 |
| EF066125.1 |  |  | SP_185_cDNA_130 |
| EF065864.1 |  |  | SP_185_cDNA_131 |
| EF066135.1 |  |  | SP_185_cDNA_132 |
| EF066120.1 |  |  | SP_185_cDNA_133 |
| EF066137.1 |  |  | SP_185_cDNA_134 |
| EF066107.1 |  |  | SP_185_cDNA_135 |
| EF066115.1 |  |  | SP_185_cDNA_136 |
| EF066064.1 |  | SP136 | SP_185_cDNA_137 |
| EF066118.1 |  |  | SP_185_cDNA_138 |
| EF066126.1 |  |  | SP_185_cDNA_139 |
| EF066161.1 |  |  | SP_185_cDNA_140 |
| EF065826.1 |  |  | SP_185_cDNA_141 |
| EF066132.1 |  |  | SP_185_cDNA_142 |
| EF066071.1 |  |  | SP_185_cDNA_143 |
| EF066136.1 |  |  | SP_185_cDNA_144 |
| EF066074.1 |  |  | SP_185_cDNA_145 |
| EF066114.1 |  | SP145 | SP_185_cDNA_146 |
| EF066206.1 |  |  | SP_185_cDNA_147 |
| EF066134.1 |  |  | SP_185_cDNA_148 |
| EF066128.1 |  |  | SP_185_cDNA_149 |
| EF066098.1 |  |  | SP_185_cDNA_150 |
| EF066111.1 |  |  | SP_185_cDNA_151 |
| EF066112.1 |  |  | SP_185_cDNA_152 |
| EF065963.1 |  |  | SP_185_cDNA_153 |
| EF065915.1 |  |  | SP_185_cDNA_154 |
| EF065823.1 |  | SP154 | SP_185_cDNA_155 |
| Genbank accession | Sequence name in Genbank file | Sequence name for phylogenetic analysis (figure 7 in main text) | Sequence name for diversity analysis (figure 8 in main text) |
| EF065912.1 |  |  | SP_185_cDNA_156 |
| EF065822.1 |  |  | SP_185_cDNA_157 |
| EF065909.1 |  |  | SP_185_cDNA_158 |
| EF065824.1 |  |  | SP_185_cDNA_159 |
| EF065831.1 |  |  | SP_185_cDNA_160 |
| EF066038.1 |  |  | SP_185_cDNA_161 |
| EF066025.1 |  |  | SP_185_cDNA_162 |
| EF066021.1 |  |  | SP_185_cDNA_163 |
| EF066022.1 |  | SP163 | SP_185_cDNA_164 |
| EF066029.1 |  |  | SP_185_cDNA_165 |
| EF065991.1 |  |  | SP_185_cDNA_166 |
| EF066026.1 |  |  | SP_185_cDNA_167 |
| DQ183104.1 |  |  | SP_185_cDNA_168 |
| EF066024.1 |  |  | SP_185_cDNA_169 |
| EF066030.1 |  |  | SP_185_cDNA_170 |
| EF065869.1 |  |  | SP_185_cDNA_171 |
| EF065871.1 |  |  | SP_185_cDNA_172 |
| EF065904.1 |  | SP172 | SP_185_cDNA_173 |
| EF065913.1 |  |  | SP_185_cDNA_174 |
| EF065869.1 |  |  | SP_185_cDNA_175 |
| EF065905.1 |  |  | SP_185_cDNA_176 |
| EF065903.1 |  |  | SP_185_cDNA_177 |
| EF065901.1 |  |  | SP_185_cDNA_178 |
| EF066033.1 |  |  | SP_185_cDNA_179 |
| EF066034.1 |  |  | SP_185_cDNA_180 |
| EF065992.1 |  |  | SP_185_cDNA_181 |
| EF065995.1 |  | SP181 | SP_185_cDNA_182 |
| EF066027.1 |  |  | SP_185_cDNA_183 |
| EF066032.1 |  |  | SP_185_cDNA_184 |
| EF065906.1 |  |  | SP_185_cDNA_185 |
| EF065860.1 |  |  | SP_185_cDNA_186 |
| EF065829.1 |  |  | SP_185_cDNA_187 |
| EF065911.1 |  |  | SP_185_cDNA_188 |
| EF065876.1 |  |  | SP_185_cDNA_189 |
| EF065908.1 |  |  | SP_185_cDNA_190 |
| EF065861.1 |  | SP190 | SP_185_cDNA_191 |
| DQ183104.1 |  |  | SP_185_cDNA_192 |
| EF065833.1 |  |  | SP_185_cDNA_193 |
| EF065863.1 |  |  | SP_185_cDNA_194 |
| DQ183128.1 |  |  | SP_185_cDNA_195 |
| DQ183144.1 |  |  | SP_185_cDNA_196 |
| DQ183145.1 |  |  | SP_185_cDNA_197 |
| DQ183164.1 |  |  | SP_185_cDNA_198 |
| DQ183183.1 |  |  | SP_185_cDNA_199 |
| Genbank accession | Sequence name in Genbank file | Sequence name for phylogenetic analysis (figure 7 in main text) | Sequence name for diversity analysis (figure 8 in main text) |
| DQ183177.1 |  | SP199 | SP_185_cDNA_200 |
| DQ183169.1 |  |  | SP_185_cDNA_201 |
| DQ183163.1 |  |  | SP_185_cDNA_202 |
| DQ183143.1 |  |  | SP_185_cDNA_203 |
| DQ183172.1 |  |  | SP_185_cDNA_204 |
| DQ183175.1 |  |  | SP_185_cDNA_205 |
| DQ183153.1 |  |  | SP_185_cDNA_206 |
| DQ183136.1 |  |  | SP_185_cDNA_207 |
| DQ183146.1 |  |  | SP_185_cDNA_208 |
| DQ183174.1 |  | SP208 | SP_185_cDNA_209 |
| DQ183132.1 |  |  | SP_185_cDNA_210 |
| DQ183130.1 |  |  | SP_185_cDNA_211 |
| DQ183171.1 |  |  | SP_185_cDNA_212 |
| DQ183176.1 |  |  | SP_185_cDNA_213 |
| DQ183179.1 |  |  | SP_185_cDNA_214 |
| DQ183170.1 |  |  | SP_185_cDNA_215 |
| DQ183180.1 |  |  | SP_185_cDNA_216 |
| DQ183162.1 |  |  | SP_185_cDNA_217 |
| DQ183156.1 |  | SP217 | SP_185_cDNA_218 |
| DQ183142.1 |  |  | SP_185_cDNA_219 |
| DQ183139.1 |  |  | SP_185_cDNA_220 |
| DQ183148.1 |  |  | SP_185_cDNA_221 |
| DQ183129.1 |  |  | SP_185_cDNA_222 |
| DQ183150.1 |  |  | SP_185_cDNA_223 |
| DQ183140.1 |  |  | SP_185_cDNA_224 |
| DQ183165.1 |  |  | SP_185_cDNA_225 |
| DQ183138.1 |  |  | SP_185_cDNA_226 |
| DQ183135.1 |  |  | SP_185_cDNA_227 |
| DQ183178.1 |  |  | SP_185_cDNA_228 |
| DQ183141.1 |  |  | SP_185_cDNA_229 |
| DQ183134.1 |  |  | SP_185_cDNA_230 |
| DQ183147.1 |  |  | SP_185_cDNA_231 |
