# Supplementary material for: Characterization of the Highly Variable Immune Response Gene Family, He185/333, in the Sea Urchin, Heliocidaris erythrogramma
Source: PLoS One. 2014 Oct 21;9(10):e62079. doi: 10.1371/journal.pone.0062079 (PMC4204807; doi:10.1371/journal.pone.0062079)
Supplement: Table S3 — Summary of diversity analysis carried out on He185/333 and Sp185/333 sequences. Only those codons (codon#) that are positively or negatively selected are indicated in this table. The diversity analysis was carried out using three separate algorithms (SLAC, FEL and iFEL) and the table indicates whether codons are positively (+) or negatively (−) selected according to each of the algorithms. Codons are considered to be under selection (+ or −) if two or more of the analytical algorithms indicate significant selection pressure (columns entitled ‘consensus’). Blank columns specify codons that are not considered to be under significant selection by an algorithm. (DOC) [file pone.0062079.s009.doc]

**Supplemental table S3.**  **Summary of diversity analysis carried out on *He185/333* and *Sp185/333* sequences.** Only those codons (codon #) that are positively or negatively selected are indicated in this table. The diversity analysis was carried out using three separate algorithms (SLAC, FEL and iFEL) and the table indicates whether codons are positively (+) or negatively (-) selected according to each of the algorithms. Codons are considered to be under selection (+ or -) if two or more of the analytical algorithms indicate significant selection pressure (columns entitled ‘consensus’). Blank columns specify codons that are not considered to be under significant selection by an algorithm.

| ***S. purpuratus*** | | | | | ***H. erythrogramma*** | | | | |
| --- | --- | --- | --- | --- | --- | --- | --- | --- | --- |
| **Codon #** | **SLAC** | **FEL** | **IFEL** | **Consensus** | **Codon #** | **SLAC** | **FEL** | **IFEL** | **Consensus** |
| 7 | **-** | **-** | **-** | **-** | 21 | **-** | **-** | **-** | **-** |
| 11 |  | **-** | **-** | **-** | 26 | **-** | **-** | **-** | **-** |
| 18 | **-** | **-** | **-** | **-** | 40 |  | **+** | **+** | **+** |
| 22 | **+** | **+** | **+** | **+** | 48 |  | **+** | **+** | **+** |
| 34 |  | **+** | **+** | **+** | 63 | **-** | **-** | **-** | **-** |
| 35 |  | **+** | **+** | **+** | 73 | **-** | **-** | **-** | **-** |
| 36 |  | **-** | **-** | **-** | 77 | **-** | **-** |  | **-** |
| 41 |  | **+** | **+** | **+** | 89 | **-** |  | **-** | **-** |
| 46 | **-** | **-** | **-** | **-** | 109 | **-** | **-** | **-** | **-** |
| 53 | **-** | **-** |  | **-** | 111 |  | **+** | **+** | **+** |
| 58 |  | **-** | **-** | **-** | 120 |  | **-** | **-** | **-** |
| 59 |  | **-** | **-** | **-** | 124 | **+** | **+** | **+** | **+** |
| 63 |  | **-** | **-** | **-** | 151 | **-** | **-** | **-** | **-** |
| 65 | **-** | **-** | **-** | **-** | 167 | **-** | **-** | **-** | **-** |
| 67 | **-** | **-** | **-** | **-** | 189 | **-** | **-** | **-** | **-** |
| 70 | **+** | **+** | **+** | **+** | 195 | **-** | **-** | **-** | **-** |
| 73 | **+** | **+** | **+** | **+** | 206 |  | **+** | **+** | **+** |
| 74 | **-** | **-** | **-** | **-** | 210 | **-** | **-** | **-** | **-** |
| 79 | **-** | **-** | **-** | **-** | 283 | **-** | **-** | **-** | **-** |
| 80 | **-** | **-** | **-** | **-** | 293 |  | **+** | **+** | **+** |
| 83 | **-** | **-** | **-** | **-** | 327 |  | **+** | **+** | **+** |
| 84 | **-** | **-** | **-** | **-** | 333 | **-** | **-** | **-** | **-** |
| 85 | **-** | **-** | **-** | **-** | 336 | **-** |  | **-** | **-** |
| 88 | **-** | **-** | **-** | **-** | 348 | **-** | **-** | **-** | **-** |
| 89 | **-** | **-** | **-** | **-** | 358 |  | **+** | **+** | **+** |
| 90 |  | **-** | **-** | **-** | 359 | **+** | **+** | **+** | **+** |
| 92 |  | **-** | **-** | **-** |  |  |  |  |  |
| 100 |  | **+** | **+** | **+** |  |  |  |  |  |
| 101 | **-** | **-** | **-** | **-** |  |  |  |  |  |
| 105 | **-** | **-** | **-** | **-** |  |  |  |  |  |
| 108 |  | **-** | **-** | **-** |  |  |  |  |  |
| 109 |  | **-** | **-** | **-** |  |  |  |  |  |
| 110 |  | **-** | **-** | **-** |  |  |  |  |  |
| 113 | **-** | **-** | **-** | **-** |  |  |  |  |  |
| 114 | **-** | **-** | **-** | **-** |  |  |  |  |  |
| 117 |  | **-** | **-** | **-** |  |  |  |  |  |
| 124 | **-** | **-** | **-** | **-** |  |  |  |  |  |
| 130 |  | **-** | **-** | **-** |  |  |  |  |  |
| 138 |  | **-** | **-** | **-** |  |  |  |  |  |
| 140 |  | **-** | **-** | **-** |  |  |  |  |  |
| 144 |  | **-** | **-** | **-** |  |  |  |  |  |
| 145 | **+** | **+** | **+** | **+** |  |  |  |  |  |
| 156 | **-** | **-** | **-** | **-** |  |  |  |  |  |
| 164 | **-** | **-** | **-** | **-** |  |  |  |  |  |
| 165 | **-** | **-** | **-** | **-** |  |  |  |  |  |
| 174 | **-** | **-** | **-** | **-** |  |  |  |  |  |
| 180 |  | **-** | **-** | **-** |  |  |  |  |  |
| 186 |  | **+** | **+** | **+** |  |  |  |  |  |
| 187 |  | **+** | **+** | **+** |  |  |  |  |  |
| 188 |  | **+** | **+** | **+** |  |  |  |  |  |
| 238 |  | **-** | **-** | **-** |  |  |  |  |  |
| 244 | **-** | **-** | **-** | **-** |  |  |  |  |  |
| 249 | **-** | **-** | **-** | **-** |  |  |  |  |  |
| 251 | **-** | **-** | **-** | **-** |  |  |  |  |  |
| 255 | **-** | **-** | **-** | **-** |  |  |  |  |  |
| 263 |  | **-** | **-** | **-** |  |  |  |  |  |
| 270 |  | **+** | **+** | **+** |  |  |  |  |  |
| 278 | **-** | **-** | **-** | **-** |  |  |  |  |  |
| 292 |  | **-** | **-** | **-** |  |  |  |  |  |
| 308 |  | **-** | **-** | **-** |  |  |  |  |  |
| 313 |  | **-** | **-** | **-** |  |  |  |  |  |
| 335 | **-** | **-** | **-** | **-** |  |  |  |  |  |
| 342 | **-** | **-** | **-** | **-** |  |  |  |  |  |
| 345 |  | **-** | **-** | **-** |  |  |  |  |  |
| 347 | **-** | **-** | **-** | **-** |  |  |  |  |  |
| 350 | **-** | **-** | **-** | **-** |  |  |  |  |  |
| 366 | **-** | **-** | **-** | **-** |  |  |  |  |  |
| 371 | **-** | **-** | **-** | **-** |  |  |  |  |  |
| 374 | **-** | **-** | **-** | **-** |  |  |  |  |  |
| 376 | **-** |  | **-** | **-** |  |  |  |  |  |
| 411 | **-** | **-** | **-** | **-** |  |  |  |  |  |
| 423 |  | **-** | **-** | **-** |  |  |  |  |  |
| 532 | **-** | **-** | **-** | **-** |  |  |  |  |  |
| 536 |  | **-** | **-** | **-** |  |  |  |  |  |
| 541 |  | **+** | **+** | **+** |  |  |  |  |  |
| 543 |  | **+** | **+** | **+** |  |  |  |  |  |
| 550 | **-** | **-** | **-** | **-** |  |  |  |  |  |
| 566 |  | **+** | **+** | **+** |  |  |  |  |  |
| 568 |  | **-** | **-** | **-** |  |  |  |  |  |
